# Metal–N-Heterocyclic Carbene Complexes in Buchwald–Hartwig Amination Reactions

**DOI:** 10.1021/acs.chemrev.5c00088

**Published:** 2025-06-06

**Authors:** Sourav Sekhar Bera, Greta Utecht-Jarzyńska, Shiyi Yang, Steven P. Nolan, Michal Szostak

**Affiliations:** † Department of Chemistry, 67206 Rutgers University, 73 Warren Street, Newark, New Jersey 07102, United States; ‡ Faculty of Chemistry, 49602 University of Lodz, Tamka 12, Łódź 91-403, Poland; || Department of Chemistry and Center for Sustainable Chemistry, 26656 Ghent University, Krijgslaan 281, 9000 Ghent, Belgium

## Abstract

We present a comprehensive overview of the Buchwald–Hartwig amination, one of the most useful methods for C–N bond formation, mediated by NHC–transition-metal-complexes, covering the literature since 1999 (the first report on Buchwald–Hartwig amination by Nolan et al.) through December 2024. Palladium– and nickel–N-heterocyclic carbene (NHC) complexes are key contributors to Buchwald–Hartwig amination and are thoroughly discussed in this review, along with examples of cobalt and rhodium–NHC complexes. Apart from the conventional aryl/alkyl amines and aryl halides coupling, participation of versatile and challenging functional groups like pseudohalides, amides, ester, sulfoxides, unactivated aryl sulfamates, carbamates, pivalates, as well as novel electrophiles, such as aryl fluorides, methyl ethers, and silyloxyarenes, are also presented. The Reader is provided with an overview of the key role of metal–NHC complexes, their crucial role in constructing carbon–nitrogen bonds, and their importance in medicinal and materials chemistries as well as in drug discovery.

## Graphical abstract

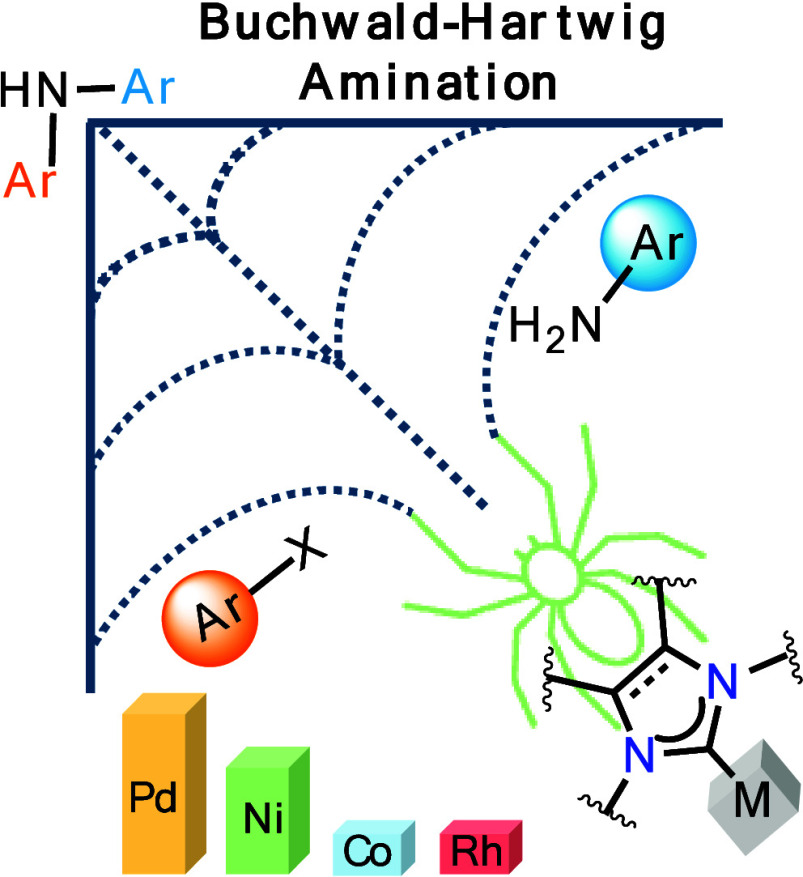

## 1 Introduction

Transition-metal-catalyzed amination of aryl halides and pseudohalides, termed the Buchwald–Hartwig amination reaction, is the most widely used C–N bond forming reaction in organic synthesis owing to the controlled nature of installing the C–N moiety and the ubiquitous presence of amines in pharmaceuticals, biological probes, agrochemicals, natural products, and organic materials.^1–15^ The use of mild reaction conditions and high catalytic efficiency have rendered the Buchwald–Hartwig amination technology the preferred methodology over the more traditional nucleophilic aromatic substitutions (S_N_Ar) and Ullmann couplings.^16–19^ In 1983, Migita reported the first example of a palladium-catalyzed C­(sp^2^)–N bond forming reaction employing aryl bromides and aminostannanes.^20^ However, the use of toxic aminostannanes and its narrow substrate scope limited applicability of this method process. In 1994, the Buchwald and Hartwig groups independently reported improved strategies for the coupling of aryl bromides with aminostannanes, where isolation of toxic and sensitive tin amides was avoided.^21,22^ In the following year, the same groups independently published a palladium-catalyzed tin-free protocol for the C–N bond-forming reaction using aryl bromides and amines.^23,24^ Subsequently, in 1997, Buchwald reported the Ni­(cod)_2_-catalyzed amination reactions of aryl chlorides.^25^ After these early reports, the use of Buchwald–Hartwig amination increased exponentially and this reaction has been quickly established as the key technology for the C–N bond coupling in both academia and industry.

The importance of this reaction is particularly evident in pharmaceutical research, where, according to a recent study by Njardarson, 82% of drugs contain at least one nitrogen heterocycle.^15^ The chart presented in Figure 1 clearly illustrates the importance of C–N cross-coupling reactions in medical chemistry, total synthesis, and materials chemistry.^12,14^ These heterocycles are now routinely installed through the Buchwald–Hartwig amination protocols.

**Figure 1:**
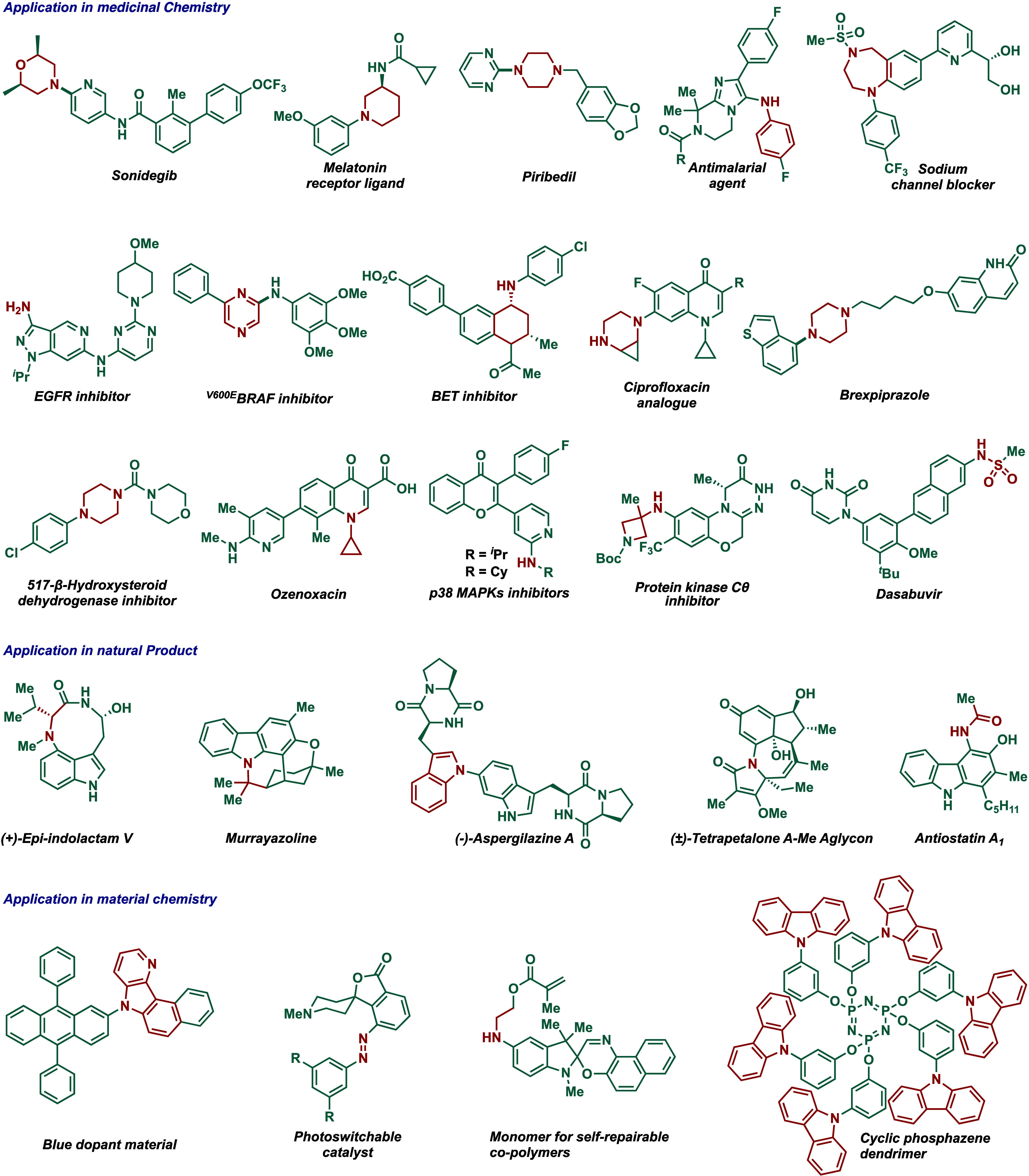
Selected applications of C–N cross-coupling reactions.

It should be clearly emphasized that the performance of Buchwald–Hartwig amination is singularly dependent on the metal supporting ligands, namely phosphines and N-heterocyclic carbenes (NHCs). Different monodentate, bidentate and biaryl phosphines, including PR_3_- or PAr_3_-type ligands, such as BINAP, Xantphos, DPEPhos, dppf, CyPF-*t*Bu, dppp, BrettPhos, RuPhos, BippyPhos, and MorDalPhos, are at present widely used for Buchwald–Hartwig amination and their performance has been the subject of several reviews.^26–35^ Simultaneously, N-heterocyclic carbenes serve as a privileged class of ancillary ligands for the Buchwald–Hartwig amination owing to their unique steric and electronic characteristics.^36–43^

Since the seminal studies of Arduengo in 1991 reporting the isolation of the free IAd carbene^44^ and of Herrmann, in 1995, on the first application of N-heterocyclic carbenes in transition-metal-catalysis,^45^ NHCs have played a prominent role in organic synthesis, ensuring stabilization of reactive metal centers.^46–54^ The major distinguishing feature of NHC ligands is their strong σ-donation to various metals, which enables difficult oxidative additions, an elementary step that is critical in cross-coupling reactions. Furthermore, the variable sterics of N-wingtips in combination with possible backbone modifications, enable a unique structural environment around the metal center. This flexible environment is critical in promoting elementary steps in cross-coupling reactions, such as transmetalation and reductive elimination.^55–70^

Importantly, the steric impact of NHC ligands is distinct from that of phosphine ligands in that cone-shaped phosphines generally expand away from the metal center and away from the coordination sphere, while umbrella-shaped NHC ligands move toward the metal center, providing a significantly different architecture than that of phosphines. In terms of steric environment, NHC ligands are significantly larger than common tertiary phosphines (percent buried volume, %V_*bur*_, [(L)­AuCl] complexes, M–L = 2.00 Å: PPh_3_, %V_*bur*_ = 27.3%; PCy_3_, %V_*bur*_ = 38.8%; P*t*Bu_3_, %V_*bur*_ = 43.9% vs IMes, %V_*bur*_ = 36.5%; IPr, %V_*bur*_ = 45.4%; IPr*, %V_*bur*_ = 50.4%)^71,72^ The unique umbrella-type architecture and large steric pressure of NHCs enable unprecedented opportunities in tailoring properties of metal centers and catalytic cycles. Furthermore, fragments of the NHCs are highly anisotropic and rotationally flexible around the metal–carbene bonds, enabling variations of the steric hindrance of bulky NHC ligands.^73–75^

In terms of electronic properties, NHC ligands combine three effects: (1) L→M σ-donation, (2) M→L π*-backbonding, (3) L→M π-donation. L–M π-donation contributes about 15–20% to the overall electronic contribution.^41^ Hence, σ-donation ability is the main component for metal–carbene bond stability. NHCs are generally better σ-donors than the most basic phosphines. The overall electronic contribution to the metal center, generally quantified through the Tolman Electronic Parameter, TEP, indicates a higher electronic contribution to the metal centers in NHCs than in phosphines (e.g., PPh_3_, TEP = 2068.9 cm^–1^; P^*i*^Pr_3_, TEP = 2059.2 cm^–1^; PCy_3_, TEP = 2056.4 cm^–1^ vs IMes, TEP = 2050.7 cm^–1^; IPr, TEP = 2051.5 cm^–1^; ICy, TEP = 2049.6 cm^–1^) (Table 1). Thus, a stronger metal–NHC bond is expected than in the corresponding phosphine–metal complexes.^76–83^

**Table 1:** Comparison of Pd/Phosphine and Pd/NHC Ligand Systems by Trudell^a^

entry	Pd/ligand	time (h)	yield (%)
1	Pd(OAc)_2_/PPh_3_	42	37
2	Pd(OAc)_2_/dppp	40	25
3	Pd(OAc)_2_/dppb	40	11
4	Pd_2_(dba)_3_/P(*o*-Tol)_3_	36	51
5	Pd_2_(dba)_3_/dppf	36	66
6	Pd_2_(dba)_3_/DiIMes·HCl	36	61
7	Pd_2_(dba)_3_/DiIPr·HCl	36	67

a dppp = 1,3-bis­(diphenylphosphino)­propane, dppb = 1,4-bis­(diphenylphosphino)­butane, dppf = 1,1′-bis­(diphenylphosphino)­ferrocene.

In 1999, Nolan and co-workers reported the first example of Pd(0)/NHC-catalyzed Buchwald–Hartwig amination using a combination of Pd_2_(dba)_3_ and IPr·HCl.^84,85^ In 2001, Fort and co-workers reported an in situ-formed Ni(0)/SIPr catalyst system for the Buchwald–Hartwig amination.^86^ These reports triggered numerous studies on catalyst diversification and reaction optimization, and a variety well-defined Pd–NHC and Ni–NHC complexes have been developed in this area of research (Figure 2).

**Figure 2:**
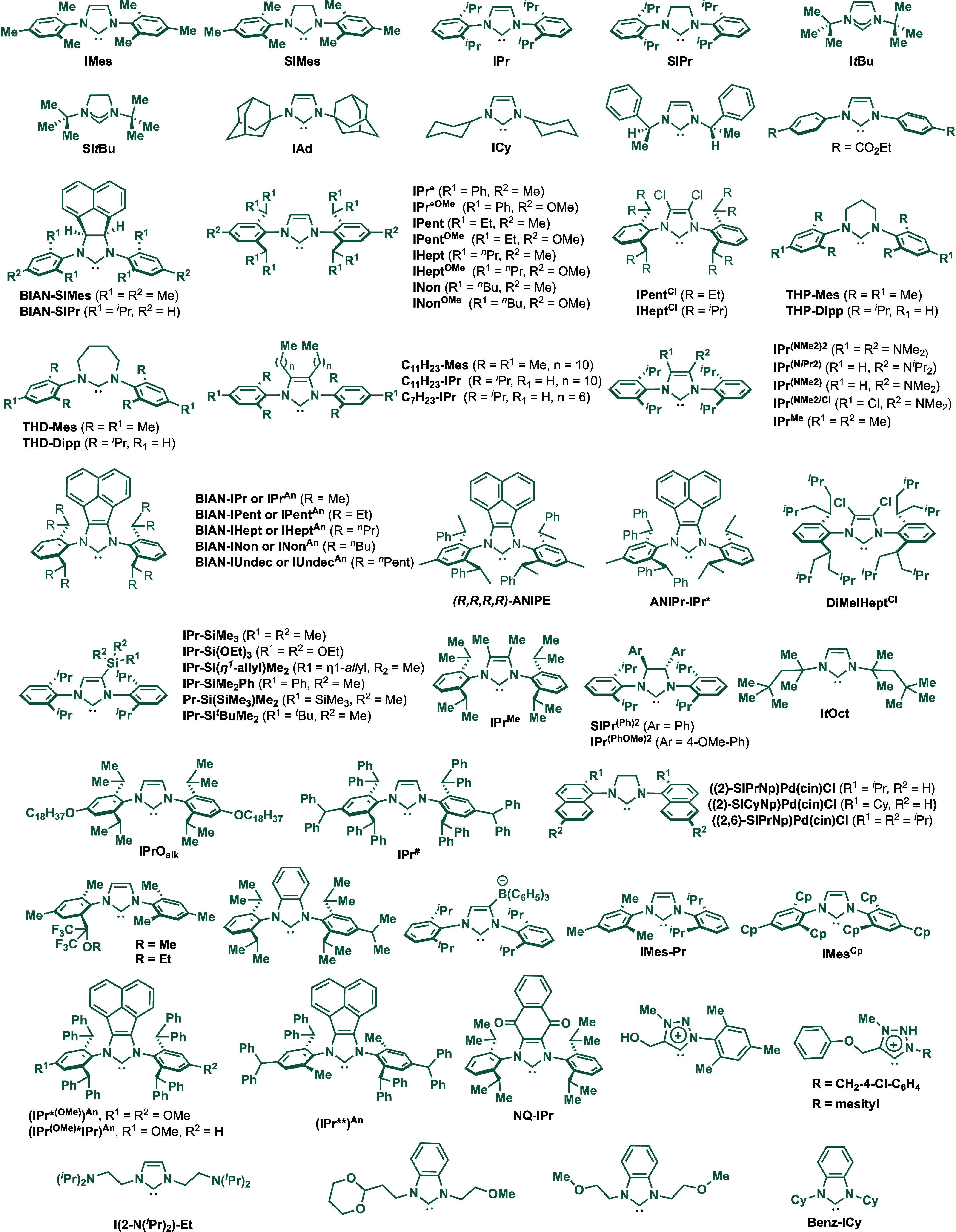

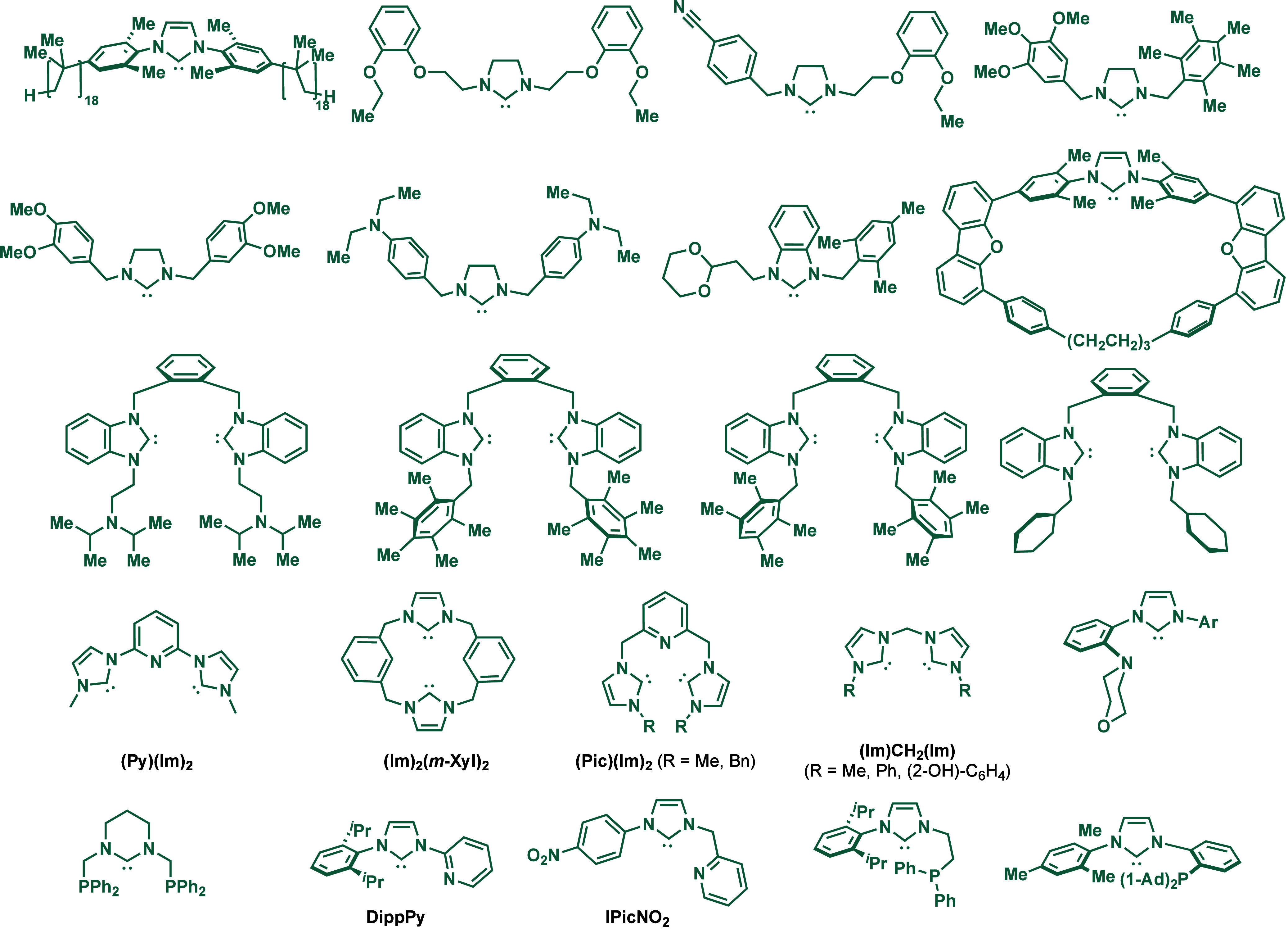
Common N-heterocyclic carbene ligands in palladium- and nickel-catalyzed Buchwald–Hartwig amination.

More recently, Buchwald–Hartwig amination of amides through N–C­(O) acyl bond cleavage has been developed.^87^ These reactions provide valuable access to amides vs amines by a mild transition-metal-catalyzed cross-coupling.

As the research in the field progressed, a wide variety of ligand systems have been developed to enhance the catalytic efficiency of Pd- and Ni-catalysts under increasingly milder reaction conditions. A variety of well-defined Pd–NHC precatalysts based on palladacycles, π-allyl-coordination, heterocycle stabilization and acetylacetonate stabilization have been developed. In these cases, the stability of the precursors and the ease of activation to the catalytically active monoligated species, [Pd(0)–NHC], are the fundamental criteria for catalyst development in this reaction. Compared to the catalyst systems relying on in situ mixing of palladium precursor and NHC ligand, the use of well-defined palladium precatalysts permits to significantly lower the catalyst loading and reaction temperature.

In terms of mechanism, after the initial activation, the active catalyst, Pd(0)/Ni(0)–NHC, promotes the oxidative addition step of the aryl halide (Figure 3).^41^ The subsequent amine coordination and deprotonation in the presence of a base result in the formation of the metal–amino complex. Finally, reductive elimination takes place to provide the amination product and regenerate the active M(0)–NHC species. In other mechanistic scenarios, a different mechanistic cycle M­(I)-M­(III) could also be proposed (see Sections 2.4 and 3.4), and more mechanistic investigations are currently underway to establish this catalytic cycle, which eventually could expand the field in other directions. For the examples of Co and Rh-catalyzed Buchwald–Hartwig reaction (see Sections 4 and 5), we expect Co(0)–Co­(II) and Rh­(I)–Rh­(III)-catalytic cycle.

**Figure 3:**
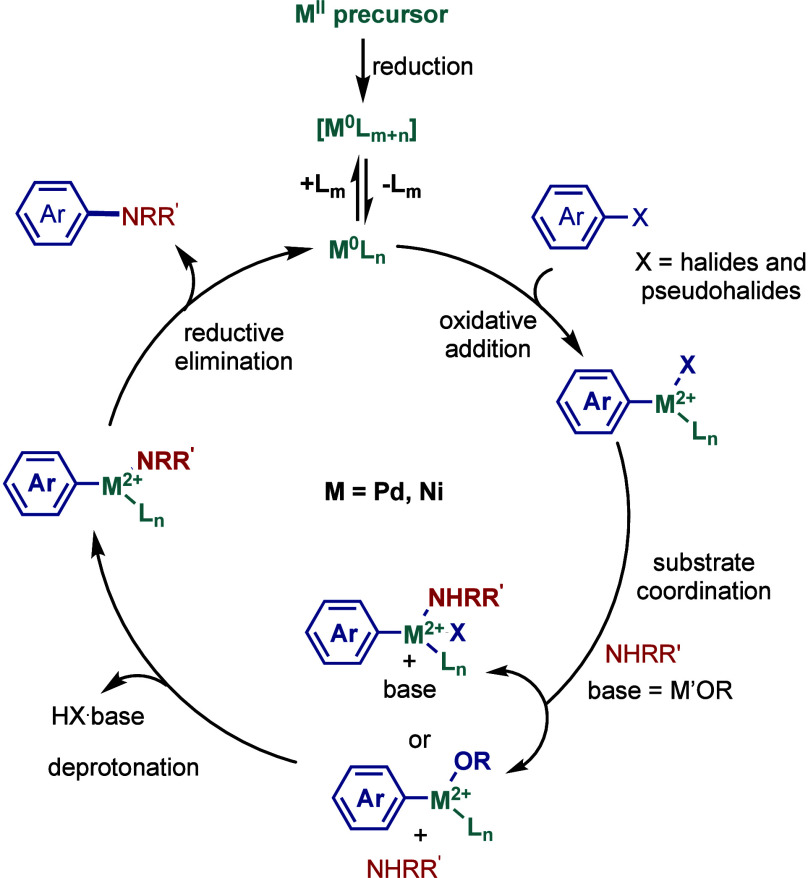
Mechanism of the Buchwald–Hartwig amination.

Even though since 1995 transition-metal-catalyzed reactions enabled by metal–NHC complexes have become indispensable in organic synthesis and major advances have been reported, a comprehensive review on Buchwald–Hartwig amination methods enabled by NHC ligands has not yet appeared. In this contribution, we provide a comprehensive survey of Buchwald–Hartwig amination reactions promoted by NHC ligands. The review focuses on reactions mediated by palladium, which historically has been the most important metal for both academic and industrial cross-coupling reactions, and includes emerging metals, such as nickel. The review covers the literature since the first report on the Buchwald–Hartwig amination in 1999 through December 2024 and provides the Reader with an overview of the remarkable advances that have taken place in the last 25 years. The review is categorized by the type of metal and the type of ligand complexes that are used for Buchwald–Hartwig amination and further categorized by classes of substrates that undergo the amination process. In particular, the review focuses on the key role of metal–NHC complexes in constructing carbon–nitrogen bonds, which represent the most important class of carbon–heteroatom bonds in organic synthesis, medicinal chemistry, and drug discovery. For simplification, we abbreviate ‘Buchwald–Hartwig amination’ as ‘BHA reaction’ onward.

## 2 Palladium–NHC Complexes

### 2.1 In Situ-Formed Pd(0)–NHC Complexes

In 1999, Nolan and co-workers reported the first application of N-heterocyclic carbene ligands in aryl amination (Scheme 1).^84^ The reaction involved cross-coupling of aryl halides with both acyclic primary and secondary alkylamines. The combination of Pd_2_(dba)_3_ and NHC·HCl salt under basic conditions (KO^*t*^Bu) formed Pd(0)–NHC complex. The role of NHC ligands was described as 2-fold in that both steric and electronic effects worked together to facilitate the cross-coupling. First, the strong σ-electron donor properties of the carbene assisted the activation of aryl chlorides through oxidative addition to Pd(0). Second, the steric bulk of the NHC wingtips accelerated the reductive elimination step, facilitating the regeneration of Pd(0). Among different NHC ligands screened, the previous thermochemistry studies^88^ showed that ITol is the best electron donor (ITol > IMes ≈ IXy > IPr); however, IPr was the bulkiest ligand (IPr > IMes ≈ IXy > ITol), and it worked best for this reaction (IPr = 98%, IMes = 22%, IXy = 11%, ITol <5%). In terms of substrate scope, a wide range of electronically- and sterically diverse substrates worked well under the reaction conditions. Furthermore, aryl iodide and aryl bromides reacted at room temperature, highlighting the exceedingly mild operating conditions of this early catalyst system.

**Scheme 1:**
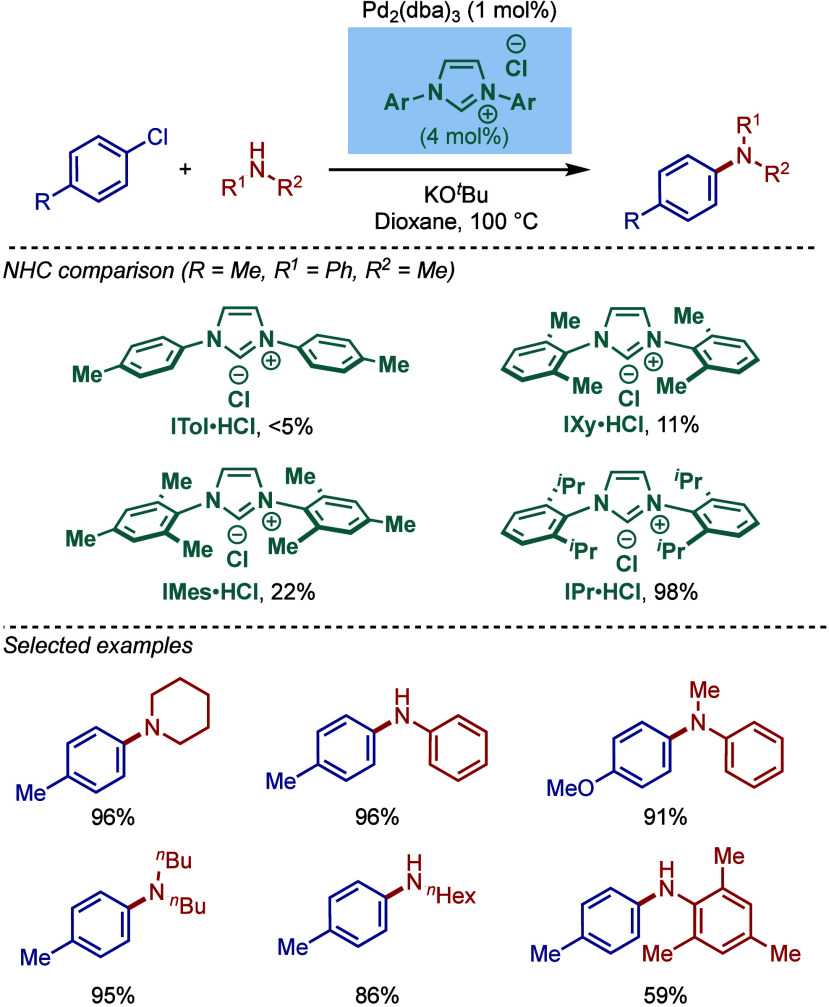
Pd­(0)/NHC-Catalyzed BHA Reaction of Aryl Halides with Primary and Secondary Amines by Nolan

In the following year, Hartwig and co-workers reported an amination protocol for the cross-coupling of aryl chlorides at room temperature using saturated imidazolidene carbene, SIPr, and Pd­(dba)_2_ as the precursor (Scheme 2).^89^ The dimeric precatalyst Pd_2_(dba)_3_ was found to be equally effective as Pd­(dba)_2_, while the reaction rate using Pd­(OAc)_2_ proved slower. Using milder bases than NaO^*t*^Bu, such as Cs_2_CO_3_ and K_3_PO_4_, was ineffective for this reaction. This protocol worked efficiently with a variety of amines and unactivated chloroarenes. However, sterically hindered secondary amines, such as diphenylamine and dicyclohexylamine, proved unreactive even at 70 °C.

**Scheme 2:**
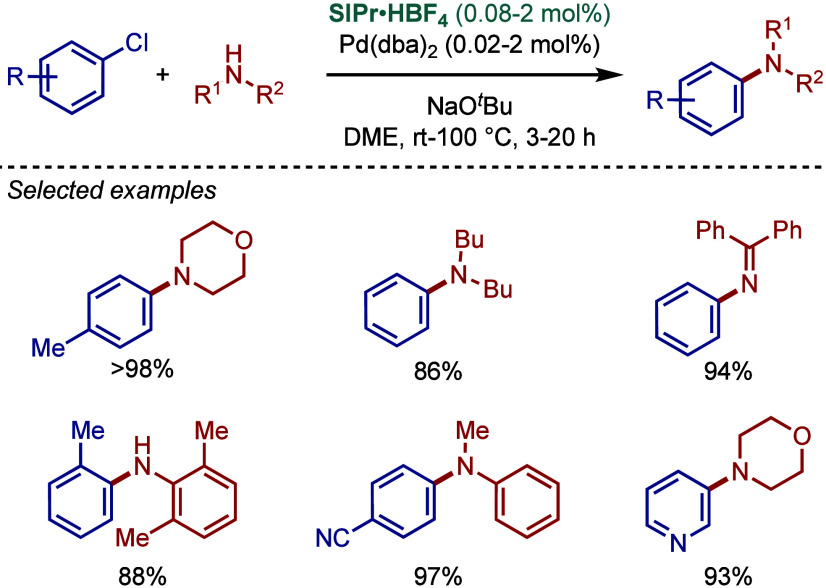
Pd­(0)/SIPr-Catalyzed Room Temperature BHA Reaction of Chloroarenes by Hartwig

In 2001, Trudell and co-workers demonstrated a convenient route for constructing a wide range of N-aryl-substituted-7-azabicyclo[2.2.1]­heptane derivatives using an in situ-generated Pd(0)–NHC catalyst system based on bisimidazolium precursors (Scheme 3).^90^ The optimized conditions utilized NaO^*t*^Bu as base and dioxane as a solvent. A comparative study between different phosphines and NHC ligands, such as PPh_3_, dppb, dppf, P­(*o*-Tol)_3_, mesityl bisimidazolium salt (DiIMes·HCl, Table 1, entry 6), and 2,4,6-triisopropylphenyl bisimidazolium salt (DiIPr·HCl, Table 1, entry 7), showed that NHCs were generally superior to phosphines. While dppf provided a similar yield, this ligand required higher loading. This coupling has significantly shortened the synthesis of the product N-aryl-7-azabicyclo[2.2.1]­heptanes, which represent important motifs in drug discovery.

**Scheme 3:**
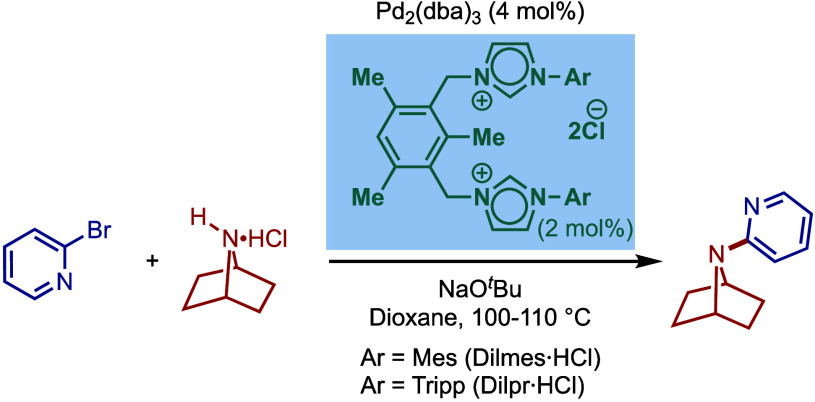
Pd­(0)/Bisimidazol-2-ylidene-Catalyzed BHA Reaction of Aryl Halides with *7-*Azabicyclo­[2.2.1]­heptane by Trudell

In the same year, Nolan and co-workers reported the first Pd/NHC-catalyzed Buchwald–Hartwig protocol for the cross-coupling of aryl bromides and chloride with benzophenone imines and indoles (Scheme 4).^91^ The protocol for coupling of imines involves a combination of Pd_2_(dba)_3_ with IPr·HCl in the presence of KO^*t*^Bu as a base in dioxane. Various electronically diverse aryl halides were found to react efficiently to furnish the corresponding imine products in excellent yields. These imines can be readily hydrolyzed to access primary amines. Furthermore, the Buchwald–Hartwig cross-coupling of indoles was found to be effective using Pd­(OAc)_2_/SIPr·HCl in the presence of NaOH as a base in dioxane at 100 °C. Interestingly, the Pd_2_(dba)_3_/IPr·HCl combination was not effective. This led to a comparative study of various NHC ligands, where imidazolin-2-ylidene, SIPr, gave the best yield (ITol = 0%, ICy = 30%, SIMes = 66%). The authors concluded that a stronger base, NaOH, was required to generate the free carbene since backbone saturated imidazolin-2-ylidenes, such as SIPr, as more σ-donating than imidazol-2-ylidenes, such as IPr. This protocol afforded excellent yields with a range of electronically varied substrates, while a sterically hindered bromide converted with slightly lower efficiency. From a mechanistic standpoint, the authors proposed that Pd­(OAc)_2_ is reduced to Pd(0) in the presence of a base, which is followed by oxidative addition in analogy to the previous Pd(0)/NHC catalytic systems.

**Scheme 4:**
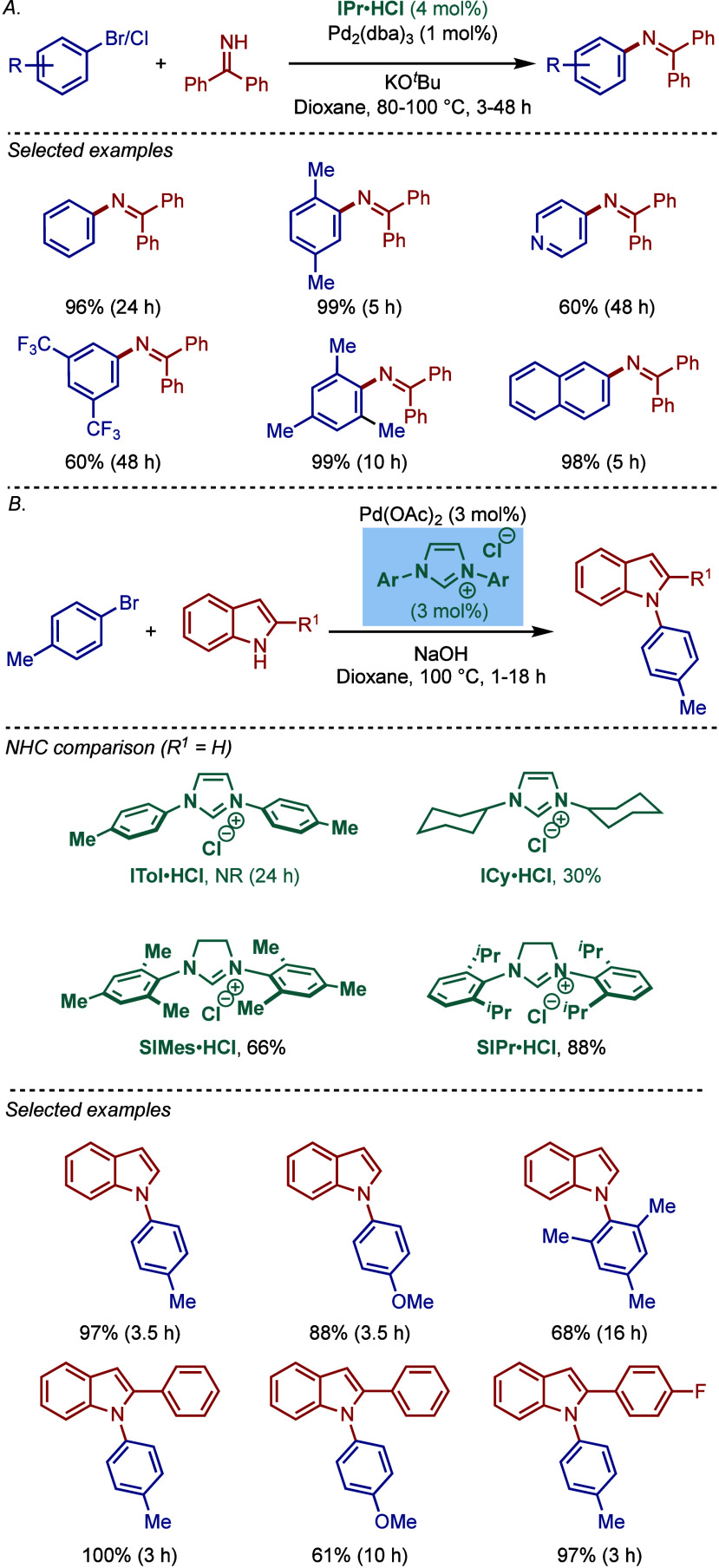
Pd­(0)/NHC-Catalyzed BHA Reaction of Aryl Halides with Imines and Indoles by Nolan

An in situ-formed heterogeneous catalytic system for the Buchwald–Hartwig cross-coupling of aryl bromides using Pd/Al_2_O_3_ and IPr·HBF_4_ in the presence of KO^*t*^Bu in toluene at 110 °C was reported by Glorius (not shown).^92^ The authors proposed that the NHC ligand lowered the bromobenzene activation barrier by coordinating to the metal nanocluster.

In 2007, Yang and co-workers reported the synthesis of triarylamines by the cross-coupling of aryl chlorides and bromides using a Pd_2_(dba)_3_·CHCl_3_/NHC·HCl catalyst system in the presence of KO^*t*^Bu as a base (Scheme 5).^93^ A comparative study showed that imidazol-2-ylidene-based, IPr, gave higher yields than its saturated imidazolin-2-ylidene congener, SIPr. Interestingly, triarylamine products could be accessed through either monoamination of diarylamines or diamination of primary aryl amines. The degree of N-arylation is controlled by the quantity of base used under the reaction conditions. With the same catalyst loading, 2.6 equiv of KO^*t*^Bu promoted monoarylation, while a large excess of base (8 equiv of KO^*t*^Bu relative to aniline) resulted in diarylation. It is important to note that electron-rich aryl halides resulted in lower yields.

**Scheme 5:**
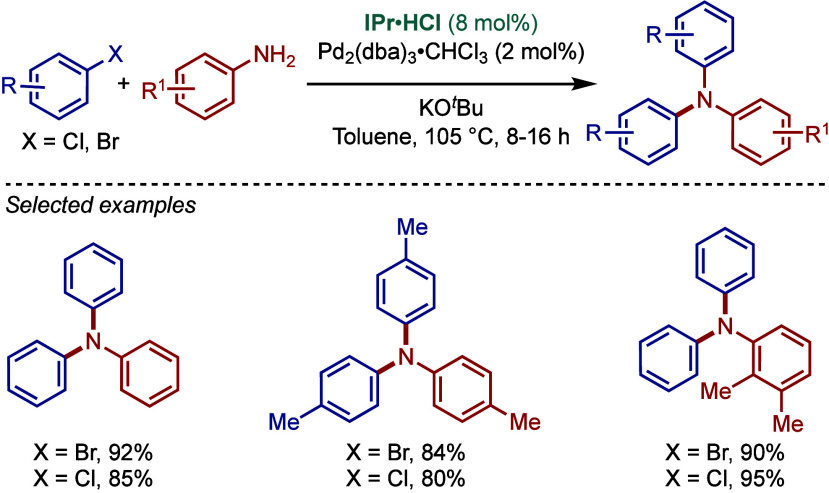
Pd­(0)/IPr-Catalyzed Synthesis of Triarylamines by BHA Reaction by Yang

In 2010, Chen and co-workers reported a Buchwald–Hartwig cross-coupling as part of their protocol to access unsymmetrical arenes (Scheme 6).^94^ They found that Pd_2_(dba)_3_/IPr·HCl as a catalyst in the presence of KO^*t*^Bu as a base in dioxane worked well for the amination of substituted chlorobiphenyls to access important nitrogen-containing pharmaceutical scaffolds.

**Scheme 6:**
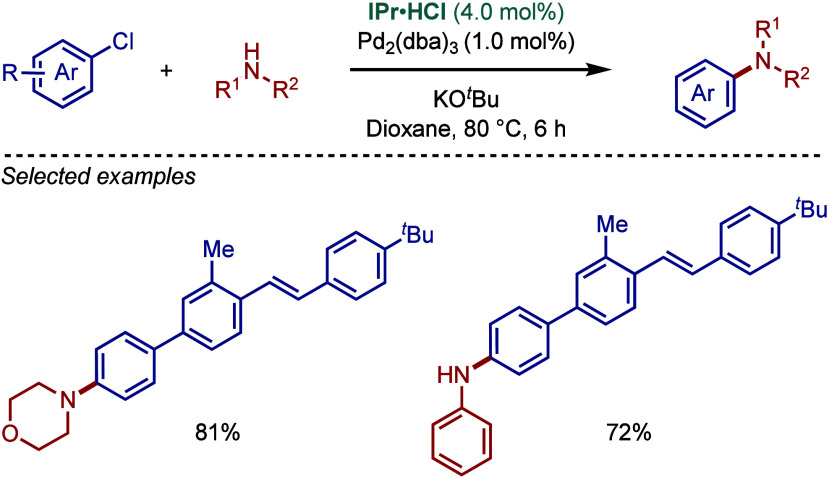
Pd­(0)/IPr-Catalyzed BHA Reaction of Substituted Chlorobiphenyls by Chen

In 2004, Fort, Schneider, and co-workers developed a one-pot tandem inter/intramolecular amination of aryl chlorides for the synthesis of N-arylated heterocycles using the Pd­(OAc)_2_/SIPr·HCl catalyst system in the presence of NaO^*t*^Bu as base (Scheme 7).^95^ This inventive method allowed for the rapid access to N-arylated 5-, 6-, and 7-membered heterocycles, such as indolines, tetrahydroquinolines, benzazepines, benzoxazines, and benzoxazepines. This protocol was also compatible with electronically deactivated and sterically hindered substrates. The coupling of 3-chloropyridine was comparatively slower due to a competing coordination of the pyridine nitrogen to palladium, thereby inhibiting the amination reaction. The authors proposed a mechanistic pathway involving the reduction of Pd­(OAc)_2_ to Pd(0) and an in situ formation of a monoligated Pd(0)–SIPr active catalyst to enable the oxidative addition step.

**Scheme 7:**
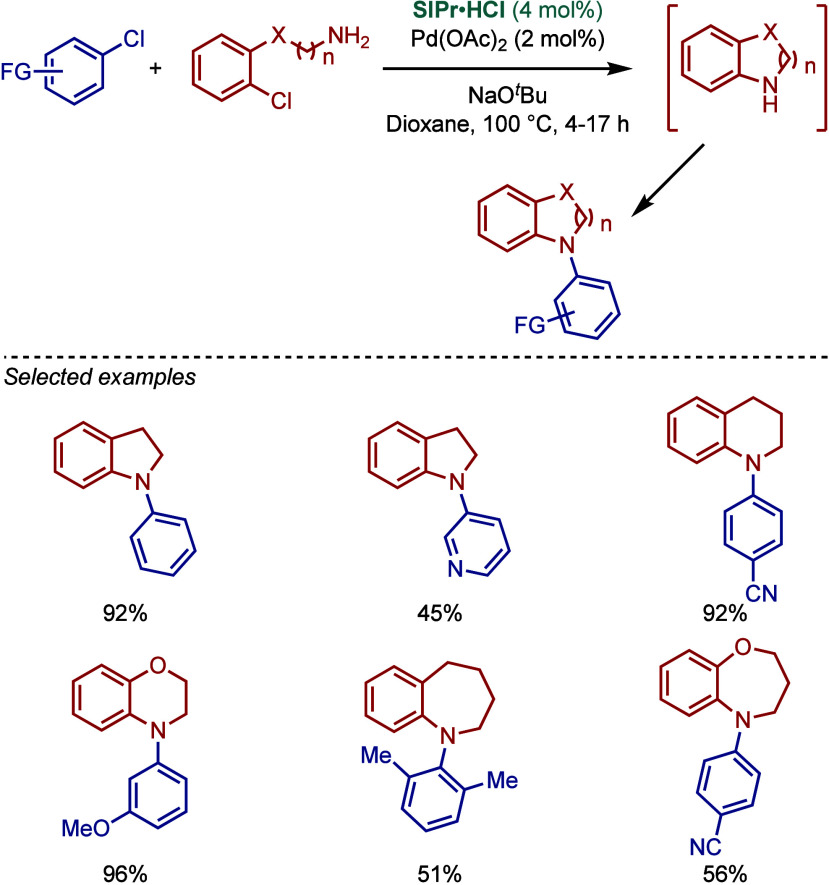
Pd/SIPr-Catalyzed Synthesis of N-Arylated Heterocycles by a Tandem Inter/Intramolecular BHA Reaction by Fort and Schneider

In 2005, Ackermann and co-workers reported a tandem one-pot BHA reaction protocol for the synthesis of functionalized indoles from *o*-alkynylhaloarenes catalyzed by the Pd­(OAc)_2_/IPr·HCl system (Scheme 8a).^96^ Significantly, this reaction is distinguished by the fact that in addition to the usual *tert*-butoxide base, KO^*t*^Bu, the less expensive, less toxic and weaker phosphate base, K_3_PO_4_, was also effective for this reaction, thus permitting to considerably expand the functional group tolerance. A broad variety of alkyl and arylalkynes and amines were efficiently reacted to provide the 2-substituted indole derivatives in excellent yields.

**Scheme 8:**
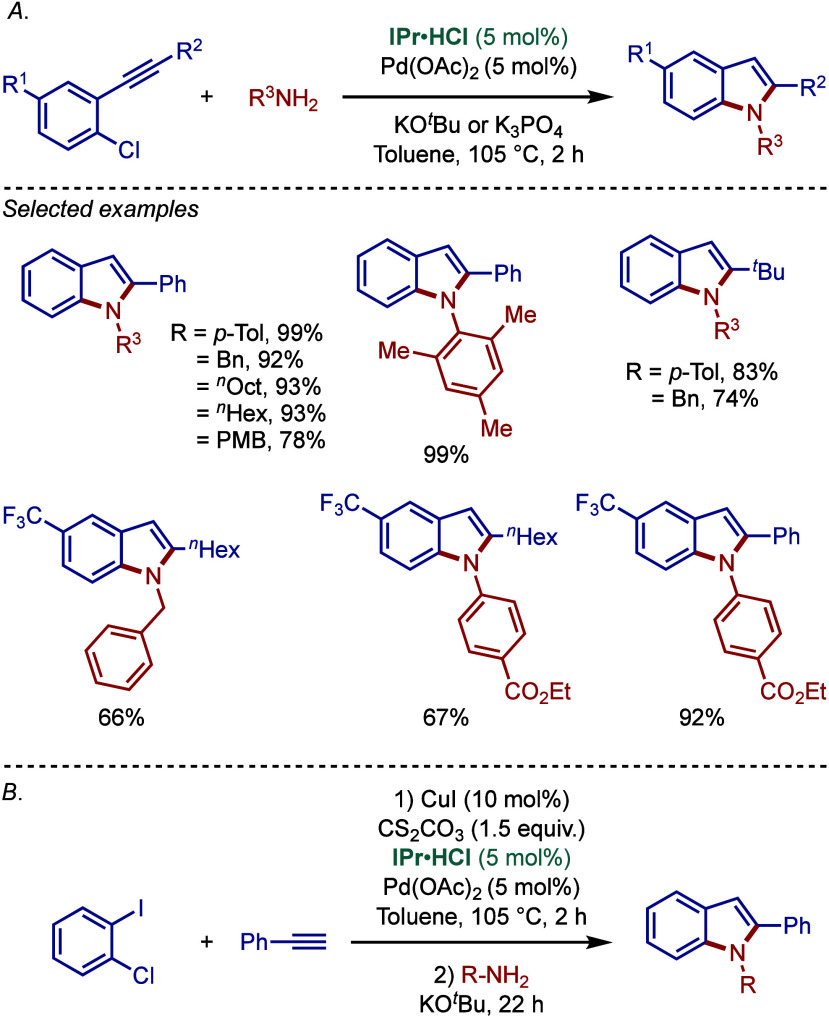
Pd/IPr-Catalyzed Synthesis of Indoles from *o*-Alkynylhaloarenes and *o-*Dihaloarenes by Ackermann

In the same year, Ackermann and co-workers also reported a BHA reaction approach to indoles using Pd­(OAc)_2_/IPr·HCl by exploiting a three-component coupling of readily accessible *o*-dihaloarenes, terminal alkynes and differently substituted amines (Scheme 8b).^97^ First, in the presence of CuI and Cs_2_CO_3_, Sonogashira coupling took place to afford *o*-alkynylhaloarenes, which then underwent the BHA reaction to furnish the indole products. Interestingly, imidazolin-2-ylidenes, such as SIPr, were less effective for this tandem coupling, revealing a subtle electronic effect of the N-heterocyclic ligand.

In 2009, Ackermann and co-workers reported the synthesis of challenging sterically hindered N-substituted indoles by using a combination of Pd­(OAc)_2_/IPr·HCl and KO^*t*^Bu (Scheme 9).^98^ Importantly, this methodology allowed to successfully install different sterically hindered groups at the N-position of indoles, such as mesityl, 2,6-diisopropylaniline, adamantyl, *tert*-butyl, and neopentyl. It is worth noting that the prenyl group, which is found in fungal natural products^99^ and exhibits promising antitumor properties could also be installed using this protocol.

**Scheme 9:**
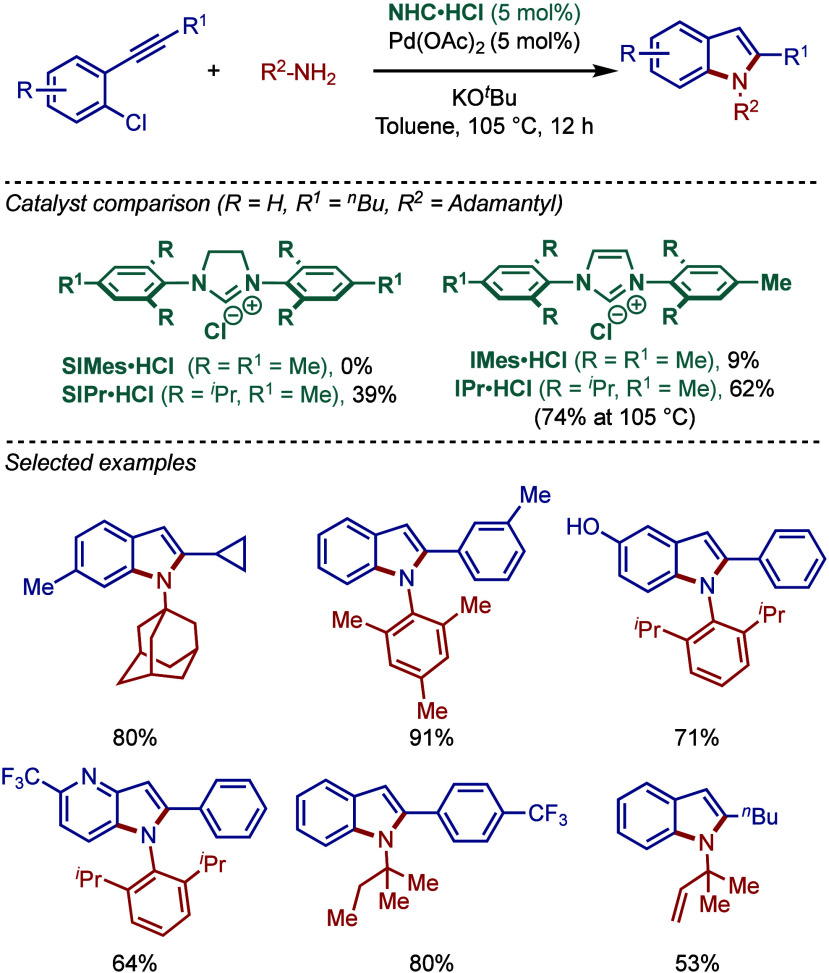
Pd/IPr-Catalyzed Synthesis of Sterically-Hindered Indoles by Ackermann

Yang and Mao employed N-2-pyridyl-functionalized tetrahydropyriidinium salts as precursors for the palladium-catalyzed BHA reaction of heteroaryl halides and heterocyclic amines under microwave irradiation conditions (Scheme 10a).^100^ The optimized reaction conditions utilized Pd­(OAc)_2_ along with the 6-membered NHC-ligand and KO^*t*^Bu as a base in DME. A series of 6-membered NHC-ligands, as well as bridged bis-tetrahydropyrimidinium salts with different linkages, were tested.

**Scheme 10:**
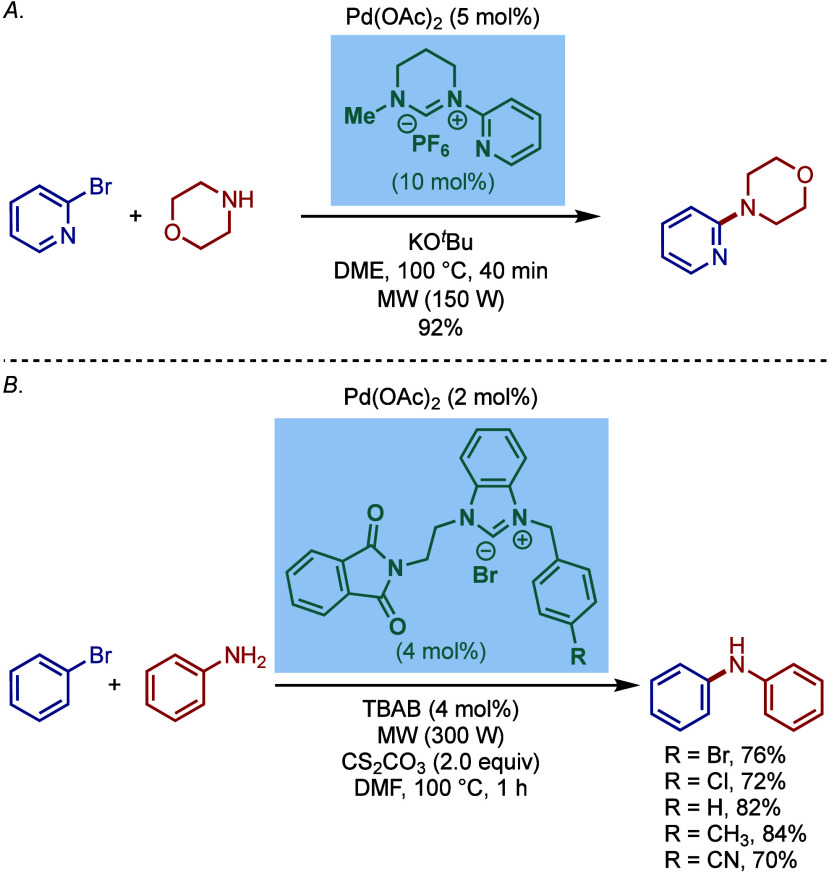
Pd/NHC-Catalyzed Microwave Assisted BHA Reaction of Aryl Halides: a) Yang and Mao, b) Küçükbay

Furthermore, another microwave-assisted Buchwald–Hartwig cross-coupling was reported by Küçükbay and co-workers using unsymmetrical benzimidazolium salts bearing N-phthalimido-ethyl and N-benzyl groups as precursors under palladium catalysis (Scheme 10b).^101^ Importantly, this protocol featured a mild cesium carbonate base in the presence of TBAB as a phase transfer catalyst in DMF as a solvent.

A related N-2-pyridyl-functionalized N-heterocyclic carbene ligand was also reported by Chen and co-workers for the palladium-catalyzed Buchwald–Hartwig diarylation of primary aromatic amines with 2-halobenzothiazoles (Scheme 11).^102^ Interestingly, a phosphine-based Xantphos provided the monoarylation product.

**Scheme 11:**
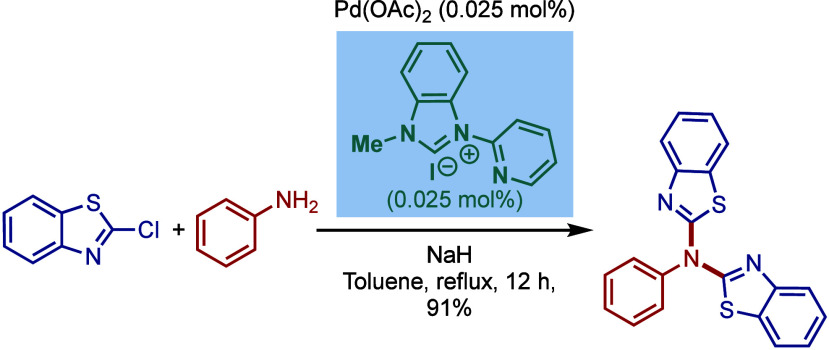
Pd/NHC-Catalyzed Buchwald–Hartwig Diarylation of Anilines with 2-Halobenzothiazoles by Chen

In 2013, Stradiotto and co-workers developed a new class of catalysts featuring a mixed phosphine/NHC scaffold and evaluated its utility in a room-temperature palladium-catalyzed BHA reaction (Scheme 12a).^103^ Both the NHC and phosphine fragments were sterically- and electronically diversified using aryl, mesityl, cyclohexyl, and adamantyl groups. The bulkiest ligand featuring the combination of a bis­(1-adamantyl)­phosphine donor group and N-Mes imidazolium wingtip showed the most promising activity. This ligand showed excellent reactivity in the monoarylation of amines, including 1° alkyl- and arylamines, as well as 2° dialkylamines.

**Scheme 12:**
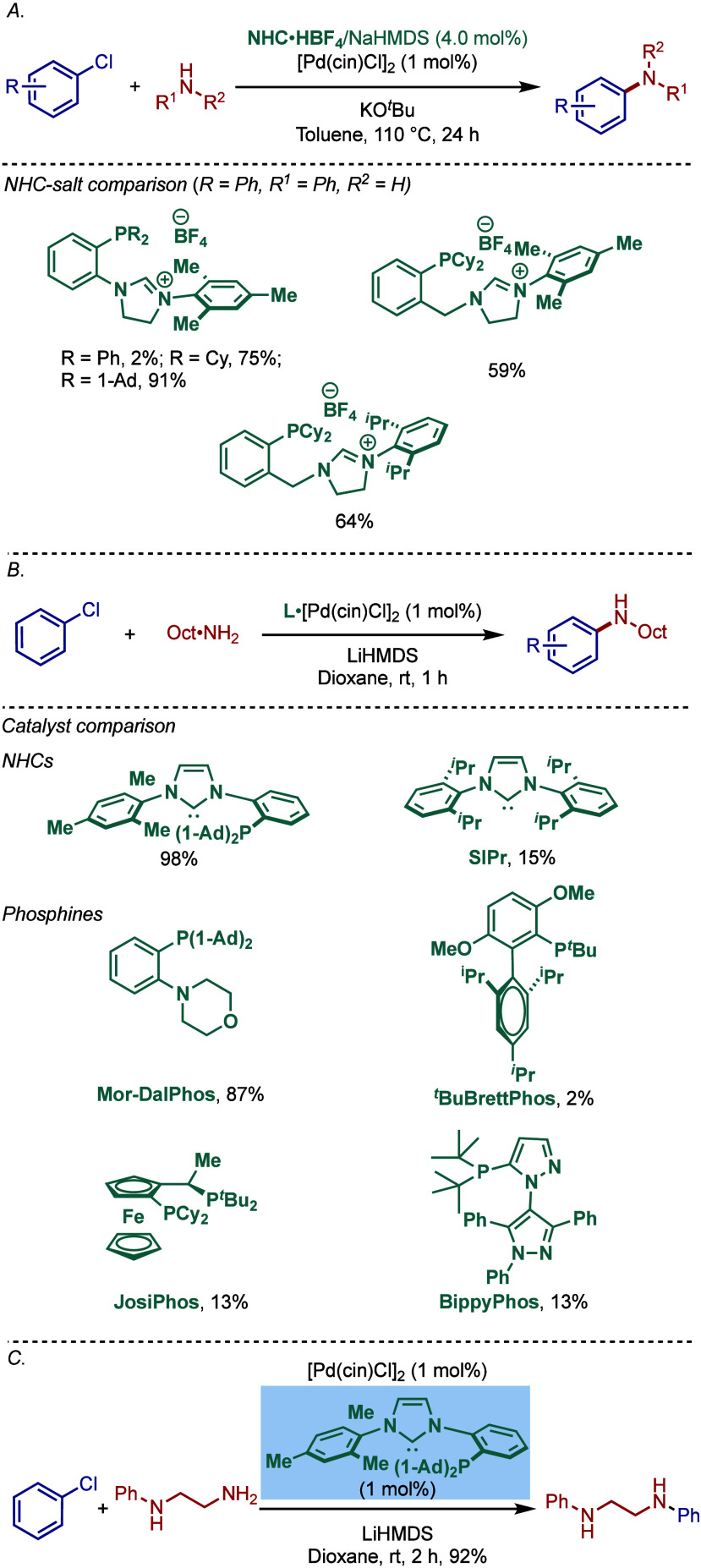
Pd/NHC-Catalyzed BHA Reaction using Mixed NHC/Phosphine Ligands by Stradiotto

Furthermore, a comparative study with different NHC and phosphine ligands (MorDalphos, *t*BuBrettphos, Joshiphos, Bippyphos) showed that the mixed phosphine/NHC ligand was the most effective for the BHA reaction of chlorobenzene with octylamine (Scheme 12b). Moreover, the comparative study between 1° and 2° amines demonstrated that 1° amines are preferentially coupled (Scheme 12c).

It is also worth noting that more recently, Ghadwal reported highly reactive sterically hindered C5–mesoionic carbene ligands (super iMICs) for the Buchwald–Hartwig cross-coupling of chlorotoluene under palladium catalysis at room temperature (Scheme 13).^104^ An evaluation of ligands showed that S-iMIC^DMP^ was vastly superior to its C2–imidazolium and abnormal C4–imidazolium congeners, such as IPr and iMIC^Ph^.

**Scheme 13:**
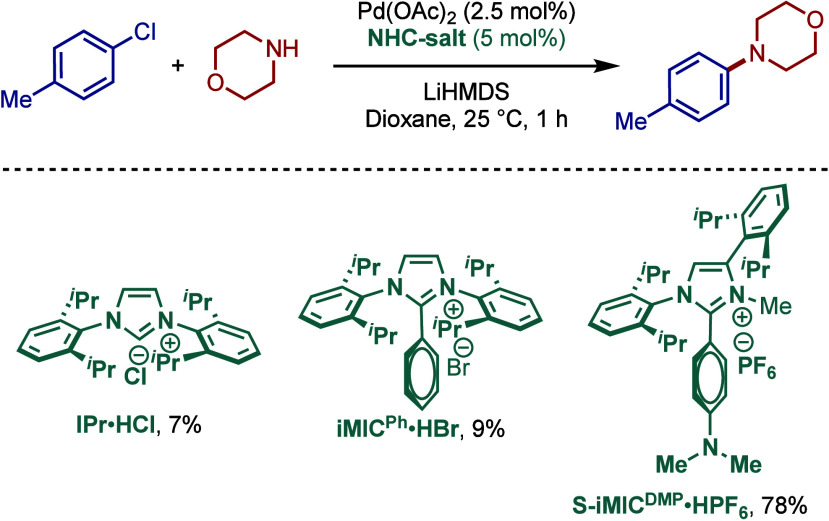
Pd/S-iMIC^DMP^-Catalyzed BHA Reaction by Ghadwal

### 2.2 Well-Defined Pd(0)–NHC Complexes

#### 2.2.1 [Pd­(NHC)_2_] Complexes

After the first report of well-defined two-coordinated palladium(0) N-heterocyclic carbene complexes by Herrmann in 2000,^105^ in 2001, Caddick, Cloke, and co-workers reported similar [Pd­(NHC)_2_] complexes for the Buchwald–Hartwig cross-coupling of aryl halides using KO^*t*^Bu as a base (Scheme 14a).^106^ These authors succeeded in the synthesis of unsymmetrical bis­(NHC)–palladium complexes by ligand exchange of [Pd­(NHC)_2_], which was an advance from the symmetrical analogues prepared by Herrmann. Subsequently, the same authors reported a modified protocol to synthesize symmetrical bis­(I*t*Bu)–palladium from {Pd­(η^3^-C_4_H_7_)­Cl}_2_ and demonstrated high reactivity in the BHA reaction of chlorotoluene (Scheme 14b).^107^

**Scheme 14:**
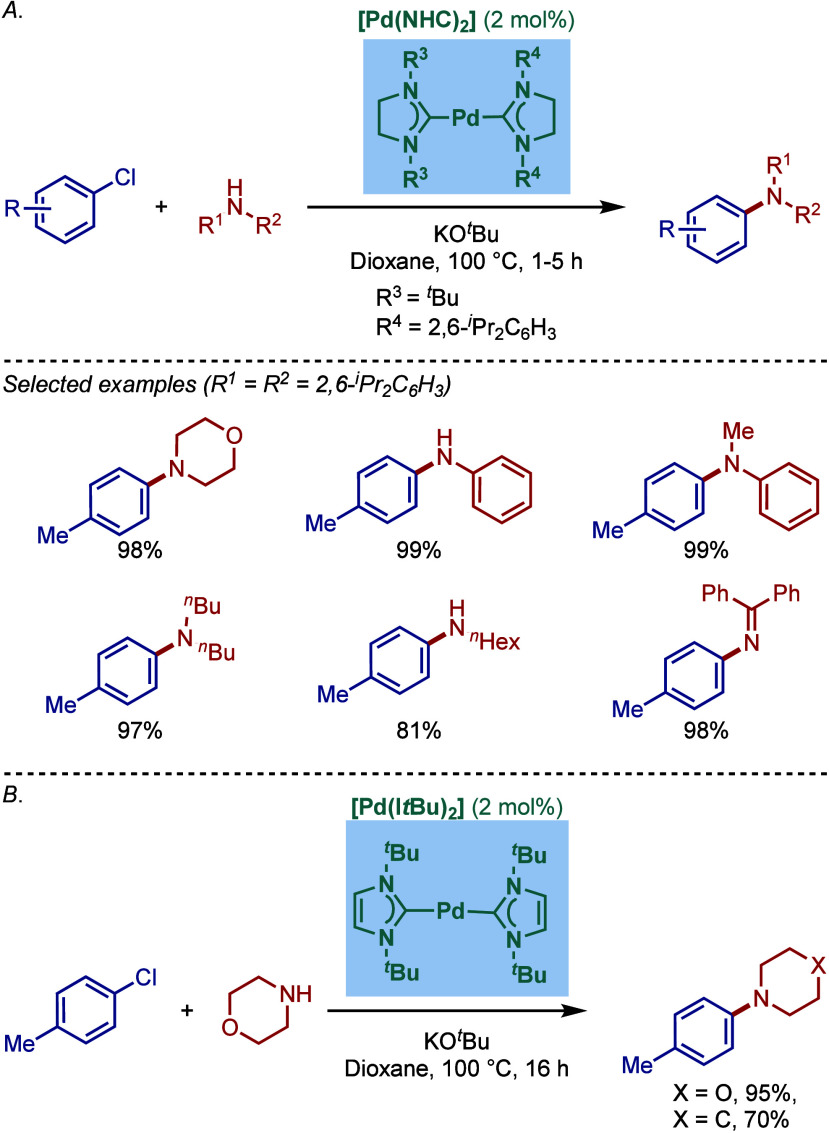
Well-Defined [Pd­(NHC)_2_]-Catalyzed BHA Reaction of Aryl Halides by Caddick and Cloke

In 2005, they reported a family of sterically- and electronically distinct two-coordinate [Pd­(NHC)_2_] complexes, including [Pd­(IPr)_2_], [Pd­(SIPr)_2_], [Pd­(I*t*Bu)_2_], and [Pd­(SI*t*Bu)_2_], and evaluated their reactivity in the BHA reaction of 4-chlorotoluene (Scheme 15).^108,109^ Interestingly, this comparison study revealed that imidazolin-2-ylidene-based [Pd­(SIPr)_2_] was the best catalyst. This study further suggested that the *tert*-butyl substitution does not offer enough steric-protection around palladium and that the more σ-donating NHC scaffold is preferred. Furthermore, a Pd(0) precursor, Pd_2_(dba)_3_ in the presence of imidazolium salt was also reactive under similar Buchwald–Hartwig conditions. From a practical standpoint, it is worth mentioning that these [Pd­(NHC)_2_] complexes are quite air- and moisture-sensitive.

**Scheme 15:**
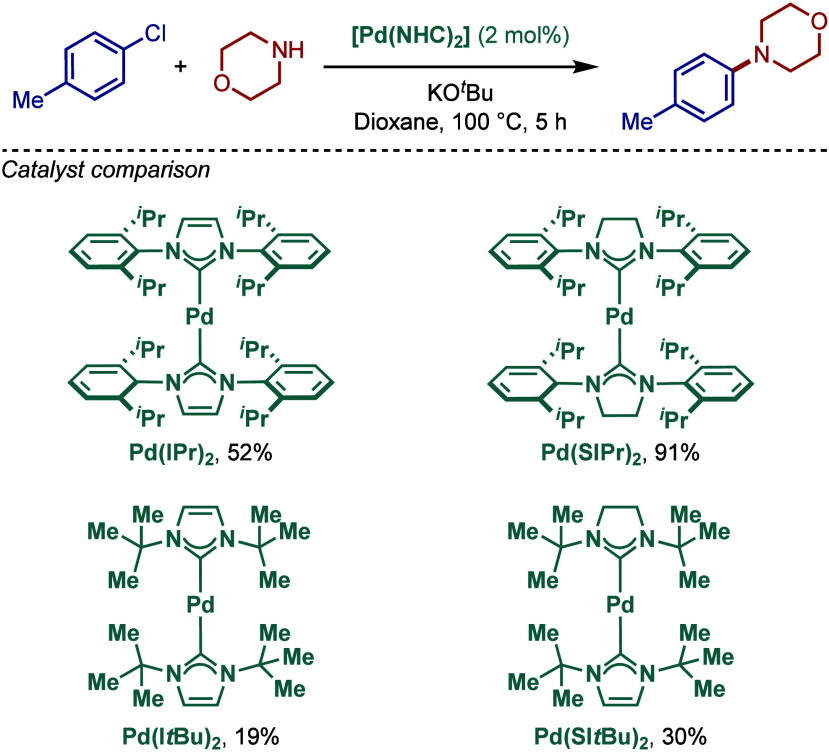
Well-Defined [Pd­(NHC)_2_]-Catalyzed BHA Reaction by Caddick

#### 2.2.2 [Pd­(NHC)­(PR_3_)] Complexes

The utility of well-defined [Pd­(NHC)­(PR_3_)] complexes in Buchwald–Hartwig cross-coupling was first evaluated by Caddick, Cloke, and co-workers (Scheme 16).^106^ These mixed phosphine/N-heterocyclic carbene complexes are known to provide complementary reactivity to their mono-N-heterocyclic carbene congeners in cross-coupling reactions.^36–43^ [Pd­(NHC)­(PR_3_)] complexes were synthesized by a ligand exchange reaction between [Pd­(NHC)_2_] and phosphines (PR_3_). An alternative exchange route between [Pd­(NHC)_2_] and [Pd­(PR_3_)_2_] complexes was also developed. This allowed for the synthesis of a series of monocarbene ligated palladium phosphine complexes, including [Pd­(I*t*Bu)­(P­(*o*-tolyl)_3_)], [Pd­(IPr)­(P­(*o*-tolyl)_3_)], [Pd­(I*t*Bu)­(PCy_3_)]. One of the complexes, [Pd­(IPr)­(P­(*o*-tolyl)_3_)], was employed for the Buchwald–Hartwig cross-coupling of 1°, 2° amines and imines in excellent yields using KO^*t*^Bu as a base in dioxane at 100 °C.

**Scheme 16:**
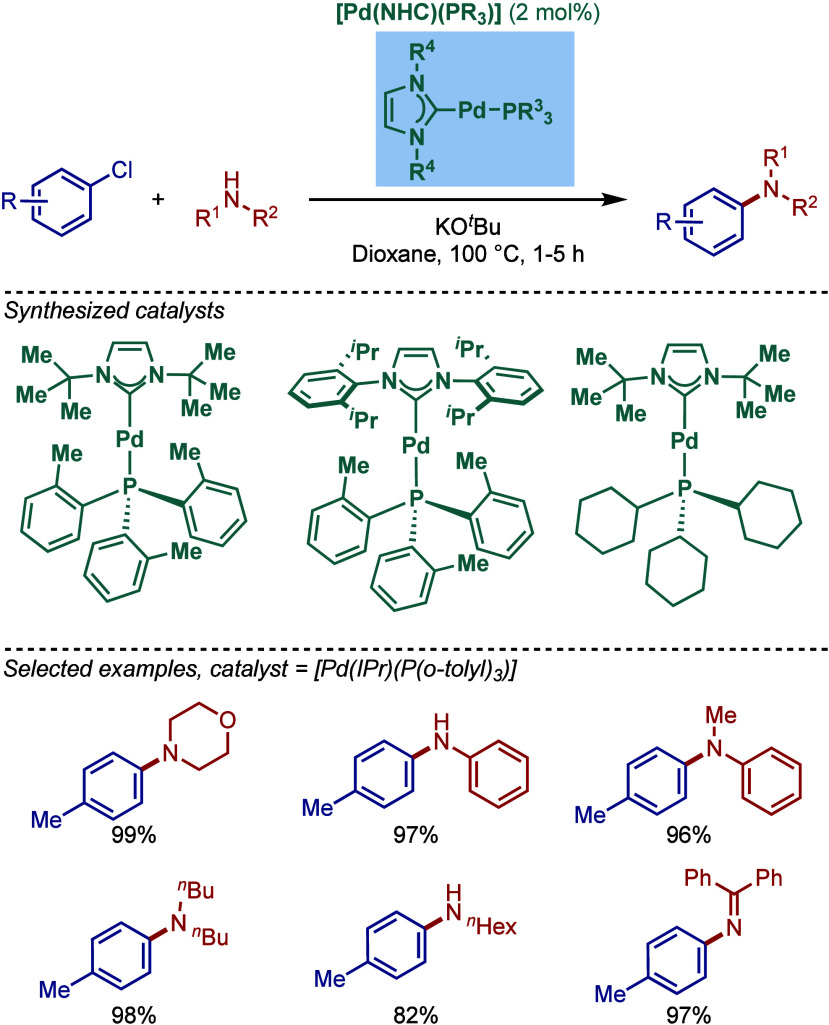
Well-Defined [Pd­(NHC)­(PR_3_)]-Catalyzed BHA Reaction by Caddick and Cloke

#### 2.2.3 [Pd­(NHC)­(BQ)] Complexes

Monocarbenepalladium­(0) complexes of N-heterocyclic carbenes with olefins were first reported by Beller and co-workers in 2002 for telomerization of 1,3-dienes and C–C bond cross-coupling (Suzuki and Heck).^110,111^ These authors found that coordination olefins, such as 1,1,3,3-tetra-methyl-1,3-divinyl-disiloxane (dvds), benzoquinone (BQ), and naphthoquinone (NQ), render the corresponding NHC–palladium(0) complexes remarkably stable, permitting their handling even under air (not shown).

Subsequently, the same authors took the advantage of these monocarbenepalladium(0) complexes, [Pd­(IMes)­(dvds)], [Pd­(IMes)­(BQ)]_2_, and [Pd­(IMes)­(NQ)]_2_, in the Buchwald–Hartwig cross-coupling of 4-chloro- and 4-bromoanisole with mesitylamine (Scheme 17).^112^ Among the three catalysts tested, [Pd­(IMes)­(NQ)]_2_ afforded the highest yield of the cross-coupling, although the reactivity was moderate (41% yield). Interestingly, the corresponding [Pd­(NHC)­(dvds) and [Pd­(NHC)­(BQ) complexes were much less productive; the presence of dvds or BQ on palladium significantly slowed the coupling reaction. In these cases, reductive dehalogenation of aryl halides became the dominant process.

**Scheme 17:**
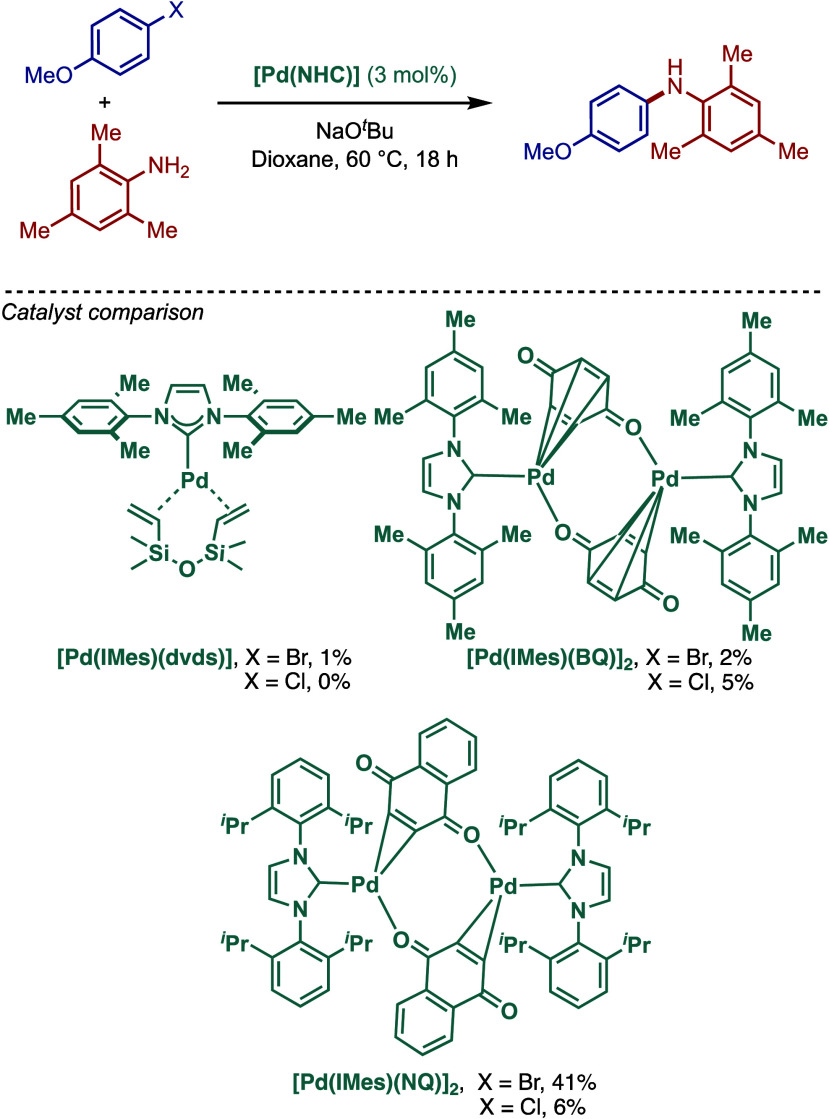
Well-Defined [Pd­(NHC)­(BQ)]-Catalyzed BHA Reaction by Beller

Later, in 2005, the important drawback of the previous protocol using [Pd­(NHC)­(NQ)]_2_ was addressed by Gooßen and co-workers (Scheme 18).^113^ These authors found that [Pd­(NHC)­(NQ)]_2_ complexes containing more sterically demanding IPr gave excellent yields in BHA reaction of aryl chlorides at a 0.5% catalyst loading using KOH in dioxane at 100 °C. In contrast, complexes bearing the less bulky IMes ligand were significantly less reactive. Various primary and secondary amines were reacted with aryl chlorides to afford the cross-coupling products in excellent yields. Interestingly, these well-defined Pd(0)–NHC complexes are significantly air- and moisture-stable and could be considered as an alternative to other classes of well-defined Pd–NHCs in the BHA reaction.

**Scheme 18:**
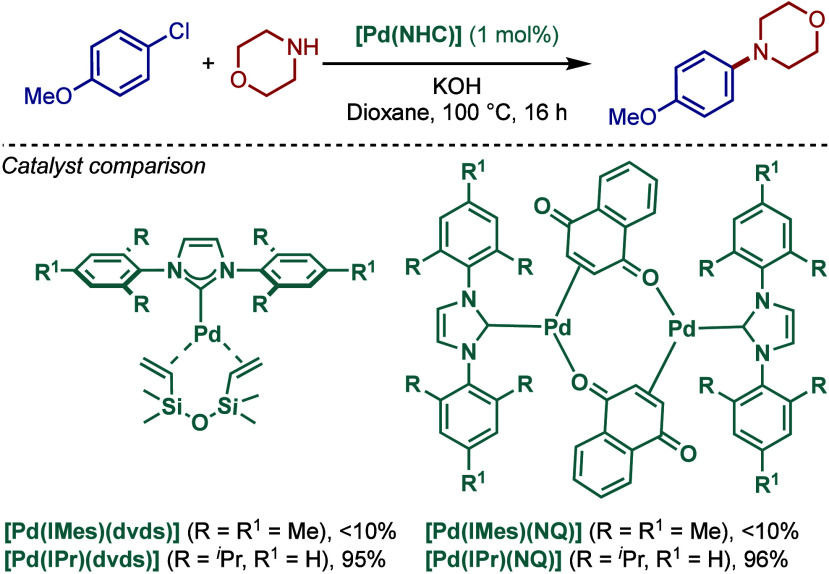
Well-Defined [Pd­(NHC)­Pd­(BQ)]-Catalyzed BHA Reaction by Gooßen

### 2.3 Well-Defined Pd­(II)–NHC Complexes

#### 2.3.1 [Pd­(NHC)­(η^3^-allyl)­Cl] Complexes

In 2002, Nolan and co-workers reported a series of air- and moisture-stable [Pd­(NHC)­(η^3^-allyl)­Cl] complexes for Buchwald–Hartwig cross-coupling (Scheme 19).^114^ Single-crystal X-ray analysis disclosed η^3^-coordination mode of the allyl fragment and a distorted-square-planar geometry around Pd. The synthesis of these well-defined Pd­(II)–NHCs is facile and involves a direct reaction of [Pd­(allyl)­Cl]_2_ with the corresponding free NHC. Complexes bearing different imidazol-2-ylidenes as well as imidazolin-2-ylidenes, such as [Pd­(IPr)­(η^3^-allyl)­Cl], [Pd­(IMes)­(η^3^-allyl)­Cl], [Pd­(I*t*Bu)­(η^3^-allyl)­Cl], and [Pd­(SIPr)­(η^3^-allyl)­Cl], could be readily accessed by this route.

**Scheme 19:**
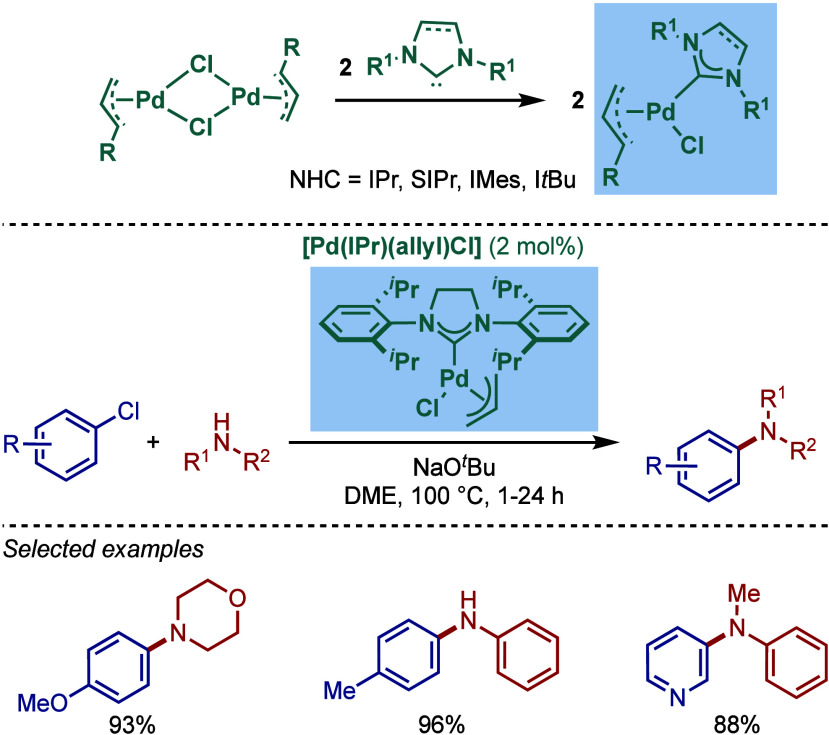
Synthesis of Well-Defined [Pd­(NHC)­(η^3^-allyl)­Cl] Complexes and the Use of [Pd­(SIPr)­(η^3^-allyl)­Cl] in BHA Reaction of Aryl Halides by Nolan

The authors proposed that a facile formation of Pd(0) from Pd­(II) is the key to the high reactivity in BHA reaction. Imidazolin-2-ylidene-based [Pd­(SIPr)­(η^3^-allyl)­Cl] was found the most reactive in the intermolecular amination (vide infra). Subsequently, an intramolecular variant was also developed, and this study featured a successful synthesis of a complex precursor for Cryptaustoline and Cryptowoline alkaloids (Scheme 20).^115^ A study comparing different [Pd­(NHC)­(η^3^-allyl)­Cl] catalysts revealed that for this intramolecular variant, the less sterically demanding [Pd­(IMes)­(η^3^-allyl)­Cl] was the most reactive catalyst.

**Scheme 20:**
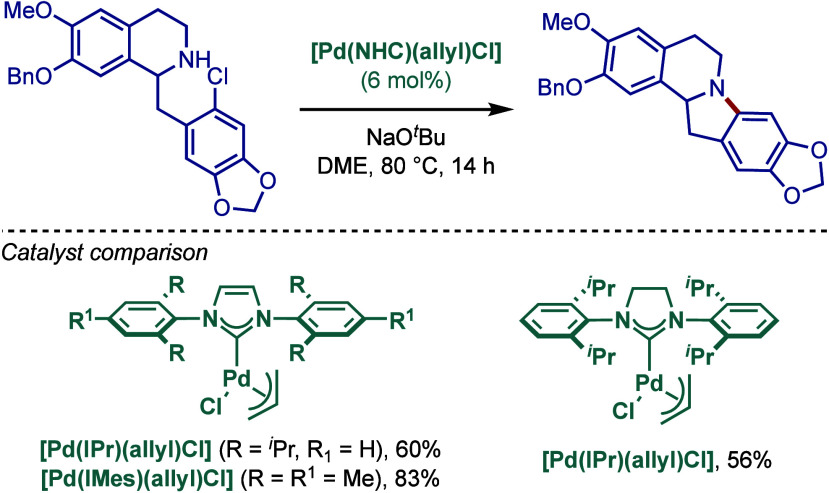
Intramolecular BHA Reaction Catalyzed by [Pd­(NHC)­(h^3^-allyl)­Cl] Complexes by Nolan

Furthermore, the same authors showed that not only aryl halides but also aryl triflates could be successfully used for the BHA reaction protocol using well-defined [Pd­(NHC)­(η^3^-allyl)­Cl] complexes (Scheme 21).^116^ Interestingly, in this case, imidazol-2-ylidene-based [Pd­(IPr)­(η^3^-allyl)­Cl] was the superior catalyst using toluene as solvent and NaO^*t*^Bu as base at 70 °C. These results highlight the importance of screening a set of electronically- and sterically differentiated NHC ligands to obtain the optimum reaction conditions for BHA reaction.

**Scheme 21:**
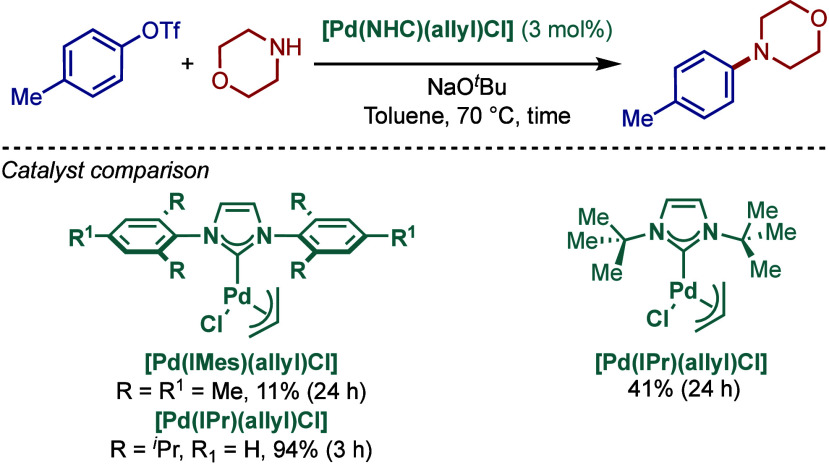
BHA Reaction of Aryl Triflates Catalyzed by [Pd­(NHC)­(η^3^-allyl)­Cl] Complexes by Nolan

In 2004, Nolan and co-workers reported a comprehensive study on the synthesis and structural characterization of well-defined [Pd­(NHC)­(η^3^-allyl)­Cl] complexes (Scheme 22).^117^ The complexes were investigated in BHA reaction under temperatures ranging from room temperature to 80 °C. Single crystal analysis revealed a distorted square-planar geometry around palladium, wherein chloride is positioned *cis* to carbene and the allyl moiety shows η^3^-coordination to palladium with one terminal carbon trans to carbene and the other terminal carbon trans to chloride. Interestingly, more strongly σ-donating alkyl-substituted NHC ligands featured longer Pd–C_carbene_ bonds in their [Pd­(NHC)­(η^3^-allyl)­Cl] complexes. The steric demand of each NHC ligand was evaluated using the percent buried volume (%V_*bur*_), and an attempt was made to correlate the steric demand with reactivity. The imidazolin-2-ylidene and N-alkyl-imidazol-2-ylidene complexes, such as [Pd­(SIPr)­(η^3^-allyl)­Cl], [Pd­(I*t*Bu)­(η^3^-allyl)­Cl], and [Pd­(IAd)­(η^3^-allyl)­Cl], featured the highest %V_*bur*_ (>32%) vs their congeners (%V_*bur*_ of 25% or less). These [Pd­(NHC)­(η^3^-allyl)­Cl] complexes showed excellent reactivity in Buchwald–Hartwig coupling (Scheme 22). The only exceptions were [Pd­(ICy)­(η^3^-allyl)­Cl] and [Pd­(IBn^aMe^)­(η^3^-allyl)­Cl], two complexes that featured the lowest %V_*bur*_ values (<24%).

**Scheme 22:**
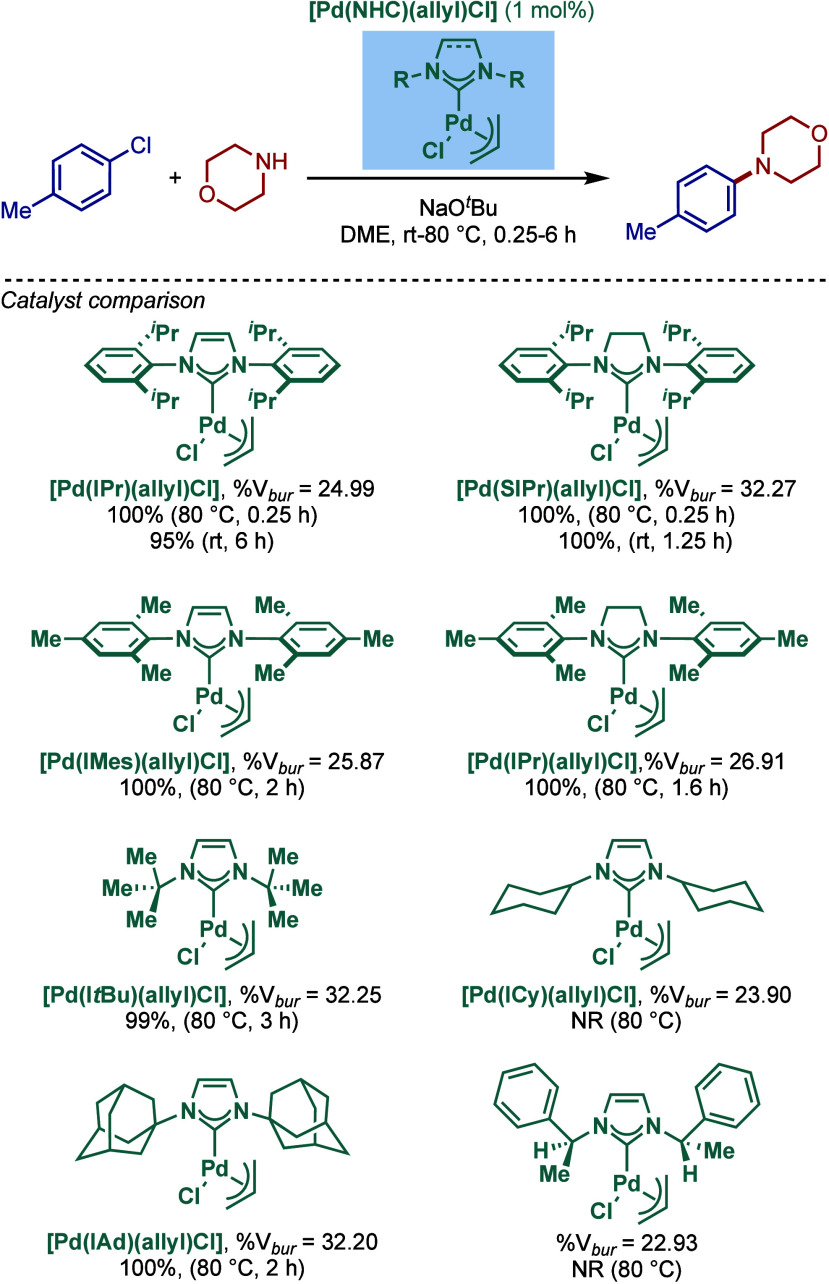
Steric Effect of Well-Defined [(NHC)­Pd­(η^3^-allyl)­Cl] Complexes in BHA Reaction by Nolan

The reaction mechanism has been proposed to involve an associative oxidative addition to [Pd–NHC] species, which contrasts with a dissociative pathway of bis-phosphine palladium systems.^118^ The activation mode is initiated with a nucleophilic attack at the allyl moiety or through a chloride replacement with alkoxide, which is followed by reductive elimination (Scheme 23).^119^ These pathways deliver [(NHC)–Pd(0)], which is an active species that initiates oxidative addition. Furthermore, to elucidate the effect of allyl substitution, the terminal position of the allyl ligands was also varied, including allyl, crotyl, prenyl, and cinnamyl groups (Scheme 23). Interestingly, the authors found that the catalytic efficiency of these [Pd­(II)–NHC] complexes in BHA reaction also significantly depended on the type of allyl group. Thus, substitution at the terminal position of the allyl group, enhances the allyl’s dissymmetry, which in turn facilitates the activation step (elimination of the allyl moiety) from Pd­(II) to Pd(0).

**Scheme 23:**
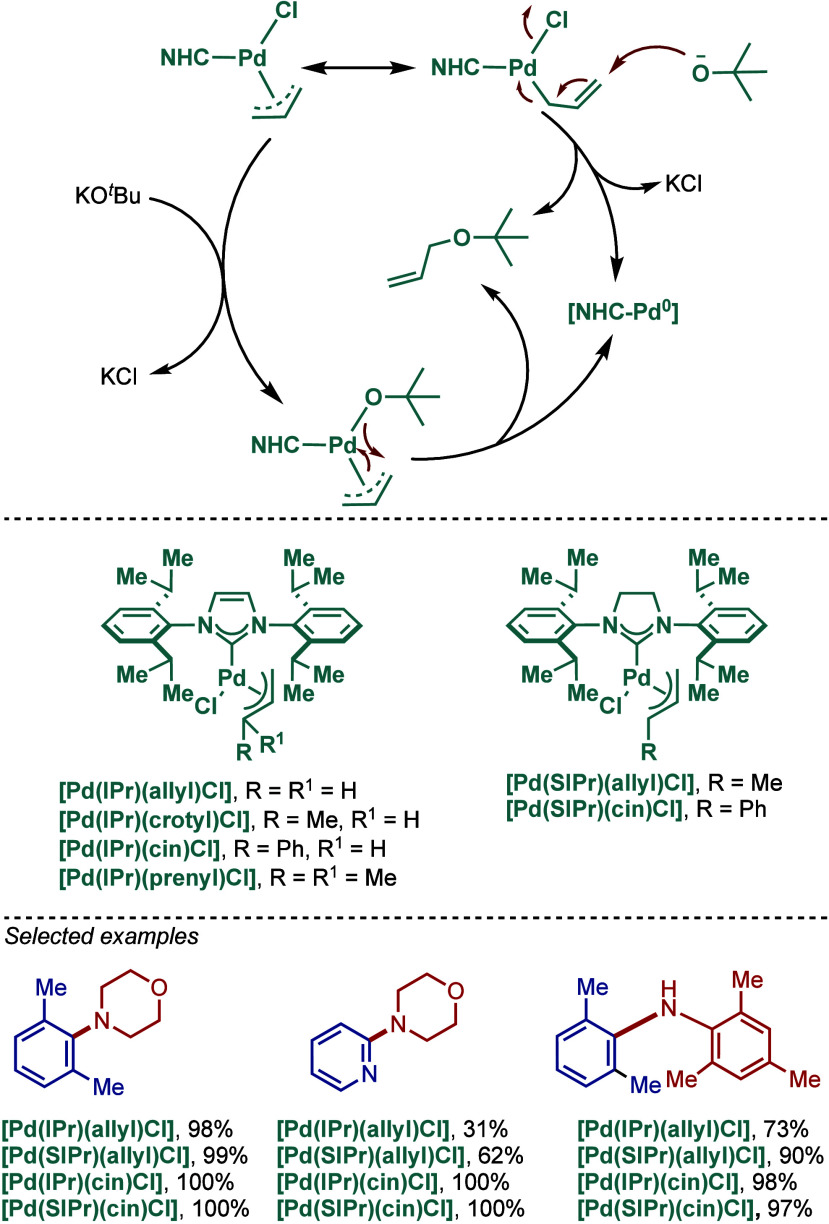
Influence of Allyl-Substitution on Well-Defined [Pd­(NHC)­(allyl)­Cl] Complexes in BHA Reaction by Nolan

Impressively, this ease of activation translated into an extraordinarily high catalytic activity even at room temperature. As a result, these allyl complexes showed high efficiency in BHA reaction of a variety of 1°, 2°, alkyl and arylamines with unactivated, neutral and activated aryl chlorides and bromides. [Pd­(SIPr)­(cin)­Cl] was found to be the most active catalyst and promoted the amination at as low as 10 ppm catalyst loading at 80 °C. These catalysts were also highly effective for BHA reactions of sterically hindered substrates, producing tri*ortho*- and tetra-*ortho*-substituted diarylamines at room temperature. Furthermore, this [Pd­(SIPr)­(cin)­Cl] complex was also equally effective for the challenging Buchwald–Hartwig cross-coupling of heteroaromatic halides at room temperature.^120^

Later, in 2008, Caddick and co-workers reported a related second-generation [Pd­(NHC)­(allyl)­Cl] catalyst, [Pd­(SIPr)­(methallyl)­Cl], for BHA reaction of aryl chlorides and bromides using LiHMDS as a base (not shown).^121^ Furthermore, Nolan and co-workers reported that these [Pd­(NHC)­(η^3^-cin)­Cl] complexes (NHC = IPr, SIPr) can be used to deliver the corresponding palladium hydroxide dimers [Pd­(NHC)­(η^1^-cin)­(μ-OH)]_2_ in the presence of cesium hydroxide, which were also effective in BHA reaction (not shown).^122^ Several other studies have also been reported, including solvent-resistant nanofiltration of [Pd­(NHC)­(allyl)­Cl] complexes and their application in BHA reaction by Plenio,^123^ where the use of polydimethylsiloxane membrane on polyacrylonitrile offered a high retention (97–99.9%) of [(NHC)–Pd], while the amination product contained residual Pd in 3.5–25 ppm range as well as the synthesis of polytriarylamines by Buchwald–Hartwig cross-coupling of aryl chlorides using [Pd­(SIPr)­(cin)­Cl] by Navarro (not shown)^124–126^ and polyisobutylene-supported [Pd­(NHC)­(allyl)­Cl] complexes for BHA reaction by Bergbreiter (not shown).^127^

In 2009, Dorta and co-workers introduced a new class of sterically demanding, 1-naphthyl-based [Pd­(NHC)­(cin)­Cl] complexes that feature 2-mono or 2,6-disubstitution for BHA reaction of aryl chlorides (Scheme 24).^128–130^ In this design, 1-naphthyl moiety generates C_2_-symmetric (anti) and C_*s*_-symmetric (syn) atropisomers. Rotational energy barriers between two isomers in both N-heterocyclic carbene salts and free carbenes were calculated using variable temperature-dependent ^1^H NMR spectroscopy. The corresponding palladium complexes showed similar rotational barriers, (Pd­[(2-^*i*^PrSINp)­(cin)­Cl], ΔG^‡^ = 80.6 kJmol^–1^; [Pd­(2,6-^*i*^PrSINp)­(cin)­Cl], ΔG^‡^ = 80.4 kJmol^–1^; [Pd­(2-CySINp)­(cin)­Cl], ΔG^‡^ = 80.6 kJmol^–1^), however, the barrier rapidly decreased for the corresponding Me-complex ([Pd­(2-MeSINp)­(cin)­Cl], ΔG^‡^ = 56.9 kJmol^–1^). These sterically demanding complexes were evaluated in BHA reaction, and the most sterically demanding complex, [Pd­(2-CySINp)­(cin)­Cl], was found to be the most active. However, these catalysts proved less reactive than their imidazolin-2-ylidene congener, [Pd­(SIPr)­(cin)­Cl].

**Scheme 24:**
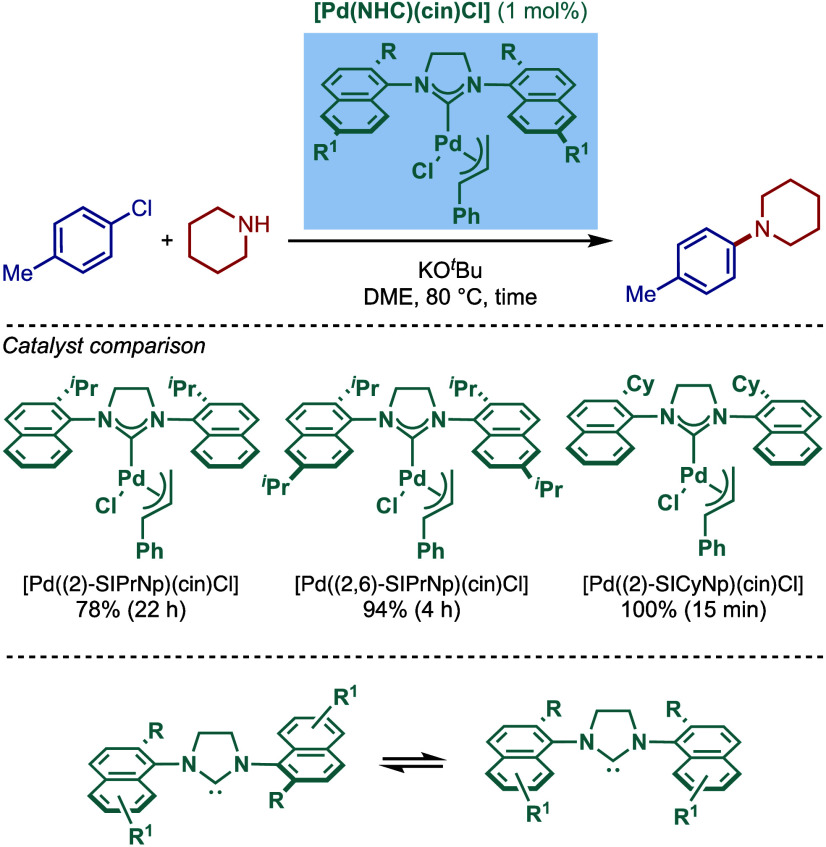
Atropisomeric, 1-Naphthyl [(SINp)­Pd­(cin)­Cl] Complexes in BHA Reaction by Dorta

In 2010, Cowley, Green, and co-workers reported another class of [Pd­(NHC)­(allyl)­Cl] complexes where the imidazole backbone was modified by saturated BIAN substitution (BIAN–H, 6b, 9a-dihydroacenaphtho­[1,2-*d*]­imidazolinium (Scheme 25).^131^ Two different sterically hindered catalysts, namely [Pd­(BIAN–SIMes)­(allyl)­Cl] and [Pd­(BIAN–SIPr)­(allyl)­Cl], were synthesized and evaluated in the Buchwald–Hartwig cross-coupling. Structural characterization revealed that [Pd­(BIAN–SIMes)­(allyl)­Cl] showed only one diastereomer at room temperature as probed in ^1^H NMR studies. The allyl carbon atoms attached to the palladium center were not symmetric. The authors proposed that strong σ-donation and greater trans effect of the carbene ligand (cf. chloride) resulted in weak bonding of the allyl carbon atom trans to carbene, which results in the asymmetry of the allylic bonding and facilitates complex activation. Interestingly, a dynamic fluxional behavior was observed for the more sterically demanding [Pd­(BIAN–SIPr)­(allyl)­Cl] at room temperature, while the complex conformationally froze at 233 K.

**Scheme 25:**
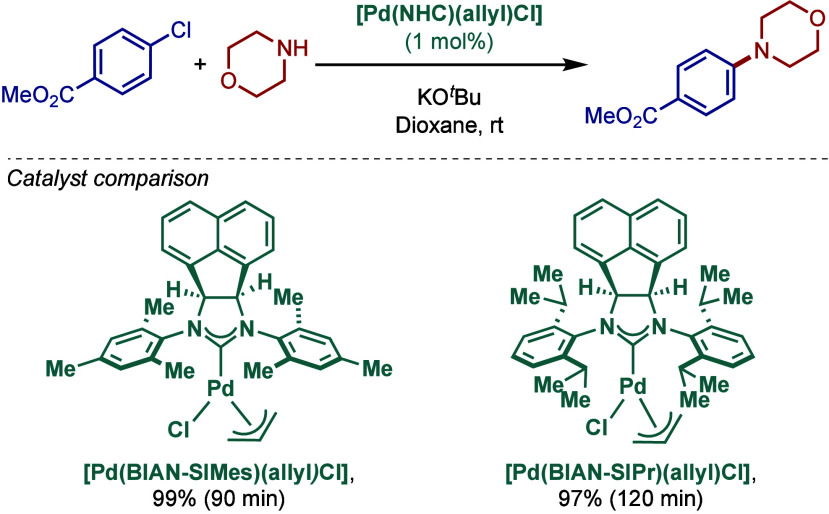
Well-Defined BIAN–NHC Complexes [Pd­(BIAN–NHC)­(allyl)­Cl] for BHA Reaction by Cowley and Green

Subsequently, Tu and co-workers reported a related BIAN–NHC complex, [Pd­(BIAN–IPr)­(allyl)­Cl], for aminocarbonylation of iodoarenes (Scheme 26).^132^ This complex was found to promote the coupling of a wide variety of aryl and heteroaryl substrates under atmospheric pressure of carbon monoxide in excellent yields. The catalyst was showcased in a gram scale synthesis of an anticancer drug, tamibarotene, using this protocol.

**Scheme 26:**
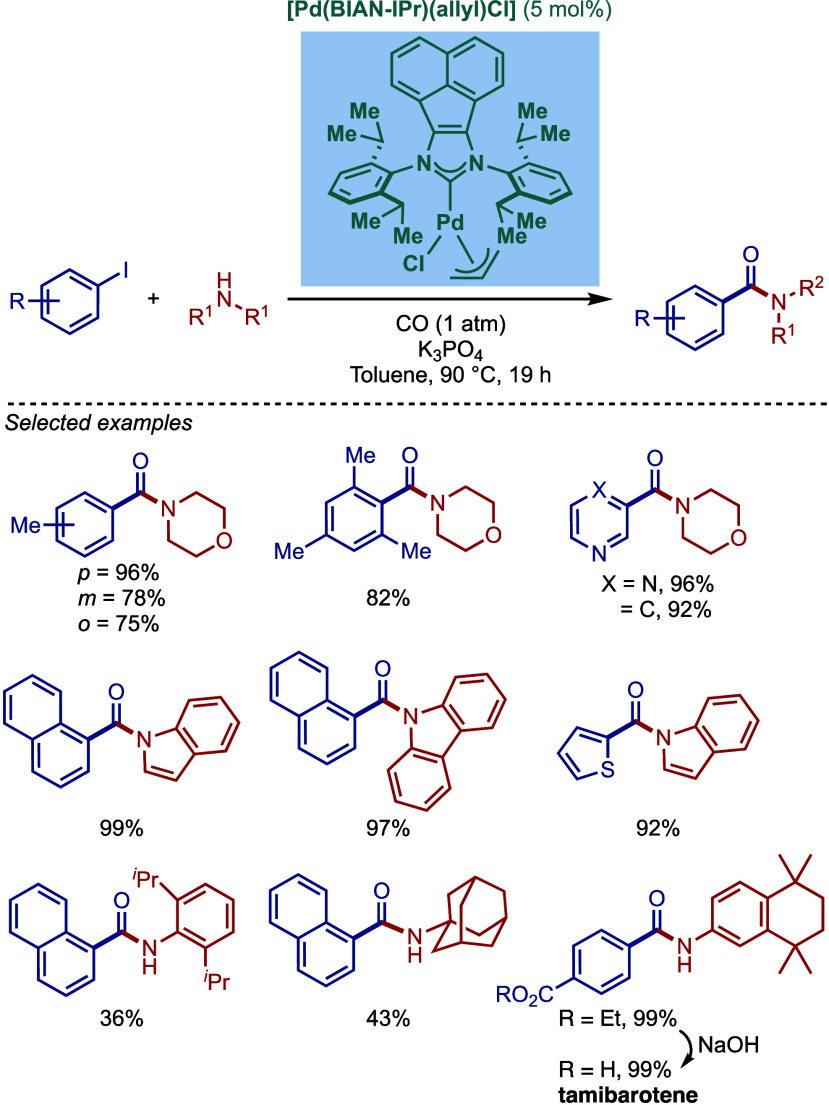
Well-Defined BIAN–NHC Complex [Pd­(BIAN–IPr)­(allyl)­Cl] for Aminocarbonylation of Aryl Iodides by Tu

In 2010, Marko and co-workers reported a new sterically bulky N-heterocyclic carbene, IPr*, bearing 2,6-diphenylmethyl substitution at the *ortho*-position of the N-aromatic ring.^133^ This ligand design is highly modular, enabling significant conformational flexibility around the catalytic center.^134^

In 2012, Nolan and co-workers showed the practical advantages of this ligand class in BHA reaction (Scheme 27).^135,136^ They found that [Pd­(IPr*)­(cin)­Cl] and [Pd­(IPr*^OMe^)­(cin)­Cl] complexes are some of the most reactive N-heterocyclic carbene-based catalysts for the amination of aryl chlorides. The reaction proceeded at room temperature with several sterically hindered aryl chlorides and amines at 1 mol % catalyst loading, while the loading could be further decreased to 0.05 mol % at 110 °C using toluene and KO^*t*^Am as a base. Subsequently, the Nolan group reported a solvent-free approach for BHA reaction using the same class of catalysts.^137^ A continuous flow microreactor approach was also developed for BHA reaction.^138,139^ To address the clogging in both microreactors and continuous flow reactive systems, a novel four-feed flow system was developed. In these cases, efficient heat transfer of microreactor permits faster conversion at slightly increased temperatures.

**Scheme 27:**
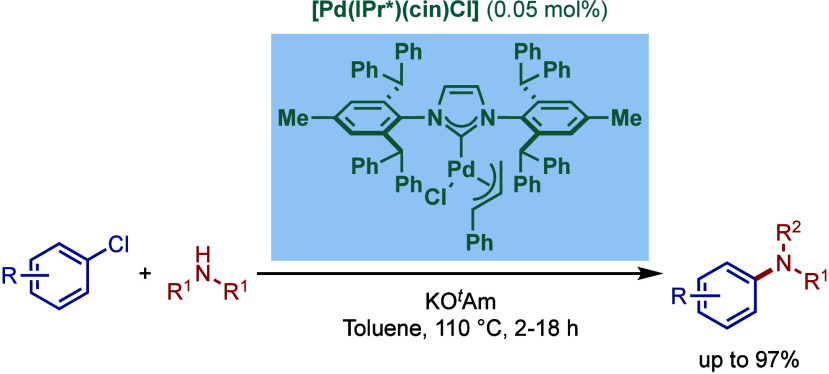
Sterically-Hindered [Pd­(IPr*)­(cin)­Cl] in BHA Reaction of Aryl Halides by Nolan

In 2016, Meadows and co-workers at AstraZeneca reported [Pd­(IPr*)­(cin)­Cl] as an excellent catalyst for continuous flow BHA reaction (Scheme 28).^140–142^ This catalyst was synthesized on a multihundred-gram scale in batches up to 168 g for the imidazolium salt. The continuous BHA reaction was used for the synthesis of a key pharmaceutical intermediate for the treatment of central nervous disorders. A continuous method was developed for continuous workup and purification, catalyst recycling, and reuse. The flow workup methodology featured the selective extraction of the Buchwald–Hartwig product into the aqueous stream as a salt, while the aryl bromide starting material and the catalyst were extracted in the organic stream, simplifying further purification process.

**Scheme 28:**
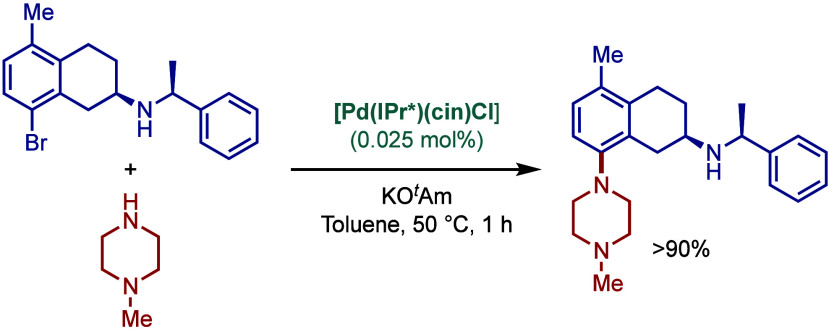
Continuous Flow BHA Reaction of Pharmaceuticals Catalyzed by [Pd­(IPr*)­(cin)­Cl] by Meadows

In 2015, Nolan and co-workers reported the ITent (Tent = tentacular) series of [Pd­(NHC)­(allyl)­Cl] catalysts (Scheme 29).^143^ These catalysts feature bulky-yet-flexible N-heterocyclic carbene ligands, such as IPent, IHept, and INon, where steric flexibility of the N-aromatic wingtips facilitates reductive elimination. Interestingly, these catalysts were found to be particularly effective in Buchwald–Hartwig cross-coupling in apolar hydrocarbon solvents. The use of alkane solvents in BHA reactions is rare due to poor solvation in apolar solvents. The authors hypothesized that long alkyl chains of the catalyst N-aryl wingtip facilitated solvation in apolar solvents. The most active catalyst was the one bearing the longest alkyl chains [Pd­(INon)­(allyl)­Cl], which promoted the BHA reaction in heptane at 80 °C in the presence of KO^*t*^Bu as a base.

**Scheme 29:**
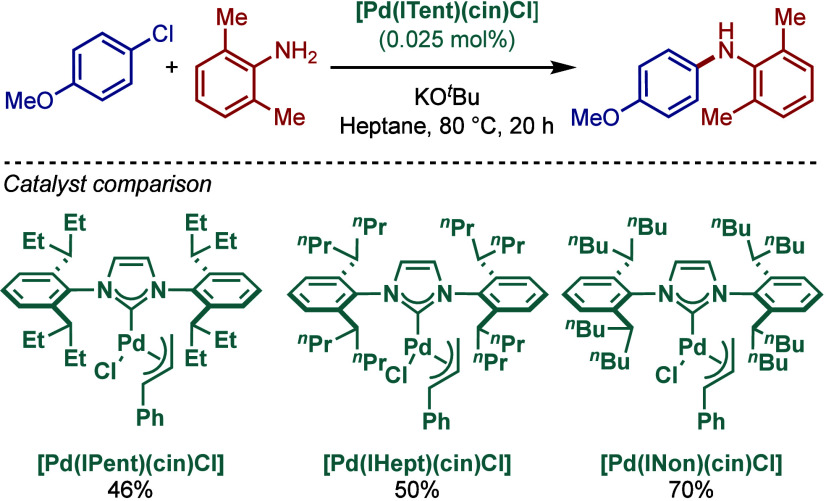
Bulky-Yet-Flexible [Pd­(ITent)­(allyl)­Cl] Catalysts in BHA Reaction in Apolar Solvents by Nolan

An alternative approach to BHA reactions in apolar solvents was reported by Glorius and co-workers using backbone-modified NHC ligand with long alkyl chains (Scheme 30).^144^ Three different imidazol-2-ylidene catalysts based on the [Pd­(NHC)­(allyl)­Cl] system were synthesized [Pd­(IMes^C11H23^)­(allyl)­Cl], [Pd­(IPr^C11H23^)­(allyl)­Cl], [Pd­(IPr^C7H15^)­(allyl)­Cl]. Evaluation of their reactivity in BHA reaction in heptane in the presence of KO*t*Bu at 75 °C revealed that [Pd­(IPr^C11H23^)­(allyl)­Cl] was the best catalyst, although [Pd­(IPr^C7H15^)­(allyl)­Cl] gave comparable reactivity. These two approaches offer benefits of using hydrocarbon solvents for BHA reactions for industrial applications.

**Scheme 30:**
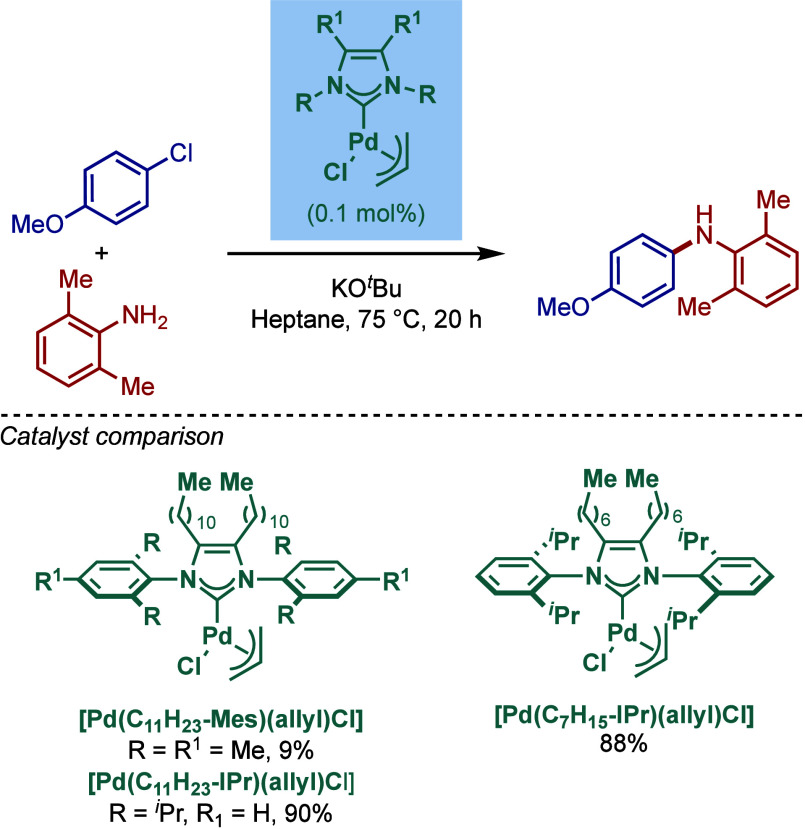
Non-Polar [Pd­(NHC^Alkyl^)­(allyl)­Cl] Complexes in BHA Reaction in Apolar Solvents by Glorius

Amides represent a high attractive yet very challenging class of substrates for the BHA reaction because of the low nucleophilicity of the amide nitrogen (p*K*_a_ of ∼ 23) compared to alkylamines (p*K*_a_ of ∼ 43) and arylamines (p*K*_a_ of ∼ 30; all p*K*_a_ values in DMSO). In 2017, Organ and co-workers introduced a Lewis acid strategy as an excellent promoter for the Pd–NHC-catalyzed amide BHA reaction (Scheme 31).^145^ They showed that sterically bulky [Pd­(^DiMe^IHept^Cl^)­(cin)­Cl] was an excellent catalyst for this reaction outperforming its Pd–PEPPSI (PEPPSI = 3-Cl-py) congeners, such as [Pd­(IPent^Cl^)­(3-Cl-py)­Cl_2_] and [Pd­(IHept^Cl^)­(3-Cl-py)­Cl_2_]. The key to the success of this coupling is the use of a Lewis acid, such as B­(*sec*Bu)_3_, BEt_3_, or B­(C_6_F_5_)_3_. The ^11^B and ^13^C NMR data suggested that the Lewis acid coordinates to the amide oxygen atom to form a boron–amidonium complex, which is deprotonated in the presence of cesium carbonate forming cesium boron amidate salt. This in turn increases the amide bond nucleophilicity, enabling for highly efficient coupling. It is worth noting that these B­(*sec*Bu)_3_-promoted conditions demonstrate high functional group tolerance, even with base-sensitive functional groups.

**Scheme 31:**
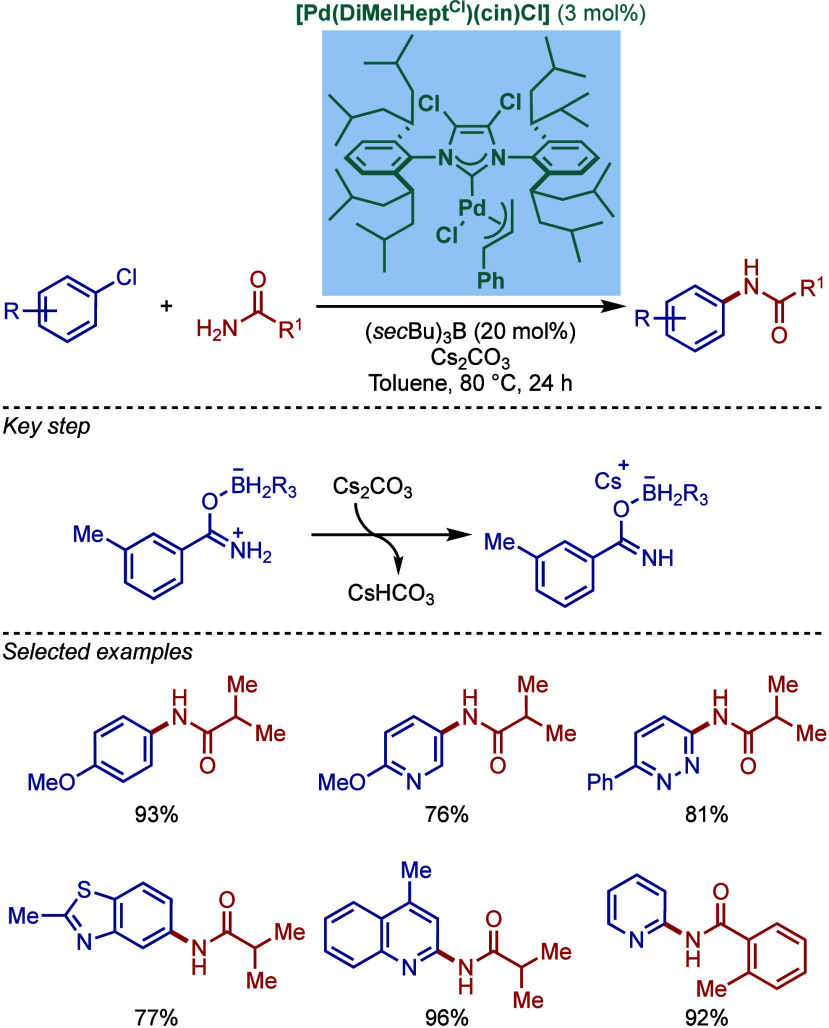
Buchwald–Hartwig Cross-Coupling of Amides Catalyzed by [Pd­(^DiMe^IHept^Cl^)­(cin)­Cl] and Lewis Acids by Organ

Very recently, Organ and co-workers reported a mechanistic investigation of Pd–NHC-catalyzed C–N bond-forming reaction where they supported the presence of a zerovalent Pd­(NHC) species using sterically hindered DiMeIHept^Cl^ as a carbene ligand. The reactive Pd(0)–NHC species was trapped by molecular nitrogen, benzene, and pyridine, and the structures unambiguously confirmed by X-ray crystallography. Interestingly, the 14-electron Pd-species, [Pd­(DiMeIHeptCl)­(Ph)­Cl] was isolated after the oxidative addition step. This complex was found to be stable under air, which can be a result of dispersion interactions between the alkyl chains of the NHC wingtip and the Pd–Ph group.^146^

Ring-expanded N-heterocyclic carbenes,^61^ which benefit from higher σ-donicity than the classical imidazolium and imidazolinium-based carbenes, were reported by Nechaev and co-workers in 2016 for the solvent-free BHA reaction of anilines, diarylamines, and dialkylamines mediated by [Pd­(RE–NHC)­(allyl)­Cl] complexes (Scheme 32).^147^ Catalysts based on six- and seven-membered ring-expanded carbenes were comprehensively tested, and the authors found that [Pd­(THP–Dipp)­(cin)­Cl] (>99%) was better catalyst than the other three counterparts, [Pd­(THP–Mes)­(cin)­Cl] (95%), [(THD–Dipp)­Pd­(cin)­Cl] (93%), [Pd­(THD–Mes)­(cin)­Cl] (trace) in this amination reaction (THP = 3,4,5-tetrahydropyrimidin-2-ylidene; THD = 3,4,5,6-tetrahydrodiazepin-2-ylidene) (Table 2). The utility of this method was further highlighted in the synthesis of commercially available organic light-emitting diodes (OLEDs) containing triarylamines in a single step. A comprehensive comparison with different classes of NHC and phosphine ligands showed excellent reactivity of these RE–NHC-based Pd­(II)–NHC catalysts in BHA reaction.

**Scheme 32:**
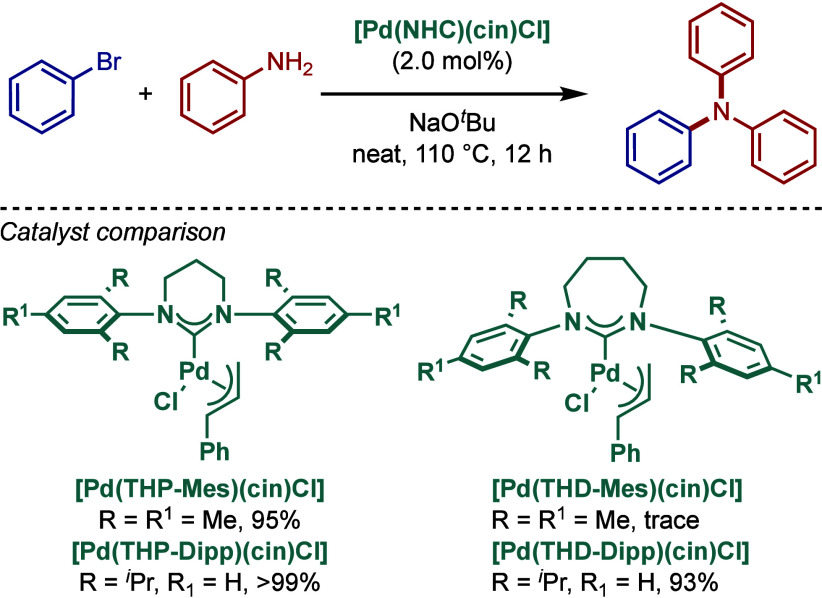
BHA Reaction Catalyzed by Ring–Expanded [Pd­(RE–NHC)­(cin)­Cl] Complexes by Nechaev

**Table 2:** Comparison between Different NHC–Pd Complexes in BHA reaction by Nechaev

entry	NHC	yield (%)
1	[Pd(PPh_3_)_4_]	-
2	[Pd(PPh_3_)_2_Cl_2_]	-
3	[Pd(P(*o*-Tol)_3_)_2_Cl_2_]	-
4	Pd(dba)_2_+SPhos	-
5	Pd(OAc)_2_+SPhos	-^a^
6	Pd(OAc)_2_+RuPhos	-^a^
7	[Pd(SIPr)(cin)Cl]	-^a^
8	[Pd(IPr)(cin)Cl]	-
9	[Pd(IPr)(PEPPSI)]	-
10	[Pd(THP-Mes)(cin)Cl]	95
11	[Pd(THP-Dipp)(cin)Cl]	>99
12	[Pd(THD-Mes)(cin)Cl]	93
13	[Pd(THD-Dipp)(cin)Cl]	-^a^
14	Pd(OAc)_2_ + IPr·HCl	-

a Traces of diphenylamine observed.

In 2017, César and co-workers reported an important study on the synthesis and application of [Pd­(NHC)­(cin)­Cl] complexes in the BHA reaction, where the NHC backbone has been modified by an amino group (NR_2_, R = Me, ^*i*^Pr) (Scheme 33).^148^ In this design, a tridimensional geometry of amines can accommodate flexible conformations to improve the catalytic efficiency. The steric constraint of the backbone substituent is translated on the N-aryl wingtips, which forces them to be twisted and interact closely with the metal coordination sphere during the catalytic cycle. This buttressing effect can be compared with the well-known chloride substitution of the NHC backbone; however, apart from the sterics, the NR_2_ group also exerts a strong σ-donating electronic effect on the carbene center. The same authors earlier reported related [Pd­(NHC)­(3-Cl-py)­Cl_2_] complexes (Scheme 72 and Scheme 73, see Section 2.3.8). These [Pd­(IPr^NR2^)­(allyl)­Cl] complexes have been used to promote highly challenging BHA reactions of sterically hindered trisubstituted primary amines with aryl chlorides under mild conditions. A comparative study revealed that the allyl-based Pd­(II)–NHC complexes, [Pd­(IPr^NR2^)­(allyl)­Cl], were more effective than their pyridine-supported counterparts, [Pd­(IPr^NR2^)­(3-Cl-py)­Cl]. The diamine substituted [Pd­(IPr^(NMe2)2^)­(cin)­Cl] complex was found to be the most active catalyst, which was rationalized by the best match of the steric and electronic effect of the backbone substitution.

**Scheme 33:**
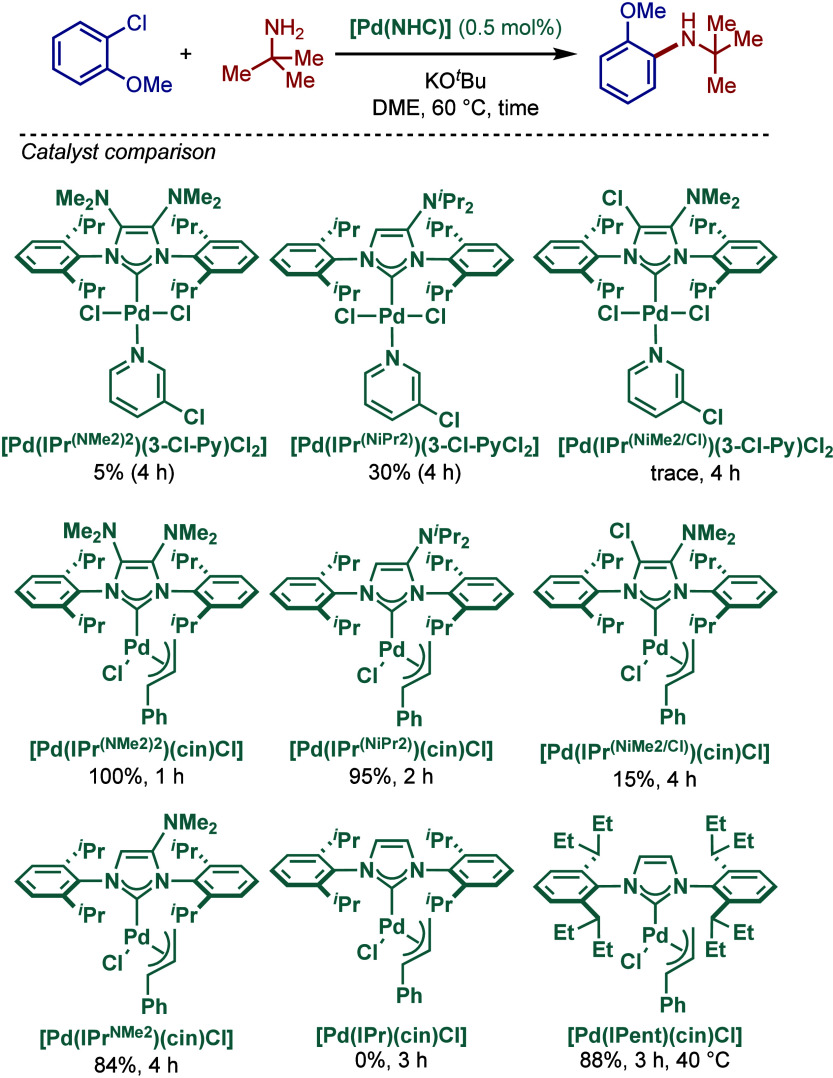
BHA Reaction Catalyzed by Amino–Backbone Modified [(IPr^NR2^)­Pd­(cin)­Cl] Complexes by César

In 2019, Choi and co-workers reported on the effect of silane-substitution of the NHC backbone in [Pd­(NHC)­(allyl)­Cl] complexes in the BHA reaction (Scheme 34).^149^ A series of sterically- and electronically differentiated [Pd­(IPr^SiR3^)­(allyl)­Cl] complexes were synthesized, where the backbone featured electropositive and bulky R_3_Si substituents at the 4-position. Interestingly, the most sterically hindered catalysts, such as [Pd­(IPr^IPr‑Si(SiMe3)Me2^)­(allyl)­Cl] and [Pd­(IPr^Si(*t*BuMe2)^)­(allyl)­Cl], were not effective. This reaction significantly depended on electronic factors, where two complexes featuring more electron-rich character, [Pd­(IPr^η1‑allylMe2^)­(η^3^-allyl)­Cl] (TEP = 2037.8 cm^–1^) and [Pd­(IPr^SiMe3^)­(η^3^-allyl)­Cl] (TEP = 2040.1 cm^–1^), outperformed other catalysts.

**Scheme 34:**
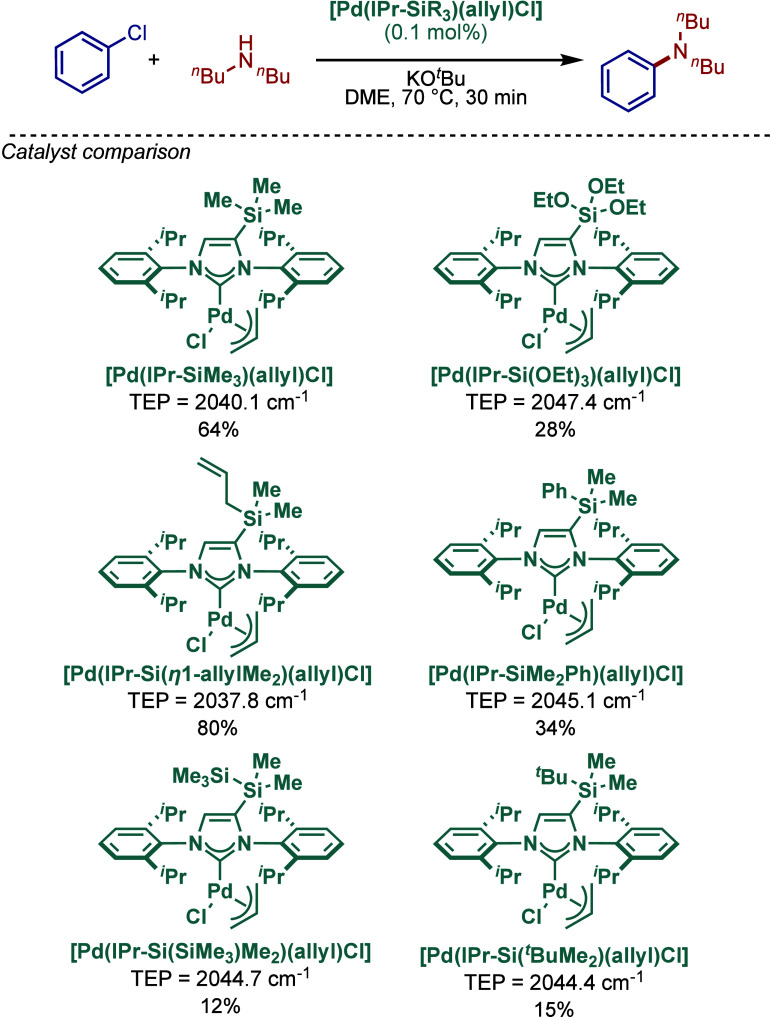
BHA Reaction Catalyzed by Silane–Backbone Modified [Pd­(IPr^SiR3^)­(η^3^-allyl)­Cl] Complexes by Choi

Subsequently, the same authors reported an immobilized IPr^SiR3^ N-heterocyclic carbene via direct silyl linker installation (not shown). This complex quantitatively facilitated BHA reaction of aryl chlorides within 10 min, even at a low Pd loading of 0.2 mol %. This catalyst could not be reused due to the formation of Pd nanoparticles.^150^

In another approach, the Tamm group reported a series of anionic N-heterocyclic carbenes in BHA reaction, featuring a weakly coordinating anion borate moiety at the NHC backbone (WCA–NHCs) (Scheme 35).^151^ The effect of varying the allyl ancillary ligand on palladium was investigated with allyl, crotyl, methallyl, cinnamyl, and [(WCA–IPr)­Pd­(allyl)­Cl] was found to be a superior catalyst for the amination reaction. A THF-coordinated [Li­(THF)_3_]­[(WCA–IPr)­Pd­(allyl)­Cl] was also reported and examined for BHA reaction. Spectroscopic studies revealed that the allyl ligand of these WCA–NHC complexes showed higher fluxional character than in the analogous [Pd­(NHC)­(allyl)­Cl] complexes, again hinting that the ease of allyl removal is a key factor in catalyst activation. Interestingly, a rare intramolecular Pd–arene coordination was observed in these WCA–NHC ligands, which could be compared with the Pd···C_ipso_ interaction in sterically hindered diaryl phosphines.^152^

**Scheme 35:**
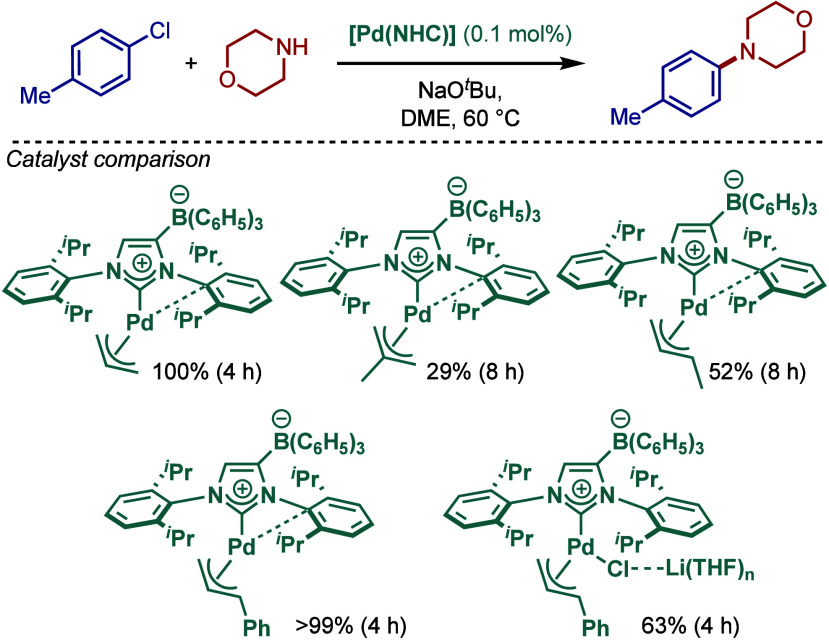
BHA Reaction Catalyzed by Weakly Coordinating Anion [Pd­(WCA–NHC)­(cin)­Cl] Complexes by Tamm

In 2019, Nolan, Cazin, and co-workers reported a quantitative synthesis of [NHC·H]­[Pd­(η^3^-R-allyl)­Cl_2_] complexes using a solvent-free method by grinding the corresponding NHC salt with [Pd­(η^3^-R-allyl)­(μ-Cl)]_2_ dimers (Scheme 36).^153^ These complexes were tested in BHA reaction of aryl chlorides. Among the catalysts tested, [IPr*·H]­[Pd­(cin)­Cl_2_] was found to be the most reactive. The substrate scope of this coupling is broad, including sterically- and electronically diverse aryl chlorides as well as 1° and 2° amines in cyclopentyl methyl ether as a green solvent at 60 °C.

**Scheme 36:**
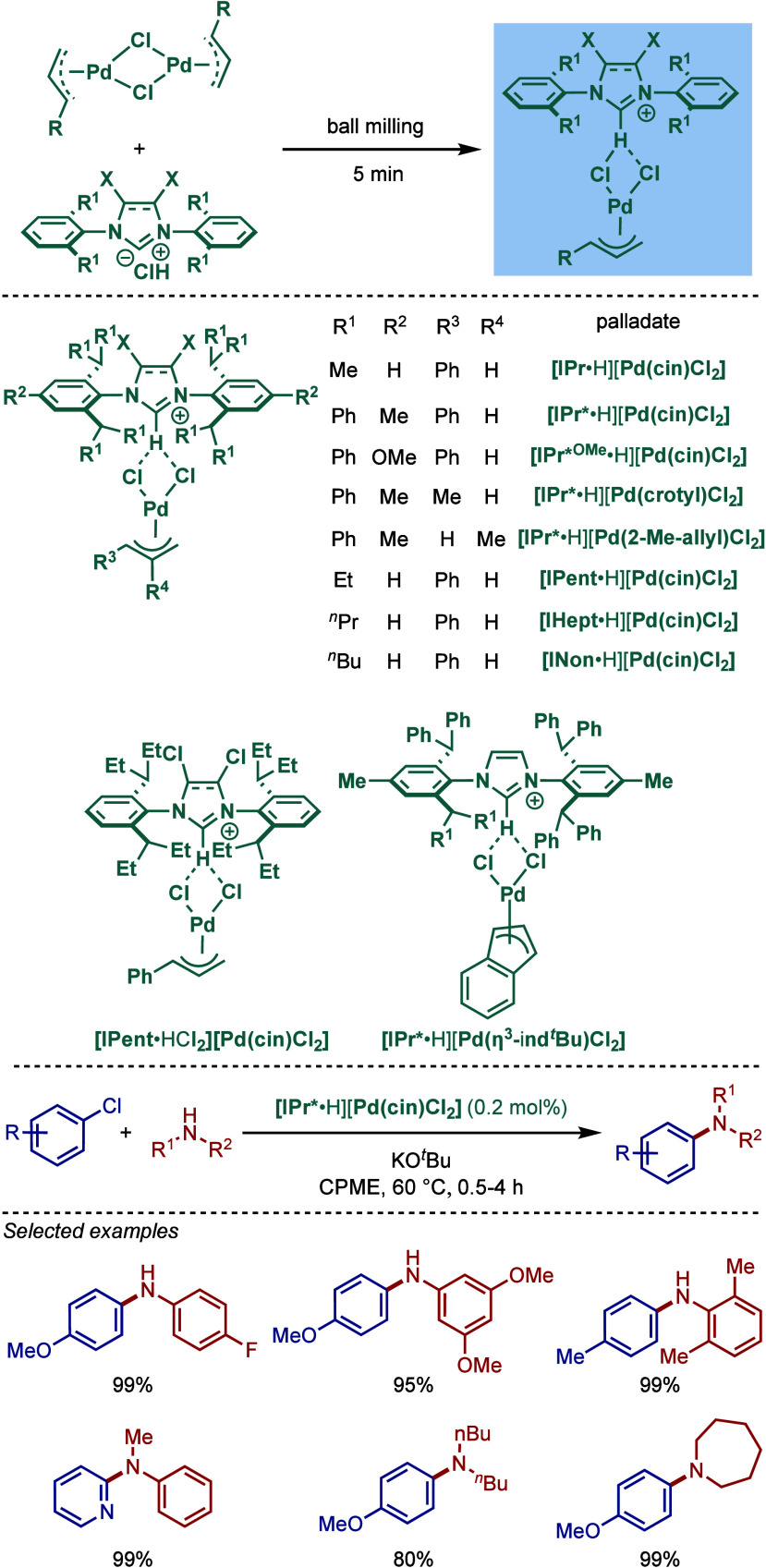
BHA Reaction using Palladate [NHC·H]­[Pd­(allyl)­Cl_2_] Precatalysts by Nolan and Cazin

The effect of the backbone substitution by the phenyl groups on the saturated imidazolinyl-2-ylidene NHC backbone in BHA reaction using [Pd­(NHC)­(allyl)­Cl] complexes was reported by Qui and co-workers (Scheme 37).^154^ Impressively, these authors determined that a bulky and electron-rich [Pd­(SIPr^Ph2^)­(cin)­Cl] catalyst was highly effective for the room temperature BHA reaction of a wide range of aryl and heteroaryl chlorides with five- or six-membered ring heteroaryl amines. Based on DFT studies, the authors proposed that the sterically induced effect of the phenyl rings renders the NHC ligand more electron-donating. The steric effect of phenyl groups controls the rotation of the N-wingtip substitution, which in turn affects the ligand coordination sphere. The computations also showed that the energy barriers for the oxidative addition step between SIPr^Ph2^ and SIPr-based catalysts were similar; however, the steric hindrance of the SIPr^Ph2^ ligand significantly reduced the energy barrier of the reductive elimination step, which was proposed to be the rate-determining step. Notably, this coupling was employed for the direct room temperature amination of pharmaceuticals, such as piribedil, sonidegib, brexpiprazole, and buspar, as well as drug candidates, such as ^V600E^BRAF inhibitor and 517-β-hydroxysteroid dehydrogenase inhibitor. Subsequently, the same catalyst, [Pd­(SIPr^Ph2^)­(cin)­Cl] was employed for a solvent-free BHA reaction of heteroaryl chlorides with various amines (not shown).^155^

**Scheme 37:**
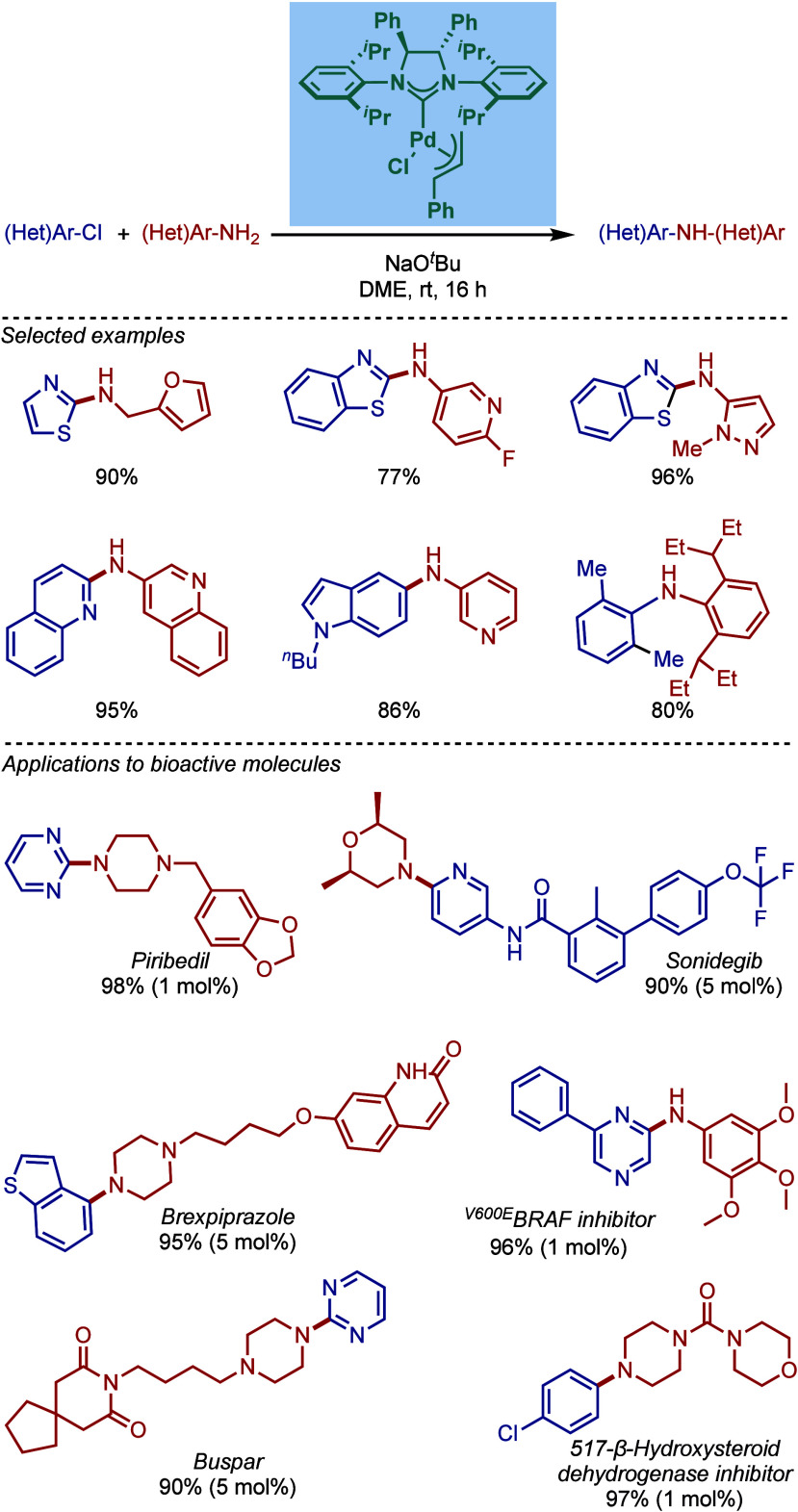
BHA Reaction Catalyzed by Backbone-Modified [Pd­(SIPr^Ph2^)­(cin)­Cl] by Qiu

In 2022, Nolan, Cazin, and co-workers reported the synthesis of [Pd­(NHC)­(1-*t*Bu-ind)­Cl] complexes containing saturated and unsaturated NHC ligands (NHC = IPr, IPr^Cl^, IMes, SIMes, IPr*) using a weak base route (Scheme 38).^156^ Previously, the synthesis of [Pd­(IPr)­(1-*t*Bu-ind)­Cl] had been reported by Hazari and co-workers using free NHC and [(1-*t*Bu-ind)­Pd­(μ-Cl)]_2_.^157^ As a major practical synthetic step forward, these complexes can now be synthesized directly in the reaction of [Pd­(1-^*t*^Bu-ind)­(μ-Cl)]_2_ and NHC salts under mild basic conditions. These complexes were evaluated in the BHA reaction of aryl chlorides and secondary amines. Interestingly, [Pd­(IPr^Cl^)­(1-*t*Bu-ind)­Cl] complex was found to be the most active catalyst, while the smaller metal-bearing and saturated and unsaturated IMes and SIMes congeners and sterically demanding [Pd­(IPr*)­(1-*t*Bu-ind)­Cl] were less effective. This observation may very well hint at the more difficult activation involving a bulkier allyl fragment as the [Pd­(IPr*)­(cin)­Cl] complex behaves so extremely well in most BHA reactions.

**Scheme 38:**
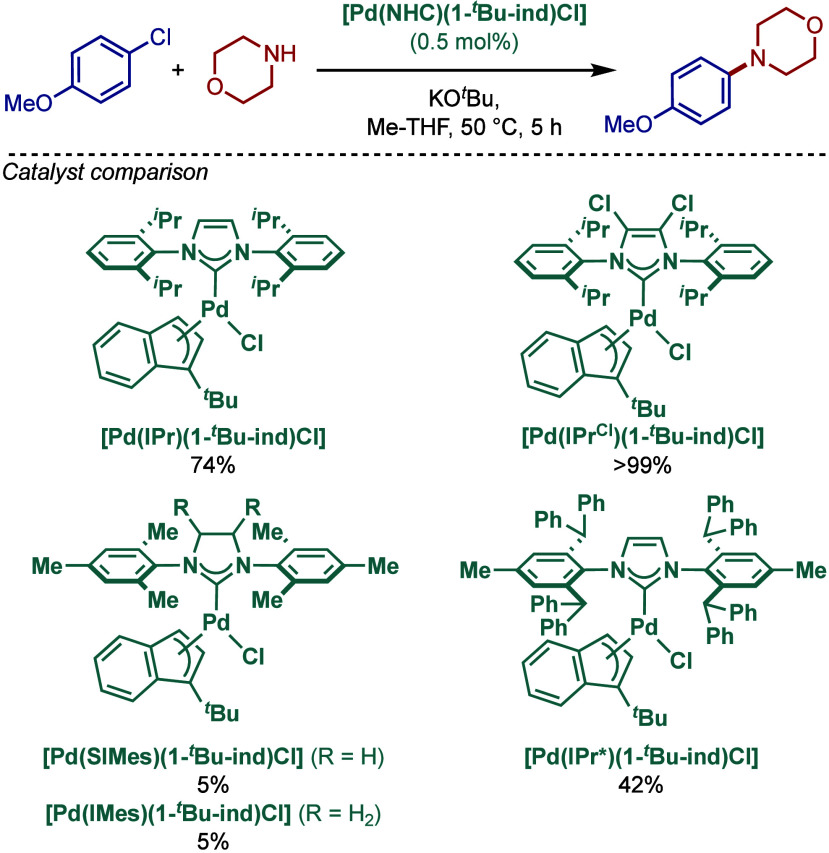
BHA Reaction using [Pd­(NHC)­(1-*t*Bu-ind)­Cl] Complexes by Nolan and Cazin

In 2022, Osipov and co-workers reported Buchwald–Hartwig cross-coupling reaction of 5-amino-1,2,3-triazoles with aryl halides to access 5-arylamino-1,2,3-triazole derivatives, a class of compounds with a prominent importance in medicinal chemistry (Scheme 39A).^158^ Different substituted 5-arylamino-1,2,3-triazoles were synthesized in excellent yields using the ring-expanded [Pd­(THP–Dipp)­(cin)­Cl] catalyst.

**Scheme 39:**
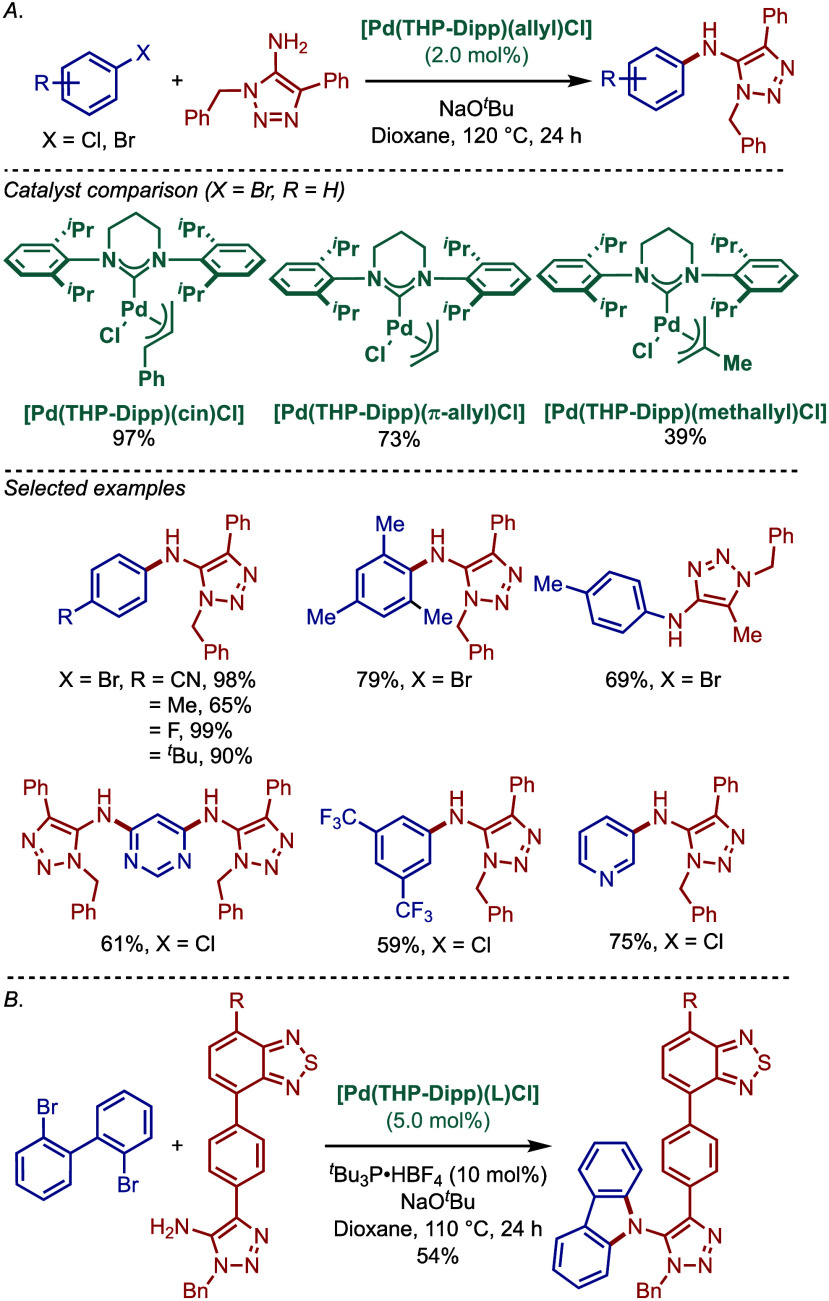
Synthesis of 5-Arylamino-1,2,3-Triazoles via BHA Reaction Catalyzed by [Pd­(THP–Dipp)­(cin)­Cl] by Osipov

In a subsequent study, the same group reported the synthesis of 5-arylamino-1,2,3-triazoles containing 2,1,3-benzothiadiazole moiety (Scheme 39B).^159^ This reaction could also be used to directly install carbazoles through double amination.

#### 2.3.2 [Pd­(NHC)­(μ-Cl)­Cl]_2_ Complexes

In 2002, Nolan and co-workers reported a remarkable air-stable NHC–palladium dichloride dimer, [Pd­(IPr)­(μ-Cl)­Cl]_2_, and its application in BHA reaction (Scheme 40A).^160^ Now, these [Pd­(NHC)­(μ-Cl)­Cl]_2_ have been established as the most reactive Pd­(II)–NHC precatalysts developed to date for a variety of cross-coupling reactions by C–X, C–O, C–N, C–S activation, where the complexes readily dissociate to monomers and are readily activated to Pd(0)–NHCs.^161^

**Scheme 40:**
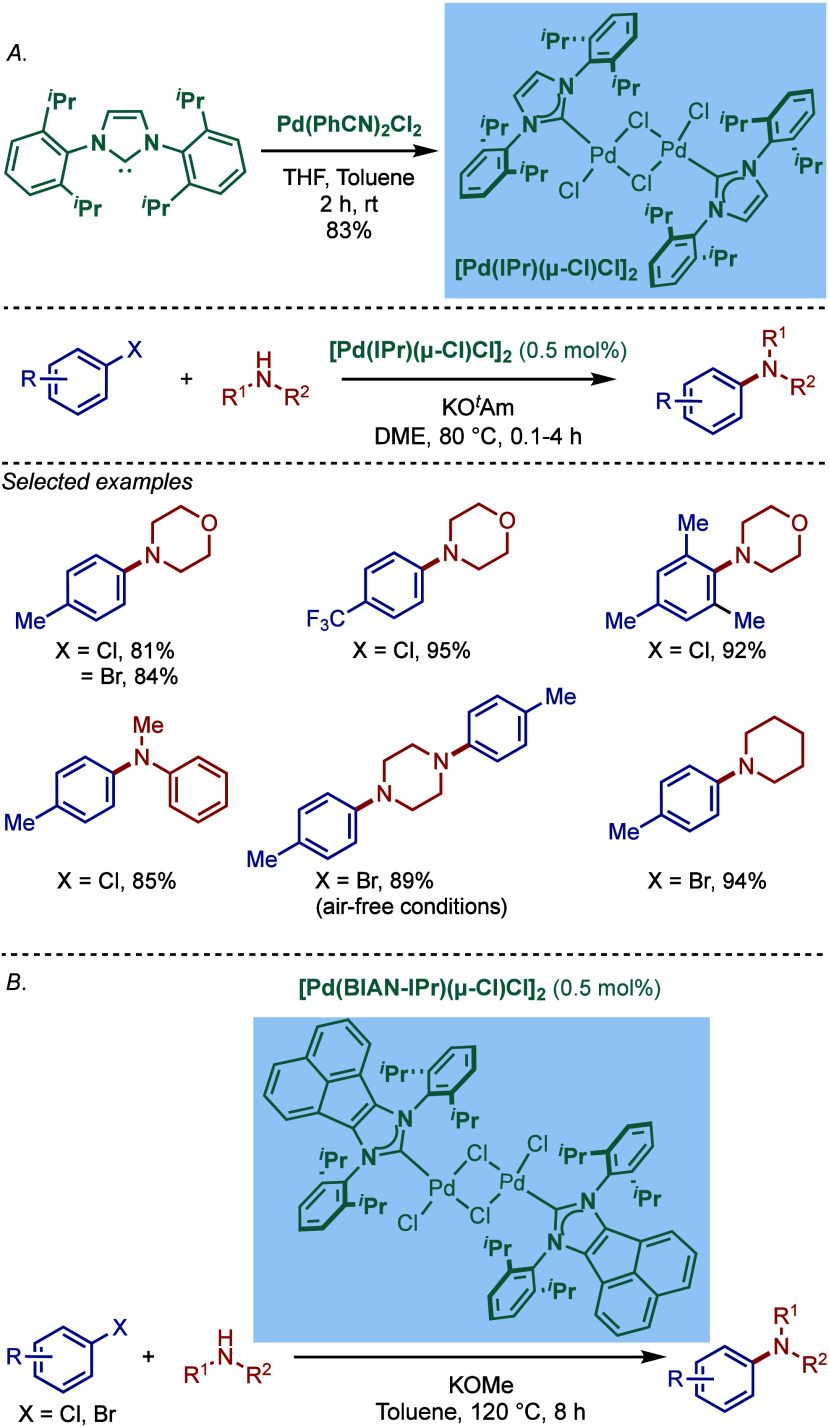
BHA Reaction of Aryl Chlorides Catalyzed by (A) Dimeric [Pd­(IPr)­(μ-Cl)­Cl]_2_ Complex by Nolan; (B) Dimeric [Pd­(BIAN–IPr)­(μ-Cl)­Cl]_2_ by Zhang

This [Pd­(IPr)­(μ-Cl)­Cl]_2_ complex was synthesized by the addition of free IPr to PdCl_2_(MeCN)_2_. The geometry at the palladium centers was found to be distorted square planar with all Pd and chloride atoms coplanar, while the N-aryl wingtip groups were aligned perpendicular to each other. This dimeric [Pd­(IPr)­(μ-Cl)­Cl]_2_ complex promoted the amination of aryl chlorides and bromides with a variety of amines under air, showing outstanding tolerance to oxygen and moisture.

Recently, Zhang, Szostak, and co-workers reported a related dimeric BIAN–IPr complex, [Pd­(BIAN–IPr)­(μ-Cl)­Cl]_2_,^162^ which showed even higher reactivity in the Buchwald–Hartwig cross-coupling of aryl halides, including diaminations and direct functionalization of pharmaceuticals (Scheme 40B).^163^ This BIAN–IPr dimer mergers the reactive properties of well-defined [Pd­(NHC)­(μ-Cl)­Cl]_2_ complexes with the steric protection of the BIAN scaffold, resulting in one of the most reactive Pd­(II)–NHCs for BHA reactions reported to date.

#### 2.3.3 [Pd­(NHC)­(R)­Cl] Palladacycle Complexes

In 2007, Wu and co-workers reported a novel air- and moisture-stable [Pd­(IPr)­(R)­Cl] cyclopalladated complex of ferrocenylimine and evaluated its reactivity in BHA reaction of aryl chlorides (Scheme 41).^164,165^ This complex was synthesized from the reaction of free carbene and cyclopalladated ferrocenylimine dimer. The catalyst proved to be highly efficient in the BHA reaction of aryl chlorides with 1° and 2° amines at 1 mol % catalyst loading in the presence of KO^*t*^Bu as a base in dioxane at 110 °C.

**Scheme 41:**
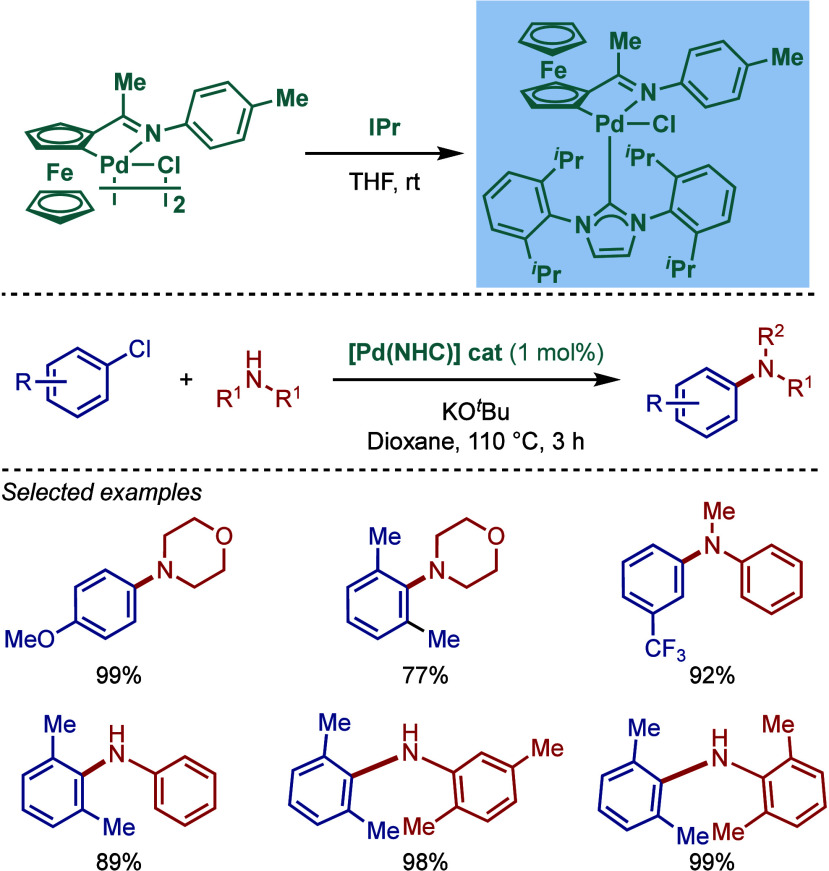
BHA Reaction Catalyzed by NHC–Cyclopalladated Ferrocenylimine Complex by Wu

The use of this catalytic system in a poly­(ethylene glycol-400) solvent was also reported, where it could be recycled and reused three times without a loss of catalytic activity (not shown).^166^

In 2003, Nolan and co-workers reported a new class of NHC N,N-dimethylbiphenylamine palladacycles, [Pd­(NHC)­(R)­Cl], and applied them in the BHA reaction of aryl chlorides and aryl triflates (Scheme 42).^167,168^ These palladacycles feature a square planar geometry around palladium, where the NHC ligand is trans to the amine around the palladium center. The synthesis proceeds readily by the reaction of free carbenes with palladacycle dimers. By varying the NHC ligand (NHC = IMes, IPr, SIMes, SIPr), the authors accessed different sterically- and electronically differentiated palladacycle–NHC catalysts. Mechanistically, catalyst activation is initiated in the presence of NaO^*t*^Bu, leading to aryl–alkoxy palladium species that are prone to reductive elimination ether under thermal conditions affording the active Pd(0)–NHC species stabilized by the electron-rich NHC ligand. The most reactive was the IPr-complex, affording high yields of the amination products using NaO^*t*^Bu in dioxane at 70 °C. We suspect the synthetic assembly of this catalyst family could also be easily achieved through the weak base route.

**Scheme 42:**
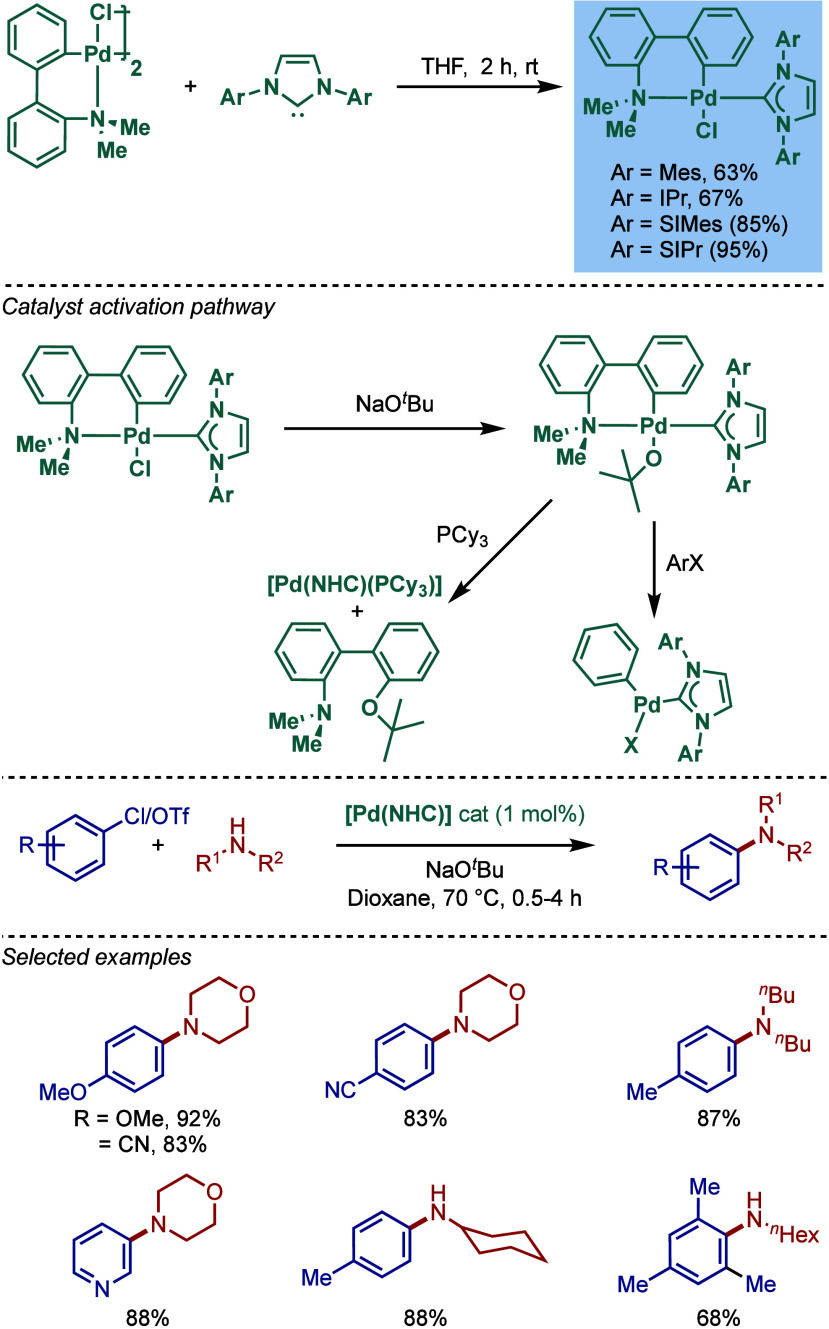
BHA Reaction Catalyzed by NHC–N,N-Dimethylbiphenylamine Palladacycle Complexes by Nolan

In 2017, Tu and co-workers reported a different class of cyclometalated [Pd­(NHC)­(R)­Cl] complexes based on the BIAN scaffold and N,N-diethylbenzylamine cyclopalladation (Scheme 43A).^169^ A catalyst comparison study in the BHA reaction using NaO^*t*^Bu in dioxane at 70 °C revealed that these BIAN–NHC palladacycles showed superior reactivity to their imidazol-2-ylidene as well as ancillary ligand congeners. This coupling methodology was successfully applied to the synthesis of rosiglitazone, an antidiabetic pharmaceutical.

**Scheme 43:**
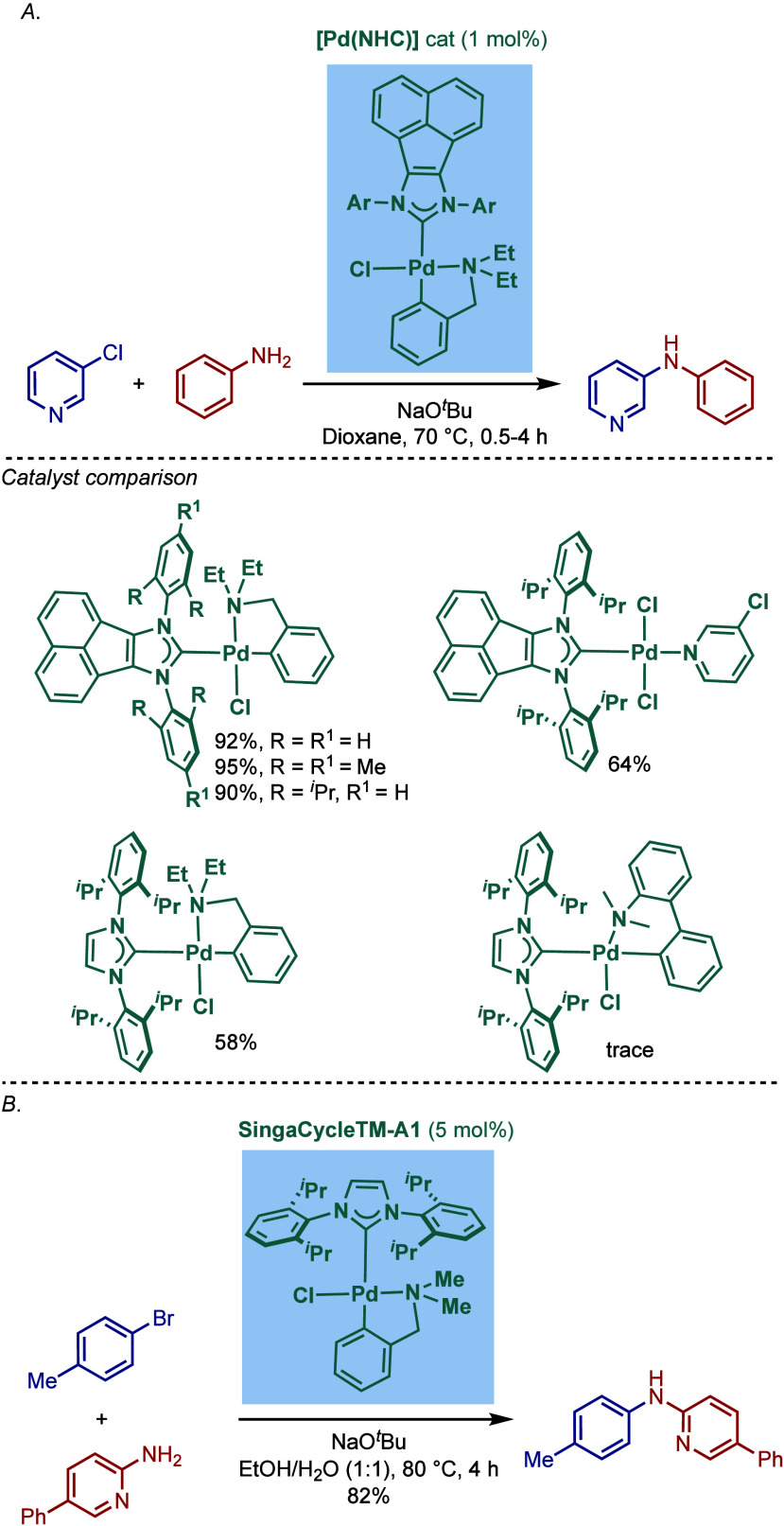
BHA Reaction Catalyzed by (A) BIAN–NHC–N,N-Diethylamine Palladacycles by Tu; (B) NHC–N,N-Dimethylamine Palladacycle by Reddy

Subsequently, Reddy and co-workers reported a related NHC–palladacycle, SingaCycle–A1, for BHA reaction of 2-aminopyridines (Scheme 43b).^170^ This class of Pd­(II)–NHC complexes was first introduced by Kantchev, Ying, and co-workers.^171^ The authors showed by competition experiments that SingaCycle–A1 was superior to phosphine-bearing systems in this amination. Furthermore, an alkoxy-modified N-heterocyclic carbene–palladacycle was reported by Deng and co-workers for the Buchwald–Hartwig cross-coupling, deploying the catalyst in the synthesis of Piribedil, a clinical drug for the treatment of Parkinson’s disease (not shown).^172^

In 2018, Lu and co-workers reported a [Pd­(NHC)­(R)­Cl] palladacycle based on benzo­[*h*]­quinoline cyclometalation (Scheme 44).^173^ These complexes were readily obtained by the direct reaction of NHC·HCl salt (NHC = IPr, IMes) with benzo­[*h*]­quinoline in the presence of K_2_CO_3_ in THF at 50–90 °C. These catalysts are air- and moisture-stable and were shown to be highly effective in the BHA reaction of aryl chlorides even at 0.01 mol % catalyst loading for sterically hindered substrates.

**Scheme 44:**
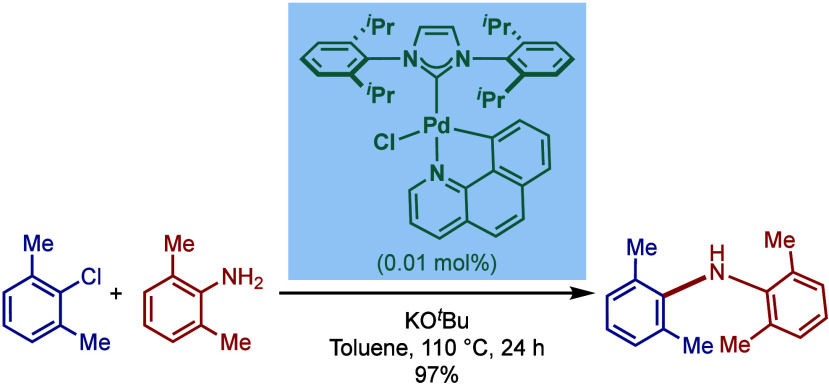
BHA Reaction Catalyzed by NHC–Benzo­[*h*]­quinoline Palladacycles by Lu

In an alternative approach, in 2023, Nolan and co-workers reported air- and moisture-stable palladate complexes bearing a 2-aminobiphenyl backbone and an imidazolium counterion (Scheme 45).^174^ The imidazol-2-ylidene analogue, [IPr·H]­[Pd­(R)­Cl_2_] was successfully employed in the BHA reaction of aryl chlorides with 1° and 2° amines.

**Scheme 45:**
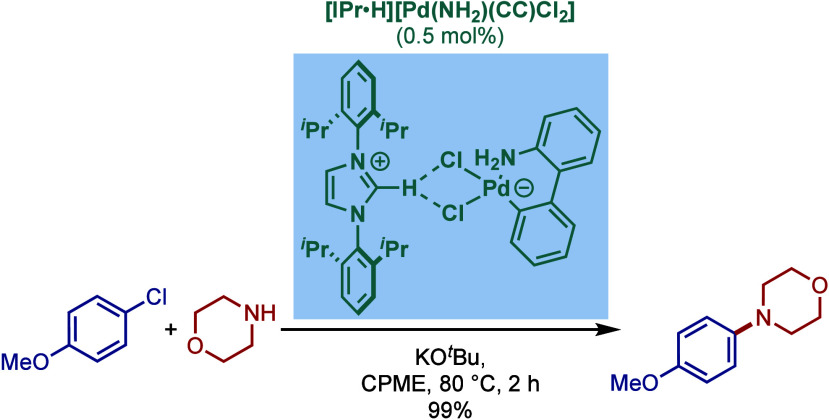
BHA Reaction Catalyzed by Palladate Complexes [NHC·H]­[Pd­(R)­Cl_2_] by Nolan

#### 2.3.4 [Pd­(NHC)­(acac)­Cl] Complexes

Acetylacetonate (acac) and other β-carbonyl compounds have been used as versatile ligands for the stabilization of Pd­(II)–NHC complexes. In 2005, Nolan and co-workers reported the first synthesis of [Pd­(NHC)­(acac)­Cl] complexes (Scheme 46A).^175^ This method follows a modified procedure used for the analogous [Pd­(PR_3_)­(acac)_2_] complexes.^176^ Thus, the direct reaction between free IPr and Pd­(acac)_2_ provided the [Pd­(IPr)­(acac)_2_] complex, which was characterized by X-ray analysis with slightly distorted square planar geometry around the palladium center. The addition of HCl in anhydrous dioxane resulted in consecutive oxidative addition/reductive elimination to afford [Pd­(IPr)­(acac)­Cl] complex.

**Scheme 46:**
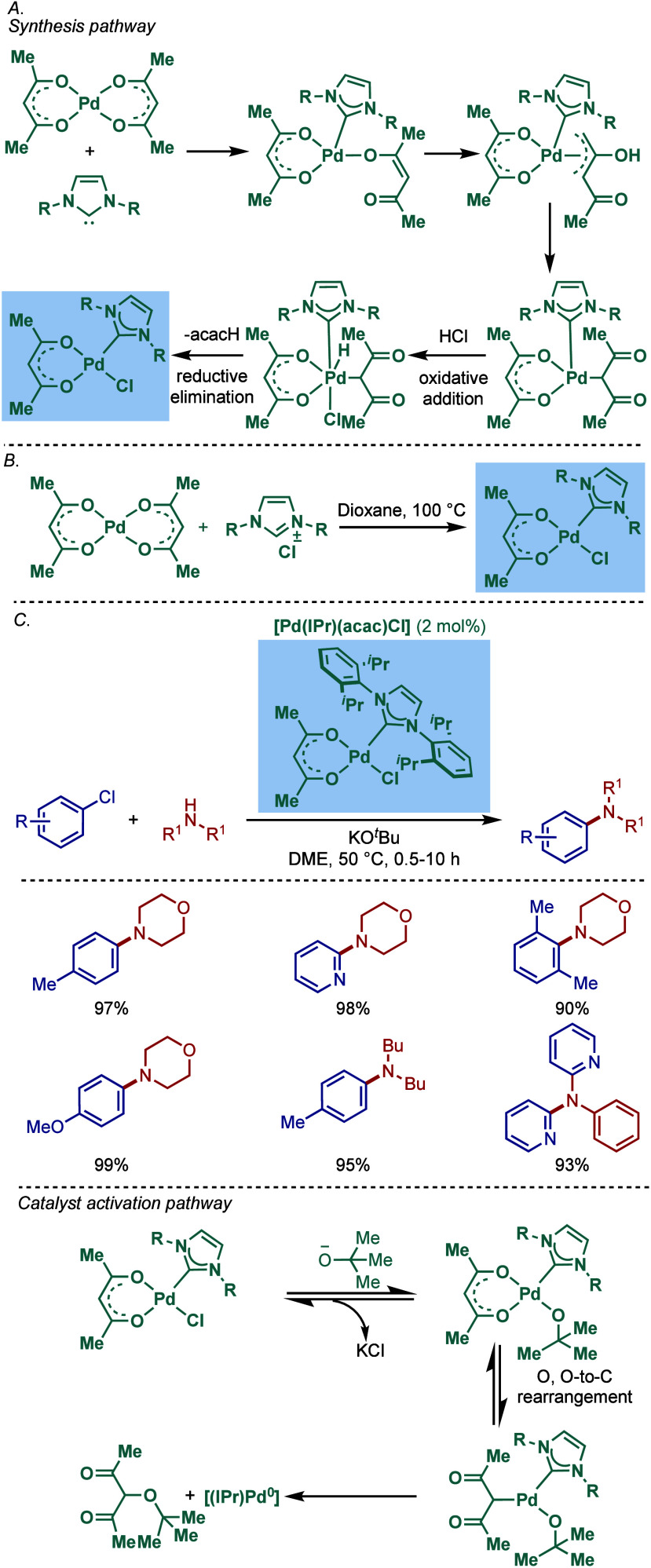
Synthesis of [Pd­(NHC)­(acac)­Cl] Complexes and Application in BHA Reaction by Nolan

Subsequently, Nolan and co-workers reported a very straightforward route to [Pd­(IPr)­(acac)­Cl] involving a direct reaction of [Pd­(acac)_2_]­with a slight excess of IPr·HCl in 1,4-dioxane at reflux (Scheme 46B).^177^ This procedure represents the most straightforward synthesis of any of the well-defined Pd­(II)–NHC complexes prepared to date, and considering the high reactivity of [Pd­(NHC)­(acac)­Cl], these complexes should be routinely considered for catalytic applications (Scheme 47C). Mechanistically, the bound acac serves as an internal base leading to the liberation of acacH permitting the binding of the chloride originating from the imidazolium salt. The air-stable [Pd­(IPr)­(acac)­Cl] complex was synthesized on multigram-scale and showed excellent catalytic activity in the BHA reaction of aryl and heteroaryl chlorides and bromides with 1° and 2° amines. In catalyst comparison studies, [Pd­(IPr)­(acac)­Cl] was more effective than its less sterically hindered congener, [Pd­(IMes)­(acac)­Cl].^178^

**Scheme 47:**
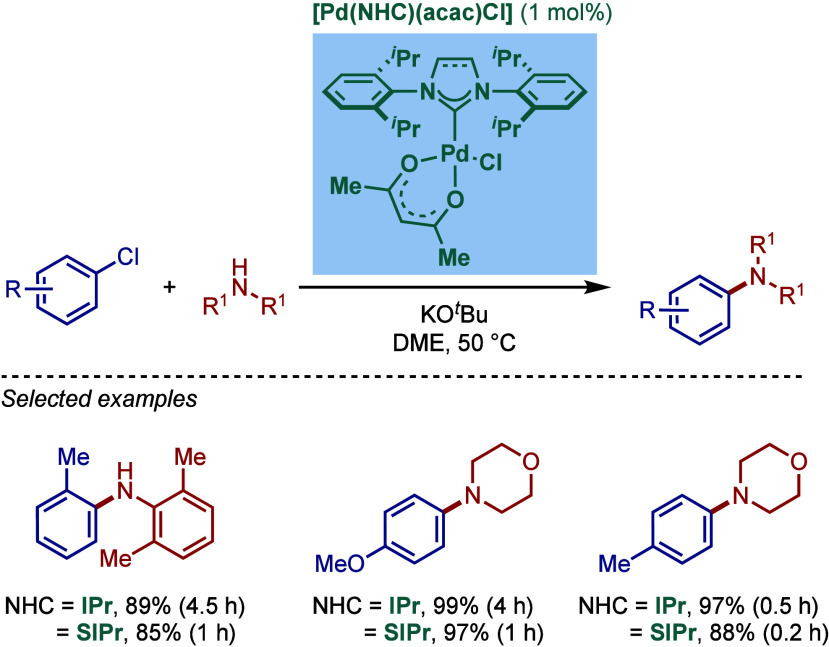
Comparative Reactivity of [Pd­(SIPr)­(acac)­Cl] and [Pd­(IPr)­(acac)­Cl] Complexes in BHA Reaction by Navarro

In 2009, Navarro and co-workers evaluated the catalytic difference in BHA reaction of aryl chlorides between the saturated imidazolin-2-ylidene-based complex, [Pd­(SIPr)­(acac)­Cl], and its unsaturated imidazol-2-ylidene congener, [Pd­(IPr)­(acac)­Cl] (Scheme 47).^179^ The authors found that the more electron-rich [Pd­(SIPr)­(acac)­Cl] showed better reactivity using comparatively mild conditions at 1 mol % catalyst loading with KO^*t*^Bu at 50 °C, which is in line with the reactivity trend observed earlier in [Pd­(NHC)­(allyl)­Cl] complexes. This reactivity was further explained by the greater steric demand of SIPr compared to IPr around the palladium center based on X-ray crystallographic studies and variable temperature NMR studies,

The effect of the acetylacetonate (acac) substitution on the catalytic activity of [Pd­(NHC)­(acac)­Cl] complexes in BHA reaction was further evaluated by Nolan and co-workers (Scheme 48).^180^ Inspired by their earlier work on the effect of allyl substitution in [Pd­(NHC)­(allyl)­Cl] complexes (see Section 2.3.1),^44a^ four different acac-substituted complexes were synthesized, including dibenzoylmethanato (dbm), benzoylacetonato (bac), tetramethylheptanedionato (tmhd), and hexafluoroacetylacetonato (hfac) complexes. The synthesis was again straightforward by using both the free carbene procedure and NHC salt procedure in 87–94% overall yields. According to the proposed activation pathway (Scheme 47), increased steric hindrance of the ancillary ligand resulted in a faster activation. As a result, the strongly electron-withdrawing but sterically less-hindered complex, [Pd­(IPr)­(hfac)­Cl], proved less effective, while the most sterically demanding complex, [Pd­(IPr)­(tmhd)­Cl], was most effective in the BHA reaction. Interestingly, [Pd­(IPr)­(hfac)Cl and [Pd­(IPr)­(acac)­Cl] showed similar efficacy.

**Scheme 48:**
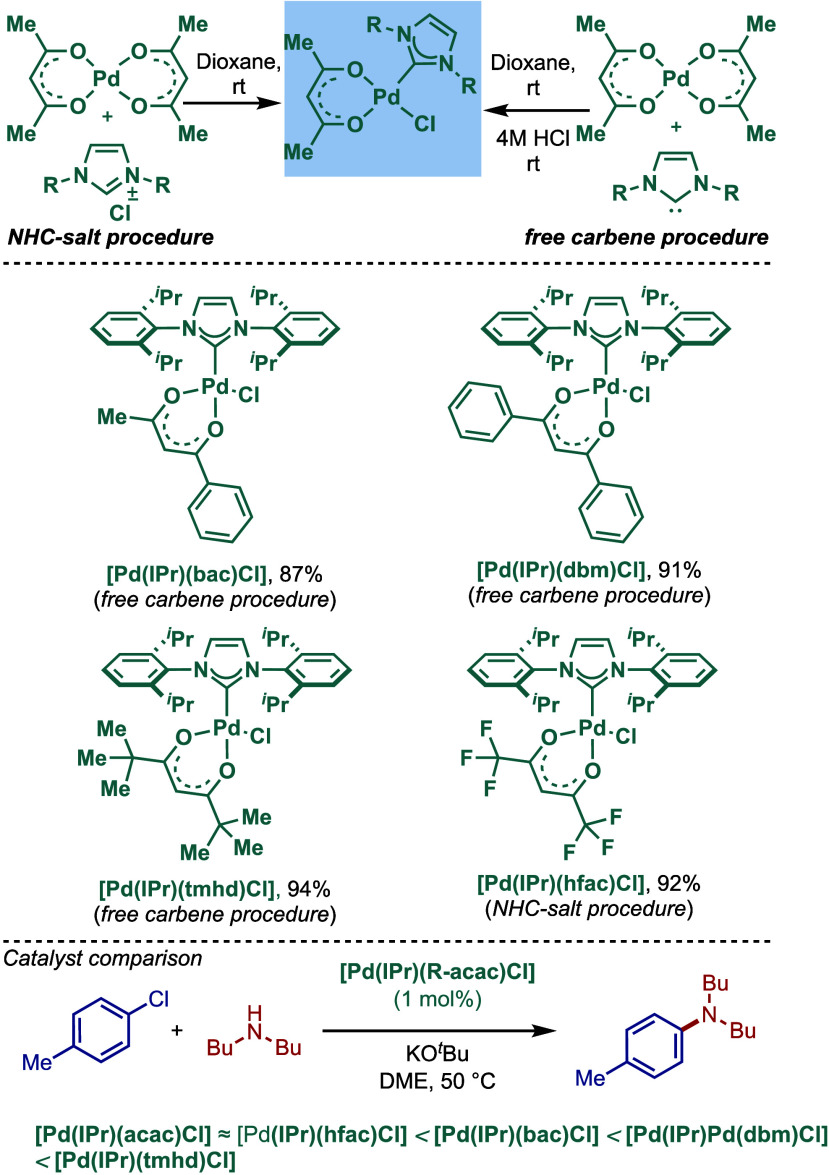
Effect of Acac Substitution on [Pd­(NHC)­(R-acac)­Cl] Complexes in BHA Reaction by Nolan

In 2012, Nolan and co-workers reported another modification of [Pd­(NHC)­(acac)­Cl] catalysts by tuning the catalyst efficiency through wingtip modification using sterically demanding IPr* ligand (Scheme 49).^181^ This extremely bulky-yet-flexible IPr* carbene was used to directly synthesize the corresponding air- and moisture-stable [Pd­(IPr*)­(acac)­Cl] complex through the reaction of Pd­(acac)_2_ and IPr*·HCl. This catalyst showed high reactivity in BHA reaction of sterically hindered and electronically deactivated substrates using LiHMDS in dioxane.

**Scheme 49:**
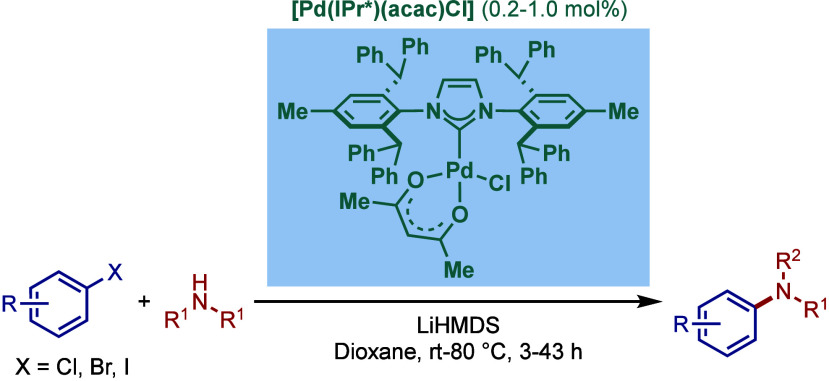
BHA Reaction Using Bulky-Yet-Flexible [Pd­(IPr*)­(acac)­Cl] Complex by Nolan

Another variant of this bulky-yet-flexible catalyst, featuring more electron-rich N-aryl wingtip, [Pd­(IPr*^OMe^)­(acac)­Cl], was reported in 2013 (Scheme 50).^182^ This catalyst showed a very high activity in BHA reaction at 0.05 mol % catalyst loading. This catalyst was shown to be superior to the previously synthesized [Pd­(IPr*)­(acac)­Cl] catalyst.

**Scheme 50:**
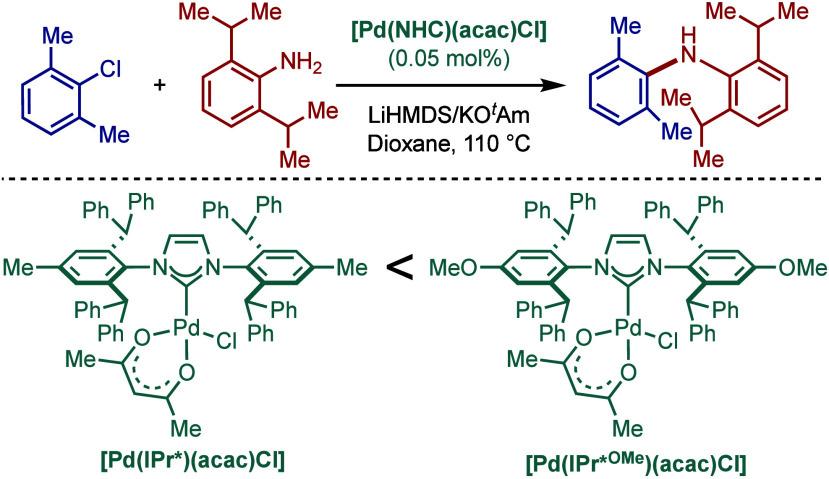
BHA Reaction Using Electron-Rich and Bulky-Yet-Flexible [Pd­(IPr*^OMe^)­(acac)­Cl] by Nolan

Furthermore, in 2018, Wang and co-workers reported another modification by tuning of the *para*-position of the IPr* ligand with isopropyl and *tert*-butyl substitution to give [Pd­(IPr*^iPr^)­(acac)­Cl] and [Pd­(IPr*^tBu^)­(acac)­Cl] catalysts, which also showed better reactivity than [Pd­(IPr*)­(acac)­Cl] in BHA reaction using LiHMDS in dioxane (not shown).^183^

In 2013, the Nolan group reported a sterically modified series of [Pd­(ITent)­(acac)­Cl] catalysts by wingtip modification, (Scheme 51).^184^ Complexes with variable length of the *ortho*-positions of the N-aromatic wingtips were synthesized, [Pd­(IPent))­(acac)­Cl], [Pd­(IHept)­(acac)­Cl] and [Pd­(INon)­(acac)­Cl]. These bulky-yet-highly flexible NHC ligands were accessed by an eight-step synthesis starting from inexpensive and readily available 2,6-dimethylnitrobenzene on multigram scale. The steric demand was comprehensively evaluated through the percent buried volume (%V_*bur*_) (IPent, %V_*bur*_ = 46.6; IHept, %V_*bur*_ = 45.7; INon, %V_*bur*_ = 43.7), which was reasonably greater than the parent IPr ligand (%V_*bur*_ = 37.4). Furthermore, TEP values indicated that IPent (2049.3 cm^–1^), IHept (2048.6 cm^–1^) and INon (2048.5 cm^–1^) are more σ-donating than the parent IPr ligand (2051.5 cm^–1^).^185^ In the same year, more electron-rich analogues bearing the *para*-OMe substitution of the N-aromatic wingtips were synthesized and evaluated in BHA reaction.^186^ It was found that [Pd­(IHept^OMe^)­(acac)­Cl] was the most reactive in the coupling of 4-chloroanisole and 4-fluoroaniline using KO^*t*^Am in toluene at 80 °C at 0.05 mol % loading. In general, more electron-rich ITent^OMe^ ligands performed better than ITent ligand. The study also revealed that IPr was completely ineffective under these conditions (Table 3).

**Scheme 51:**
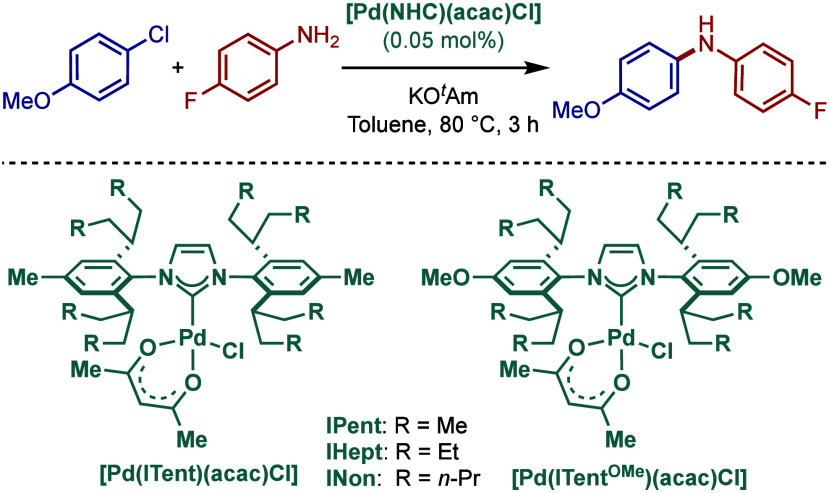
BHA Reaction Catalyzed by [Pd­(ITent)­(acac)­Cl] Complexes by Nolan

**Table 3:** Comparison between Different NHCs in [Pd­(NHC)­(acac)­Cl] Complexes in BHA Reaction by Nolan

entry	NHC	yield (%)
1	IPr	0
2	IPent	58
3	IPent-OMe	70
4	IHept	82
5	IHept-OMe	98
6	INon	76
7	INon-OMe	86

#### 2.3.5 [Pd­(NHC)­(PR_3_)_2_Cl] Complexes

In 2019, Mani and co-workers reported a cationic [Pd­(6-Dipp-PR_3_)­Cl]­BF_4_ pincer complex and evaluated its reactivity in the BHA reaction of aryl bromides (Scheme 52).^187^ Interestingly, this complex was directly accessible from the ring-expanded saturated precursor, 1,3-bis­(diphenylphosphanylmethyl)­hexahydropyrimidine. Based on DFT calculations, the authors proposed that the palladium carbene complex was formed through double C–H activation of methylene hydrogens with the liberation of H_2_. However, the reactivity was rather moderate using NaHMDS in toluene/dioxane at 100 °C for cross-coupling of aryl bromides.

**Scheme 52:**
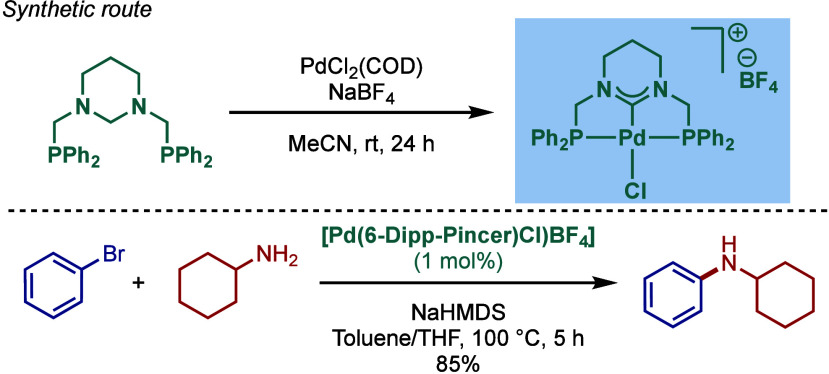
BHA Reaction using Mixed Cationic Hexahydropyrimidin-2-ylidene/Phosphine–Palladium Pincer Complex by Mani

Another class of mixed cationic phosphine–NHC complexes for BHA reaction was reported by Fürstner and co-workers (Scheme 53).^188^ They synthesized two different classes of Pd­(II)–NHC complexes through the oxidative insertion of [Pd­(PPh_3_)_4_] into the C–Cl bond of the corresponding 2-chloroimidazolinium or amidinium salts. The neutral and cationic complexes were found to be in equilibrium in NMR solution studies. These complexes showed high activity in BHA reaction of 2-halopyridines. [Pd­(NHC)­(PR_3_)­Cl_2_] complexes represent a prototype of Fischer carbene complexes.

**Scheme 53:**
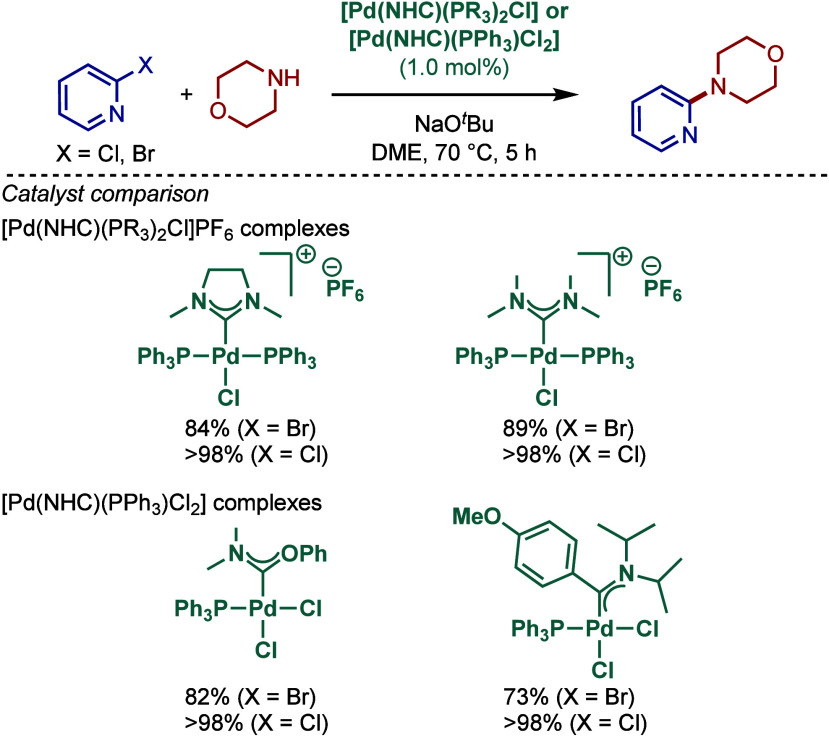
BHA Reaction Catalyzed by Diaminocarbene- and Fischer-Carbene Complexes by Fürstner

#### 2.3.6 [Pd­(NHC)­(PR_3_)­Cl_2_] Complexes

In 2018, Kim and co-workers reported a new class of mixed [Pd­(NHC)­(PR_3_)­Cl_2_] complexes featuring a combination of σ-donating NHCs and π-acceptor bicyclic bridgehead phosphoramidite (briphos) ligands (Scheme 54).^189^ These briphos ligands can be used to systematically tune steric and electronic properties of the complex. The evaluation of different [Pd­(IPr)­(briphos)­Cl_2_] complexes in BHA reaction of chlorobenzene revealed that briphos ligands substituted with 3,5-dimethylphenyl and cyclohexyl groups were the most efficient among the catalysts tested. Interestingly, the authors determined the relative binding affinity of phosphorus ligands and found that the cyclohexyl-substituted phosphine was strongly binding to palladium. This suggested that the binding affinity is not the major factor in catalytic activity, which involves phosphine dissociation to form the catalytically active monoligated Pd(0)–NHC. The scope of the BHA reaction was broad using KO^*t*^Bu in DME at 80 °C.

**Scheme 54:**
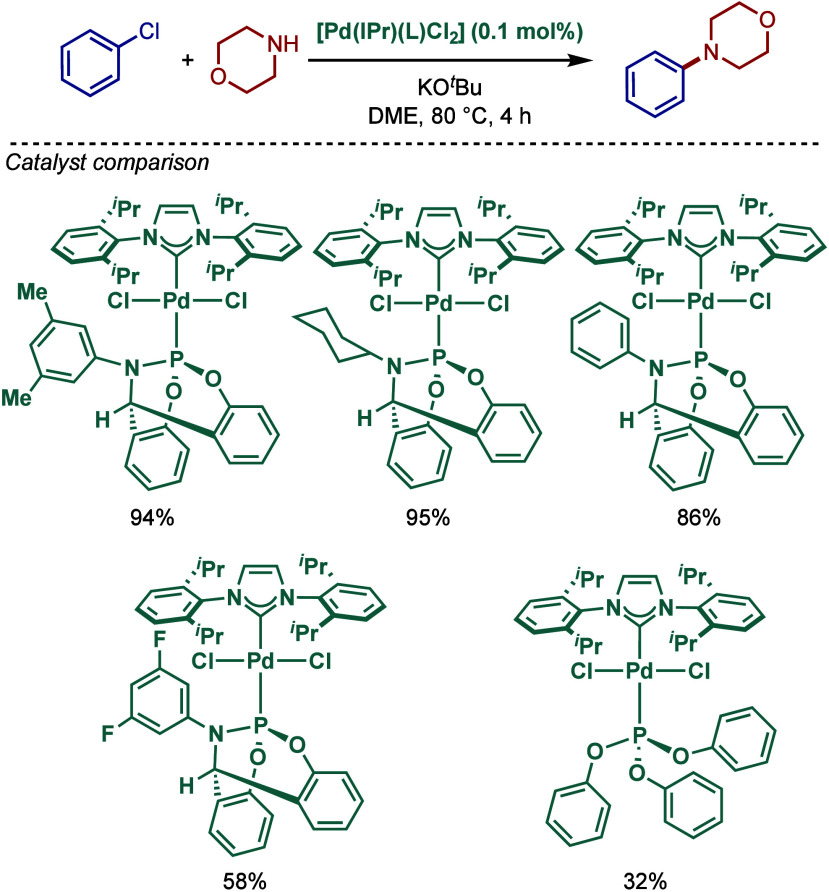
BHA Reaction Catalyzed by [(IPr)­Pd­(briphos)­Cl_2_] Complexes by Kim

The following year, Bermeshev and co-workers reported a series of mixed [Pd­(NHC)­(PR_3_)­Cl_2_] complexes and compared their reactivity in BHA reaction under solventless conditions (Scheme 55A).^190^ Five different NHCs (IPr, SIPr, IMes, SIMes, and 6-Dipp) and six different phosphines (PPh_3_, P­(*o*-Tol)_3_, SPhos, RuPhos, DavePhos, and CyJohnPhos) were investigated. The donating ability of phosphine ligands was gauged by ^13^C and ^31^P NMR spectroscopy and showed the following order: RuPhos > SPhos ∼ DavePhos > CyJohnPhos ≫ PPh_3_ > P­(*o*-Tol)_3_. The catalytic comparison in the BHA reaction of 1-bromonaphthalene and aniline showed that [Pd­(IMes)­(SPhos)­Cl_2_] and [Pd­(SIMes)­(SPhos)­Cl_2_] complexes were completely unreactive. In contrast, [Pd­(6-Dipp)­(SPhos)­Cl_2_], [Pd­(SIPr)­(SPhos)­Cl_2_], [Pd­(IPr)­(SPhos)­Cl_2_] showed high reactivity with the following order of efficiency: 6-Dipp > SIPr > IPr. Different [Pd­(6-Dipp)­(PR_3_)­Cl_2_] were evaluated and the reactivity was in the following order: SPhos = CyJohnPhos = P­(*o*-Tol)_3_ > RuPhos = DavePhos ≫ PPh_3_ (Table 4). The most reactive catalyst, [Pd­(6-Dipp)­(SPhos)­Cl_2_], was used for solvent-free BHA reaction of aryl chlorides and bromides using NaO^*t*^Bu at 110 °C as well as for the challenging di- and triaminative intermolecular and diarylative intramolecular coupling to afford carbazoles and related heterocycles (Scheme 55B).^191^

**Scheme 55:**
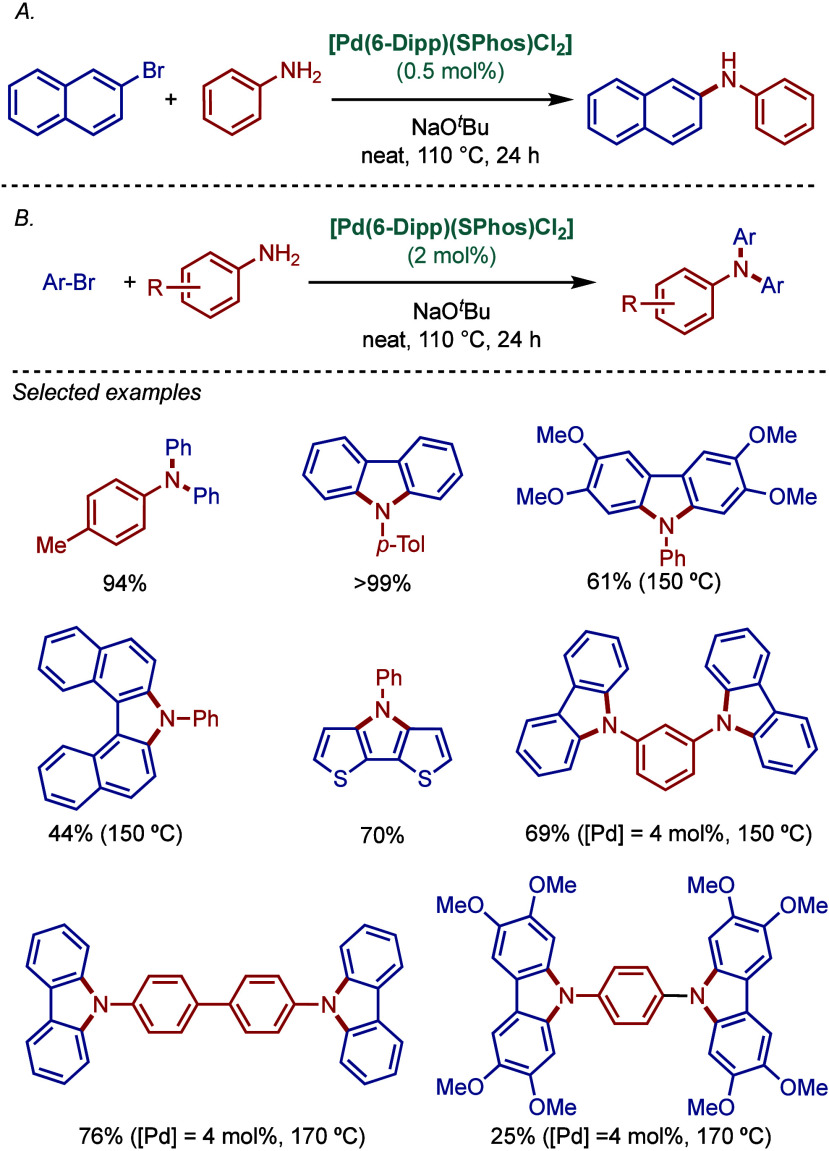
BHA Reaction Using Mixed [Pd­(NHC)­(PR_3_)­Cl_2_] by Bermeshev

**Table 4:** Comparison between Different Pd–NHC Complexes in BHA Reaction by Bermeshev

entry	catalyst	yield (%)
1	[Pd(6-Dipp)(SPhos)Cl_2_]	98
2	[Pd(IPr)(SPhos)Cl_2_]	86
3	[Pd(SIPr)(SPhos)Cl_2_]	90
4	[Pd(IMes)(SPhos)Cl_2_]	0
5	[Pd(SIPr)(SPhos)Cl_2_]	0
6	[Pd(6-Dipp)(RuPhos)Cl_2_]	86
7	[Pd(6-Dipp)(DavePhos)Cl_2_]	86
8	[Pd(6-Dipp)(PPh_3_)Cl_2_]	48
9	[Pd(6-Dipp)(P(*o*-Tol)_3_)Cl_2_]	98
10	[Pd(6-Dipp)(CyJohnPhos)Cl_2_]	98

In 2021, Nolan and co-workers reported a bulky 1,4,7-triaza-9-phosphatricyclo[5.3.2.1]­tridecane (CAP) ligand for the synthesis of mixed NHC/phosphine–palladium complexes (Scheme 56).^192^ The CAP ligand is characterized by strong electron-donating ability (TEP = 2056.8 cm^–1^) and reduced steric hindrance around phosphorus (cone angle = 109°). Four different NHC ligands (NHC = IPr, SIPr, IPr*, IPr*^OMe^) were selected to synthesize the corresponding [Pd­(NHC)­(CAP)­Cl_2_] complexes. The synthesis of [Pd­(NHC)­(CAP)­Cl_2_] was readily accomplished by ligand exchange from *trans*-[Pd­(NHC)­(py)­Cl_2_] or by the reaction of CAP with dimeric [Pd­(NHC)­Cl_2_]_2_ complexes. The evaluation of catalytic activity in the BHA reactions showed that [Pd­(IPr)­(CAP)­Cl_2_] was the most reactive complex at 0.5 mol % loading using NaO^*t*^Bu in THF at 80 °C.

**Scheme 56:**
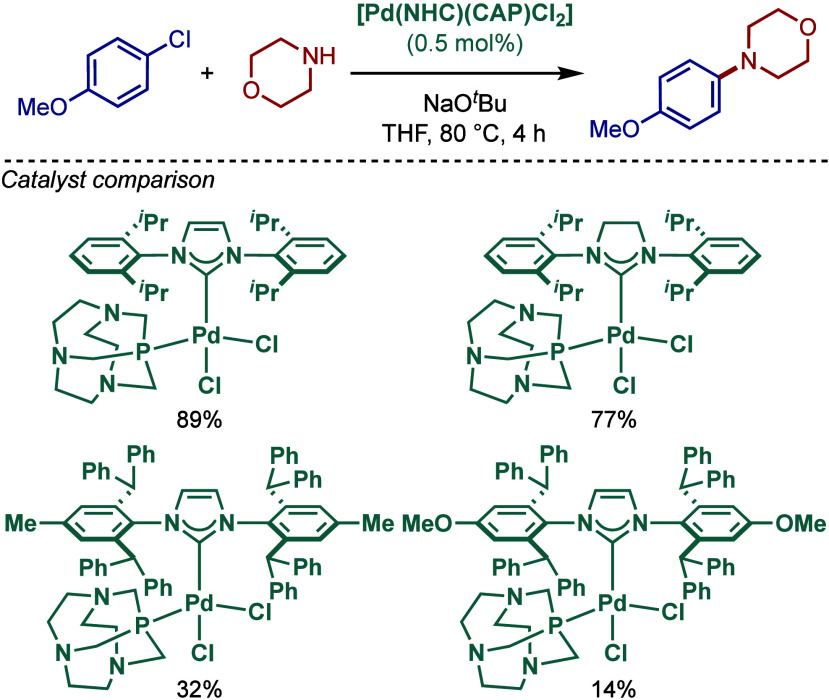
BHA Reaction Catalyzed by [Pd­(NHC)­(CAP)­Cl_2_] Complexes by Nolan

Wang and co-workers reported dinuclear N-heterocyclic carbene–palladium complexes, where two palladium­(II) centers were coordinated to bridging diphosphine ligands (not shown).^193^ These complexes showed good catalytic activity in BHA reaction under microwave irradiation conditions.

A related class of Pd–NHC complexes bearing intramolecular coordination of hemilabile morpholine was reported by Stradiotto (Scheme 57).^194^ The synthesis of monodentate [Pd­(NHC)­(cin)­Cl] complexes proceeds from the corresponding N-heterocyclic carbene ligands by the reaction with [Pd­(cin)­Cl]_2_ dimers. The morpholine moiety can then act in a bidentate coordination mode after cin displacement. These four complexes were evaluated in the BHA reaction of chlorobenzene using KO^*t*^Bu in toluene at 110 °C. The [Pd­(bidentate-SIPr)­Cl_2_] complex was the most reactive of the series; however, in general, these catalysts were less efficient compared to [Pd­(SIPr)­(cin)­Cl], indicating the importance of the steric-hindrance on both aromatic wingtips to provide a well-defined environment during the catalytic cycle.

**Scheme 57:**
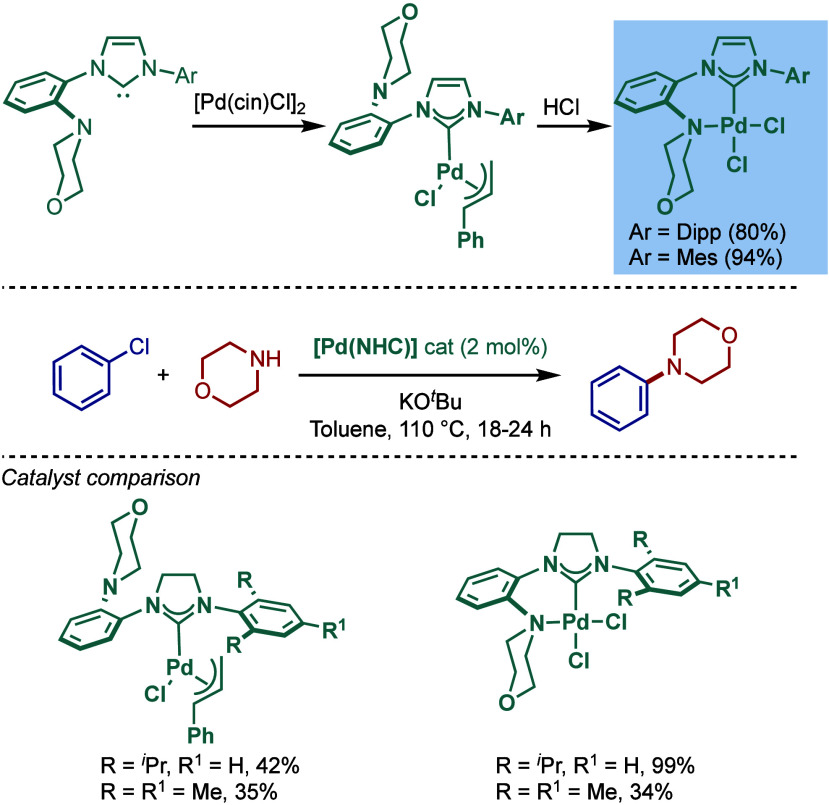
BHA Reaction using Morpholine-Functionalized Pd–NHC Complexes by Stradiotto

#### 2.3.7 [Pd­(NHC)­(μ-Cl)­R]_2_ Complexes

In 2008, Caddick, Cloke, and co-workers reported dimeric alkyl–palladium [Pd­(NHC)­(μ-Cl)­R]_2_ complexes (NHC = IPr, I*t*Bu) and evaluated their reactivity in BHA reaction (Scheme 58).^195^ These complexes were synthesized by alkylation of [Pd­(cod)­Cl_2_] followed by the addition of free carbenes. Interestingly, under the identical reaction conditions, IPr afforded [Pd­(IPr)­(*cis*-neopentyl)­(μ-Cl)]_2_ complex, while its N-alkyl congener, I*t*Bu, gave [Pd­(I*t*Bu)­(*trans*-neopentyl)­(μ-Cl)]_2_. In the presence of 1° and 2° amines, these chloride-bridged dimers were readily dissociated to [Pd­(NHC)­(NHRR′)­Cl­(R)] transamination products. The reactivity of these complexes was evaluated in BHA reaction of chlorobenzene, where the IPr complex showed higher reactivity using LiHMDS in toluene at 80 °C. Subsequently, this [Pd­(IPr)­(*cis*-neopentyl)­(μ-Cl)]_2_ complex was used to perform room temperature aminations of aryl chlorides, including deactivated and sterically hindered substrates.

**Scheme 58:**
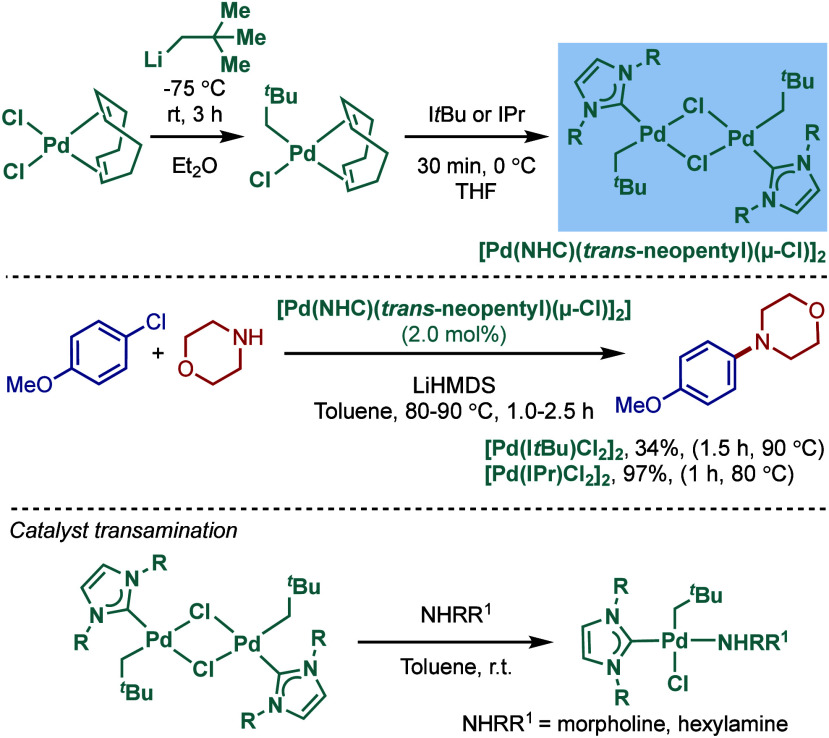
Synthesis of [Pd­(NHC)­(*trans*-neopentyl)­(μ-Cl)]_2_, Their Use in BHA Reaction, and Catalyst Comparison by Cloke, Caddick, and Co-workers

#### 2.3.8 [Pd­(NHC)­(3-Cl-py)­Cl_2_] Complexes

The clear advantages of Pd­(II)–NHC complexes in Buchwald–Hartwig cross-coupling reactions is their higher stability and an easier preparation than the analogous Pd(0)–NHC complexes. However, the use of Pd­(II)–NHC complexes must involve a mandatory activation step, to generate the monoligated Pd(0)–NHC species. Therefore, the presence of labile ancillary ligands on palladium with no significant rebinding capacity is necessary to facilitate the activation process. In this context, heterocycle-coordinated Pd­(II)–NHCs are among the most attractive complexes for BHA reactions. In 2002, Grubbs and co-workers showed that replacement of phosphorus ligands with pyridine-based ligands (py, 3-Br-py, 4-Ph-py) resulted in a remarkably more rapid initiation of at least 6 orders of magnitude in Ru–NHC-catalyzed olefin metathesis in the [Ru­(SIMes)­(=CHPh)­(Cl_2_)­(PR_3_)] system.^196^ The fastest initiation was observed for a bromopyridine ligand containing bromide at the C3-position of the pyridine ring. Taking this study into account, in 2006, Organ and co-workers reported air- and moisture-stable Pd­(II)–NHC complexes, [Pd­(NHC)­(3-Cl-py)­Cl_2_], with 3-chloro-pyridine as a labile ancillary ligand on palladium, and called them PEPPSI (Pyridine-Enhanced Precatalyst Preparation, Stabilization, and Initiation).^197^ This class of [Pd­(NHC)­(3-Cl-py)­Cl_2_] precatalysts was prepared using a very straightforward procedure, including heating of the corresponding NHC·HCl salt with PdCl_2_ in the presence of K_2_CO_3_ in 3-chloropyridine as a solvent, which afforded the products in 91%–98% yields on a multigram scale (Scheme 59). Furthermore, this method allowed the reaction to be carried out in air, while the pyridine solvent could be reused after distillation. An improved synthetic method to these complexes has been recently reported by Nolan that circumvents the use of such large volumes of pyridines.^198^ These [Pd­(NHC)­(3-Cl-py)­Cl_2_] precatalysts are advertised as benefiting from both fast dissociation of the electron-deficient 3-chloropyridine ligand and slow rebinding ability of the throw-away ligand, which has resulted in an explosion of interest in this class of Pd­(II)–NHC complexes in cross-coupling and other organic transformations. However, it should be clearly noted that in practice these [Pd­(NHC)­(3-Cl-py)­Cl_2_] precatalysts activate more slowly than the corresponding [Pd­(NHC)­(allyl)­Cl] and [Pd­(NHC)­(μ-Cl)­Cl]_2_ complexes (see Sections 2.3.1 and 2.3.2).

**Scheme 59:**
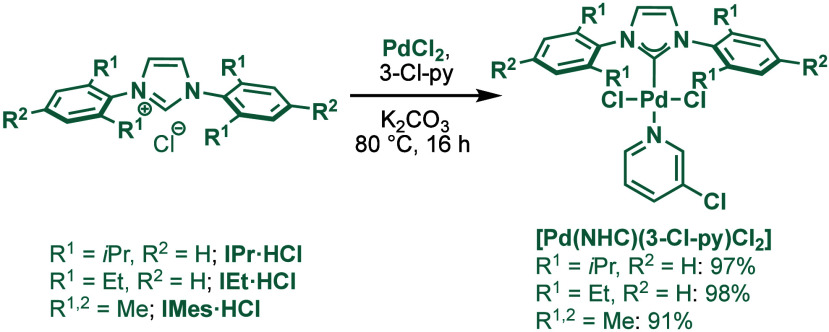
Synthesis of [Pd­(NHC)­(3-Cl-py)­Cl_2_] Complexes by Organ

In 2008, Organ and co-workers reported on a BHA reaction of aryl halides mediated by [Pd–PEPPSI–NHC] precatalysts (Scheme 60).^199^ Pd­(II)–NHC complexes containing IMes, IEt, IPr, and SIPr ligands were tested using different reaction conditions, such as solvents with different polarities (e.g., toluene, DME, DMSO) and base strength (e.g., KO^*t*^Bu, Cs_2_CO_3_). The best results were observed for the [Pd–PEPPSI–IPr] and [Pd–PEPPSI–SIPr] complexes, including the reactions carried out at room temperature, a result which was ascribed to the higher steric hindrance around the Pd center. Identical results as previous studies performed with other throw-away ligands (see above). The optimized protocol enabled efficient coupling of a wide range of aryl- and heteroaryl halides with various 1° and 2° aliphatic and aromatic amines using KO^*t*^Bu as a base in DME. The authors further extended the scope to more sensitive substrates using Cs_2_CO_3_ as a milder and functional group tolerant base in DME, which enabled the cross-coupling of heterocycles, such as quinoline, pyrazine, and tetrazole derivatives, in good to excellent yields. In the case of 3-halopyridines or 5-halopyrimidines, the use of more σ-donating imidazolin-2-ylidene-based SIPr and slow addition of electrophiles were necessary to obtain good yields. The authors hypothesized that this is likely due to the electronic properties of these N-heterocycles, which may behave as a catalyst inhibitor, leading to a decrease in the turnover frequency of the catalyst. The authors also noted that higher temperatures could lead to β-hydride elimination, which results in the reduction of aryl halides.

**Scheme 60:**
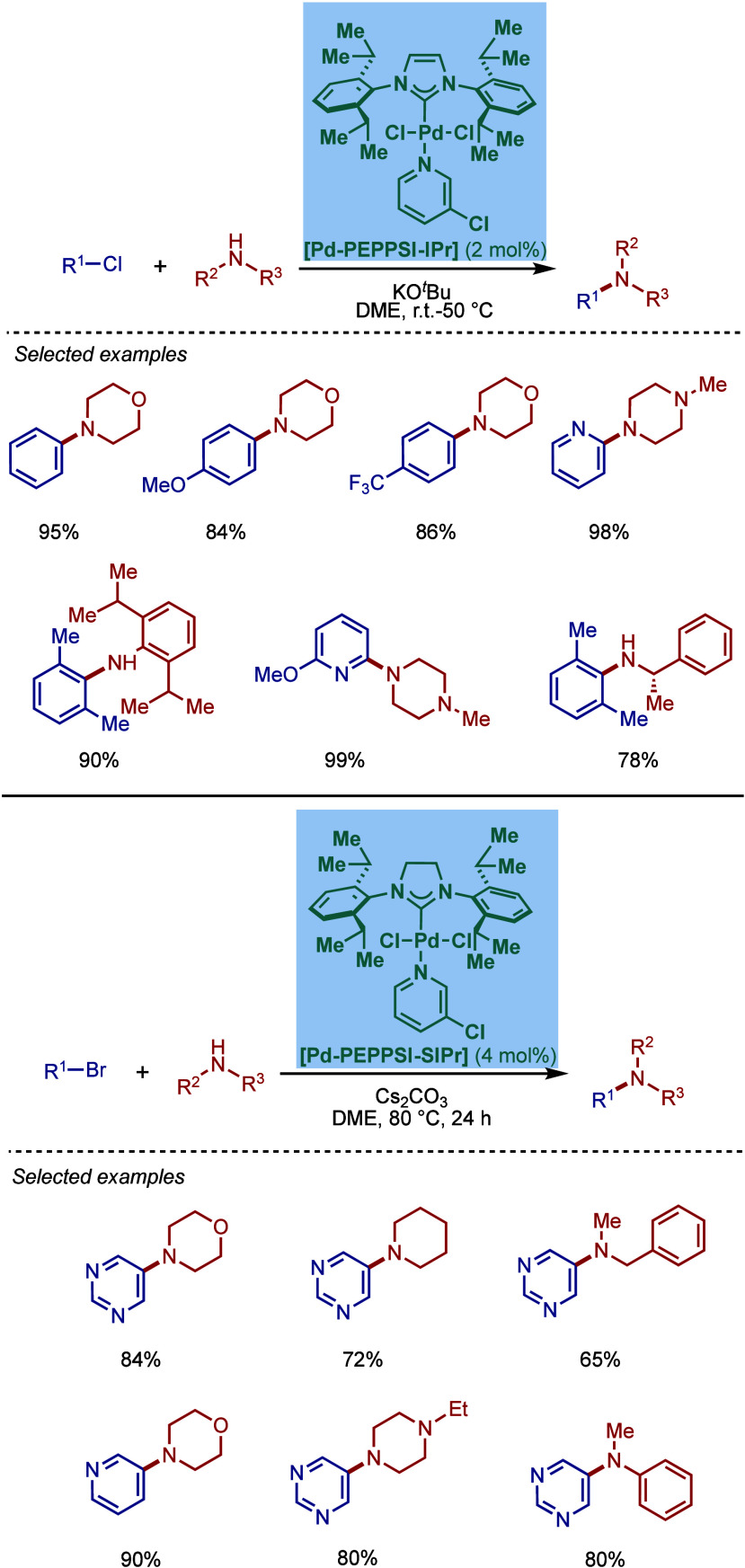
BHA Reaction Using [Pd–PEPPSI–IPr] and [Pd–PEPPSI–SIPr] Complexes by Organ

In 2008, Kirschning described the coordinative immobilization of [Pd–PEPPSI–IPr] on polyvinylpyridine (Scheme 61).^200^ This immobilization led to highly active heterogeneous Pd­(II)–NHC precatalyst for C–C and C–N cross-coupling reactions using standard as well as continuous flow conditions. Prior to this study, very few heterogeneous Pd–phosphine complexes for the BHA reaction of aryl halides were known due to their relatively low stability to air and moisture. Using the immobilized Pd–PEPPSI-type precatalyst (0.2 mol %), the reactions were carried out in DME at 50 °C and resulted in products in good to high yields. The use of potassium *tert*-pentoxide instead of potassium *tert*-butoxide enabled the use of a continuous flow method that led to products in a shorter time; however, this procedure was not effective for sterically hindered or less active aryl chlorides.

**Scheme 61:**
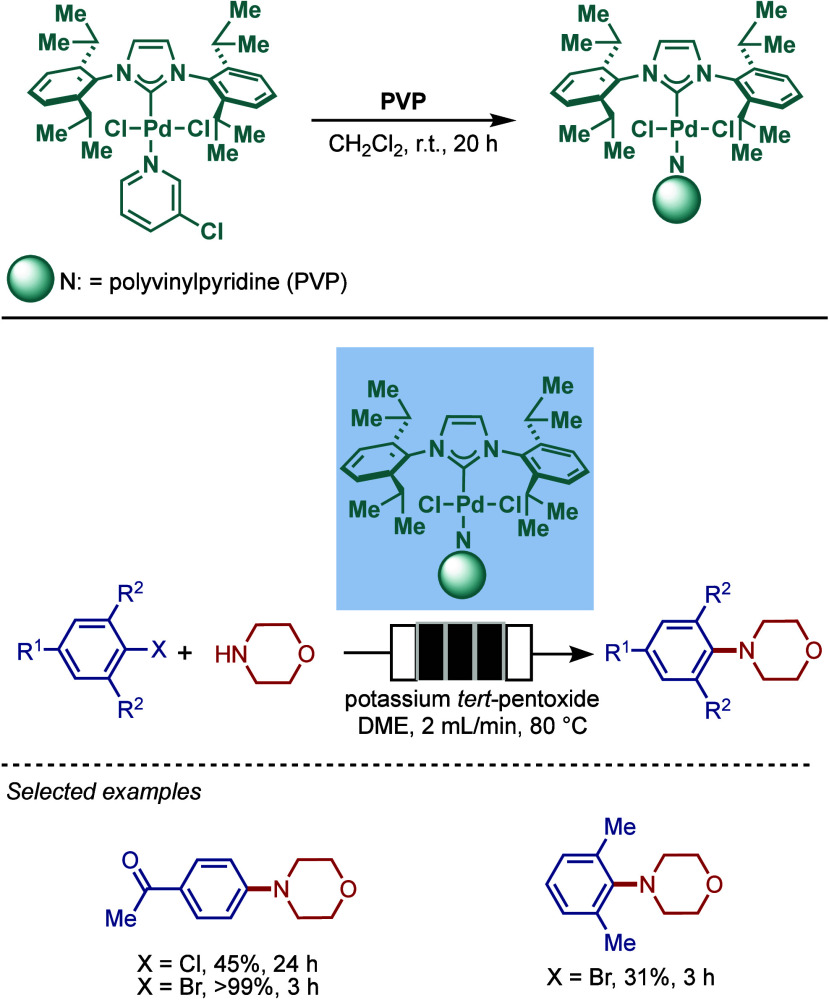
BHA Reaction Using Immobilized [Pd–PEPPSI–NHC] Precatalyst by Kirschning

In 2011, Organ and co-workers reported a comprehensive study on the evaluation of the steric and electronic properties of NHC ligands on catalyst performance in [Pd­(NHC)­(3-Cl-py)­Cl_2_] complexes using kinetic and computational studies (Scheme 62).^201^ The nature of the N-heterocyclic carbene ligand was found to have a key impact on the rate-limiting step of the catalytic cycle (Figure 4). In contrast to phosphine-type catalysts, Pd–NHC complexes can readily undergo oxidative addition even with unactivated aryl chlorides due to the strong σ-donating properties of N-heterocyclic carbenes. The previous results^199^ showed a significant impact of the electronic character of the aryl electrophile on the oxidative addition step. Furthermore, utilization of more sterically hindered NHC ligands enabled the cross-coupling under milder conditions. The experimental data suggested that there is a link between the steric properties of the ligand and the charge of the metal. Thus, the authors prepared more sterically hindered [Pd–PEPPSI–IPent] precatalyst (IPent = 2,6-diisopentylphenylimidazolium) and tested its impact on BHA reaction of different electron-donating and electron-withdrawing aryl chlorides. They found that the sterically bulkier IPent catalyst uniformly outperformed its IPr congener under the conditions examined. Furthermore, an increase in the concentration of aryl chloride resulted in a decrease of reaction rate, which suggested that the oxidative addition step is not rate-limiting. In the case of higher concentration of the amine component, the reaction rate increased only slightly, which was not sufficient to conclude that amine coordination was the rate-limiting step. In contrast, the amount of base had a significant effect on the reaction rate, which suggested that deprotonation could be involved in the rate-determining step. Using the optimized reaction conditions (Cs_2_CO_3_, DME, 80 °C), [Pd–PEPPSI–IPent] showed excellent reactivity in cross-coupling of various electronically substituted and sterically hindered aryl halides with 2° hindered amines under mild conditions, significantly outperforming [Pd–PEPPSI–IPr]. Again, these appear very special conditions as Cs_2_CO_3_ is not a commonly encountered base in BHA reactions.

**Scheme 62:**
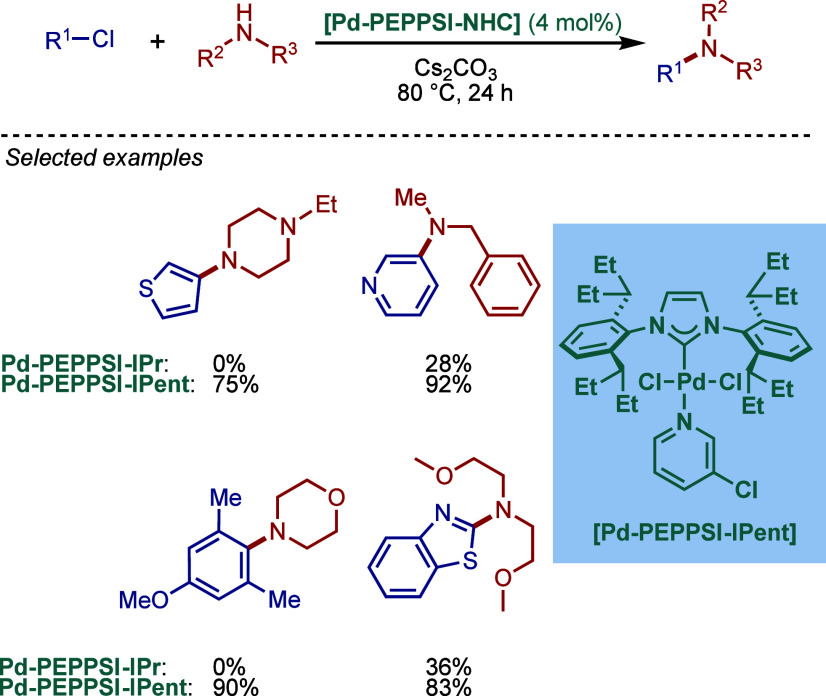
BHA Reaction using Sterically-Demanding [Pd–PEPPSI–IPent] by Organ

**Figure 4:**
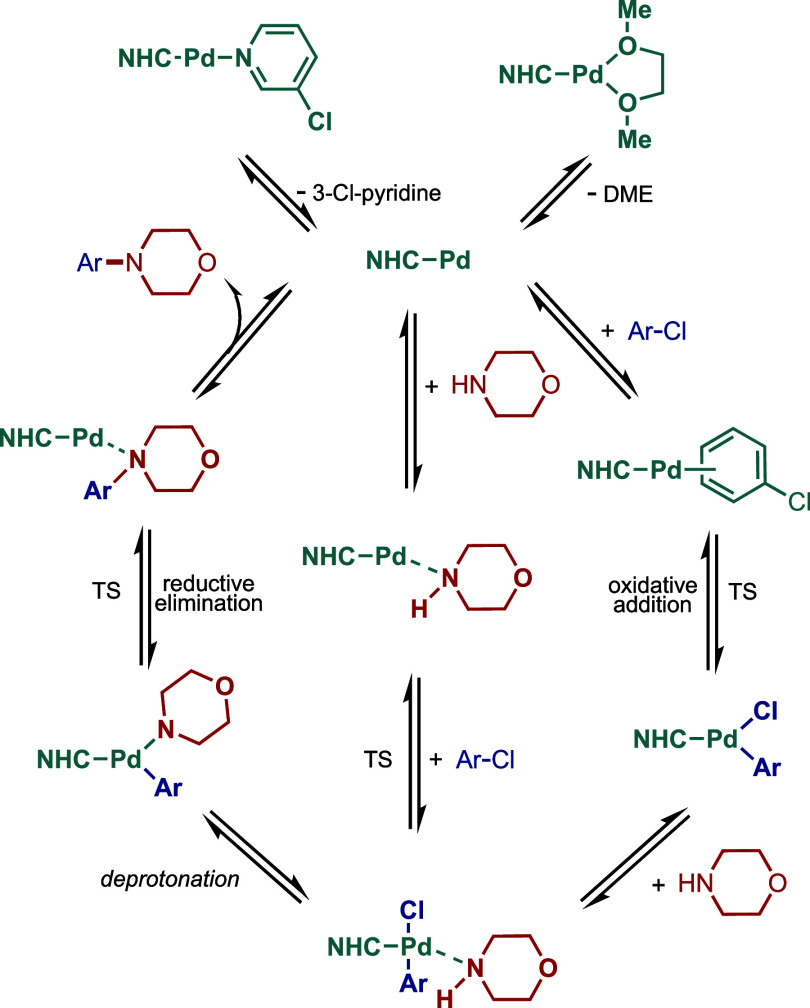
Proposed catalytic cycle for BHA reaction using Pd–PEPPSI–NHC complexes.

In 2011, Tu and co-workers, inspired by the fact that stronger σ-donation and π-accepting properties of π-extended acenaphthoimidazolylidene scaffold increase the electron density of the metal center, reported Pd–PEPPSI–BIAN–IPr (note that BIAN–IPr is also referred to as IPr^An^) for BHA reaction of aryl chlorides (Scheme 63).^202^ In general, these BIAN–NHC complexes show higher reactivity than their imidazol-2-ylidene congeners, which is due to the combination of electronic and steric properties of the scaffold (see Section 2.3.1 as well as Schemes 83, 84, and 98). In contrast to the imidazol-2-ylidene analog,^203^ the catalytic space around the metal center in BIAN–NHC complexes is more hindered with N-aryl wingtips almost perpendicular to the acenaphtylene fragment. The length of the Pd–C bond was determined as 1.960 Å, which is shorter than in the corresponding Pd–PEPPSI–IPr (1.969 Å) complex due to the stronger σ-donor character. In the optimization studies, the highest yield was observed for the reaction carried out in the presence of KO^*t*^Bu in dioxane at 80 °C with only 0.075 mol % catalyst loading. The BHA reaction of aryl chlorides with 2° amines resulted in excellent yields of the cross-coupling products. In the case of 1° amines, a higher catalyst loading (0.5 mol %) was necessary to obtain full conversions. This [Pd­(BIAN–IPr)­(3-Cl-py)­Cl_2_] catalyst is quite general and allows the use of substrates with different electronic character and steric hindrance (see also Section 2.3.2 for another example of high reactivity of BIAN–IPr in BHA reaction). Furthermore, the authors performed catalyst poisoning studies, which clearly indicated a homogeneous reaction mechanism. The utility of this method was presented in the successful synthesis of drug intermediates, such as the antibiotic Linezolid and the nonsteroidal anti-inflammatory drug Mefenamic acid. Another example of the use of [Pd**–**PEPPSI**–**IPr^An^ **–**py] as hyper-cross-linked polymer (HCP) catalysts for BHA reaction was reported by Gao and Tan.^204^

**Scheme 63:**
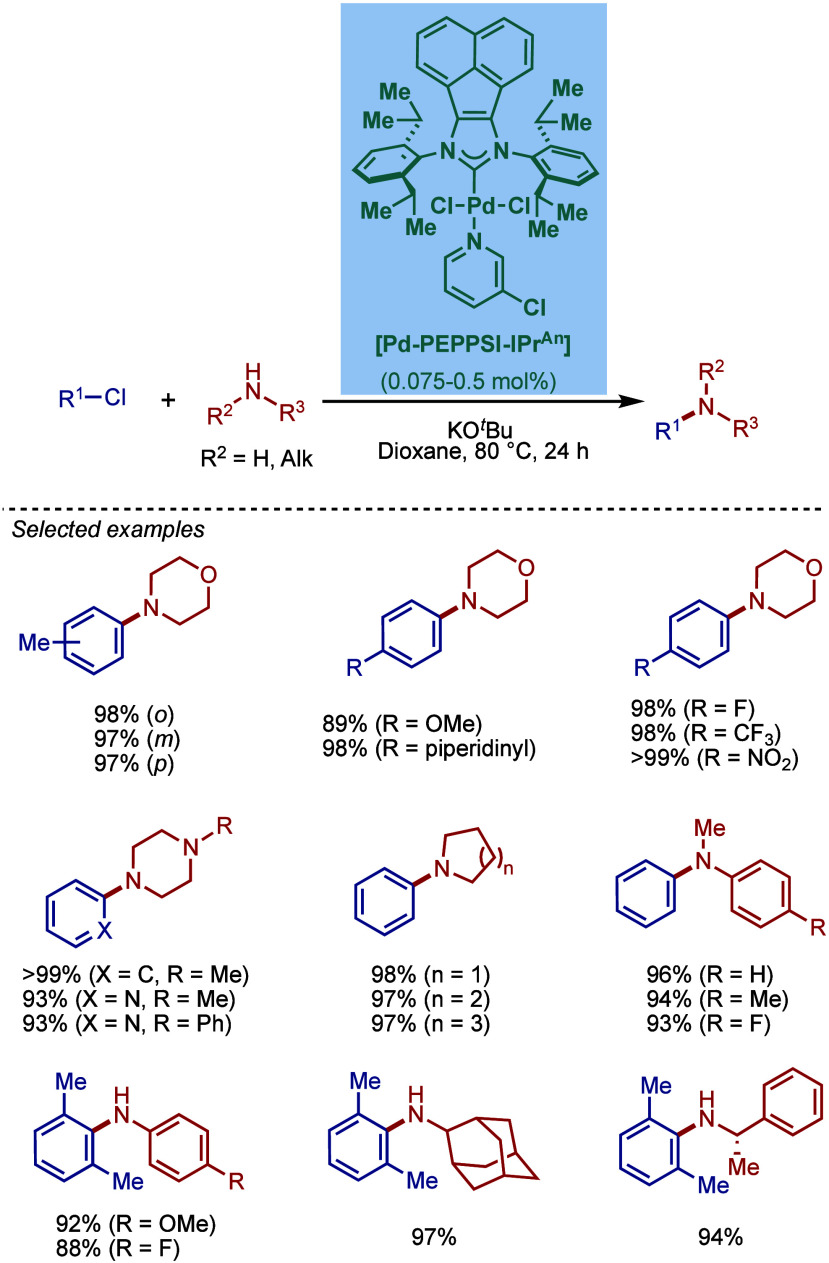
BHA Reaction Catalyzed by [Pd–PEPPSI–BIAN–IPr] by Tu

In 2012, Organ and co-workers reported another study on the high catalytic activity of [Pd–PEPPSI–IPent] in BHA reaction (Scheme 64).^205^ In order to design an improved cross-coupling process, the authors investigated the correlation of the electronic properties of the amine and the palladium center of the Pd–NHC complexes. The effect of the electronic character of the substituent located at the *para*-position of the aromatic ring of the electrophilic oxidative addition partner was also tested. The authors observed that [Pd–PEPPSI–IPr] was ineffective when electron-donating substituents were present in the aryl chloride, whereas [Pd–PEPPSI–IPent] proved to be extremely effective in these cases, providing full conversions using Cs_2_CO_3_ as a mild base. Furthermore, the use of anilines instead of morpholine allowed for an investigation of the relationship between the substituents and initial rates by kinetic studies. The most significant difference in reactivity between [Pd–PEPPSI–IPr] and [Pd–PEPPSI–IPent] was observed when anilines with strongly electron-withdrawing substituents were used. In these cases, the IPr-based catalyst was ineffective, and no reaction progress was observed, while the IPent-based catalyst led to the corresponding products in good to excellent yields. Here again, the use of Cs_2_CO_3_ is noted as is the high catalyst loading.

**Scheme 64:**
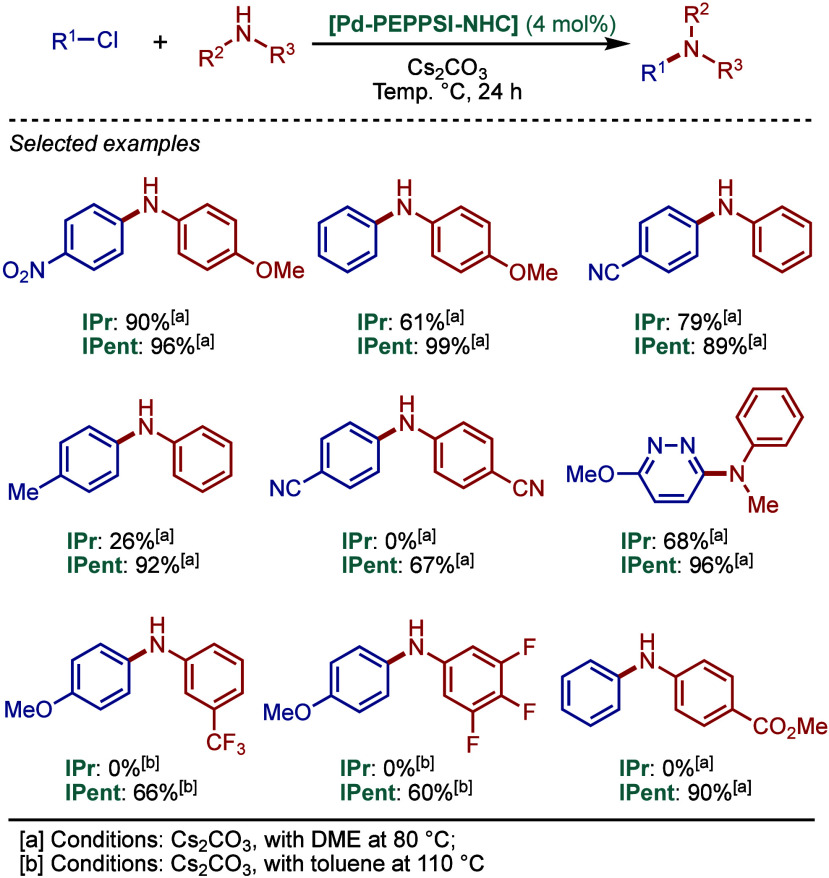
BHA Reaction Catalyzed by [Pd–PEPPSI–IPr] and [Pd–PEPPSI–IPent] by Organ

[a] Conditions: Cs_2_CO_3_, with DME at 80 °C;
[b] Conditions: Cs_2_CO_3_, with toluene at 110 °C

In 2012, inspired by the fact that potassium *tert*-butoxide is characterized by a comparatively low functional group tolerance, Organ and co-workers reported a study on using other bases for the BHA reaction using [Pd–PEPPSI–IPent] (Scheme 65).^206^ After a comprehensive evaluation of bases, such as DBU (1,8-diazabicyclo[5.4.0]­undec-7-ene), potassium trimethylsilanoate, and different salts of 2,2,5,7,8-pentamethyl-6-chromanol, they found that potassium 2,2,5,7,8-pentamethyl-6-chromanoxide was a most effective base. They hypothesized that this was due to its basicity (p*K*_a_ 11.4) that ensures both effective deprotonation of the intermediate aryl–palladium–ammonium complexes and lower nucleophilicity, preventing undesirable side reactions. [Pd–PEPPSI–IPent] showed excellent compatibility with this base, affording the cross-coupling products in high yields. The significant functional group tolerance for sensitive groups, such as esters, ketones, nitriles, and carbamates, is worth noting.

**Scheme 65:**
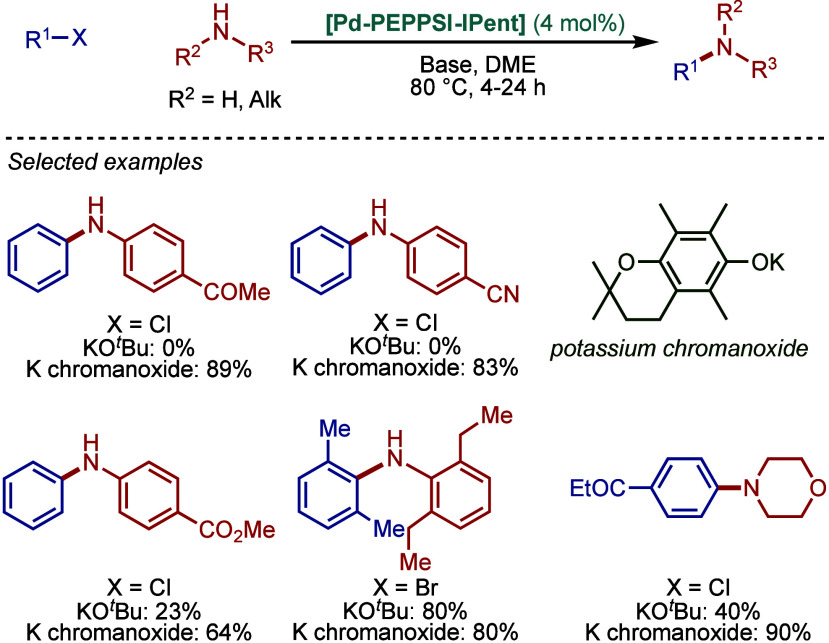
BHA Reaction of Base-Sensitive Substrates Catalyzed by [Pd–PEPPSI–IPent] Using Potassium Chromanoxide by Organ

In 2012, Nolan and co-workers reported a new, well-defined [Pd–PEPPSI–IPr*] complex (IPr* = 1,3-bis­(2,6-bis­(diphenylmethyl)-4-methylphenyl)­imidazo-2-ylidene) and evaluated its catalytic activity in BHA reaction (Scheme 66).^207^ This sterically hindered, air- and moisture-stable precatalyst was obtained by direct metalation of the imidazolium salt using K_2_CO_3_ in 3-Cl-py in 85% yield.^81^ [Pd–PEPPSI–IPr*] is characterized by a very significant steric hindrance; %V_*bur*_ of 43.1% compared to other [Pd–PEPPSI–NHC] congeners (e.g., NHC = IPr, %V_*bur*_ = 34.3%; IPent, %V_*bur*_ = 37.9%; SIPr, %V_*bur*_ = 39.3%). In the model BHA reaction of 4-chlorotoluene with morpholine carried out at room temperature at 1 mol % loading of [Pd­(IPr*)­(3-Cl-py)­Cl_2_], the reactivity was found to be similar to other complexes (NHC = IPr, SIPr). However, at high temperature (110 °C) in the presence of 0.025 mol % of the precatalyst, [Pd­(IPr*)­(3-Cl-py)­Cl_2_] significantly outperformed IPr and SIPr congeners. In the scope evaluation, this [Pd­(IPr*)­(3-Cl-py)­Cl_2_] complex showed similar efficiency to the previously described [Pd­(IPr*)­(cin)­Cl] complex (see Section 2.3.1),^48^ which may indicate the participation of a similar monoligated Pd(0)–NHC species. In 2023, a related well-defined, sterically hindered [Pd­(IPr^#^)­(3-Cl-py)­Cl_2_] was reported and tested in BHA reaction (see Scheme 98).

**Scheme 66:**
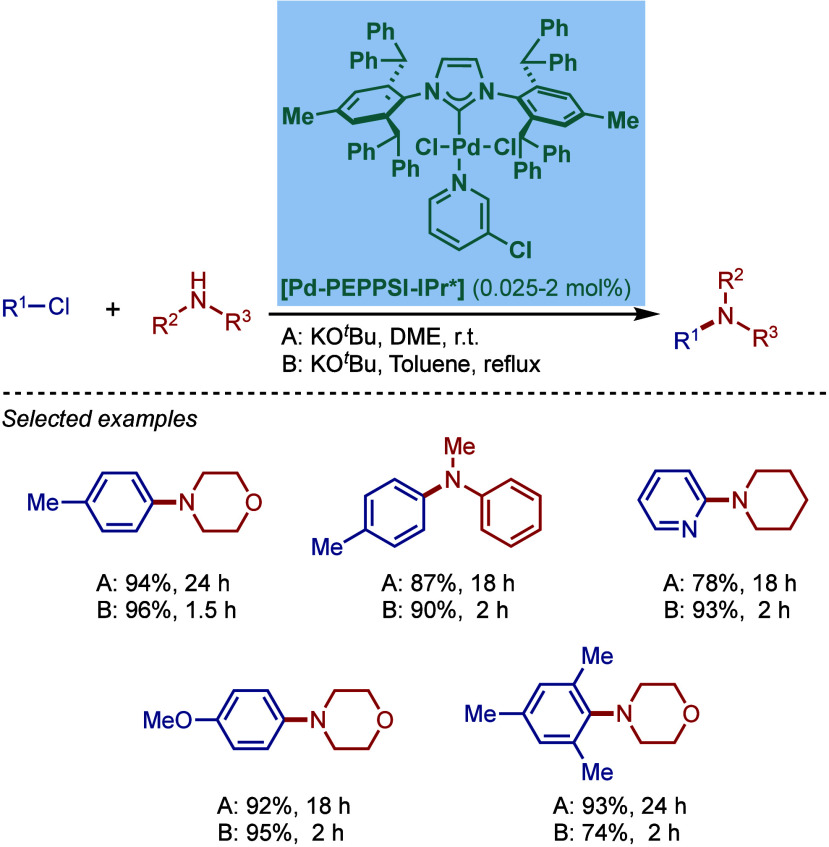
BHA Reaction Catalyzed by [Pd­(IPr*)­(3-Cl-py)­Cl_2_] by Nolan

In 2013, Organ and co-workers reported a highly efficient approach to the synthesis of challenging 3° arylamines using [Pd–PEPPSI–IPent^Cl^] (Scheme 67).^208^ This precatalyst contains electron-withdrawing chlorine substituents on the NHC backbone that also exert a steric buttressing effect on the N-aryl wingtips, and previously showed excellent selectivity in the Negishi cross-coupling of 2° organozinc reagents.^209^ The authors found that [Pd–PEPPSI–IPent^Cl^] was highly effective in the cross-coupling of aryl chlorides with mono- and disubstituted aniline derivatives. Under relatively mild conditions using KO^*t*^Bu in DME at 80 °C, a series of triarylamines featuring sensitive functional groups was obtained in high yields. The utility of this method was further highlighted in the synthesis of triarylamines containing three different aromatic substituents.

**Scheme 67:**
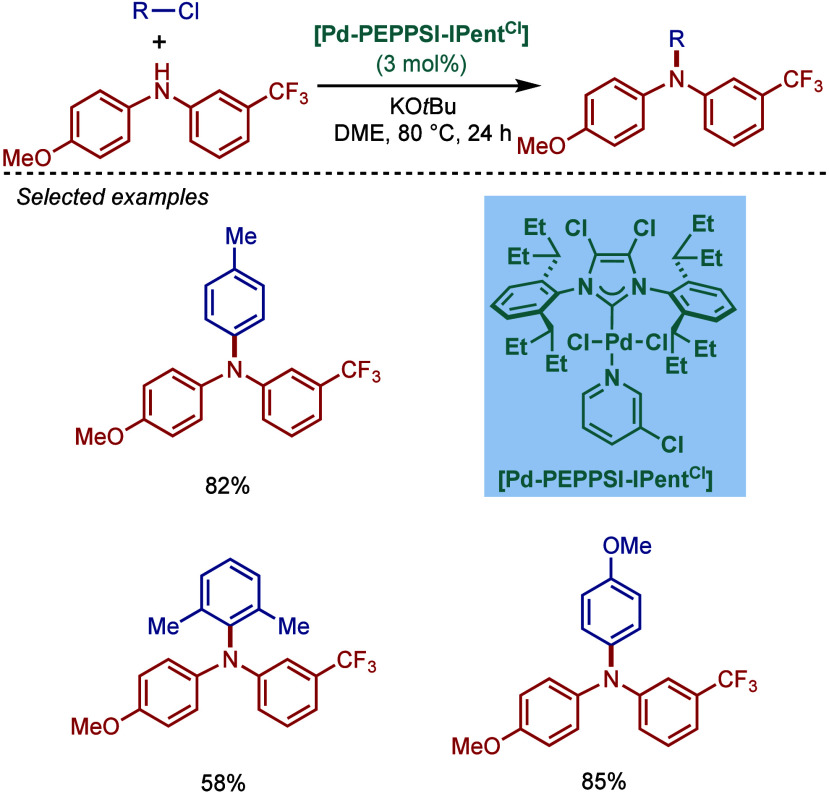
Synthesis of Functionalized Triarylamines Using [Pd–PEPPSI–IPent^Cl^] by Organ

In 2013, Tu and co-workers reported a series of unsymmetrical benzimidazol-2-ylidene based Pd–PEPPSI complexes for the BHA reaction of aryl and heteroaryl chlorides (Scheme 68).^210^ These complexes are characterized by a lower steric hindrance around the palladium center compared to their imidazol-2-ylidene and acenaphthoimidazol-2-ylidene analogues. The methylene linker in the aryl wingtip increases the flexibility of these catalysts, which facilitates substrate binding and increases the overall activity. The X-ray studies demonstrated that the Pd–C bond (1.972 Å) is longer than in the analogous Pd–BIAN–IPr (1.960 Å), which indicates weaker σ-donating properties engendered by the NHC scaffold. The authors compared the catalytic activity of these wingtip-flexible catalysts with the previously described [Pd­(IPr)­(allyl)­Cl], [Pd­(IPr)­(3-Cl-py)­Cl_2_], and Buchwald’s XPhos system, which were all less reactive in the model study. Note here the lack of comparison with state-of-the-art [Pd­(IPr)­(cinnamyl)­Cl]. The most effective catalyst in the series was the Dipp/CH_2_ *i*-Pr^bimy^ derivative, which enabled the synthesis of products using various 2° and 1° aliphatic amines and anilines. It is worth noting that this wingtip-flexibility concept was recently applied to the synthesis of unsymmetrical imidazol-2-ylidene complexes, which showed high reactivity in Cu-catalyzed borylations.^83,211^

**Scheme 68:**
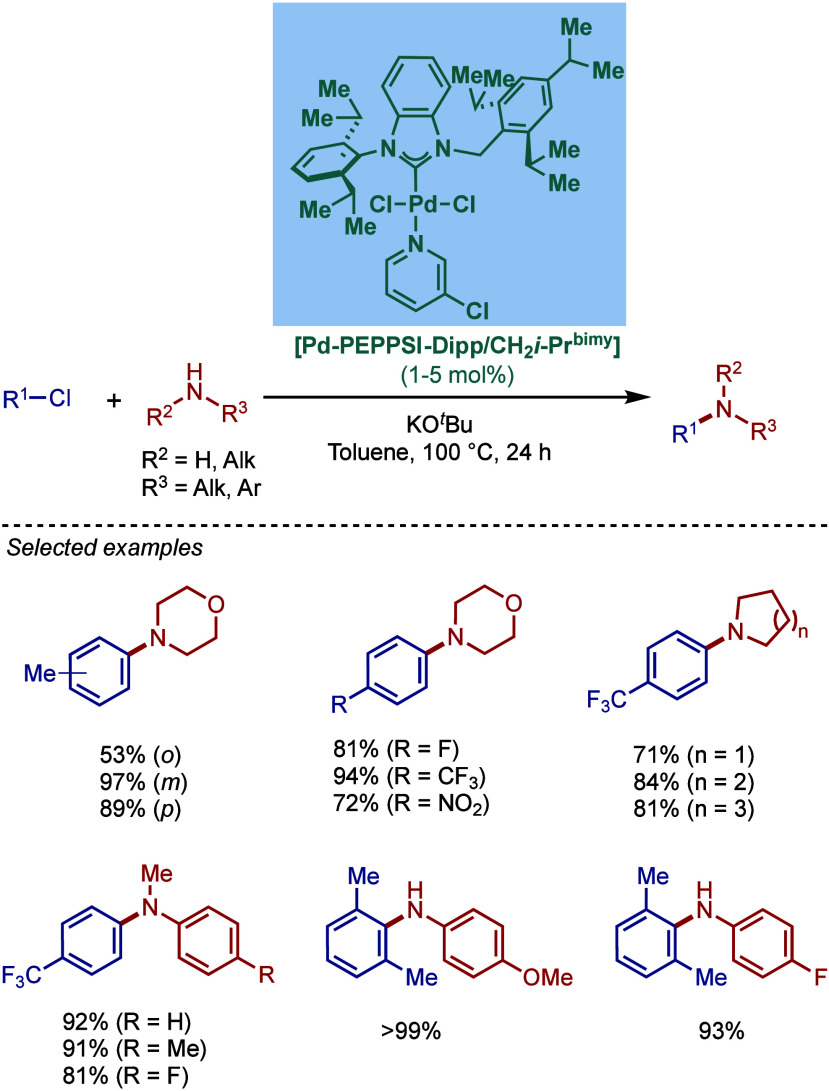
BHA Reaction Catalyzed by Unsymmetrical Benzimidazol-2-ylidene–PEPPSI Complexes by Tu

In 2013, Osuka and co-workers reported the high efficiency of [Pd–PEPPSI–IPr] and [Pd–PEPPSI–IPent] in BHA reaction of bromoporphyrins and haloanthracenes (Scheme 69).^212^ Under the optimized reaction conditions (NaO^*t*^Bu, dioxane, 100 °C), the authors evaluated different catalytic systems, such as [Pd–PEPPSI–NHC] (NHC = IPr, IMes, IPent, SIPr), [PdCl_2_(dppf)], and [Pd_2_(dba)_3_]/trialkyl- and biarylphosphines. The best overall results were observed with [Pd–PEPPSI–IPent]; however, it is worth noting that in some cases [Pd–PEPPSI–IPr] was the more effective catalyst. No comparison was again performed with the [Pd­(IPr)­(cinnamyl)­Cl] system.

**Scheme 69:**
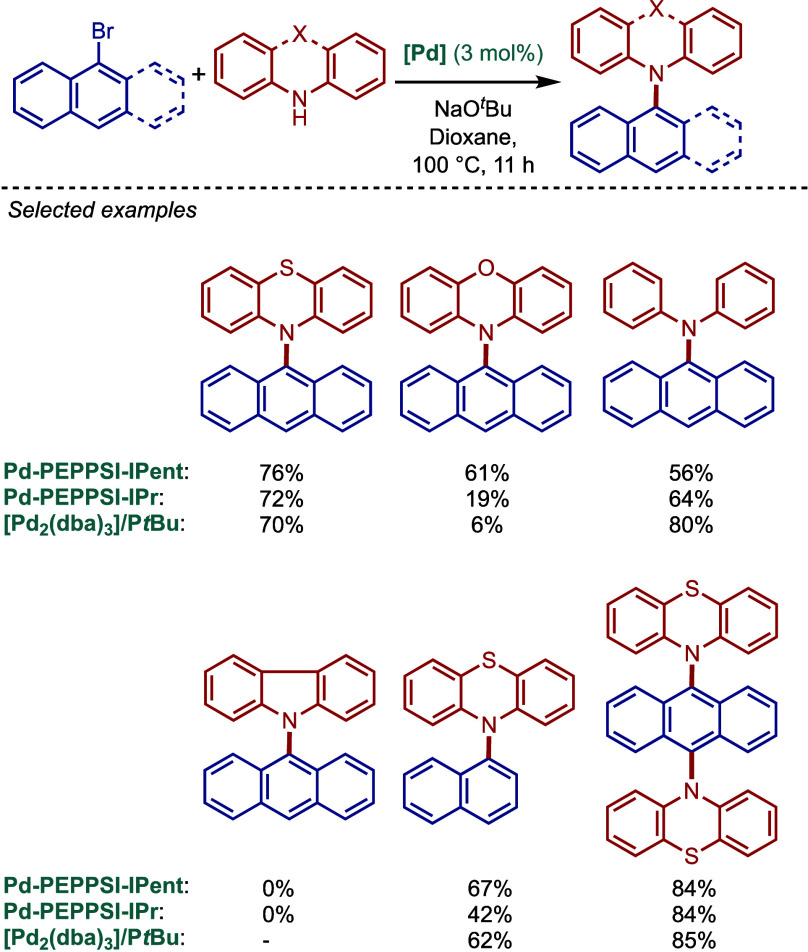
BHA Reaction of 9-Haloanthracenes Using [Pd–PEPPSI–IPr] and [Pd–PEPPSI–IPent] by Osuka

In 2014, Claver and co-workers reported the synthesis of a hydrophobic Pd–PEPPSI catalyst, [Pd­(IPr^OAlk^)­(3-Cl-py)­Cl_2_], and tested its catalytic activity in BHA reaction of aryl halides (Scheme 70).^213^ Under the optimized reaction conditions (KO^*t*^Bu, THF, 30 °C), this precatalyst provided a 98% yield of the cross-coupling product after 30 min, while using the same conditions, [Pd–PEPPSI–IPr] gave only 14% yield of the product. Furthermore, [Pd­(IPr^OAlk^)­(3-Cl-py)­Cl_2_] resulted in the complete conversion at 60 °C after only 5 min. This new catalyst was also tested in the cross-coupling of *ortho*-substituted aryl chlorides with different amines. In most cases, very good yields were obtained, attesting to the beneficial effect of the 4-alkylalkoxy substitution of the IPr scaffold on the reactivity in BHA reaction.

**Scheme 70:**
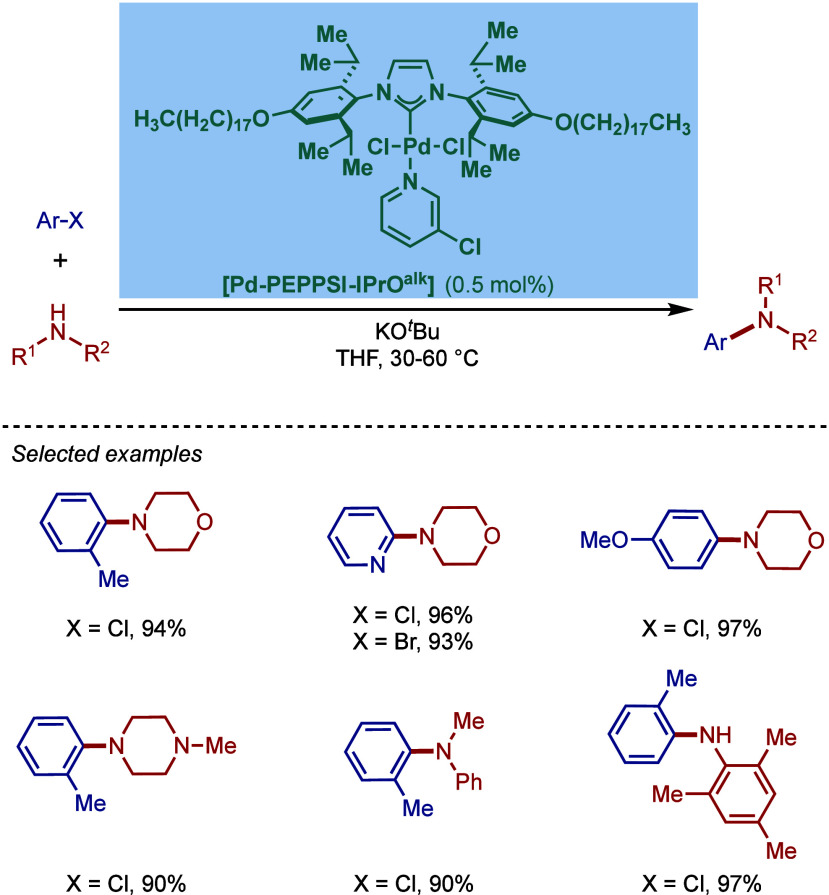
BHA Reaction Catalyzed by [Pd–PEPPSI–IPr^OAlk^] by Claver

In 2014, the Organ group reported the BHA reaction of aryl halides with deactivated amines catalyzed by [Pd–PEPPSI–IPent^Cl^-*o*-picoline] (Scheme 71).^214^ Considering that modification of the NHC backbone previously had a significant influence on the reactivity in BHA reactions, the authors tested various [Pd–PEPPSI–NHC] complexes. They found that in the case of deactivated oxidative addition partners, [Pd–PEPPSI–IPr] showed no activity. However, more promising results were obtained with more electron-deficient NHC scaffolds, such as [Pd–PEPPSI–IPr^Cl^] and [Pd–PEPPSI–IPr^NQ^]. Further comparison of the IPent complex with its chlorinated IPent^Cl^ analogue in the amination of polyfluorinated amines showed that [Pd–PEPPSI–IPent^Cl^] outperformed its IPent congener. This reaction represented the first example of using such strongly deactivated aniline derivatives in BHA reaction. The effect of modifications of the ancillary pyridine ligand was also investigated using the amination of 4-chloroanisole with 3,4,5-trifluoroaniline as a model reaction. The authors found that the best result was observed using [Pd–PEPPSI–IPent^Cl^-*o*-picoline], which afforded the desired product in 82% yield using Cs_2_CO_3_ as a base. This amination is characterized by a very wide scope, including products containing various functional groups such as esters, amides, ketones, borates, and alcohols.

**Scheme 71:**
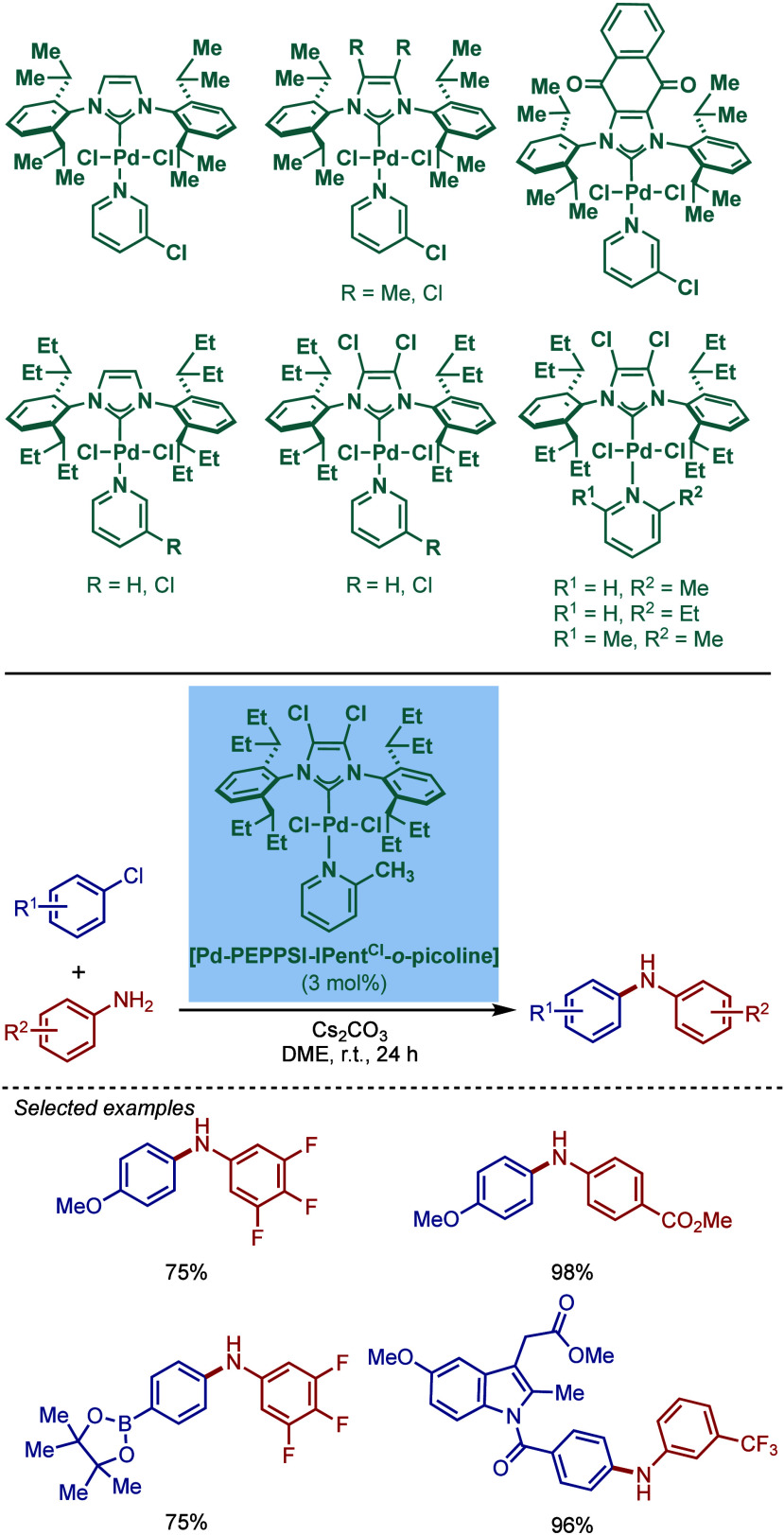
BHA Reaction of Deactivated Anilines using [Pd–PEPPSI–IPent^Cl^-*o*-picoline] by Organ

In 2014, César and Lavigne reported an interesting functionalization of the IMes and IPr imidazol-2-ylidene scaffold with one or two amino groups at the NHC backbone and tested its activity in BHA reaction (Scheme 72, see also Scheme 91,Section 2.3.1)^215^ The incorporation of the dimethyl amino group led to an increase in %V_*bur*_ ([Pd–PEPPSI–IPr^NMe2^], %V_*bur*_ = 39.5%; [Pd–PEPPSI–IPr^(NMe2)2^], 39.9%) compared to [Pd–PEPPSI–IPr] (%V_*bur*_ = 34.3%). Furthermore, the synthesis of [Rh­(NHC)­(CO)_2_Cl] complexes allowed the determinatiuon of the Tolman electronic parameter (TEP), which showed that incorporation of the NMe_2_ group increases the electron-donating ability of the carbene ligand (IMes^NMe2^, TEP = 2048.6 cm^–1^; IMes^(NMe2)2^, TEP = 2046.6 cm^–1^ vs IMes, TEP = 2050.8 cm^–1^). The high reactivity of these new catalysts was demonstrated in the BHA reaction of 4-chloroanisole with morpholine using KO^*t*^Bu in DMF at room temperature. The [Pd–PEPPSI–IPr^(NMe2)2^] complex containing two amino groups proved to be the most efficient and complete conversion was observed only after 2 h with 2 mol % loading, while for the monoamino substituted complex, [Pd–PEPPSI–IPr^NMe2^], and the parent [Pd–PEPPSI–IPr] complex, conversions were 57% and 39% after 6 h, respectively. This highly efficient catalytic system was applied to BHA reactions of a broad range of aryl chlorides and amines using only 0.005–0.1 mol % of precatalyst at 80 °C. Furthermore, remarkable turnover numbers were observed (TON up to 19,600), making this complex one of the most active catalysts in BHA reaction reported to date.

**Scheme 72:**
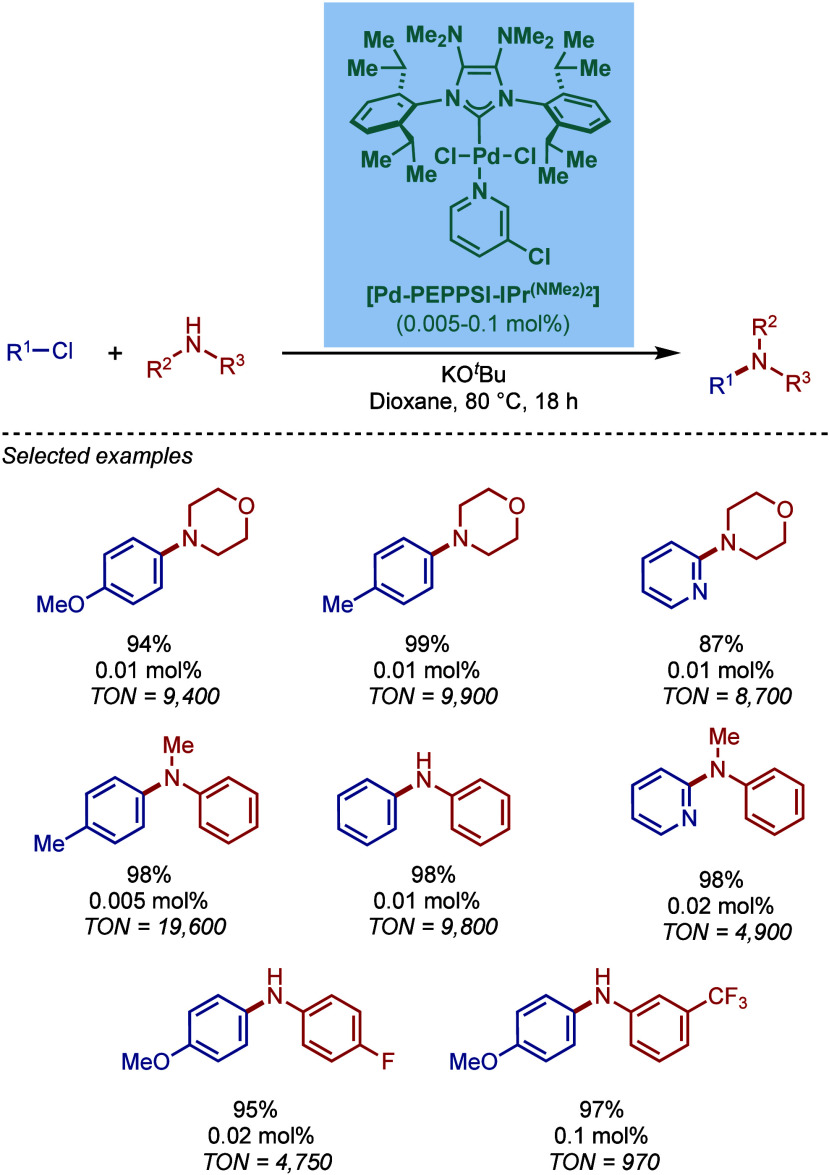
BHA Reaction Using [Pd–PEPPSI–IPr^(NMe2)2^] by César and Lavigne

In a continuation of their earlier studies, in 2015, César and Lavigne investigated the possibility of using [Pd–PEPPSI–IPr^NMe2^] and [Pd–PEPPSI–IPr^(NMe2)2^] complexes for BHA reaction in the presence of mild carbonate bases (Scheme 73).^216^ They identified conditions using [Pd–PEPPSI–IPr^(NMe2)2^] and Cs_2_CO_3_ in DME at 80 °C, which led to full conversion at 1 mol % after 24 h. The corresponding monoamino complex, [Pd–PEPPSI–IPr^NMe2^], and the parent [Pd–PEPPSI–IPr] complex were less effective, affording 75% and 15%, respectively. Furthermore, a comparison of the cross-coupling of electron-rich aryl chlorides and electron-poor anilines, allowed to determine the relative reactivity of [Pd–PEPPSI–NHC] complexes in the following order: IPent^Cl^ > IPr^(NMe2)2^ ≈ IPent ≫ IPr. This method was applied to the BHA reaction of 1° alkyl amines, which represent challenging substrates since Pd complexes can undergo β-hydride elimination in these cases. Using sterically hindered aryl halides, such as 2-chlorotoluene or 2,6-dimethylchlorobenzene, the desired products were obtained in excellent yields (97% and 92%). However, the coupling of deactivated substrates, such as 4-chloroanisole or 3-chloropyridine, resulted in a mixture of mono- and bis-arylated products.

**Scheme 73:**
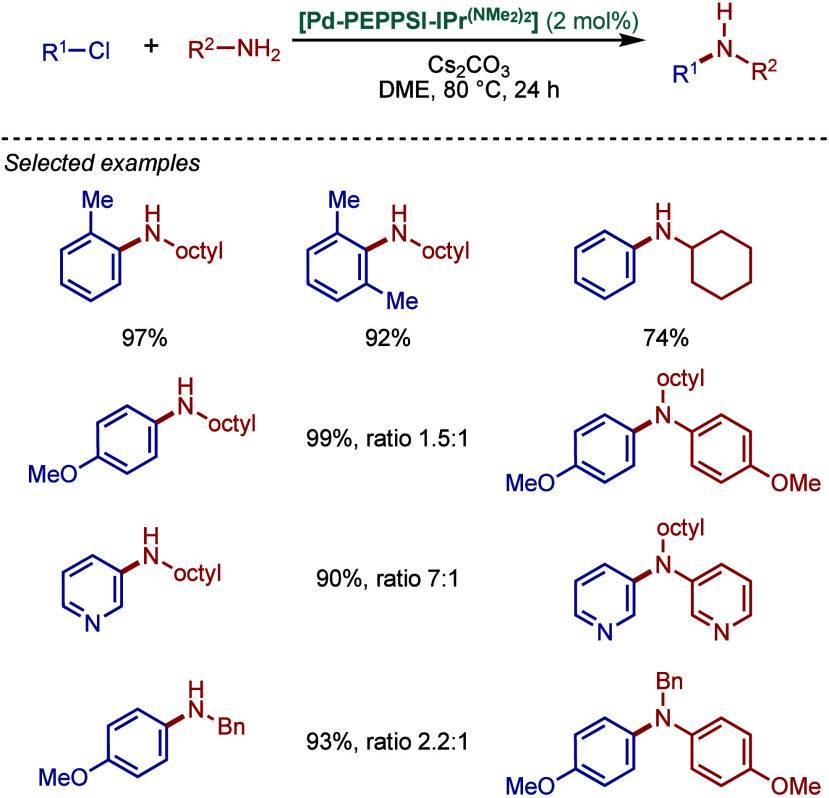
BHA Reaction with 1° Amines Using Carbonate Base Catalyzed by [Pd–PEPPSI–IPr^(NMe2)2^] by César and Lavigne

In 2015, Organ and co-workers reported a method for selective monoarylation of 1° amines using a combination of [Pd–PEPPSI–IPent^Cl^] precatalyst in the presence of sodium salt of butylated hydroxytoluene (NaBHT) (Scheme 74).^217^ This catalytic system enabled the use of a wide range of electrophiles featuring methoxy, carbonyl, cyano, trifluoromethyl and nitro functional groups, which highlighted the broad functional group tolerance of this method. Furthermore, this system resulted also in exceptionally high selectivity for monoarylation. To further extend the scope, the authors tested aryl chlorides containing acidic functional groups, such as carboxylic acids, alcohols, and indoles. These reactions were carried out in the presence of LiHMDS as a base and resulted in high yields and selectivity.

**Scheme 74:**
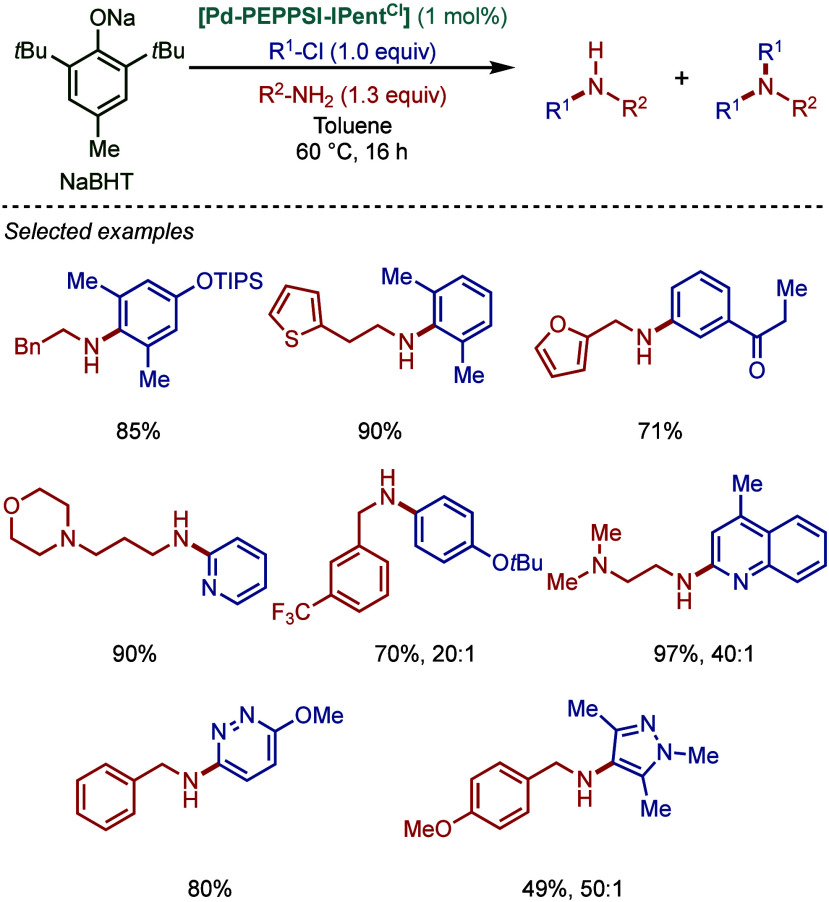
BHA Reaction of 1° Amines Using [Pd–PEPPSI–IPent^Cl^] and NaBHT by Organ

In 2015, Bao and co-workers reported a mechanistic study of the BHA reaction of chlorobenzene with aniline (Scheme 75).^218^ The authors excluded reducing agents, such as solvents and ancillary ligands, that could potentially reduce Pd­(II) to Pd(0). For this purpose, aniline was used as a solvent and Pd­(II)–NHC complexes in which the 3-chloropyridine ligand was replaced by 1-methylimidazole or aniline were tested (see also Sections 2.3.10 and 2.3.11). Catalytic studies showed that the proposed complexes catalyzed the amination reaction, while the best results were obtained for [Pd–PEPPSI–IPr] and its analogue containing aniline ligand. Furthermore, when using PdCl_2,_ only traces of the desired product were observed, which clearly indicated the key role of the NHC ligand. Using computational studies, the authors further investigated other possible BHA reaction mechanisms, such as Pd­(II)-mediated σ-bond metathesis, Pd­(II)/Pd­(IV) cycle, single electron transfer (SET) mechanism, and halide atom transfer (HAT).

**Scheme 75:**
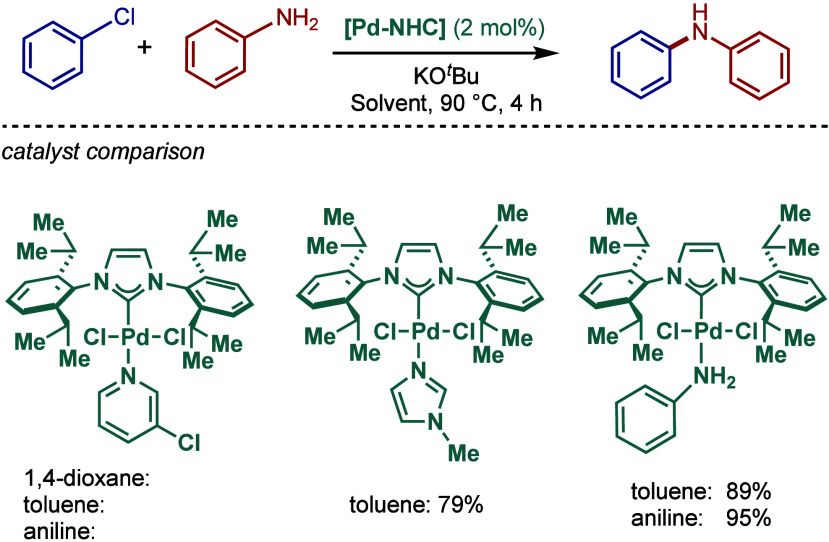
BHA Reaction of Chlorobenzene with Aniline and the Effect of Solvent and Ancillary Ligand by Bao

Another example of the use of [Pd–PEPPSI] complexes was reported by Organ in 2016 in the synthesis of optically chiral α-amino acid esters (Scheme 76).^219^ The authors found that [Pd–PEPPSI–IPent^Cl^-*o*-picoline] enabled efficient BHA reaction of chiral N-arylated amino acid derivatives with heteroaryl chlorides using Cs_2_CO_3_ in DME at 60–80 °C. These reactions are notable not only for their high yields, but also for their excellent stereoretention. The authors showed that partial racemization was a base-mediated process after the product formation. The use of sterically hindered esters resulted in a slower postcoupling racemization process.

**Scheme 76:**
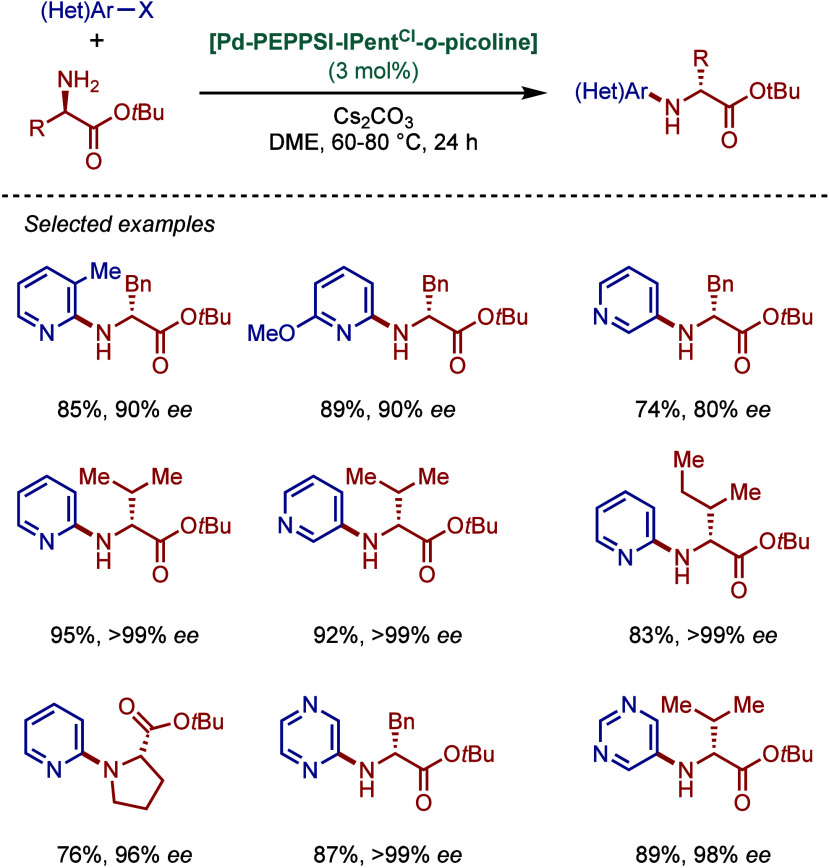
BHA Reaction of Chiral α-Amino Esters by Organ

In 2017, Osipov and co-workers reported the first example of unsymmetrical imidazol-2-ylidene complexes containing *ortho*-fluorinated N,N′-diaryl wingtips and tested their activity in BHA reaction (Scheme 77).^220^ Metal complexes containing fluorine and perfluoroalkyl groups are of significant present interest due to their unique physicochemical properties. These fluorinated [Pd–PEPPSI] complexes could be prepared directly by a one-step reaction of the corresponding NHC salt with [Pd­(3-Cl-py)_2_Cl_2_] in 85–90% yields. Structures of these air- and moisture-stable complexes were confirmed by X-ray analysis and showed that the incorporation of trifluoromethyl groups on the wingtip resulted in shortening of the Pd–C bond (1.954 Å) vs the corresponding [Pd–PEPPSI–IMes] (1.962 Å). The catalytic activity was tested in the BHA reaction of bromobenzene with morpholine at 0.5 mol % catalyst loading. The fluorinated complexes showed better activity than [Pd–PEPPSI–IMes] (59% vs 28% in heptane).

**Scheme 77:**
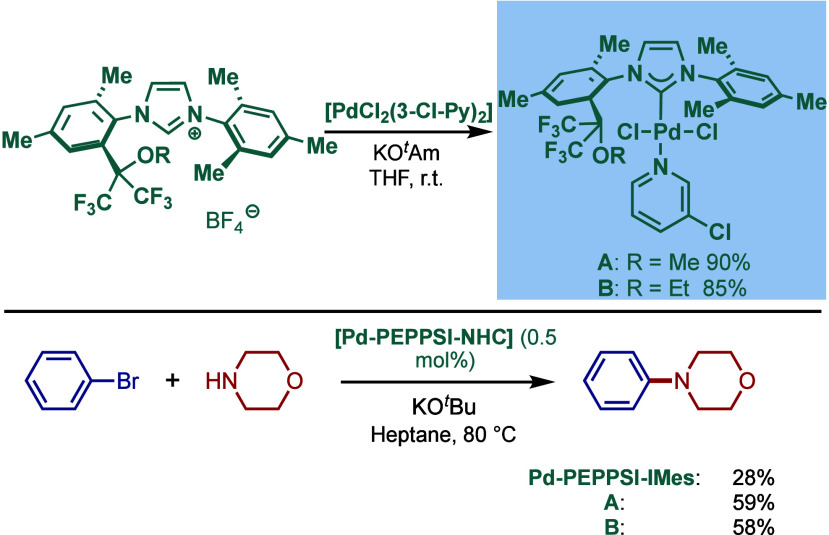
BHA Reaction Using Fluorinated [Pd–PEPPSI–NHC] Complexes by Osipov

In 2017, Bazzi and co-workers reported polyisobutylene (PIB)-supported [Pd–PEPPSI–IMes] complexes and evaluated their catalytic activity in BHA reaction (Scheme 78).^221^ The highest efficiency was observed when the reaction was carried out in lipophilic solvents, such as heptane. Interestingly, the authors also synthesized the corresponding PIB-supported [Pd–PEPPSI–BIAN–IMes] complex, which showed lower catalytic activity. The authors hypothesized that this was due to the excessive steric hindrance resulting from the presence of both the acenaphthyl moiety and the PIB fragment. In this approach, the use of lipophilic complexes facilitates the isolation of coupling products without the need for chromatography. The catalytic activity of this PIB-supported [Pd–PEPPSI–IMes] precatalyst was like that of the IXy analogue (IXy = 2,6-dimethylphenyl).

**Scheme 78:**
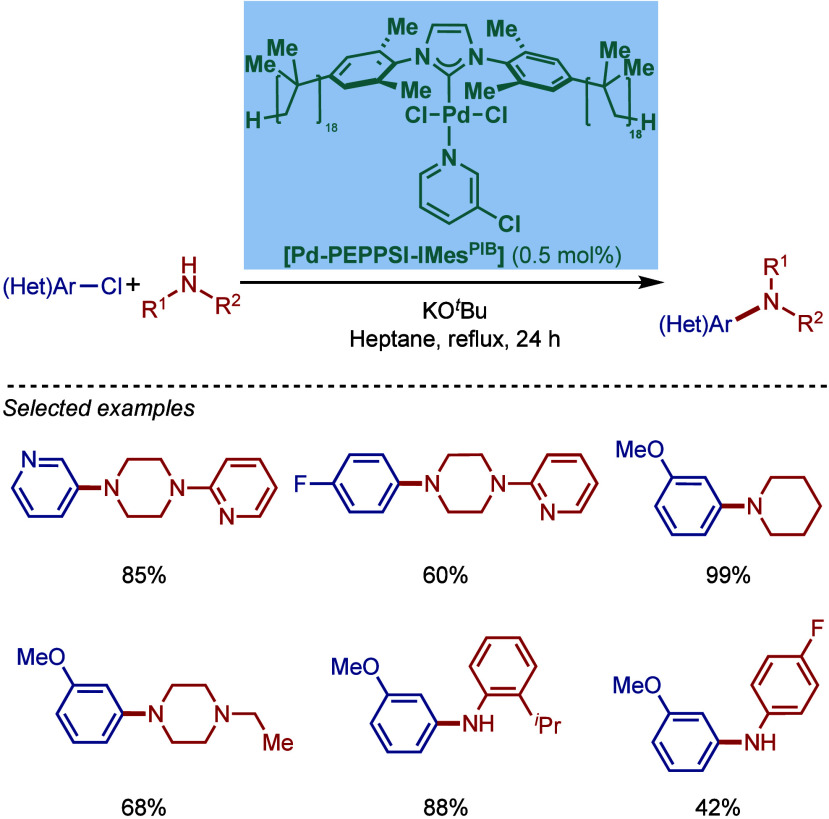
BHA Reaction Catalyzed by PIB-Supported [Pd–PEPPSI–IMes] by Bazzi

In 2017, Organ and co-workers reported BHA reaction of 2-aminopyridines catalyzed by [Pd–PEPPSI–NHC] complexes (Scheme 79).^222^ The coupling of 2-aminopyridines is often problematic due to catalyst deactivation by chelation. Thus, the authors hypothesized that the catalyst activity should correlate with steric hindrance around the Pd center. For this purpose, they tested various sterically demanding Pd–NHC complexes, such as [Pd–PEPPSI–IPent], [Pd–PEPPSI–IHept], and their chlorinated analogues [Pd–PEPPSI–IPent^Cl^] and [Pd–PEPPSI-IHept^Cl^]. They found that the best activity was displayed by the [Pd–PEPPSI–IPent^Cl^] complex. This catalyst was then successfully used in the BHA reaction of 2-aminopyridines with various aryl chlorides.

**Scheme 79:**
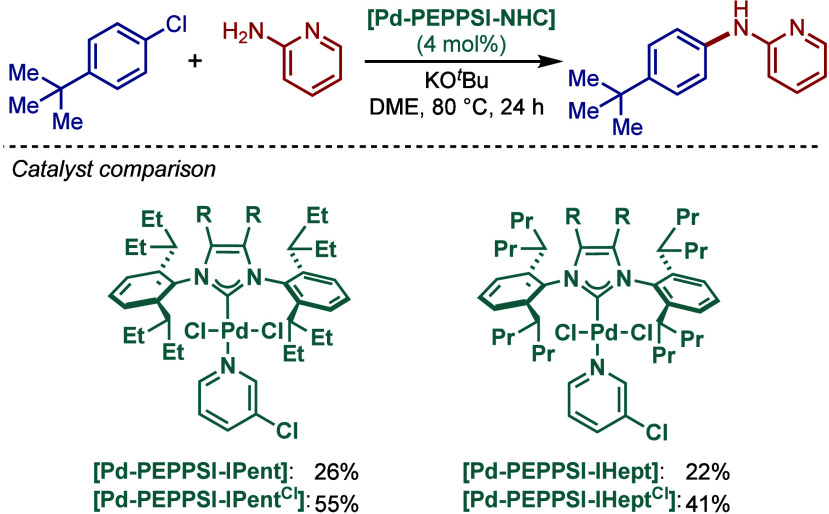
BHA Reaction of 2-Aminopyridines Using [Pd–PEPPSI–NHC] Complexes by Organ

As a further extension of their studies on the use of a [Pd–PEPPSI–IPent^Cl^] precatalyst in BHA reactions, in 2017, Organ and co-workers reported a general protocol for the coupling of unactivated, sterically hindered 1° and 2° amines (Scheme 80).^223^ Similar to their previous study,^104^ they identified sodium butylated hydroxytoluene (NaBHT) as a sterically hindered, yet strong enough base to deprotonate metal alkyl–ammonium complexes. The protocol allows for the BHA reaction of 1° and 2° sterically demanding amines with broad functional group tolerance, including base-sensitive functional groups such as esters, nitriles, and ketones.

**Scheme 80:**
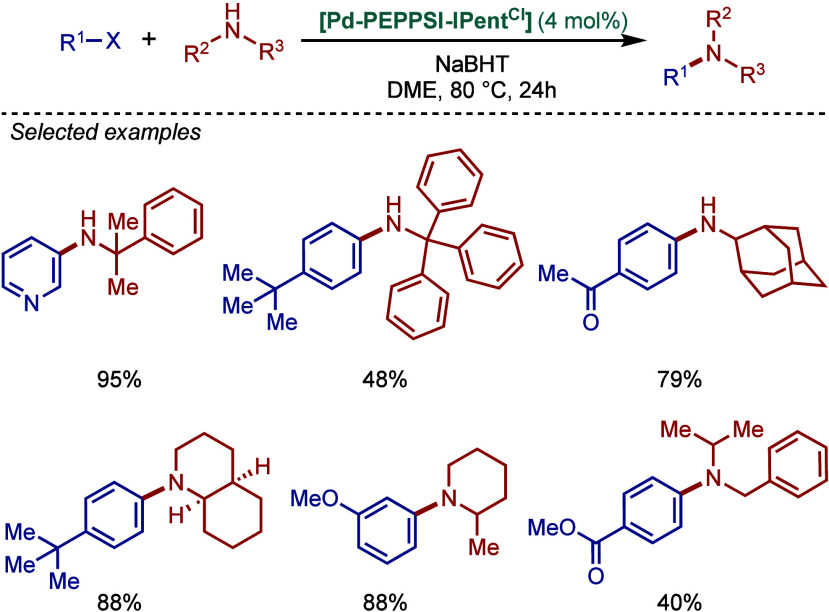
BHA Reaction of Sterically Hindered 1° and 2° Amines Using [Pd–PEPPSI–IPent^Cl^] and NaBHT by Organ

In 2017, Liu and co-workers reported a novel protocol for the BHA reaction of aryl chlorides with amines under aerobic conditions (Scheme 81).^224,225^ This methodology relies on the use of unsymmetrical [Pd–PEPPSI–BIAN–NHC] complexes, featuring flexible steric bulk of the N-aryl wingtip of the acenaphthene scaffold. Extensive studies on the electronic character of these Pd complexes and the effect of pyridine ligands have shown that precatalysts with electron-donating substituents of the aryl wingtips promote the oxidative addition step during cross-coupling process. The authors also tested the parent [Pd–PEPPSI–IPr^An^] and [Pd–PEPPSI–IPr*] complexes; however, they observed much lower reaction conversions of 46% and 92%, respectively. DFT studies established that oxidative addition is the rate-determining step. Increased σ-donating character and flexible steric bulk of unsymmetrical BIAN–NHCs gave a series of products in excellent yields using various aryl and heteroaryl chlorides as well as aromatic and aliphatic amines under highly practical aerobic conditions.

**Scheme 81:**
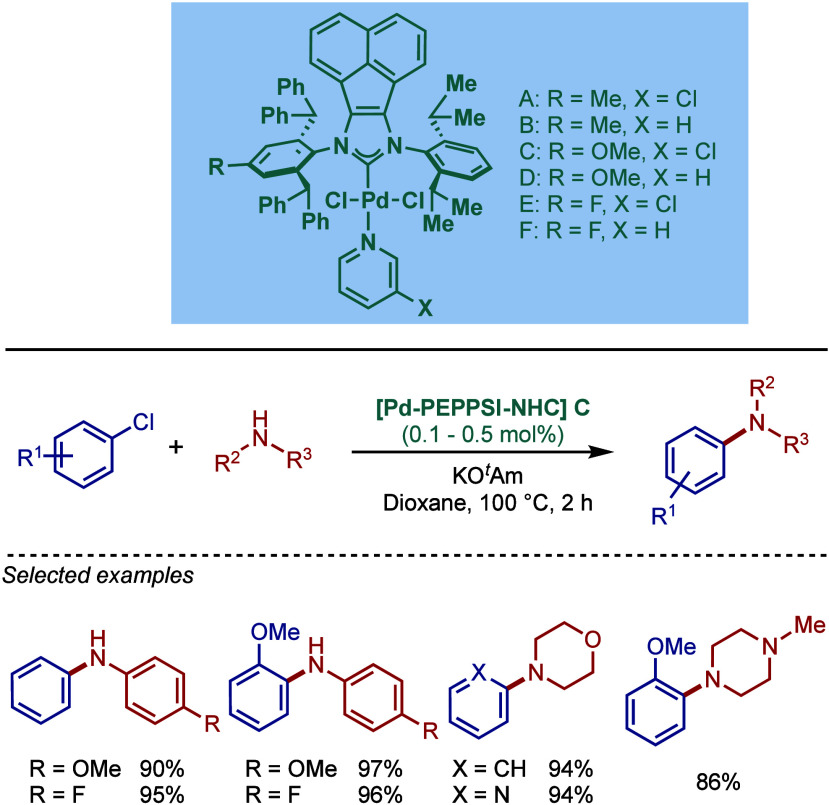
BHA Reaction under Aerobic Conditions Catalyzed by Unsymmetrical [Pd–PEPPSI–BIAN–NHC] Complexes by Liu

In 2017, Richardson and co-workers described an interesting approach for identifying functional group tolerance in BHA reactions using an intermolecular functional group additive (FGA) under different conditions (Scheme 82).^226^ A model reaction was performed using 2-bromonaphtalene and morpholine in the presence of various FGA, such as alkynes, nitriles, esters, thioamides, sulfides, sulfonamides, and heterocycles. Different catalytic systems were tested, such as Pd_2_(dba)_3_/BINAP, [Pd­(cin)­Cl]_2_/*t*-BuXPhos, [Pd­(allyl)­Cl]_2_/AdBippyPhos, BrettPhosG1/RuPhos, and [Pd–PEPPSI–IPent]. In most cases, the IPent catalyst system showed the highest efficiency with little degradation of the functional group additive. Finally, [Pd–PEPPSI–IPent] was selected for the synthesis of a library of products functionalized with amide, sulfonamide, and indole groups.

**Scheme 82:**
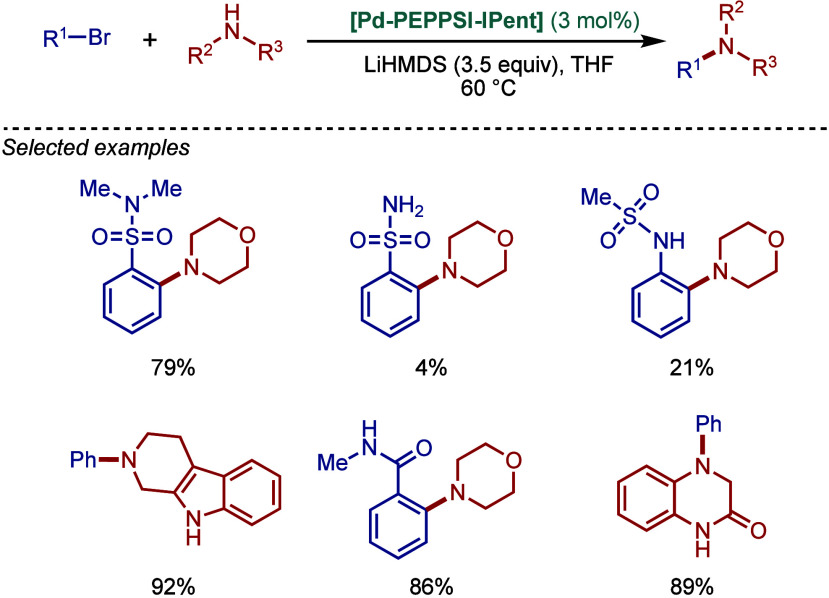
BHA Reaction for the Synthesis of Polyfunctionalized C–N Coupling Products Using [Pd–PEPPSI–IPent] by Richardson

In 2017, César and co-workers reported a study on the effect of backbone substitution of the imidazol-2-ylidene ligands on the catalytic efficiency in the BHA reaction (Scheme 83, see also Scheme 91, Section 2.3.1).^148^ New [Pd–PEPPSI] complexes containing one diisopropylamino C3-substituent or a combination of C3-chloro and C4-dimethylamino groups were synthesized from the corresponding [(IPr^N*i*Pr2^)­Pd­(allyl)­Cl] or [Pd–PEPPSI–IPr^NMe2^] complexes in 72% and 85% yield using HCl/3-Cl-py and NCS, respectively. The complexes were examined by X-ray crystallography to determine the percent buried volume. The %V_*bur*_ for the NHC in [Pd–PEPPSI–IPr^N*i*Pr2^] (%V_*bur*_ = 40.6%) and [Pd–PEPPSI–IPr^NMe2/Cl^] (%V_*bur*_ = 39.8%) were higher than for their counterparts with a single NMe_2_ group (IPr^NMe2^, %V_*bur*_ = 39.7%) and the parent NHC (IPr, %V_*bur*_ = 34.3%). Catalytic activity was tested in BHA reaction of 2- and 4-chloroanisole and morpholine using 0.5 mol % Pd complex. Nearly full conversion was observed after 120 and 30 min at room temperature for the most reactive [Pd–PEPPSI–IPr^NMe2/Cl^] precatalyst. When the more challenging *tert*-butyl amine was use, a significant decrease in reaction conversion was observed, leading to the product only in 30% after 4 h with [Pd–PEPPSI–IPr^NMe2/Cl^]. In this case, quantitative conversion was observed using the allyl-based precatalyst, [Pd­(IPr^NMe2/Cl^) (cin)­Cl] (see Section 2.3.1).

**Scheme 83:**
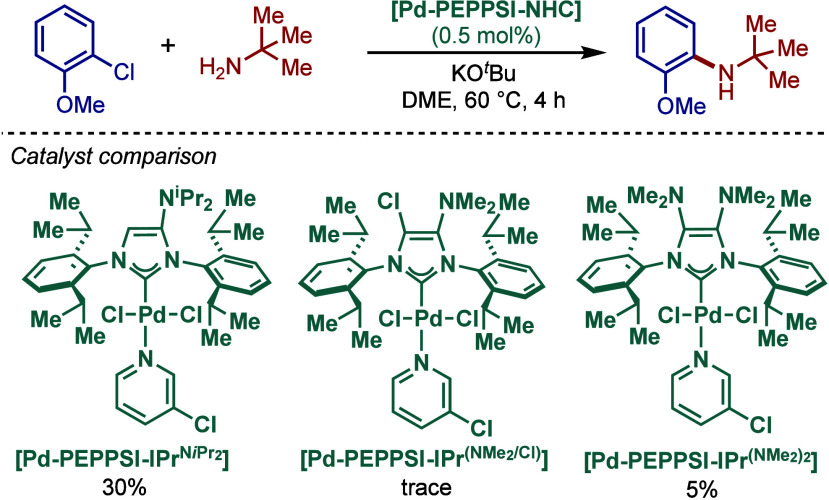
BHA Reaction Using [Pd–PEPPSI–IPr^N*i*Pr2^] and [Pd–PEPPSI–IPr^NMe2/Cl^] Complexes by César

In 2018, Liu and co-workers described a highly efficient method for BHA reaction with deactivated and sterically hindered aryl chlorides and anilines using [Pd–PEPPSI–IPent^An^] complexes (Scheme 84).^227^ They evaluated the catalytic activity of various [Pd–PEPPSI]-type precatalysts, and found that the acenaphthene-based [Pd–PEPPSI–IPent^An^] showed the highest efficiency, well outperforming other catalysts, such as [Pd–PEPPSI–IPr], [Pd–PEPPSI–IPent], and [Pd–PEPPSI–IPr*], as well as unsymmetrical [Pd–PEPPSI–BIAN–NHC] derivatives. Furthermore, the evaluation of the ancillary throw-away ligand showed that the parent pyridine was more effective than the 3-chloro-substituted derivative. Based on the X-ray structures, the percent buried volume of the NHC in Pd–PEPPSI–BIAN–IPent] (%V_*bur*_ = 38.2%) was larger than that of [Pd–PEPPSI–BIAN–IPr] (%V_*bur*_ = 34.7%) and [Pd–PEPPSI–IPr] (%V_*bur*_ = 34.3%). This novel catalyst allowed to obtain a series of valuable amine products, including anti-Parkinson’s drug, Piribedil, from sterically hindered aryl chlorides and deactivated anilines in excellent yields.

**Scheme 84:**
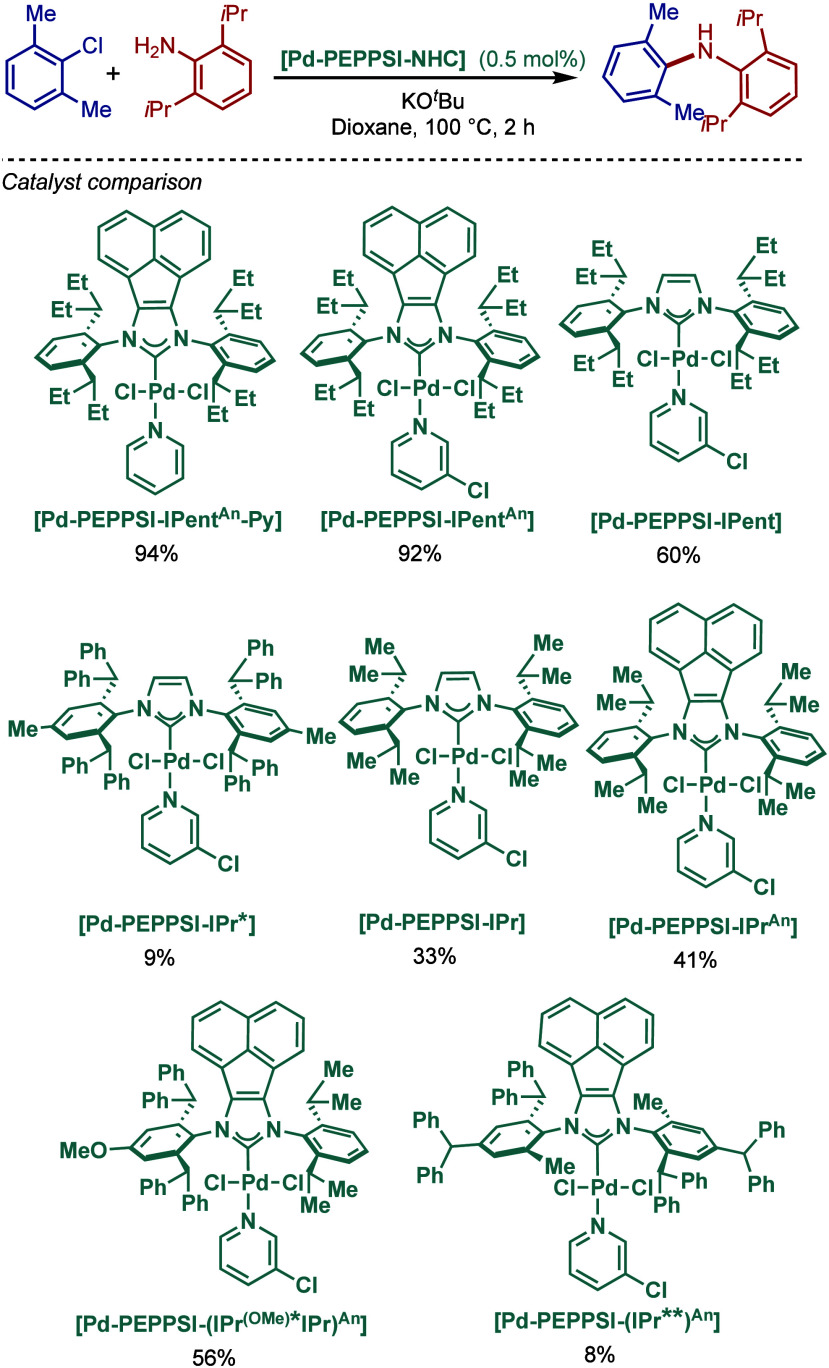
BHA Reaction of Deactivated Aryl Chlorides with Sterically-Hindered Anilines Catalyzed by [Pd–PEPPSI–IPent^An^] by Liu

In 2019, Browne and co-workers reported an interesting mechanochemical approach to BHA reaction catalyzed by [Pd–PEPPSI–NHC] complexes (Scheme 85).^228^ Using chlorobenzene and morpholine as model substrates, the authors evaluated [Pd–PEPPSI–IPent] precatalyst (1 mol %) in the presence of various grinding agents and KO^*t*^Bu as a base. To improve mixing, additives such as Celite, silica gel, sand, and NaCl were tested. Interestingly, the addition of sand (3 equiv) resulted in an improvement in reaction efficiency from 56% to 82%. Furthermore, the authors compared the catalytic activity of [Pd–PEPPSI–NHC] derivatives, such as IPent, IPr, IPr*^OMe^, and IPr^An^. The highest efficiency was observed for [Pd–PEPPSI–IPent], which outperformed other complexes in the order of IPent > IPr > IPr*^MeO^ > IPr^An^ (95% vs 50%, 31%, 23%, respectively). The scope of this method was investigated using aryl- and heteroaryl chlorides in the coupling with 2° acyclic and cyclic amines. The desired products were obtained in high yields after 3 h of ball milling at 30 Hz under aerobic conditions. The developed method was successfully applied to the synthesis of an antidepressant drug, Vortioxetine. Comparison of the reaction carried out in a ball mill with the classical solvent-based coupling (THF, KO^*t*^Bu, air) showed that the mechanochemical approach leads to a significant shortening of the reaction time, improved yields, and slower catalyst deactivation under these conditions.

**Scheme 85:**
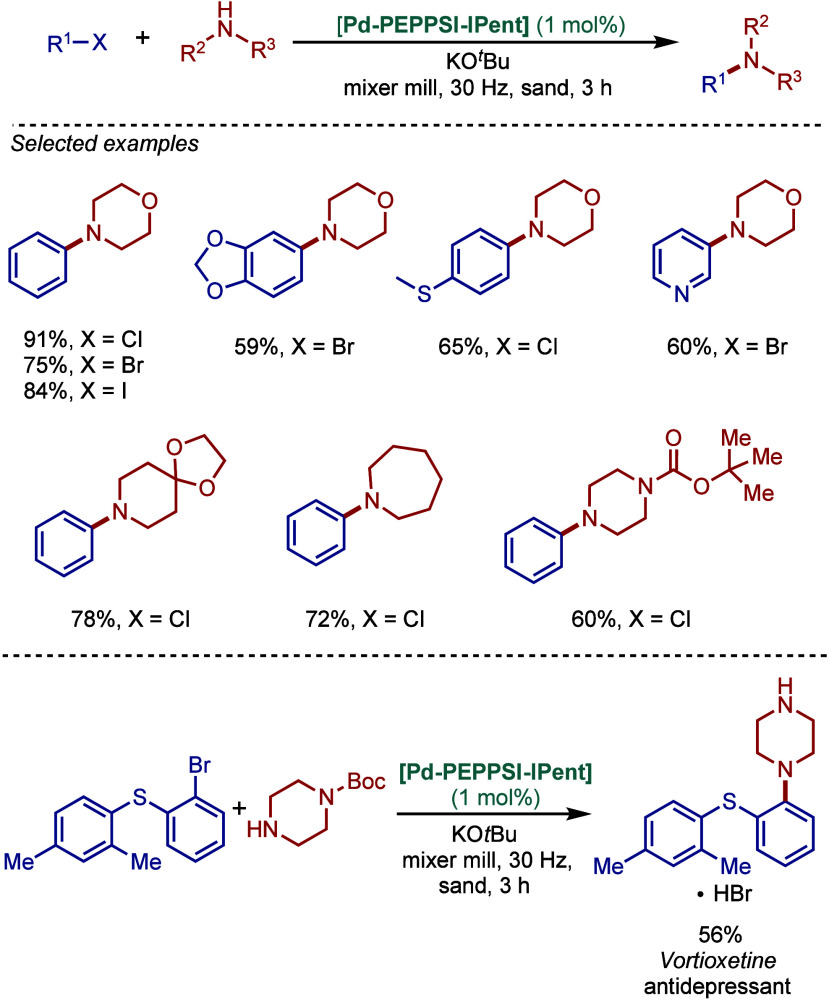
Mechanochemical BHA Reaction Catalyzed by [Pd–PEPPSI–IPent] by Browne

In 2019, Diver and co-workers reported macrocyclic N-heterocyclic carbenes based on imidazol-2-ylidene scaffold and their application in BHA reaction (Scheme 86).^229^ The corresponding imidazolium salt precursor was obtained in a multistep synthesis featuring ring-closing metathesis as the key step. The desired [Pd–PEPPSI–NHC] precatalyst was obtained in 78% yield by the reaction with PdCl_2_ in the presence of K_2_CO_3_ in a mixture of 3-chloro-pyridine and DMSO. Single crystal X-ray analysis showed that the pyridine ligand is *trans*-positioned to the NHC ligand and is located to the side of the macrocyclic cavity. Catalytic activity was tested in the BHA reaction of chlorobenzene with morpholine (KO^*t*^Bu, dioxane, 100 °C), affording the C–N coupling product in 81% yield.

**Scheme 86:**
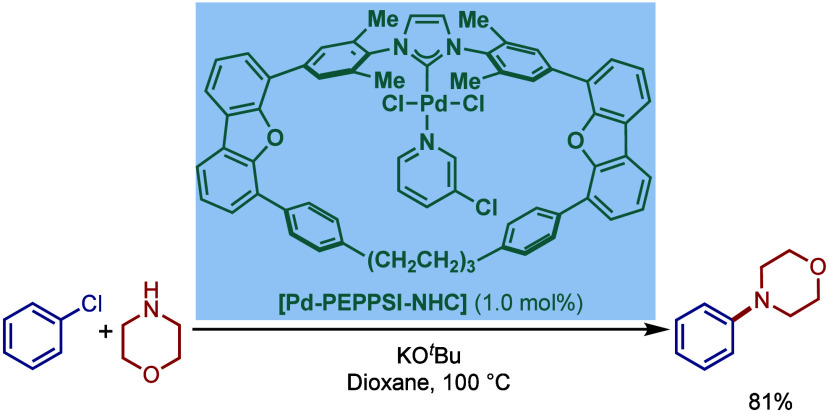
BHA Reaction Catalyzed by Macrocyclic [Pd–PEPPSI–NHC] Complex by Diver

The Organ group evaluated the reactivity of BHA reaction with 1° alkylamines and 2° anilines catalyzed by [Pd–PEPPSI–NHC] complexes (Scheme 87).^230^ They used [Pd–PEPPSI–IPent^Cl^] as a precatalyst in the model coupling of electron-rich 4-chloroanisole with octylamine to investigate the effect of reactant concentration, catalyst, and amine on the initial reaction rates. Furthermore, the effect of different NHC ligands in [Pd–PEPPSI–NHC] complexes was tested. The authors found that a higher selectivity of the amination reaction was observed for more sterically hindered ligands, favoring the formation of the monoarylated product. Interestingly, the highest selectivity (*mono*:*di*, >99:1) was achieved using an allyl-based complex, [Pd­(DiMeIHept^Cl^)­(cin)­Cl] (see Section 2.3.1).

**Scheme 87:**
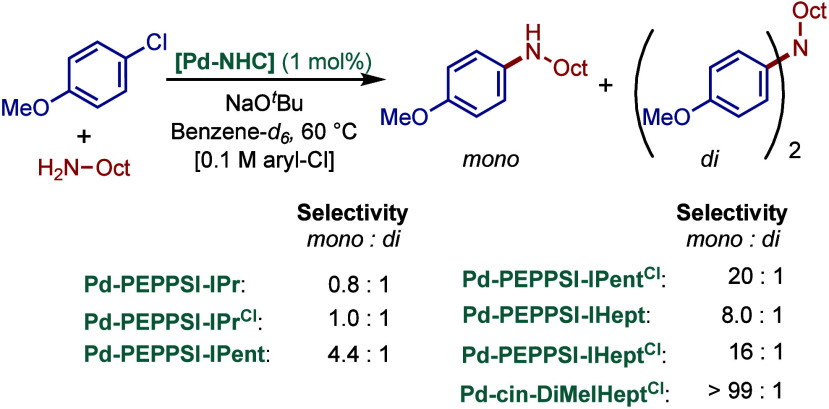
Selectivity in BHA Reaction Using [Pd–PEPPSI–NHC] Complexes by Organ

In 2019, Liu and co-workers reported another class of sterically hindered [Pd–PEPPSI–BIAN–NHC] complexes containing π-extended electron-rich acenaphthoimidazol-2-ylidene scaffold and electron-rich N-aryl wingtips and evaluated their reactivity in BHA reaction (Scheme 88).^231^ These bulky-yet-flexible NHC ligands are easily accessible by a direct condensation of anilines with acenaphthenequinone, followed by cyclization. The structures of [Pd–NHC] complexes were confirmed by X-ray analysis, which showed a distorted square planar geometry around palladium. The %V_*bur*_ of the NHC in [Pd–PEPPSI–IPr*^An^] (%V_*bur*_ = 42.0%) and [Pd–PEPPSI–IPr^OMe*An^] (%V_*bur*_ = 42.6%) were determined to be larger than for [Pd–PEPPSI–IPr] (%V_*bur*_ = 34.3%) and [Pd–PEPPSI–IPr^An^] (%V_*bur*_ = 34.7%) but like [Pd–PEPPSI–IPr*] (%V_*bur*_ = 43.1%). The electron-donating OMe substituent at the *para*-position of the N-aryl groups significantly increased the σ-donating ability as evidenced by the lower TEP value for the corresponding iridium complex, [Ir­(NHC)­(CO)_2_Cl], (IPr^OMe*An^, TEP = 2047.8 cm^–1^; IPr*^An^, TEP = 2048.7 cm^–1^; IPr*, TEP = 2052.7 cm^–1^). This catalyst showed high efficiency in BHA reaction of various heterocycles, such as thiazoles, pyridines, benzoxazoles, and diazines, with heteroaryl amines. Furthermore, this catalyst has been successfully applied to the synthesis of pharmaceuticals, such as Brexpiprazole and Piribedil.

**Scheme 88:**
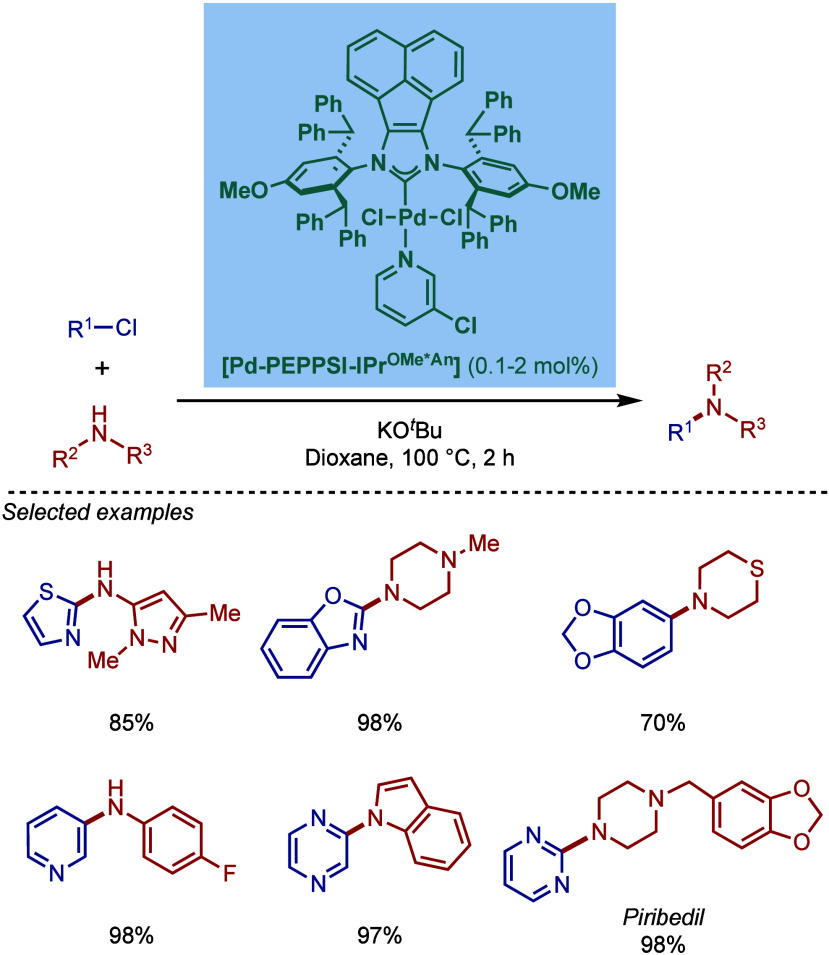
BHA Reaction Catalyzed by Electron-Rich [Pd–PEPPSI–IPr^OMe*An^] Complex by Liu

In 2020, Reddy and co-workers reported a new family of [Pd–PEPPSI–NHC] complexes bearing a benzimidazolium core functionalized with N-benzyl groups and evaluated their activity in the BHA reaction (Scheme 89).^232^ These precatalysts were tested in the synthesis of N-phenylpyridine-3-amine in toluene with KO^*t*^Bu as a base at 90 °C. The highest yield (91%) was observed for the catalyst with sterically demanding 2,4,6-triisopropylbenzyl groups, while the parent [Pd–PEPPSI–IPr] afforded the product in lower yield (86%). Furthermore, X-ray crystallography showed the Pd–C bond length of 1.939 Å, with a simultaneous tilt of the N-benzyl wingtips. The %V_*bur*_ of 44.8%, indicated a considerably greater steric demand from the NHC than that of the corresponding [Pd–PEPPSI–IPr] (%V_*bur*_ = 34.3%) and [Pd–PEPPSI–SIPr] (%V_*bur*_ = 39.3%). The synthetic utility of this catalyst was demonstrated in cross-coupling reactions of 3-chloropyridines with various 1° and 2° amines. Furthermore, this new precatalyst showed higher efficiency in the coupling of heteroaromatic chlorides than the previously reported palladacycle-based SingaCycle–A1.^170^ In the end, evaluation of reactivity versus state-of-the art IPr analogue must take into consideration catalyst synthetic access vs yield considerations and comparisons should always be made vs state-of the art to provide the community with real evidence of relative catalyst performance. This holds for this and all other such catalyst discovery/performance studies.

**Scheme 89:**
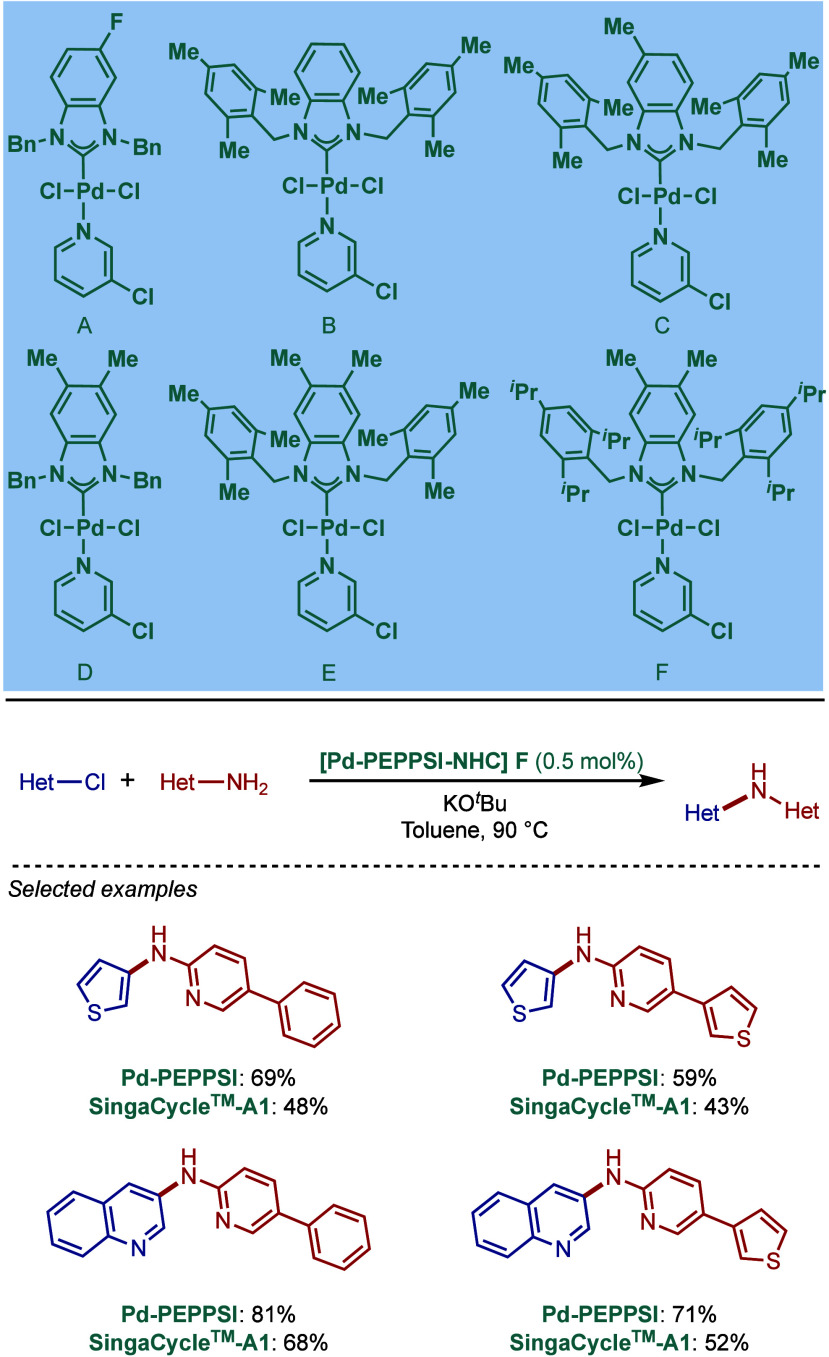
BHA Reaction Catalyzed by Benzimidazolium [Pd–PEPPSI–NHC] Complexes by Reddy

In 2020, Chen and Simmons reported BHA reaction of DNA-conjugated aryl and heteroaryl halides with various amines (Scheme 90).^233^ Screening of Pd catalytic systems, such as *t*BuXPhos–Pd–G1 and G3, BrettPhos–Pd–G3, and [Pd–PEPPSI–IPent^Cl^], showed the highest efficiency of *t*BuXPhos–Pd–G1 (75%) followed by [Pd–PEPPSI–IPent^Cl^] (63%). Interestingly, for other complexes tested, conversions of 30% or less were observed. Under the optimized reaction conditions, the catalytic activity of the [Pd–PEPPSI–IPent^Cl^] was compared with that of other [Pd–PEPPSI]-type complexes, such as IPr, IHept, IPent^Cl^-*o*-picoline, and IPent^Cl^-py. For less sterically hindered or sterically hindered but nonchlorinated precatalysts, no conversion was observed, while the remaining [Pd–NHC] complexes allowed to obtain the desired product in an excellent yields ([Pd–PEPPSI–IPent^Cl^], 92%; [Pd–PEPPSI–IPent^Cl^-*o*-picoline], 91%; [Pd–PEPPSI–IPent^Cl^-py], 91%; [Pd–PEPPSI–IHept^Cl^], 90%). High catalytic efficiency of [Pd–PEPPSI–IPent^Cl^-py] was demonstrated in the BHA reaction of DNA-encoded chemical libraries.

**Scheme 90:**
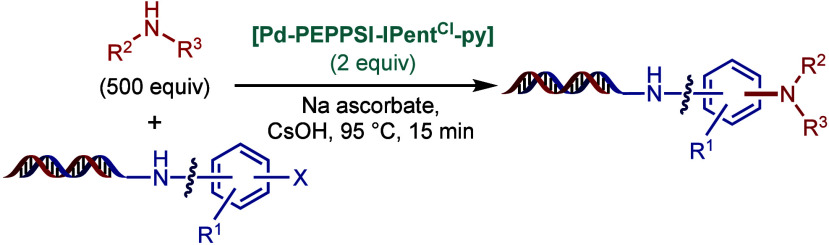
BHA Reaction of DNA-Conjugated Aryl Halides Catalyzed by [Pd–PEPPSI–IPent^Cl^-py] by Chen and Simmons

In 2020, Hayhow and co-workers at AstraZeneca reported the BHA reaction of lenalidomide-derived aryl bromides catalyzed by [Pd–PEPPSI–IHept^Cl^] to access new cereblon-based bifunctional PROTACs (PROTAC = proteolysis targeting chimera) (Scheme 91).^234^ High-throughput screening of different catalysts showed that [Pd–PEPPSI–IPent] led to a full conversion, outperforming other catalysts, such as BrettPhos–Pd–G3, DavePhos–Pd–G3, and Pd­(OAc)_2_/XantPhos. Further optimization of the NHC ligand showed that [Pd–PEPPSI–IHept^Cl^] was the most reactive catalyst. The developed method was successfully applied to the synthesis of isoindolinone derivatives and a complete PROTAC target.

**Scheme 91:**
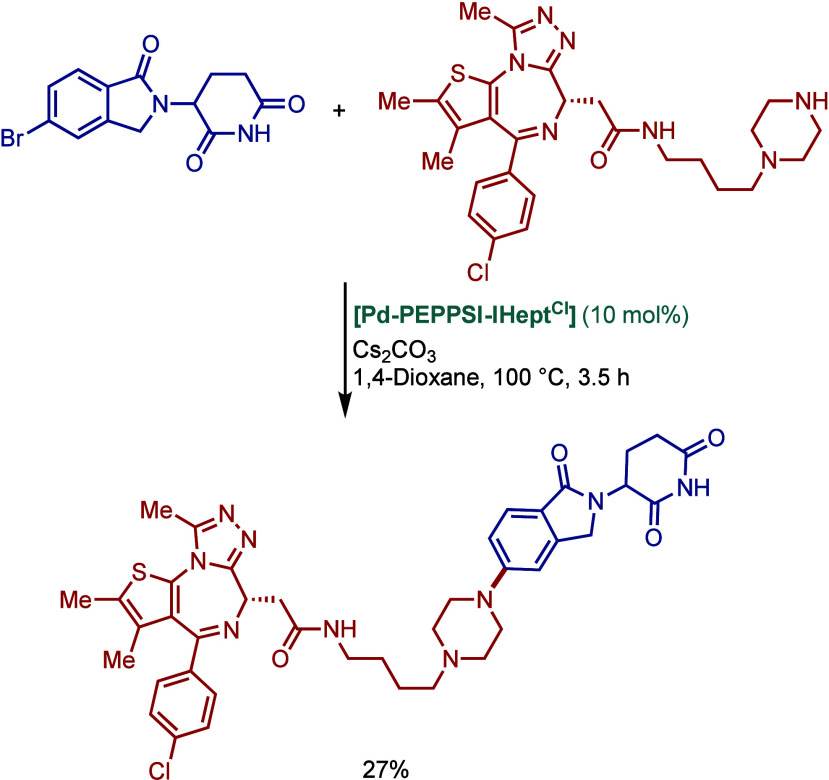
BHA Reaction Catalyzed by [Pd–PEPPSI–IHept^Cl^] for the Synthesis of PROTACs by Hayhow

In 2022, Yao and Xu reported a BHA reaction catalyzed by C3, C4–dianisole-decorated imidazol-2-ylidene [Pd–PEPPSI–IPr] complex (Scheme 92).^235^ Incorporation of sterically hindered and electron-donating *para*-methoxyphenyl groups into the ligand backbone significantly increased the catalytic activity of these complexes. The reactivity was tested in a model reaction between electron-rich 4-chloroanisole and sterically hindered 2,6-diisopropylaniline. This new [Pd–PEPPSI–IPr^4‑MeOC6H4^] complex outperformed both [Pd–PEPPSI–IPr] and [Pd–PEPPSI–IPr^An^], affording the coupling product in 93% yield vs 11% and 20%, respectively. The developed procedure allowed to obtain a series of products with excellent chemoselectivity and functional group tolerance using 0.1 mol % of the catalyst in dioxane at 100 °C under practical aerobic conditions.

**Scheme 92:**
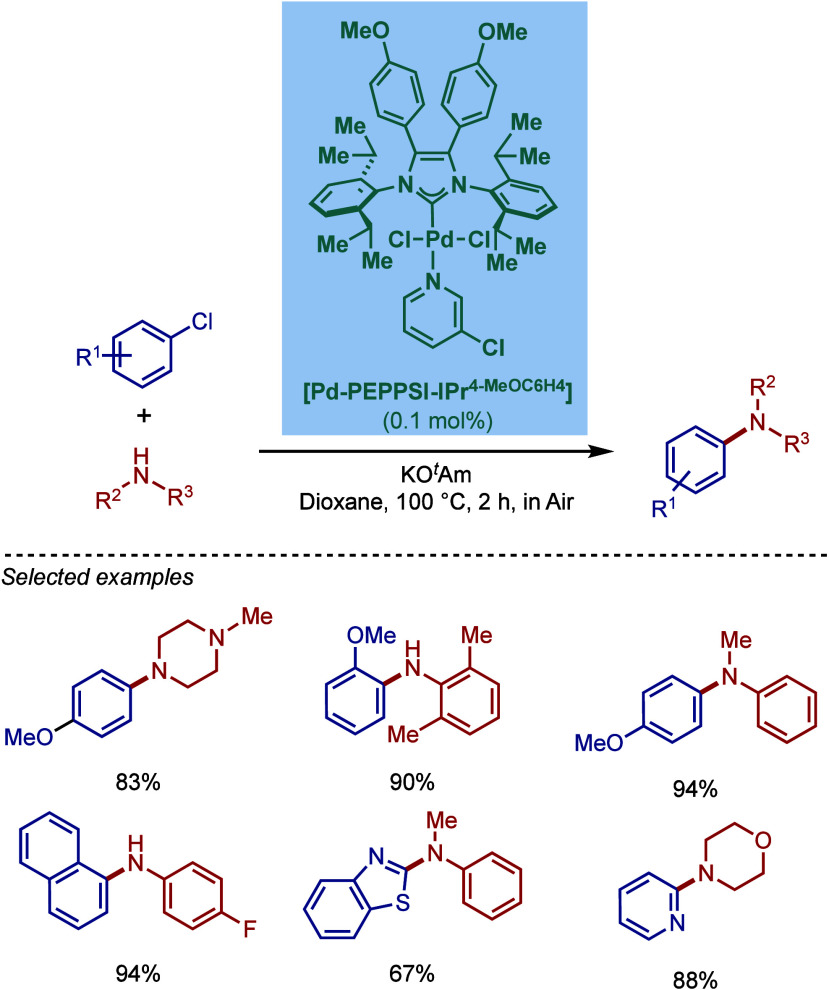
BHA Reaction Catalyzed by Backbone-Modified [Pd–PEPPSI–IPr^4‑MeOC6H4^] Complex by Yao and Xu

Tan and Shen reported a new type of [Pd–PEPPSI–NHC] complexes bearing N-(4-indolyl) wingtips and evaluated their reactivity in BHA reaction (Scheme 93).^236^ The ligands were obtained from the corresponding 4-aminoindoles, representing a rare example of N-heterocycle-functionalized imidazol-2-ylidenes. These [Pd–PEPPSI–NHC] precatalysts were tested in BHA reaction, where the highest catalytic activity was observed for the unsymmetrical precatalyst featuring a bulky 3-isopropyl-1,5,7-trimethyl-2-phenylindol-4-yl moiety and 3-chloropyridine as the ancillary ligand. The X-ray crystallographic analysis showed that this complex is characterized by the Pd–C bond length of 1.966 Å and the %V_*bur*_ of 34.8%, which can be compared with the parent [Pd–PEPPSI–IPr] %V_*bur*_ of 34.3%.

**Scheme 93:**
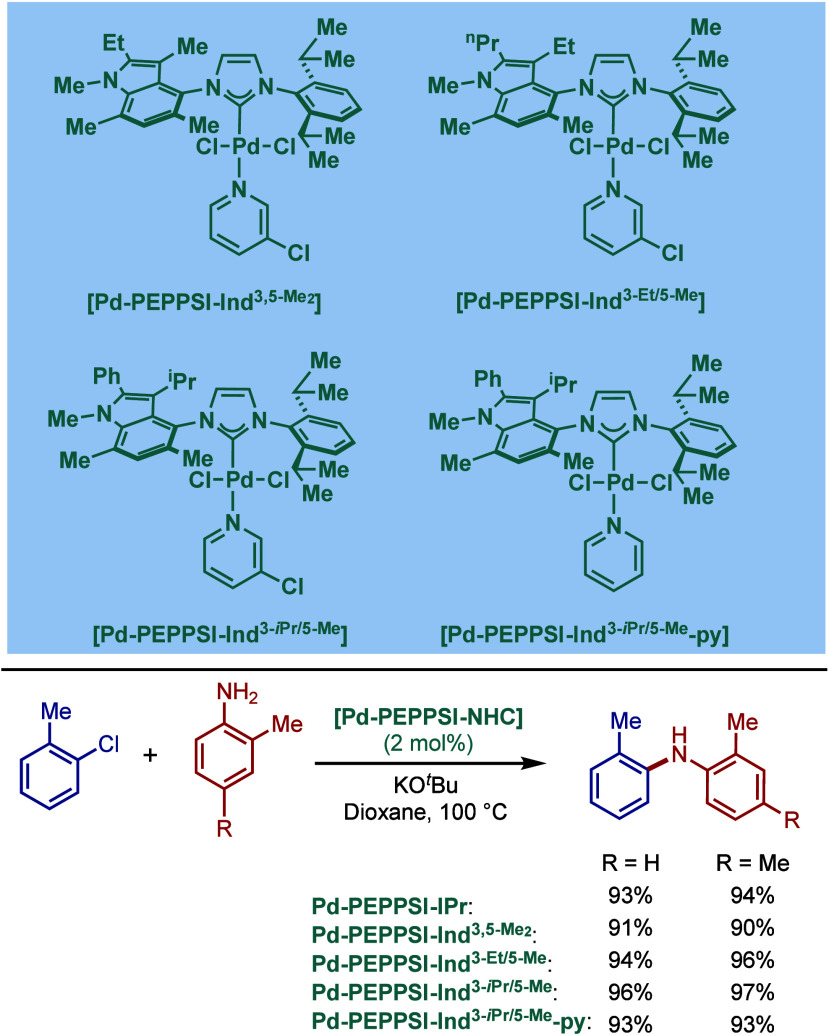
BHA Reaction Catalyzed by N-4-Indolyl-Wingtip-Modified [Pd–PEPPSI–NHC] Complexes by Tan and Shen

In 2022, Liu, Szostak, and co-workers reported the synthesis, characterization, and catalytic performance in BHA reaction of a novel class of large-yet-flexible [Pd–BIAN–NHC] complexes decorated with ITent ligands (Scheme 94, see also Scheme 87, Section 2.3.1).^237^ These air- and moisture-stable precatalysts bear different lengths of α-branched side chains in the *ortho*-positions of the N-aryl wingtips of the acenaphthoimidazol-2-ylidene scaffold. The structures of [Pd–PEPPSI–IHept^An^] and [Pd–PEPPSI–INon^An^] complexes were determined by X-ray crystallographic analysis, and showed distorted square planar geometry around Pd with Pd–C bond lengths of 1.974 Å and 1.970 Å. Catalytic studies in the Buchwald–Hartwig cross-coupling of 4-bromothiazole and N-methylaniline identified [Pd–PEPPSI–INon^An^] as a highly efficient catalyst for this challenging amination (77% yield vs 6% for [Pd–PEPPSI–IPr^An^] or 28% for [Pd–PEPPSI–IPent^An^]). The utility of this novel class of BIAN-derived catalysts was demonstrated in BHA reactions of challenging deactivated five- and six-membered heterocycles with various 1° and 2° amines, affording the coupling products in good to excellent yields. Moreover, this protocol has been successfully applied to the synthesis of polyheteroarylated anilines by double C–N cross-coupling.

**Scheme 94:**
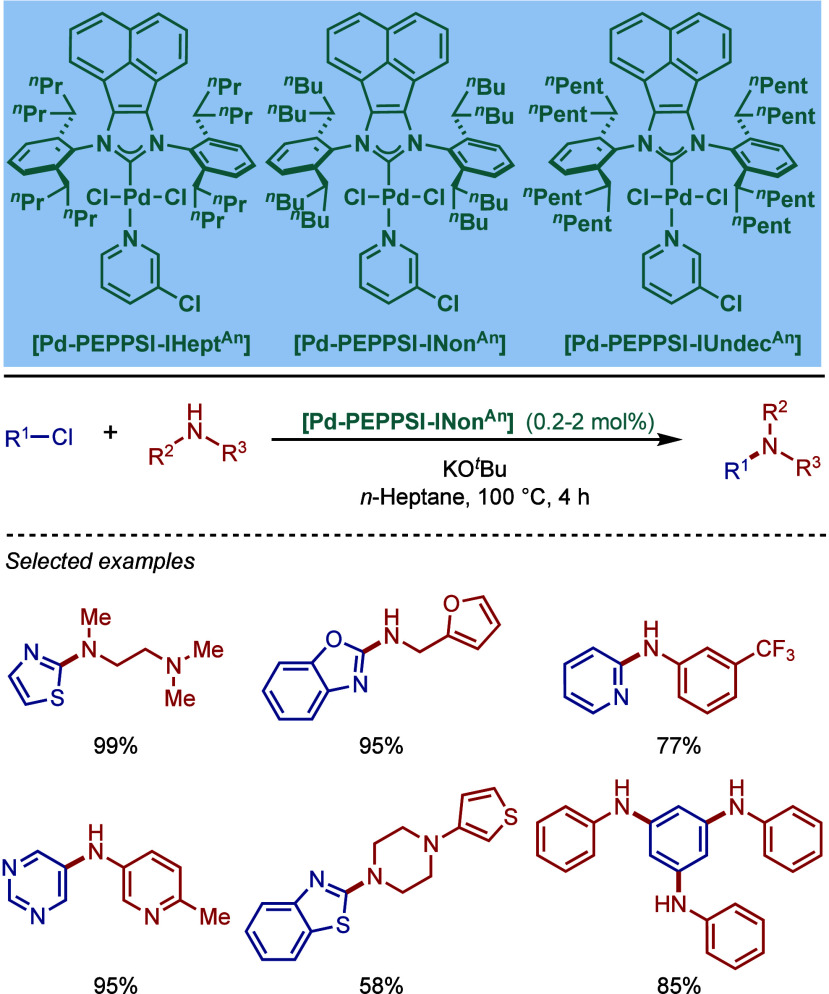
BHA Reaction of Coordinating Heterocycles Catalyzed by [Pd–BIAN–NHC] by Liu and Szostak

In 2022, Suwal and co-workers reported chemoselective BHA reactions of ester-containing heterocyclic halides catalyzed by [Pd–PEPPSI–IPr] (Scheme 95).^238^ The authors identified cesium carbonate as a base permitting for high chemoselectivity of the cross-coupling, while other bases, such as K_2_CO_3_, resulted in lower conversions.

**Scheme 95:**
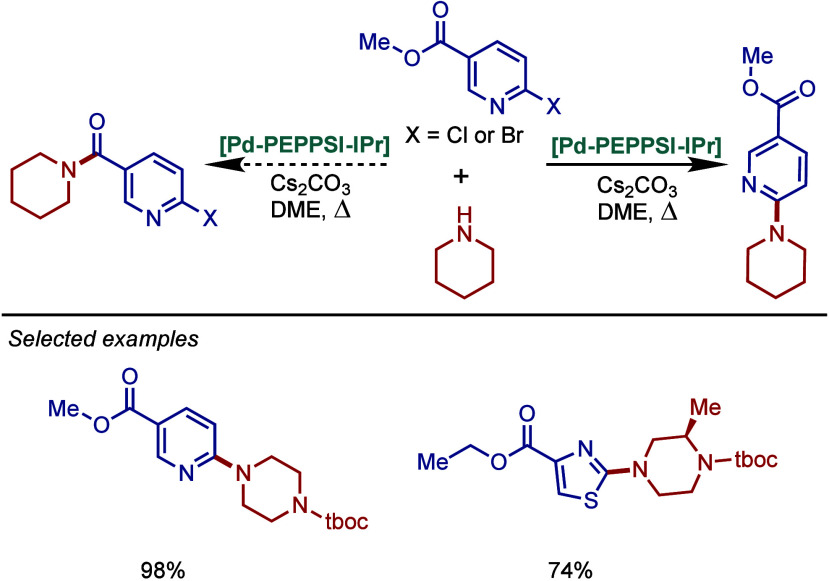
Chemoselective BHA Reaction Catalyzed by [Pd–PEPPSI–IPr] by Suwal

The authors conducted a series of experiments to elucidate the role of the neighboring nitrogen atom of the heterocycle on the course of the catalytic cycle. The authors proposed a mechanism involving coordination of the heterocyclic electrophile to the palladium center, facilitating the transmetalation step.

In 2022, Ananikov, Chernyshev, and co-workers reported N–NHC coupling as a possible catalyst deactivation pathway during BHA reactions catalyzed by [Pd–PEPPSI–NHC] precatalysts (Scheme 96).^239^ They examined a series of [Pd–PEPPSI–NHC] complexes (10 mol %) in a model coupling reaction between bromobenzene and aniline in the presence of KO^*t*^Bu in dioxane at 100 °C. The best catalytic efficiency was observed for [Pd–PEPPSI–IPr-py] complex, leading to the monoarylated product in 92% and traces of diarylated product. Electrospray ionization/high resolution mass spectrometry revealed byproducts from H–NHC, C–NHC and O–NHC bond formation. Furthermore, the formation of a N–NHC coupling product was also observed in the postreaction mixtures, which represents an underappreciated pathway for the deactivation of [Pd–PEPPSI–NHC] complexes during BHA reactions.

**Scheme 96:**
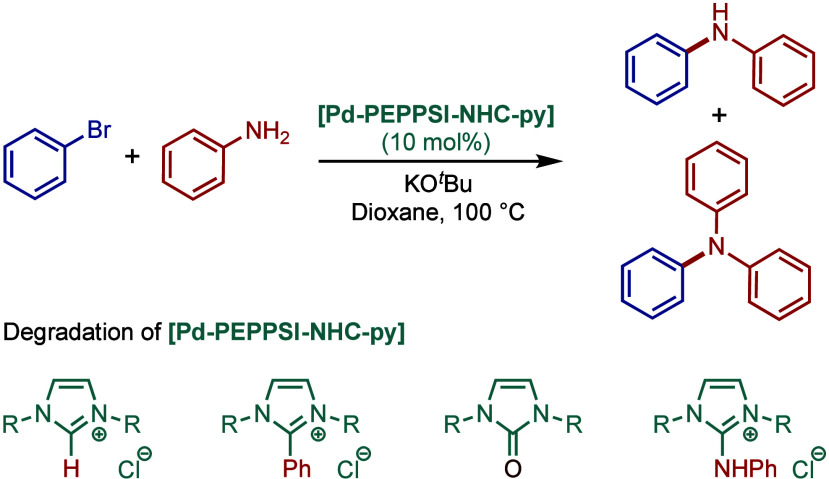
BHA Reaction of Bromobenzene Catalyzed by [Pd–PEPPSI–NHC] Complexes by Ananikov and Chernyshev

In 2023, Szostak and co-workers reported a new class of highly sterically hindered N-aliphatic NHC ligands bearing *t*-Oct side chain and evaluated their activity in BHA reaction (Scheme 97).^240^ Replacement of the *t*-Bu group in the popular I*t*Bu ligand with *t*-Oct resulted in a significant increase in %V_*bur*_ from 39.6% to 44.7% (determined for linear [Au­(NHC)­Cl] complexes, NHC = I*t*Bu, I*t*Oct). The corresponding [Pd–PEPPSI–I*t*Oct] complex was prepared using the standard procedure with PdCl_2_ and 3-Cl-py in the presence of K_2_CO_3_ in 76% yield. The catalytic activity of [Pd–PEPPSI–I*t*Oct] in BHA reaction of 4-methoxybromobenzene with morpholine afforded the coupling product in 86% yield, while the same amination catalyzed by the I*t*Bu congener, [Pd–PEPPSI–I*t*Bu], gave the corresponding product only in 18% yield.

**Scheme 97:**
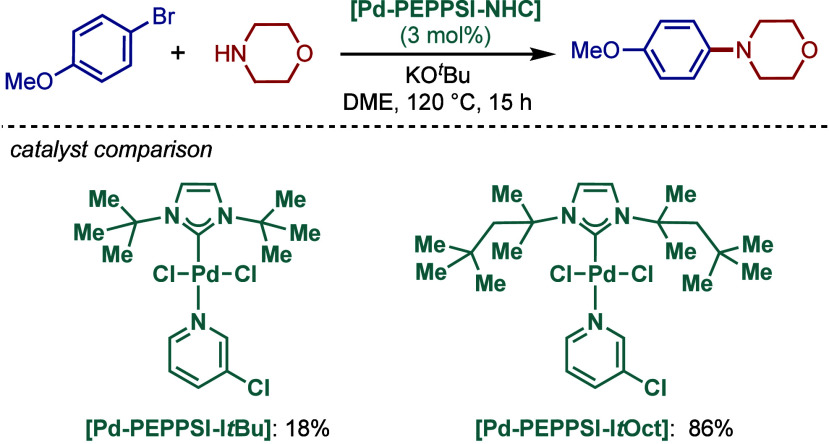
BHA Reaction Catalyzed by N-Aliphatic, Sterically-Hindered [Pd–PEPPSI–I*t*Oct] Complexes by Szostak

In 2023, Szostak and co-workers reported a well-defined, highly hindered [Pd–PEPPSI–IPr^#^] precatalyst, prepared by a modular peralkylation of anilines and evaluated its activity in BHA reaction (Scheme 98).^241^ This [Pd–PEPPSI–IPr^#^] complex has been characterized by a square planar geometry at palladium and Pd–C bond lengths of 1.978 Å and 1.965 Å (two independent molecules in the unit cell). The %V_*bur*_ was determined as 40.2% and 38.2%, which is higher than for the [Pd–PEPPSI–IPr] and [Pd–PEPPSI–IPent] counterparts (IPr: 34.3%; IPent: 37.9%), but lower than for the cinnamyl congener, [Pd­(IPr^#^)­(cin)­Cl], %V_*bur*_ = 44.7%. The catalytic activity of this new precatalyst was evaluated in BHA reaction of 4-chloroanisole with morpholine in the presence of LiHMDS as a base, which afforded the product in 98% yield. The observed activity was like that obtained in the case of the cinnamyl congener, [Pd­(IPr^#^)­(cin)­Cl] (see Scheme 27, Section 2.3.1).

**Scheme 98:**
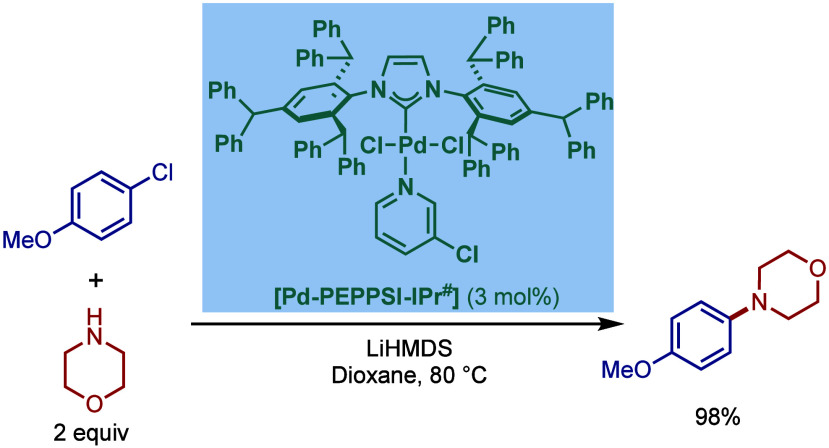
BHA Reaction Catalyzed by N-Aromatic, Sterically-Hindered [Pd–PEPPSI–IPr^#^] Complexes by Szostak

In 2023, Ananikov, Chernyshev, and co-workers reported BHA reaction of 3-amino-1,2,4-triazoles with (hetero)­aryl halides catalyzed by [Pd–PEPPSI–IPr*^OMe^-py] (Scheme 99).^242^ The authors found that the arylation of the amino group was possible using sterically hindered ligands, such as IPr*^OMe^, and TPEDO (1,1,2,2-tetraphenylethane-1,2-diol) as a precatalyst activator. Among different [Pd–NHC] and Pd/phosphine systems evaluated, [Pd–PEPPSI–IPr*^OMe^] and chloro-dimer [Pd­(IPr*^OMe^)­Cl_2_]_2_ showed the highest activity. The authors proposed that the excellent selectivity of this coupling results from the fast reductive elimination step. This protocol was successfully applied to the arylation of various coordinating heterocycles bearing amino groups.

**Scheme 99:**
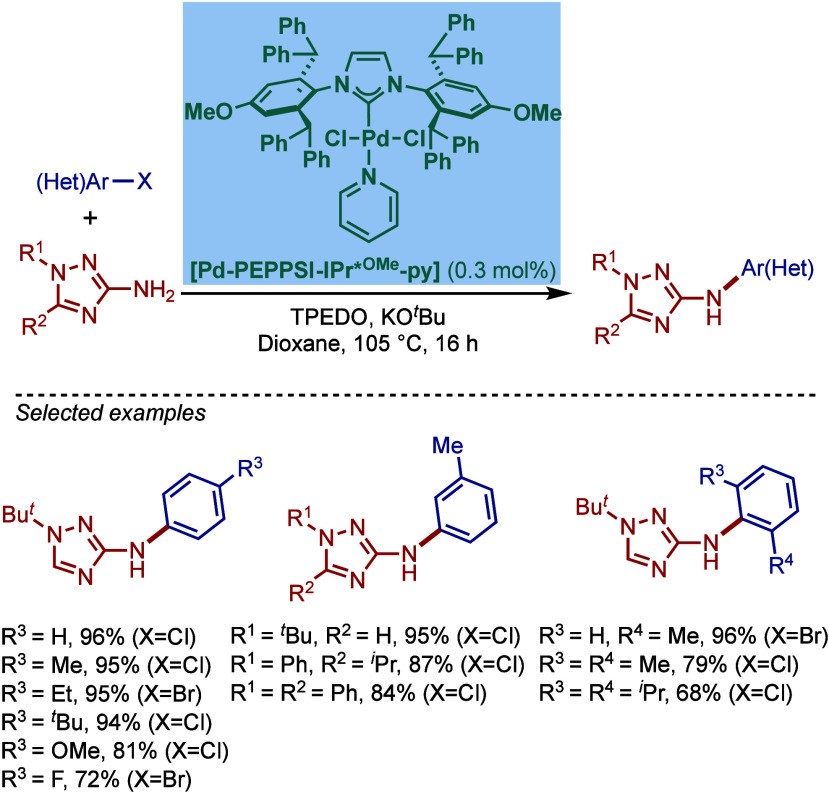
BHA Reaction of 3-Amino-1,2,4-triazoles by Ananikov and Chernyshev

The synthesis and catalytic performance of a CAAC-derived [Pd–PEPPSI] precatalyst (CAAC = cyclic­(alkyl)­(amino) carbene) was reported by Munz in 2023 (Scheme 100).^243^ The corresponding palladium–carbene complex was obtained in the presence of KHMDS in benzene (95% yield) due to the higher p*K*_a_ of the CAAC salt compared to the imidazolium congener. Based on X-ray crystallographic analysis, the lengths of Pd–C and Pd–N bonds were 1.9577 Å and 2.1193 Å, respectively, which was in the expected range for the [Pd–PEPPSI–IPr] counterpart. Furthermore, both the CAAC and [Pd–PEPPSI–IPr] complexes were successfully reduced to bis­(NHC) palladium(0) complexes, [Pd­(NHC)_2_], by using potassium on graphite. The catalytic activities of these Pd­(II) and Pd(0) complexes were tested in BHA reaction of aryl chlorides under comparatively mild reaction conditions (KO^*t*^Bu, toluene, 60 °C), leading to the amination products in similar yields.

**Scheme 100:**
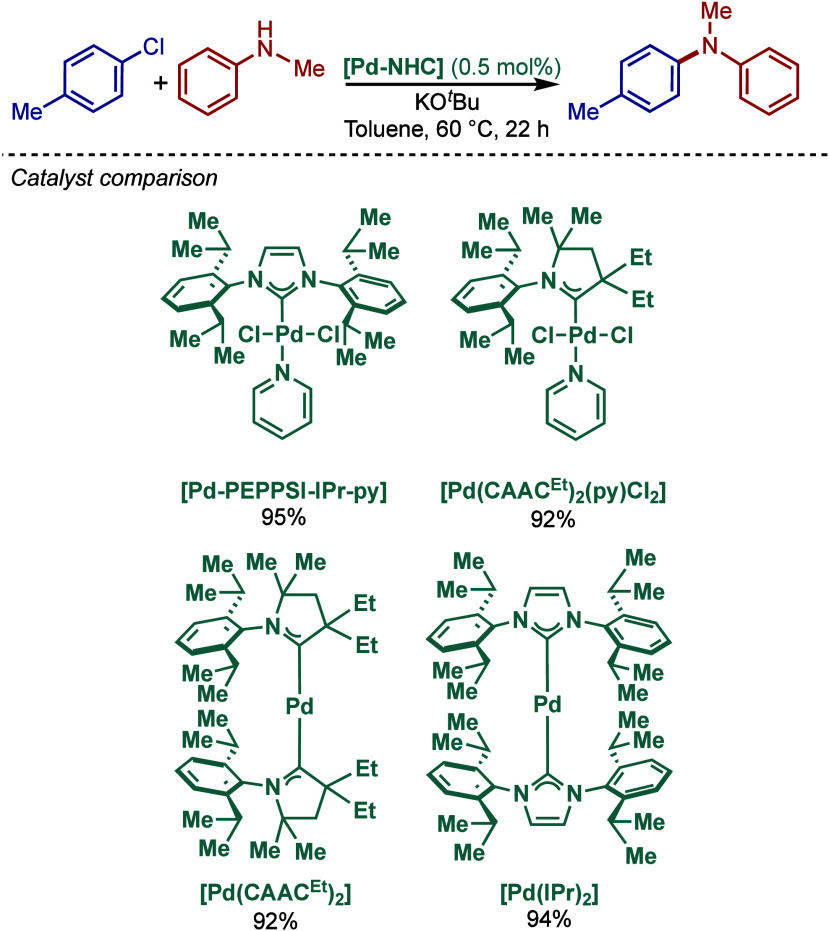
BHA Reaction Catalyzed by [Pd­(CAAC^Et^)­(py)­Cl_2_] by Munz

In 2024, Ananikov and co-workers reported a BHA reaction catalyzed by a mixed [Pd–NHC]/phosphine system for C–N cross-coupling reactions (Scheme 101).^244^ Extensive evaluation of different NHC and PR_3_ ligands, including IPr, IMes, IAd, PPh_3_, PCy_3_, JohnPhos, *t*BuXPhos, SPhos, RuPhos, enabled the identification of [Pd–PEPPSI–IPr-py] together with RuPhos as the most reactive combination of ligands. These conditions were applied to BHA reaction of aryl bromides (KO^*t*^Bu, toluene, 85 °C), where the combined use of NHC and phosphine ligand (1 mol % each) led to improved overall yields compared to the use of each ligand alone.

**Scheme 101:**
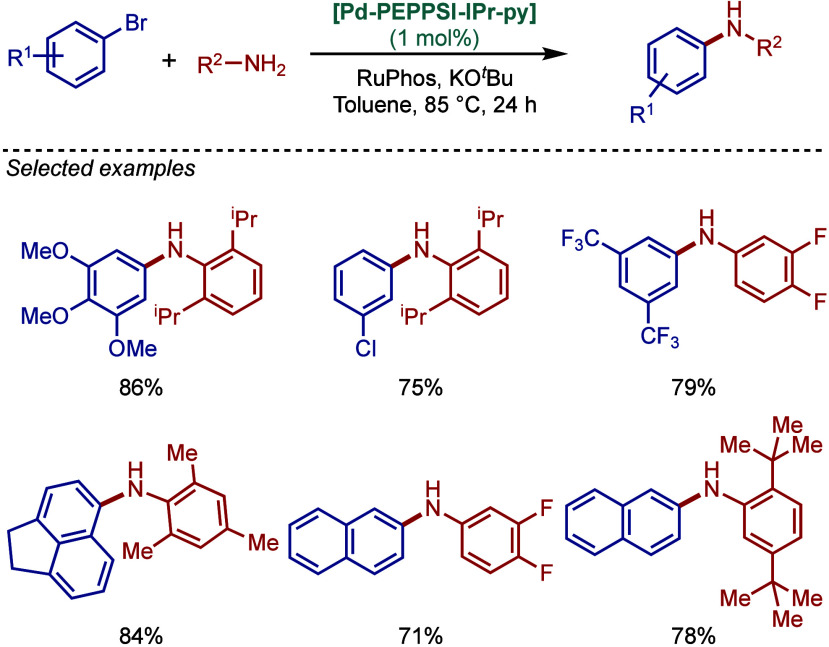
BHA Reaction Catalyzed by [Pd–PEPPSI–IPr-py]/RuPhos by Ananikov

In 2024, Korotkikh and co-workers reported a comparison of [Pd–NHC] complexes in BHA reaction of aryl chlorides (Scheme 102).^245^ The authors prepared previously described [Pd–PEPPSI–IPr*-py][Bibr ref246] by a modified method using acetonitrile as a solvent. The catalytic activity was compared with [Pd–PEPPSI–IPr] and nonpyridine ligated [Pd­(IPr*)­Cl_2_]. Under the optimized conditions (0.1 mol % [Pd], NaO^*t*^Bu, 1,4-dioxane, 110 °C), [Pd–PEPPSI–IPr*-py] was significantly more reactive than the parent [Pd–PEPPSI–IPr] complex.

**Scheme 102:**
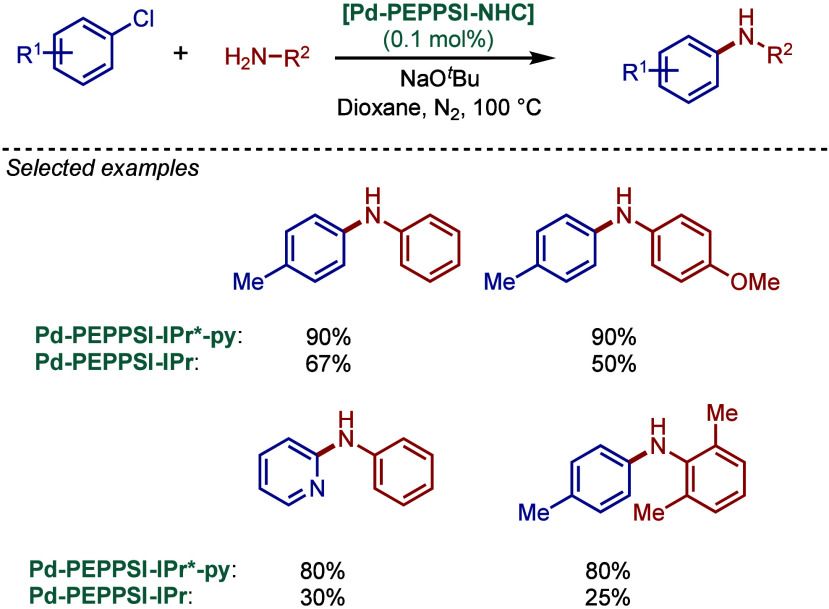
BHA Reaction Catalyzed by [Pd–PEPPSI–IPr*-py] by Korotkikh

In 2024, Shen and co-workers reported another study on BHA reaction catalyzed by [Pd–PEPPSI–NHC] complexes containing N-(4-indolyl) wingtip on the imidazol-2-ylidene scaffold (Scheme 103).^247^ Precatalysts containing sterically demanding *i*-Pr group at the C3 and C5 positions of the indole ring were synthesized in a direct analogy to the Dipp wingtip. The structure of this [Pd–PEPPSI–NHC] complex revealed a Pd–C bond length of 1.936 Å. The σ-donating properties were determined by comparison of ^1^H–^13^C coupling constants, showing a value range of 222.5 to 224.8 Hz.^248^ The catalytic performance was evaluated in BHA reaction of aryl chlorides (KO^*t*^Bu, dioxane, 100 °C) in comparison with the parent [Pd–PEPPSI–IPr]. This N-(4-indolyl)-based precatalyst showed higher efficiency than the IPr counterpart, which was ascribed to the stronger σ-donicity and π-π interactions between the indolyl fragment and electron-deficient heterocyclic substrates.

**Scheme 103:**
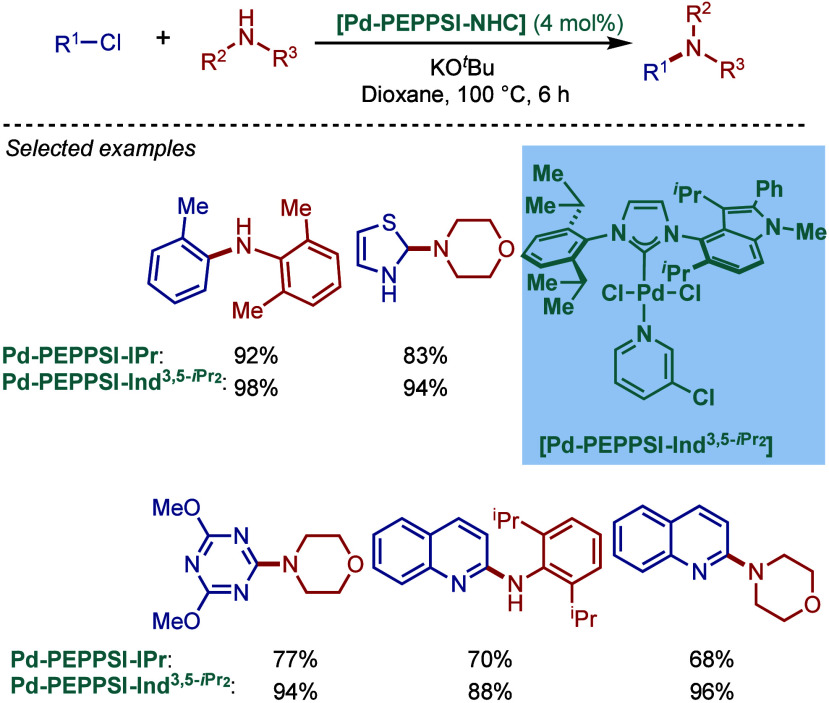
BHA Reaction Catalyzed by Sterically-Hindered N-4-Indolyl-Wingtip-Modified [Pd–PEPPSI–NHC] Complexes by Shen

#### 2.3.9 [Pd­(NHC)­CpCl] Complexes

In 2009, Jin and co-workers reported an important study on the synthesis and application in BHA reaction of well-defined, air-stable [Pd­(NHC)­CpCl] complexes (Scheme 104).^249^ These catalysts were prepared from the corresponding [Pd­(NHC)­Cl_2_]_2_ dimers (NHC = IMes, IPr, SIMes, SIPr) by the reaction with sodium cyclopentadienylide at room temperature. These complexes were found to be stable in air in a solid form but slowly decompose in solution by dissociation of the cyclopentadienyl ring. X-ray analysis of [Pd­(SIPr)­CpCl] revealed η^5^-coordination of the cyclopentadienyl ring with the Pd–C bond length of 1.977 Å. Based on previous studies showing that SIPr outperforms IPr in the BHA reaction, [Pd­(SIPr)­CpCl] was used to evaluate the catalytic activity in the amination of aryl chlorides. This catalyst showed very high reactivity in the cross-coupling with various 1° and 2° amines at room temperature (NaO^*t*^Bu, DME). Furthermore, arylation of α-chiral alkylamines led to optically pure N-arylamine derivatives.

**Scheme 104:**
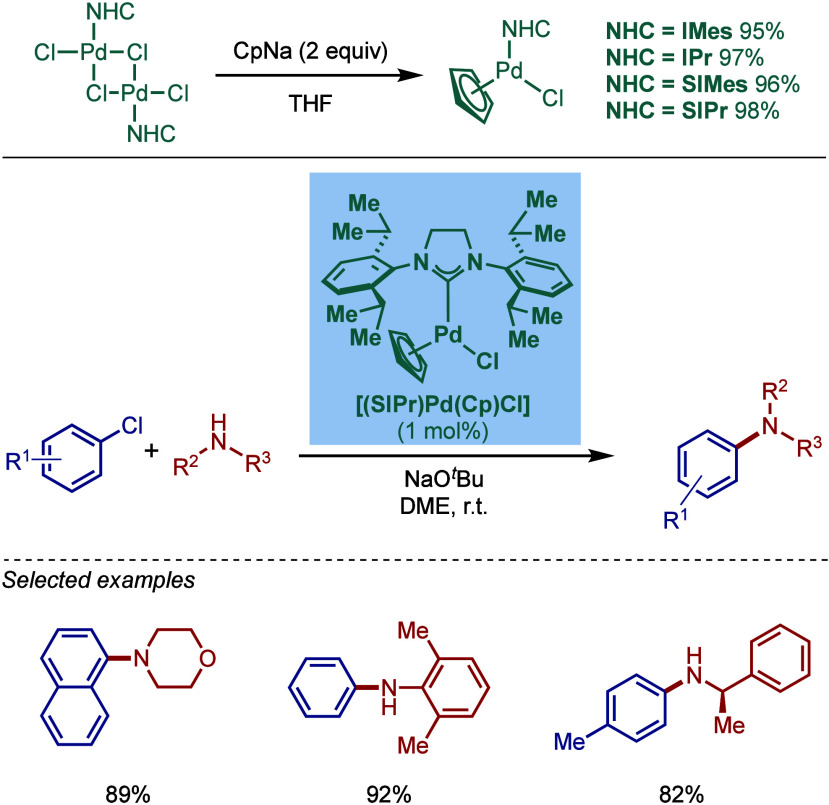
BHA Reaction Catalyzed by [(NHC)­Pd­(Cp)­Cl] Complexes by Jin

#### 2.3.10 [Pd­(NHC)­(NR_2_)­Cl_2_] Complexes

##### 2.3.10.1 Oxazoline and Oxazole

In 2014, Lu and co-workers reported the synthesis and catalytic activity in BHA reaction of [Pd­(NHC)­Cl_2_(4,5-dihydrooxazole)] complexes (Scheme 105).^250^ These precatalysts were obtained from the corresponding imidazolium salts, palladium­(II) chloride and oxazoline derivatives by complexation in THF in the presence of K_2_CO_3_ in 67–86% yields. The structure of the parent complex (NHC = IPr; 2-phenyl-4,5-dihydrooxazole) was confirmed by X-ray crystallographic analysis and showed a distorted square planar geometry around Pd with chloride anions perpendicular to the carbene center and 4,5-dihydrooxazole in a *trans*-position. The lengths of Pd–C and Pd–N bonds were of 1.959Å and 2.085Å, respectively, which are shorter than for the [Pd–PEPPSI–IPr] congener (1.969Å and 2.137 Å). The %V_*bur*_ was 35.6%, which can be compared with 34.3% for [Pd–PEPPSI–IPr]. In the model BHA reaction, complexes with stronger σ-donating properties were more efficient using KO^*t*^Bu in dioxane at 90 °C. The parent catalyst was applied to the cross-coupling of a broad range of aryl and heteroaryl chlorides with 1° and 2° amines at 0.5 mol % loading. Furthermore, amines containing IPr ligand led to series of various products in high yields using a catalyst loading of 0.5 mol %. In addition, a large-scale reaction at 100 mmol scale at 0.05 mol % loading was successfully demonstrated.

**Scheme 105:**
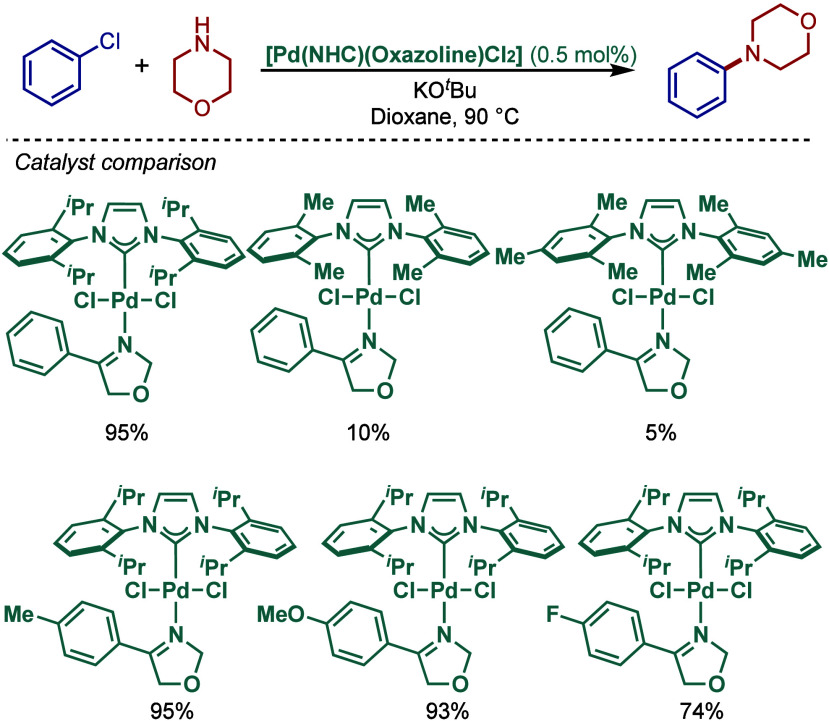
BHA Reaction Catalyzed by [Pd­(NHC)­Cl_2_(Oxazoline)] Complexes by Lu

In 2020, Lu and co-workers reported highly chemoselective BHA reaction of polyaryl chlorides catalyzed by [Pd­(IPr)­Cl_2_(2-Me-4,5-dihydrooxazole)] complex (Scheme 106).^251^ The desired precatalyst was obtained using a previously described procedure from imidazolium salt, PdCl_2_ and 2-methyl-4,5-dihydrooxazole in the presence of K_2_CO_3_ in THF at 80 °C in 92% yield.^252^ The structure was determined by X-ray analysis showing the Pd–C bond length of 1.969 Å and square planar geometry around Pd. The authors optimized monoselective BHA reaction using 1,3-dichlorobenzene and morpholine as model substrates. The highest ratio of products (*mono*:*di* = 84%:12%, isolated yields) was observed using KO^*t*^Bu as a base and toluene as a solvent at 70 °C. Using this procedure, a variety of 1° and 2° amines were coupled with 1,2-, 1,3-, and 1,4-dichlorobenzenes, leading to the desired monoaminated compounds in good to excellent yields.

**Scheme 106:**
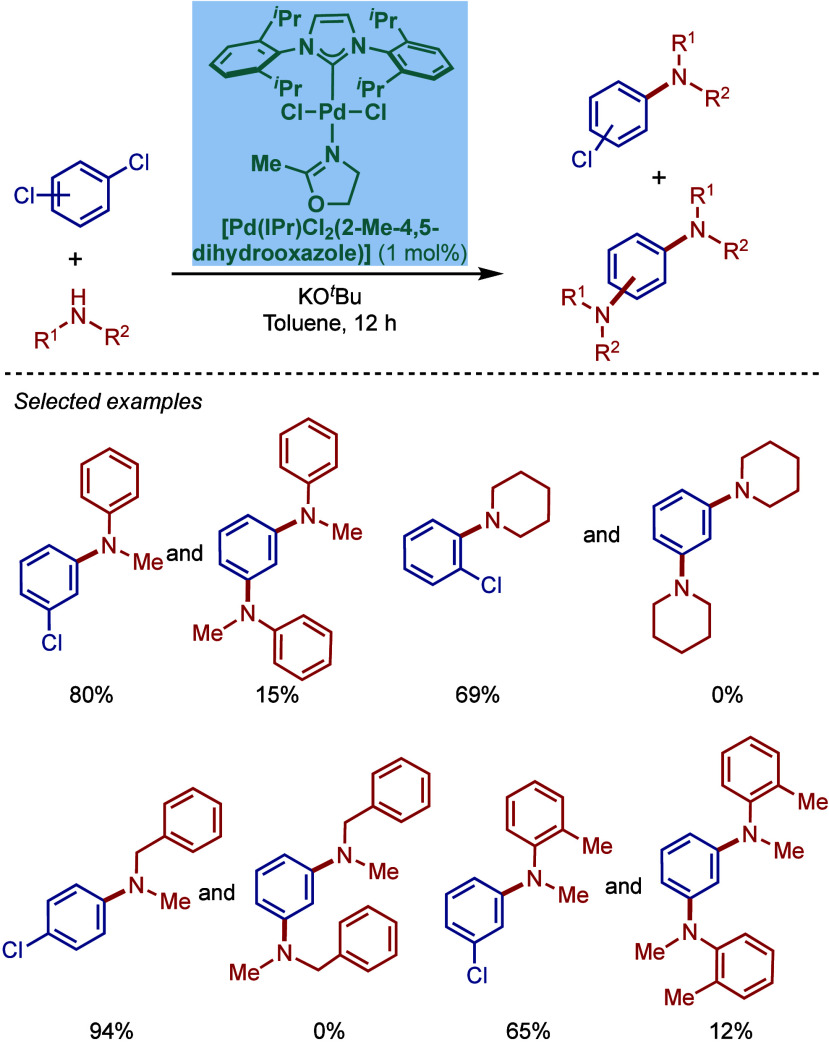
Chemoselective BHA Reaction of Dichlorobenzenes Catalyzed by [Pd­(NHC)­Cl_2_(Oxazoline)] Complexes by Lu

In 2021, Shao and co-workers reported [Pd­(NHC)­Cl_2_(oxazole)] complexes bearing 5-phenyloxazole as an ancillary ligand and evaluated their activity in BHA reaction (Scheme 107).^253^ Air-stable [Pd­(II)–NHC] precatalysts (NHC = IPr, IXyl, IMes) were synthesized from the corresponding imidazolium salts, PdCl_2_, and 5-phenyloxazole in the presence of K_2_CO_3_ in THF at reflux in 74–85% yields. The structure of [Pd­(IPr)­Cl_2_(5-Ph-oxazole)] complex was determined by X-ray crystallography and showed the Pd–C bond length of 1.963 Å. This complex was found to efficiently catalyze BHA reaction of aryl chlorides with and various 1° anilines and 2° aliphatic and aromatic amines, affording the products in excellent yields at catalyst loading of 0.01–0.05 mol % in the presence of KO^*t*^Bu in toluene at 110–130 °C.

**Scheme 107:**
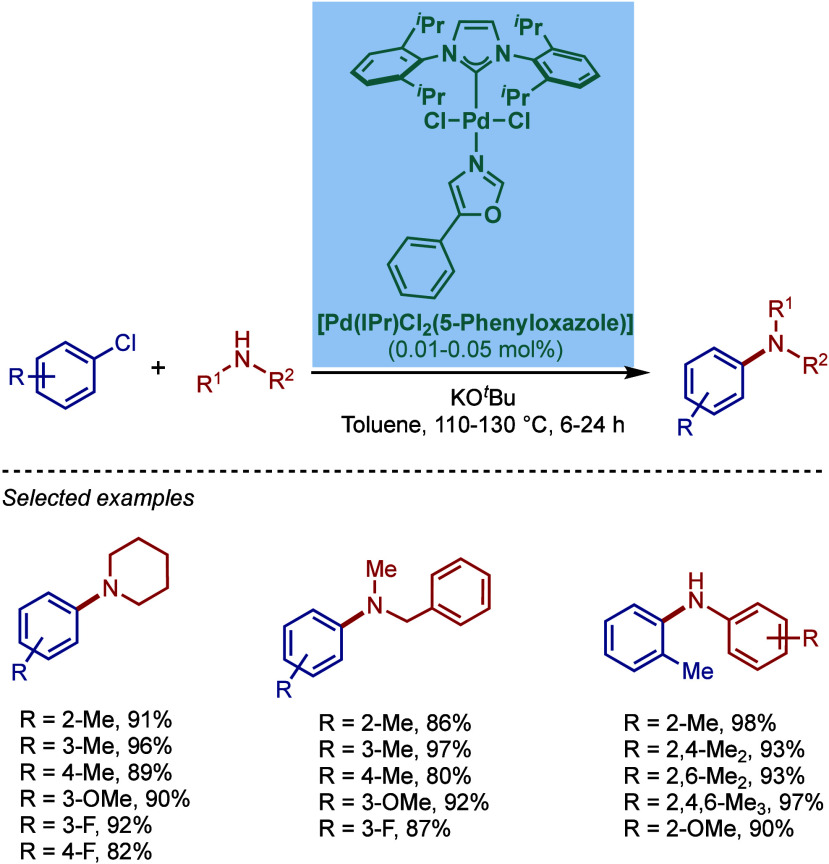
BHA Reaction Catalyzed by [Pd­(IPr)­Cl_2_–Oxazole] Complex by Shao

##### 2.3.10.2 Imidazole

In 2011, Shao and co-workers reported the synthesis of well-defined, air-stable [Pd­(NHC)­Cl_2_(im)] complexes containing 1-methylimidazole as the ancillary ligand and evaluated their reactivity in BHA reaction (Scheme 108).^254^ The desired complexes were obtained from readily available imidazolium salts, 1-methylimidazole and PdCl_2_ by complexation in refluxing THF in the presence of K_2_CO_3_. The X-ray structure of the [Pd­(IPr)­Cl_2_(im)] complex showed that the geometry around Pd is square planar with the Pd–C and Pd–N bond lengths of 1.954 Å and 2.088 Å, respectively. Catalytic activity was evaluated in the model BHA reaction of chlorobenzene with morpholine, where this complex showed high efficiency using KO^*t*^Bu in dioxane at 70 °C. The same complex was also applied to the BHA reaction of aryl chlorides with sterically hindered anilines and 1° alkyl amines using KO^*t*^Bu in toluene at 110 °C (Scheme 108).^255^ These conditions permitted for the synthesis of exceedingly sterically hindered diarylamines, while the use of 1° long-chain *n*-octylamine afforded mixtures of mono- and bis-aminated products.

**Scheme 108:**
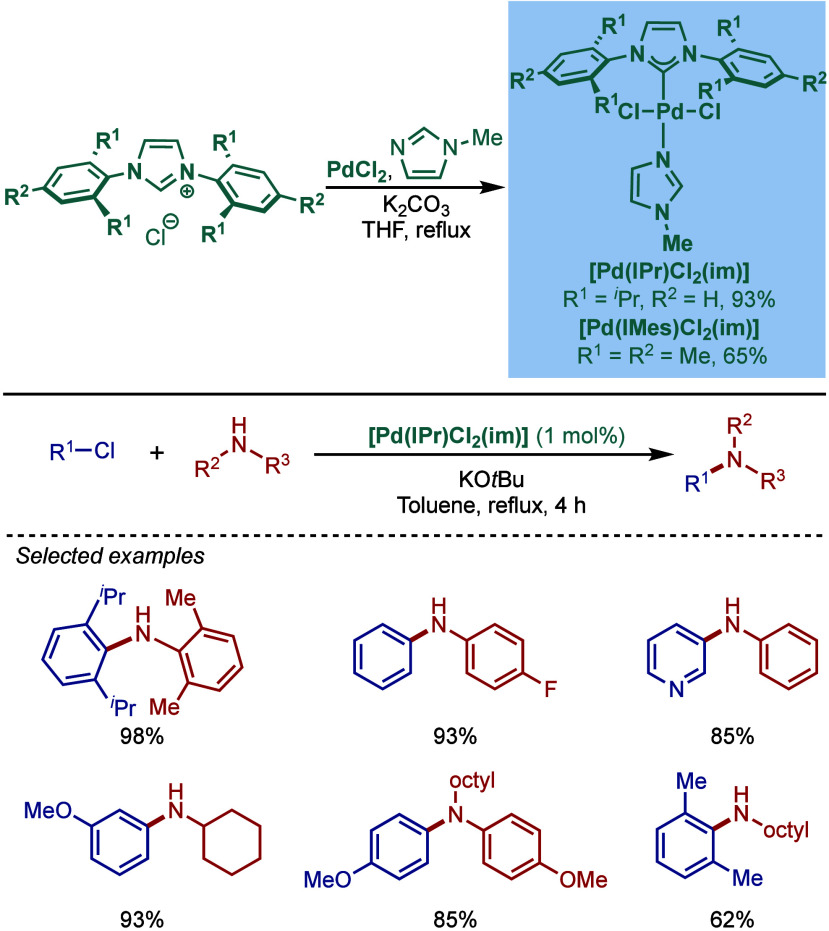
BHA Reaction Catalyzed by [Pd­(IPr)­Cl_2_(Imidazole)] Complexes by Shao

The Shao group also considered the possibility of using N,N-dimethylformamide (DMF) as a source of the NMe_2_ group in BHA reactions catalyzed by their [Pd­(IPr)­Cl_2_(im)] complex (Scheme 109).^256^

**Scheme 109:**
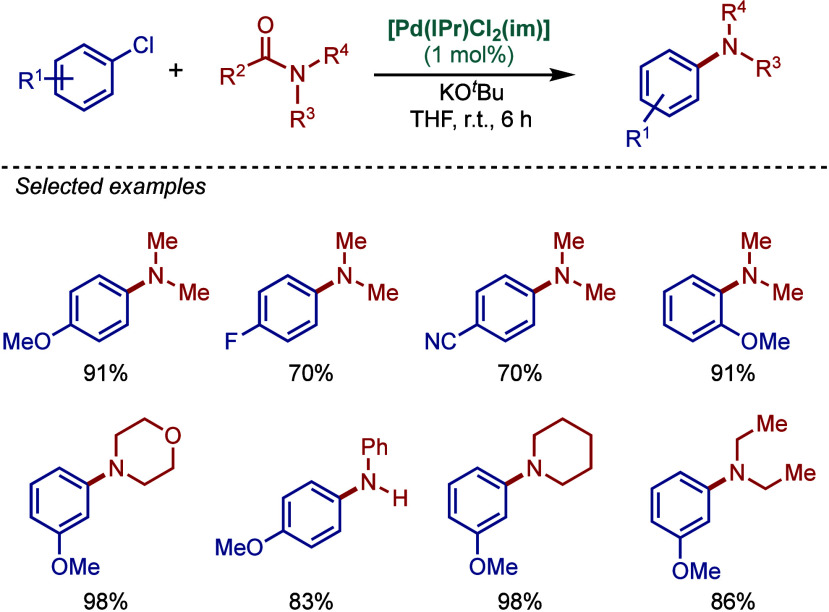
BHA Reaction Using Amides as Amine Source Catalyzed by [IPr–PdCl_2_–Imidazole] Complex by Shao

They found that this Pd–NHC efficiently catalyzed the amination of aryl chlorides in the presence of KO^*t*^Bu in THF or DMF at room temperature. This methodology allowed for the synthesis of a series of N,N-dimethylanilines in excellent yields up to 99%. Furthermore, in addition to formamide derivatives, other N-sources such as N,N-dimethylacetamide and N,N-dimethylbenzamide could be applied, affording the coupling products in 81–99% yields. The proposed mechanism involves a base-induced cleavage of the amide bond to give metalated amine, which then undergoes transmetalation with [Ar–Pd­(II)–Cl] (Figure 5).

**Figure 5:**
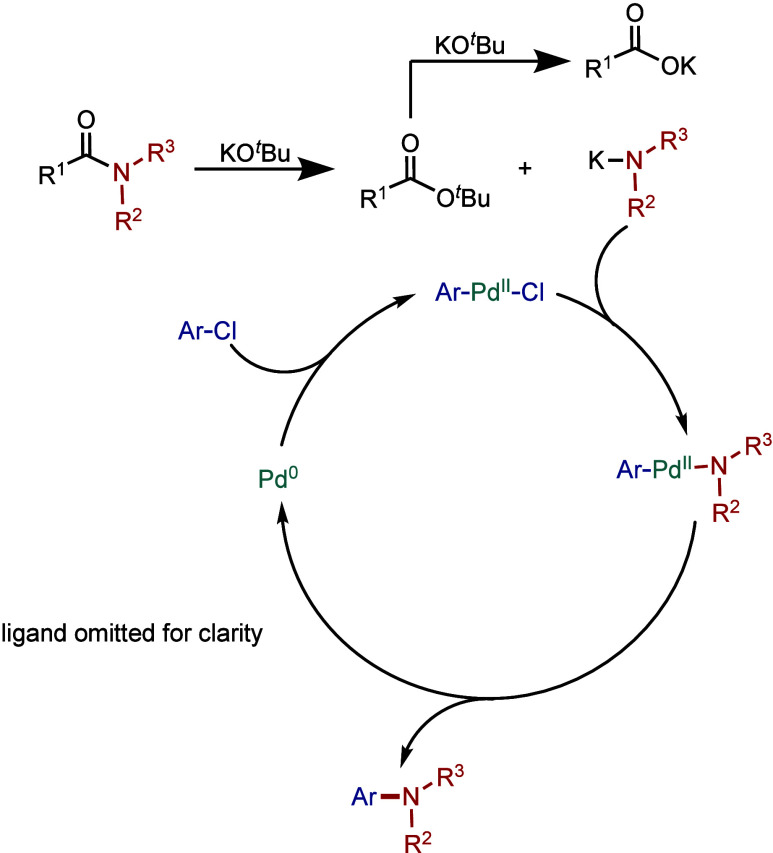
Proposed catalytic cycle for BHA reaction using amides as amine source.

In 2013, a related BHA reaction process for the C–N coupling of benzyl chlorides with amines generated by base-induced decomposition of N,N-dialkylformamides catalyzed by the same [Pd­(IPr)­Cl_2_(im)] complex was reported by Lu and co-workers (Scheme 110).^257^ In this case, the use of sodium hydroxide as a base and water as a solvent enabled the synthesis of N,N-dialkylbenzylamines in good to excellent yields under eco-friendly conditions. It is worth noting that the reaction was not observed using weaker bases, such as carbonates or bicarbonates, while other hydroxides, such as KOH, resulted in lower conversions.

**Scheme 110:**
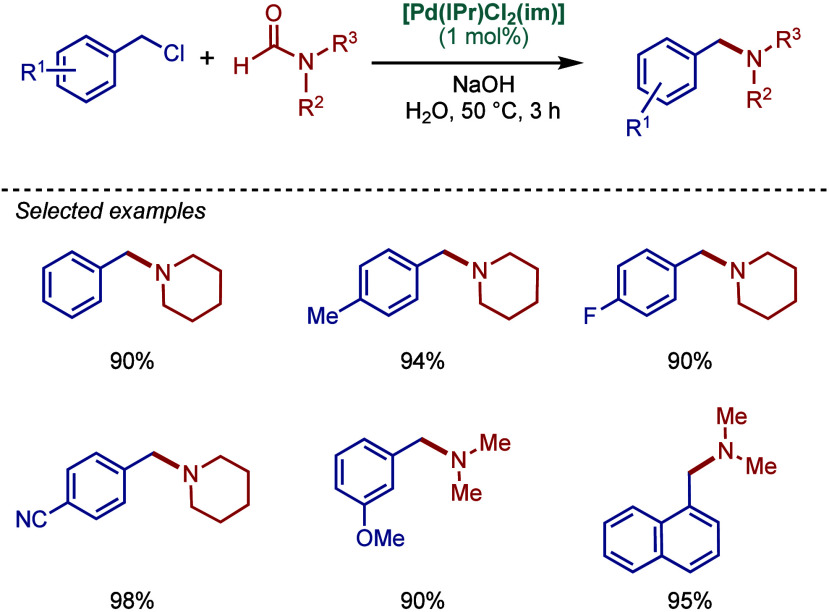
BHA Reaction Using Amides as Amine Source Catalyzed by [Pd­(IPr)­Cl_2_(Imidazole)] Complex by Lu

##### 2.3.10.3 Aliphatic Amines

In 2011, Navarro and co-workers reported [Pd–NHC] complexes containing triethylamine (TEA) as the ancillary ligand and evaluated their reactivity in BHA reaction (Scheme 111).^258^ These authors proposed that the use of triethylamine as a stabilizing ligand to Pd will show beneficial effects due to modest σ-donation of aliphatic amines and comparatively low steric demand. The desired [Pd­(NHC)­Cl_2_(TEA)] complexes (NHC = IPr, SIPr) were obtained by the reaction of [Pd­(NHC)­Cl_2_]_2_ dimers with an excess of TEA. X-ray analysis showed distorted square planar geometry with the Pd–C bond lengths of 1.968 Å and 1.970 Å for IPr and SIPr complexes, respectively. Evaluation of the catalytic activity of [Pd­(SIPr)­Cl_2_(TEA)] with the [SIPr–Pd–PEPPSI] congener and dimeric [Pd­(SIPr)­Cl_2_]_2_ showed higher efficiency of the TEA complex under the tested conditions (KO^*t*^Bu, DME, 50 °C). This complex was then applied to the BHA reaction of various aryl and heteroaryl chlorides with 1° and 2° amines, affording the amine products in high yields.

**Scheme 111:**
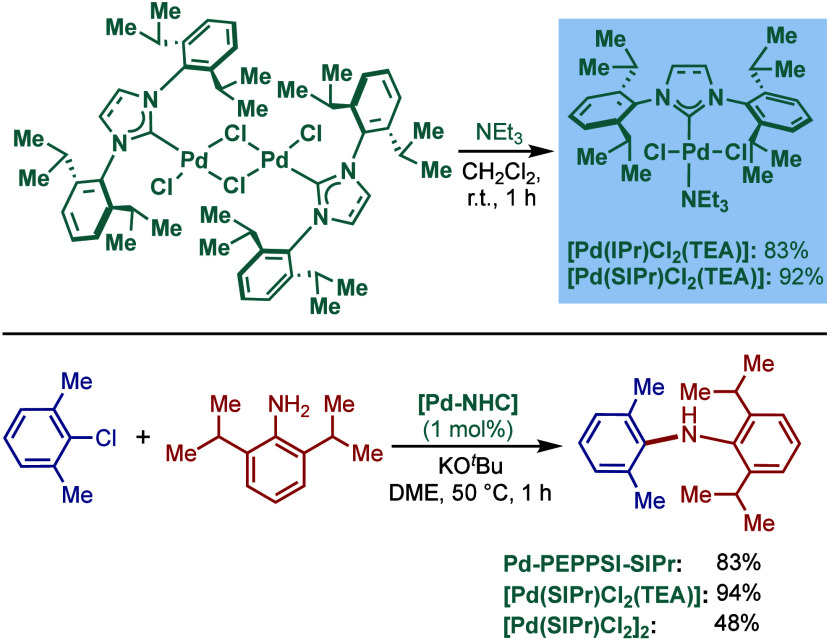
BHA Reaction Catalyzed by [Pd­(NHC)­Cl_2_(TEA)] Complexes by Navarro

In 2021 Organ and co-workers reported BHA reactions under eco-friendly conditions catalyzed by [Pd­(NHC)­Cl_2_(morpholine)] complexes in the presence of lipophilic sodium butylated hydroxytoluene (NaBHT) as the base (Scheme 112).^259^ The combination of the strong, lipophilic base with lipophilic palladium–NHC catalysts enabled to perform these reactions under solvent-free conditions. The authors found that the bulky *ortho*-wingtip substituents facilitate the reaction by diffusing the substrates, while protecting the catalyst from deactivation. Replacing KO^*t*^Bu with NaBHT base significantly increased the functional group tolerance of this method. These [Pd­(NHC)­Cl_2_(morpholine)] complexes (NHC = DiMeIHept^Cl^, IHept^Cl^, IPent^Cl^) showed high activity under mild conditions. However, it is worth noting that their cinnamyl analogs (see Section 2.3.1) were more reactive in some cases.

**Scheme 112:**
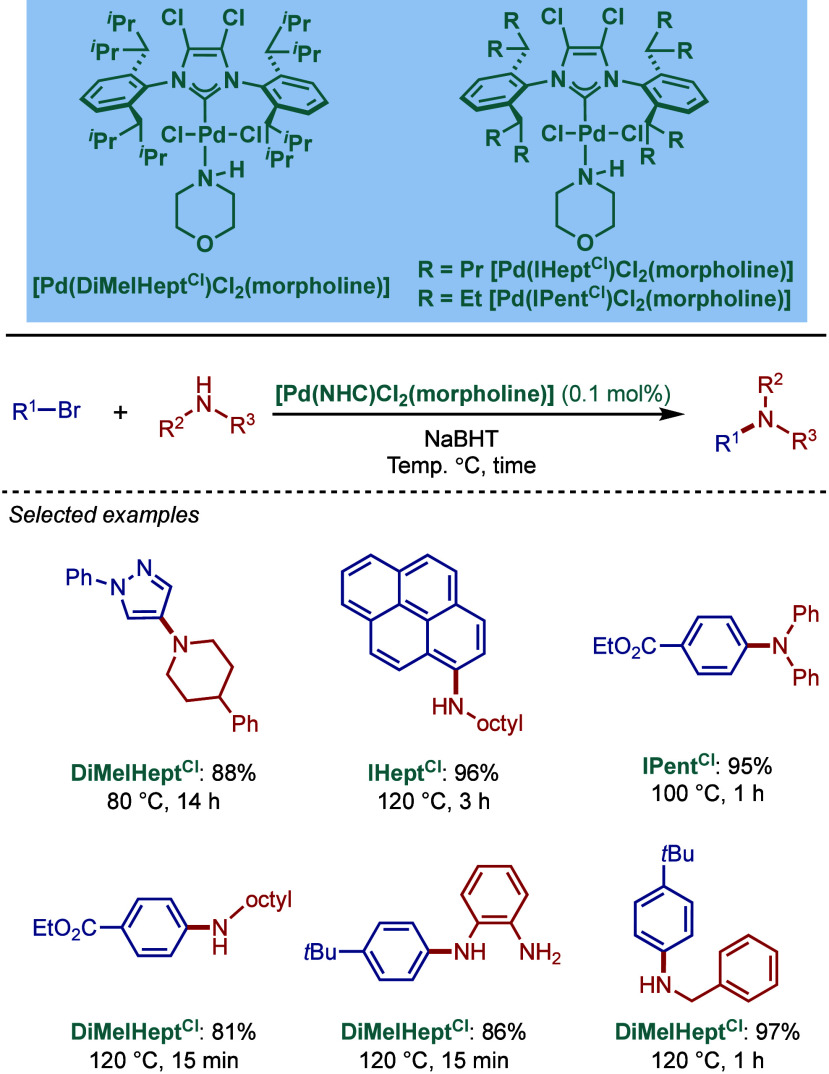
BHA Reaction in Melt Conditions Catalyzed by [Pd­(NHC)­Cl_2_(morpholine)] Complexes by Organ

In 2022, Chen and co-workers reported [Pd–BIAN–NHC] complexes with N-donor ancillary ligands and evaluated their activity in BHA reaction (Scheme 113).^260^ These precatalysts featured triethylamine (TEA) and N,N-dimethylbenzylamine (DMBA) donors and were prepared from the corresponding [Pd­(BIAN–NHC)­Cl_2_]_2_ dimer. X-ray crystallographic analysis showed distorted square planar geometry with two chloride ligands perpendicular to the carbene. The complex with DMBA ligand was evaluated in BHA reaction of aryl and heteroaryl chlorides with anilines, affording the coupling products in high yields. Furthermore, synthesis of air- and moisture-stable *trans*-[Pd­(NHC)­(NH_2_^*n*^Bu)­Cl_2_] precatalysts and their catalytic activity in BHA reactions were reported by Cazin and Nolan.^261^

**Scheme 113:**
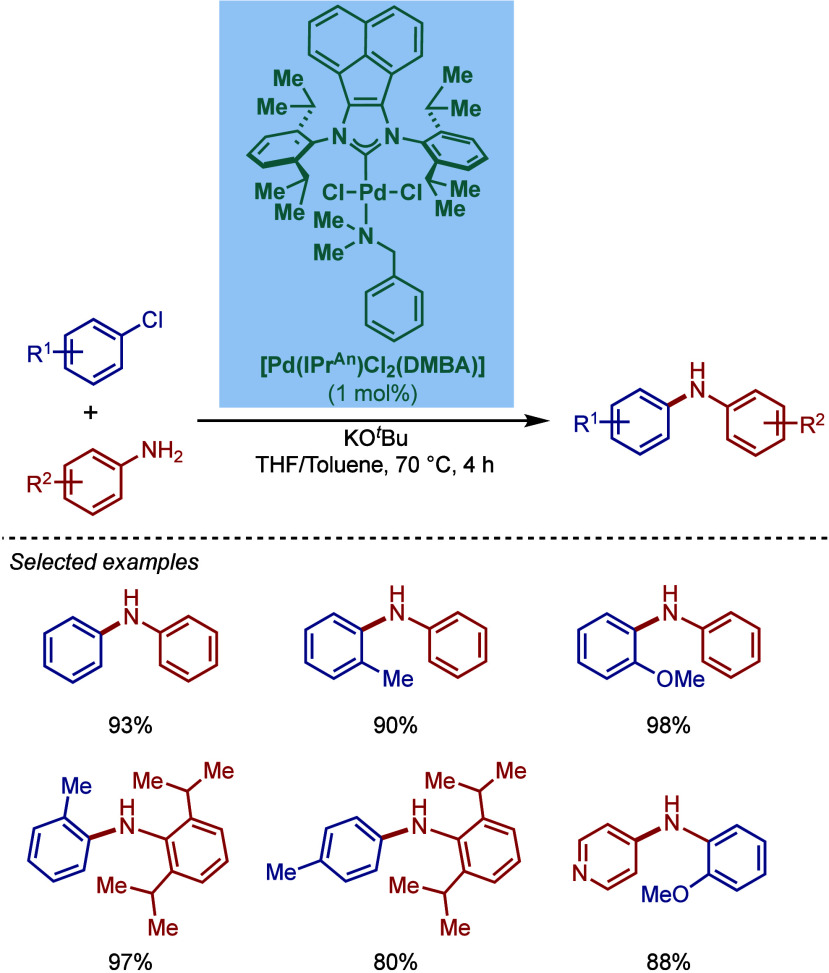
BHA Reaction Catalyzed by [Pd­(BIAN–IPr)­Cl_2_(DMBA)] Complex by Chen

##### 2.3.10.4 Aromatic Amines

The synthesis of [Pd–NHC] complexes with quinoline and isoquinoline ancillary ligands and their application in BHA reaction was reported by Lu and co-workers in 2016 was (Scheme 114).^262^ The desired [Pd­(NHC)­Cl_2_(quinoline)] and [Pd­(NHC)­Cl_2_(isoquinoline)] complexes (NHC = IPr, IMes, Xyl) were obtained in a one-step protocol from imidazolium salts, PdCl_2_, and quinoline/isoquinoline in the presence of K_2_CO_3_ in refluxing THF in 30–83% yields. The structure of the IPr-based complex, [Pd­(IPr)­Cl_2_(isoquinoline)] was determined by X-ray crystallography and showed shorter Pd–C and Pd–N bonds lengths than for the [IPr–PEPPSI] congener (1.960 Å and 2.093 Å vs 1.969 Å and 2.137 Å). This catalyst was applied to BHA reaction of aryl chlorides with 1° and 2° aryl and alkylamines at low catalyst loading (0.005–0.05 mol %) using KO^*t*^Bu in dioxane at 110 °C. of the [Pd­(IPr)­Cl_2_(isoquinoline)] complex led to arylated amines in high to excellent yields. Furthermore, evaluation of [Pd­(IPr)­Cl_2_(isoquinoline)] in comparison with other [Pd–NHC] complexes supported by N-containing ancillary ligands, such as 1-methylimidazole, morpholine, 2-phenyl-4,5-dihydrooxazole, and 3-chloropyridine, indicated that [Pd­(IPr)­Cl_2_(isoquinoline)] shows the highest efficiency.

**Scheme 114:**
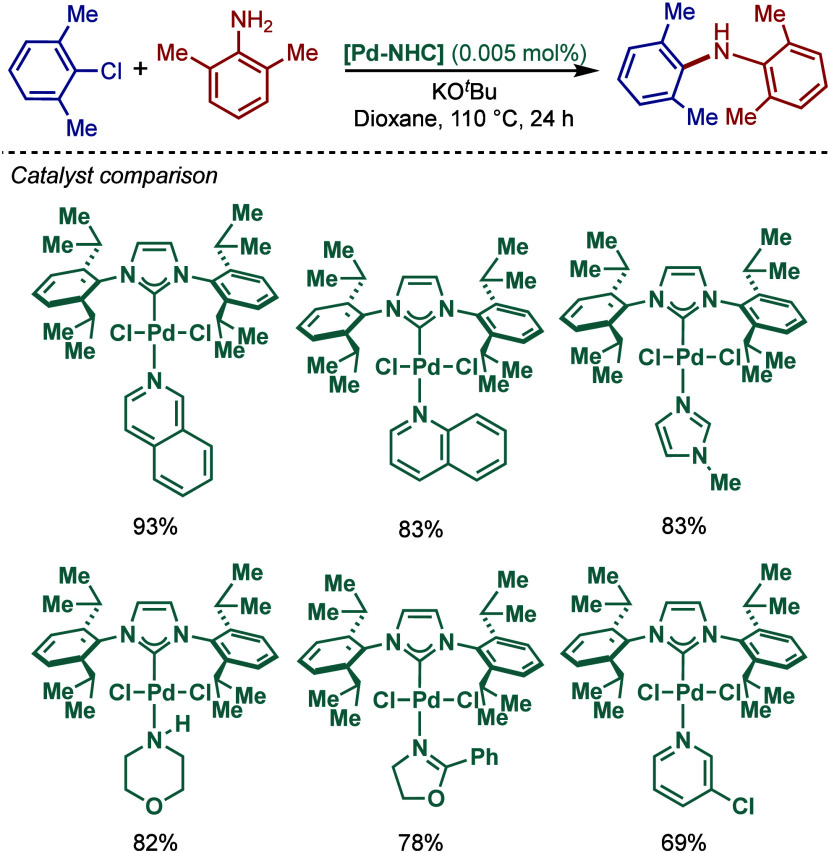
BHA Reaction Catalyzed by [Pd­(IPr)­Cl_2_(isoquinoline)] Complex by Lu

In 2021, Szostak and co-workers reported a new class of highly active [Pd­(NHC)­Cl_2_(AN)] complexes (AN = aniline) and evaluated their activity in BHA reaction (Scheme 115).^263^ Complexes [Pd­(IPr)­Cl_2_(AN)] and [Pd­(SIPr)­Cl_2_(AN)], featuring imidazol-2-ylidene and imidazolin-2-ylidene IPr and SIPr ligands were characterized by X-ray analysis, showing Pd–C and Pd–N bond lengths of 1.970 Å, 1.967 Å and 2.109 Å, 2.116 Å, respectively. The %buried volume was determined as 36.1% and 40.7%, which is larger than for the corresponding [Pd–PEPPSI] complexes (34.8% and 39.2%, respectively). These catalysts were evaluated in BHA reaction of 4-chloroanisole with morpholine, affording the coupling product in 98% yield. Furthermore, a related [Pd­(IPr)­Cl_2_(3-CF_3_-AN)] showed high activity in C–N activation, outperforming [Pd–PEPPSI–IPr] complex.^197–199^

**Scheme 115:**
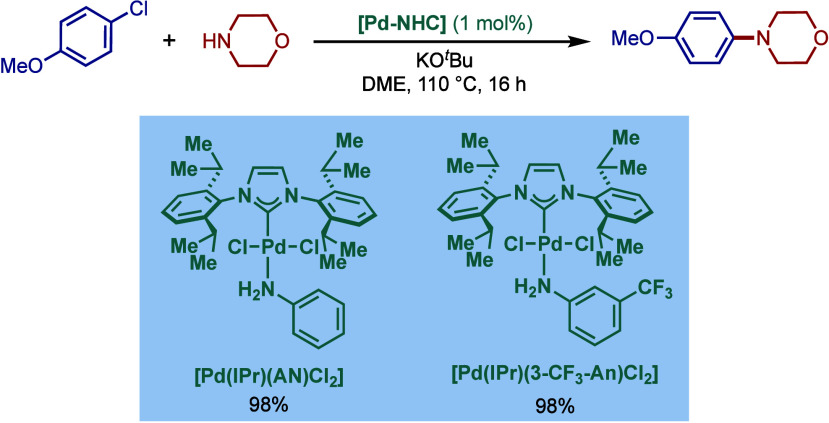
BHA Reaction Catalyzed by [Pd­(IPr)­Cl_2_(AN)] Complexes by Szostak

In 2022, Chen and co-workers reported a further application of these [Pd­(NHC)­Cl_2_(AN)] complexes in BHA reaction (Scheme 116).^264^ Precatalysts containing 2,6-diisopropylaniline, 2,4,6-trimethylaniline, 2,6-dimethylaniline, aniline, and ethylamine were prepared by the reaction with [Pd­(IPr)­Cl_2_]_2_. X-ray crystallographic analysis of the model complex with 2,6-diisopropylaniline ligand showed that aryl N-wingtips and the aniline ring are tilted due to steric repulsion of the isopropyl groups. This complex showed the highest activity in the BHA reaction of 2,6-dimethylchlorobenzene using KO^*t*^Bu in THF at 70 °C. Under the optimized reaction conditions, various aryl chlorides underwent BHA reaction with anilines and 2° aliphatic amines in high yields.

**Scheme 116:**
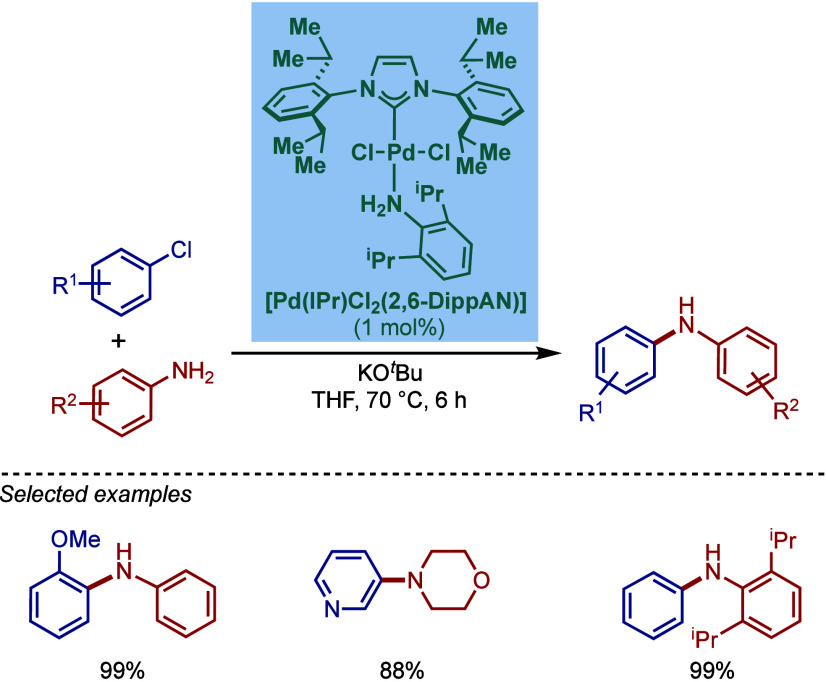
BHA Reaction Catalyzed by [Pd­(IPr)­Cl_2_(2,6-DippAN)] Complex by Chen

In 2023, Mansoori and co-workers reported a [bis­(NHC)–Pd] complex with intramolecular 2-pyridyl coordination supported on magnetic mesoporous silica and evaluated its activity in BHA reaction of aryl halides with ammonia (not shown).^265^

In 2024, Kaloğlu and co-workers reported the synthesis of [Pd–NHC] complexes with flexible N-aliphatic wingtips supported by N-heterocyclic ancillary ligands and evaluated their activity in BHA reaction (Scheme 117).^266^ Complexes of the imidazolin-2-ylidene scaffold with pyridine, 1-methylimidazole, 4,5-dimethylthiazole and 3-bromoquinoline ligands were synthesized. The catalytic activity was tested in the model BHA reaction of chlorobenzene with morpholine using KO^*t*^Bu in dioxane at 80 °C. The catalyst containing a symmetrical 4,5-dihydro-imidazole-2-ylidene scaffold and pyridine ancillary ligand showed the highest activity. Interestingly, Pd­(PPh_3_)_4_ and PdCl_2_ were also evaluated under the same conditions and found that these catalyst gave much lower yields (68% and 24%) compared to wingtip-flexible Pd–NHCs (80–97%).

**Scheme 117:**
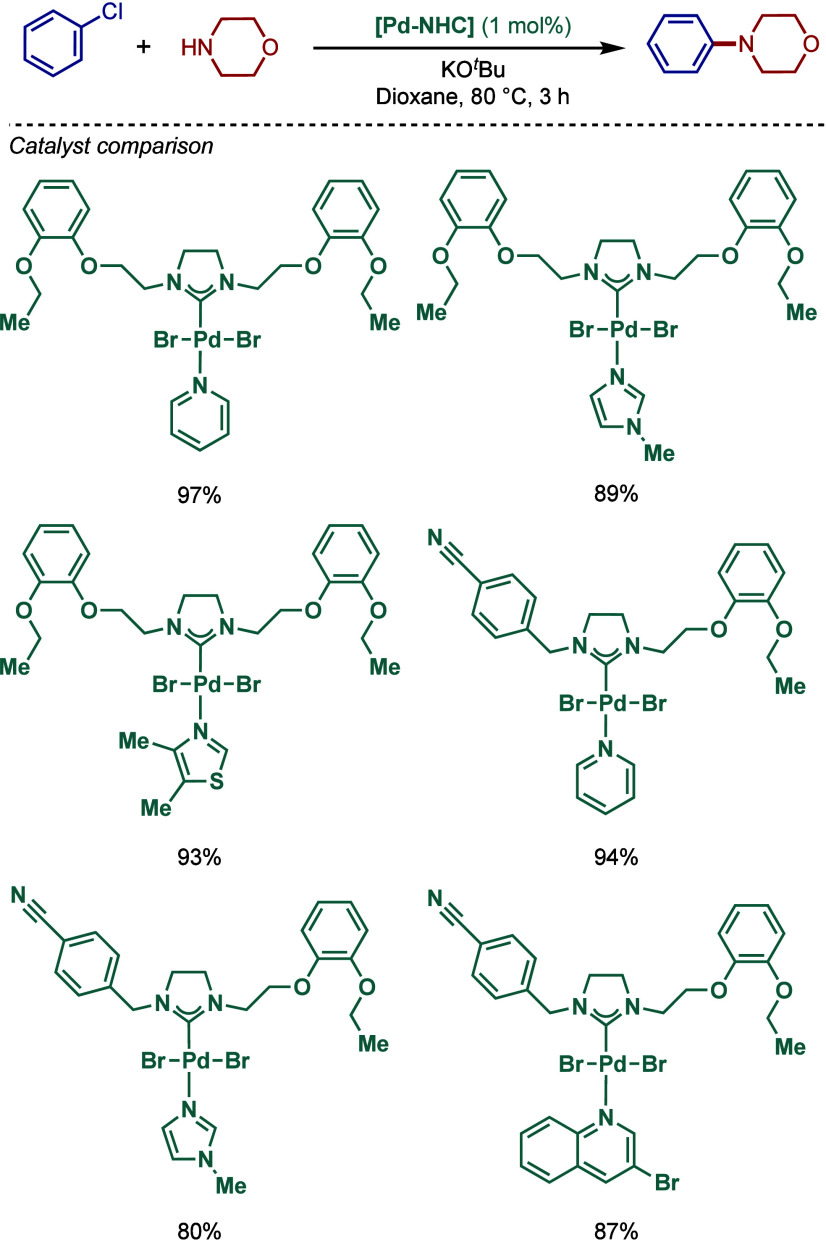
BHA Reaction Catalyzed by Wingtip N-Aliphatic [Pd–NHC] Complexes by Kaloğlu

##### 2.3.10.5 Indazole

The promising results obtained with [Pd­(NHC)­Cl_2_(oxazole)] complexes (see Section 2.3.10.1), inspired Lu and co-workers to study other heterocycles as supporting ligands. In 2016, they reported a series of complexes containing 1-methylindazole and 1-methylpyrazole (NHC = IPr, IMes, IXyl) and evaluated their activity in BHA reaction (Scheme 118).^267^ X-ray crystallographic analysis showed distorted square planar geometry around palladium with Pd–C and Pd–N bond lengths in the range of 1.968–1.972 Å and 2.086–2.093 Å, respectively. The highest catalytic activity was found for the [Pd­(IPr)­Cl_2_(1-methylpyrazole)] complex (87% yield), which outperformed [Pd­(IPr)­Cl_2_(1-methylindazole)] (67% yield). Complexes featuring IMes and IXyl ligands afforded the coupling products in low yields. This new [Pd­(IPr)­Cl_2_(1-methylpyrazole)] catalyst was successfully applied to BHA reaction of aryl chlorides with 1° and 2° amines.

**Scheme 118:**
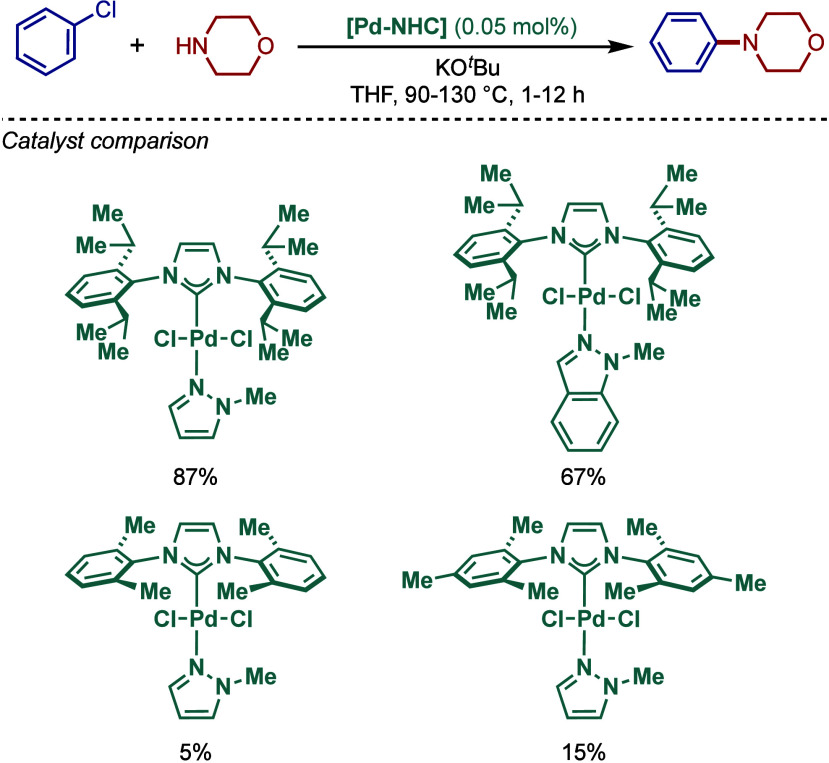
BHA Reaction Catalyzed by [Pd­(NHC)­Cl_2_(pyrazole)] and [Pd­(NHC)­Cl_2_(indazole)]­Complexes by Lu

In 2017, Yang reported related [Pd–NHC] complexes featuring N-unsubstituted pyrazole and indazole as ancillary ligands and evaluated their activity in BHA reaction (Scheme 119).^268^ These [Pd­(NHC)­Cl_2_(NH–azole)] complexes (NHC = IPr, SIPr, IMes, SIMes) were synthesized from dimeric [Pd­(NHC)­Cl_2_]_2_ (cf. complexation with PdCl_2_/K_2_CO_3_ by Lu). Characterization by X-ray crystallography showed that the NHC plane was tilted from the palladium coordination plane (dihedral angle = 69.76–90.0°), while NHC ring was perpendicular to the azole unit. Selected complexes were tested in BHA reaction of chlorobenzene with 4-methoxyaniline, showing higher activity than the parent [Pd–PEPPSI–IPr] congener and the corresponding dimer [Pd­(IPr)­Cl_2_]_2_. These [Pd­(NHC)­Cl_2_(azole)] complexes showed high activity BHA reaction of aryl chlorides using KO^*t*^Bu in toluene at 110 °C with 0.1 mol % catalyst loading.

**Scheme 119:**
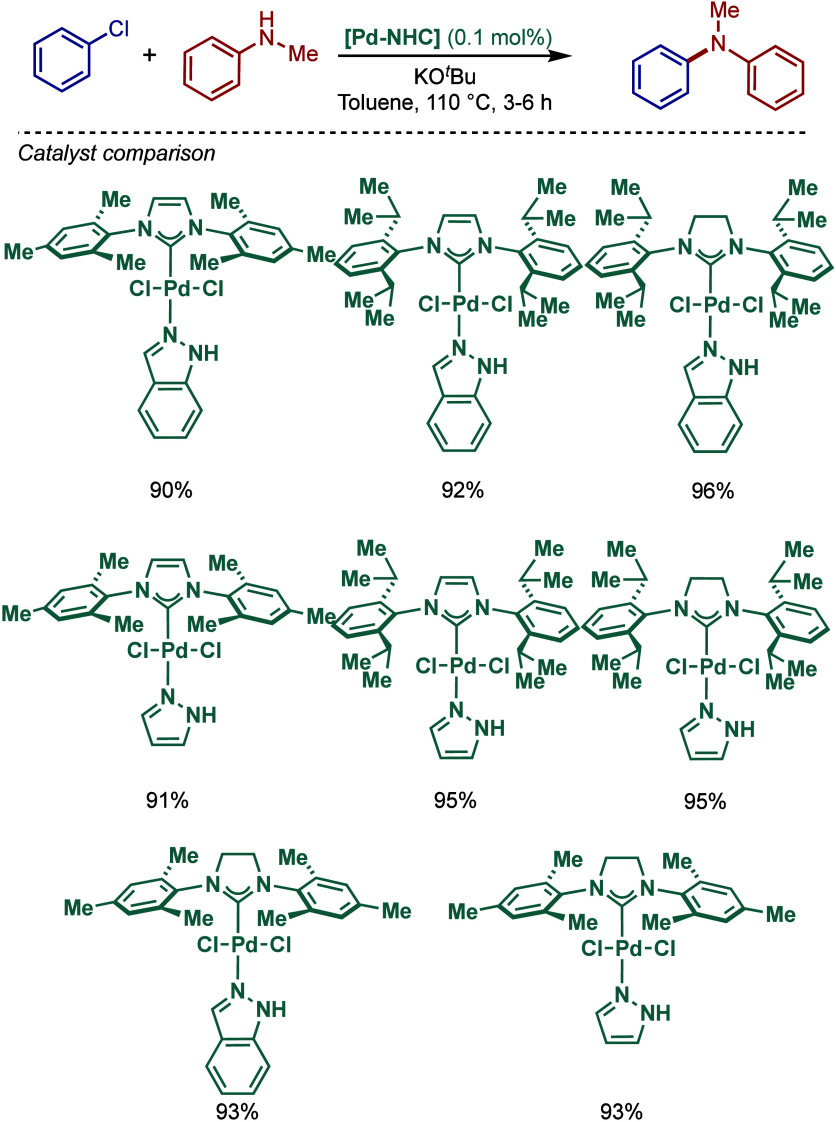
BHA Reaction Catalyzed by [Pd­(NHC)­Cl_2_(NH–azole)] Complexes by Yang

##### 2.3.10.6 Benzoxazole/Benzothiazole

In 2016, Liu and Zhao reported [Pd–NHC] complexes with benzoxazole and benzothiazole ancillary ligands (NHC = IPr, IMes) and evaluated their activity in BHA reaction (Scheme 120).^269,270^ These air- and moisture-stable complexes were prepared by complexation with PdCl_2_ in the presence of K_2_CO_3_ in THF at 65 °C in 65–76% yields. All complexes were characterized by X-ray crystallography and showed distorted square planar geometry with Pd–C and Pd–N bond lengths in the range of 1.953–1.974 Å and 2.088–2.100 Å, respectively. Comparison of catalytic activity in the model BHA reaction of chlorobenzene with morpholine using KO^*t*^Bu in toluene at 110 °C identified [Pd­(IPr)­Cl_2_(benzoxazole)] as the most effective complex. This catalyst was further applied in the BHA reaction of chlorobenzenes with various 1° anilines and 2° aliphatic amines.

**Scheme 120:**
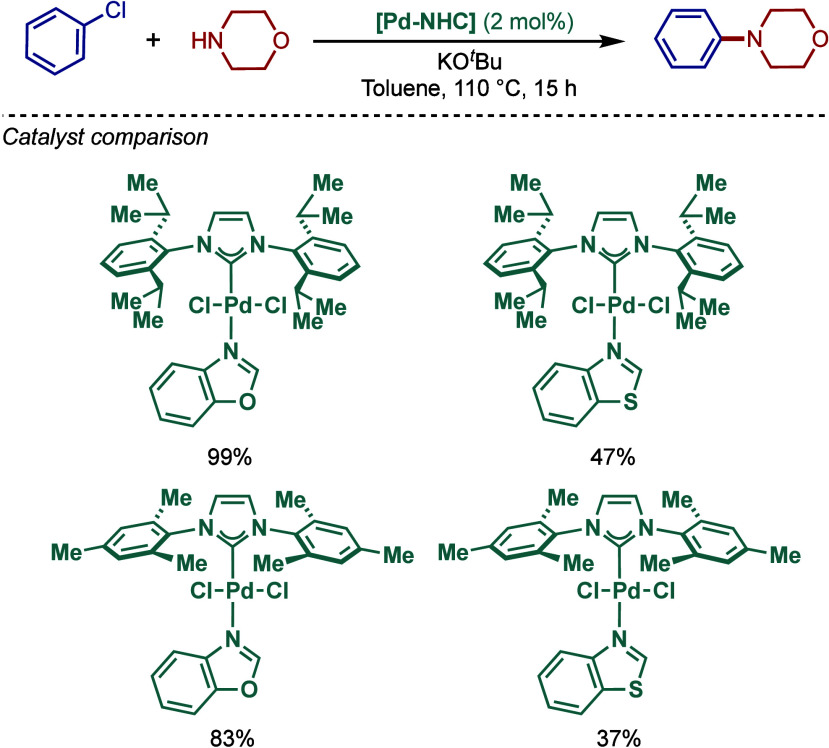
BHA Reaction Catalyzed by [Pd­(NHC)­Cl_2_(benzoxazole)] and [Pd­(NHC)­Cl_2_(benzothiazole)] Complexes by Liu and Zhao

#### 2.3.11 [Pd­(NHC)­(O,N)­Cl] Complexes

In 2010, Jin and Fang reported the synthesis of well-defined [Pd­(NHC)­(sal)­Cl] complexes (sal = salicylaldimine) and their application in BHA reaction (Scheme 121).^271^ These Pd­(II)–NHC complexes were efficiently prepared by complexation of [Pd­(NHC)­Cl_2_]_2_ dimers (NHC = IPr, SIPr) with salicylaldimine ligands in the presence of Cs_2_CO_3_ in dioxane at 100 °C. Moreover, a salicylaldehyde congener was synthesized by the same method. X-ray crystallographic analysis showed distorted square-planar geometry around Pd and twisted salicylaldimine fragments. The N–Pd bond lengths were in a range of 2.046–2.079 Å, and the C–Pd bond lengths were 1.976–1.991 Å. The catalytic activity was evaluated in BHA reaction using 4-chlorotoluene and morpholine as model substrates using NaO^*t*^Bu in DME at 80 °C. Complexes bearing the N-phenyl group on the salicylaldimine moiety gave the product in 86% (SIPr) and 73% (IPr) yield. In the case of more N-sterically substituted complexes (Mes, 2,6-Dipp), lower conversions were observed. The authors identified imidazolin-2ylidene N-3,5-(CF_3_)_2_-C_6_H_3_-derivative as the most reactive catalyst in the series, resulting in quantitative conversion. This complex was then used to catalyze the amination of aryl chlorides with 1° and 2° amines under aerobic conditions. The authors proposed that the weaker Pd–N bond in these [Pd­(NHC)­(sal)­Cl] complexes permit for a faster activation to give the active, monoligated Pd(0)–NHC species.

**Scheme 121:**
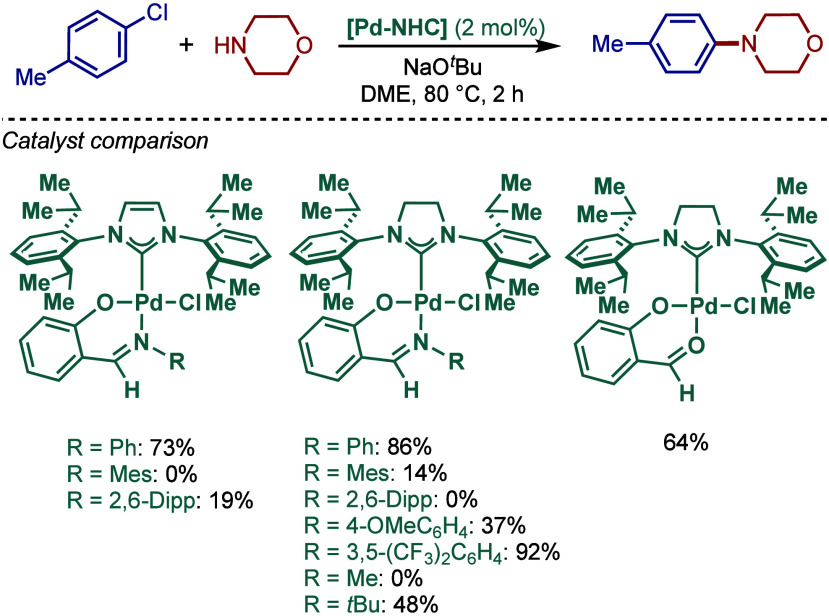
BHA Reaction Catalyzed by [Pd­(NHC)­(sal)­Cl] Complexes by Jin and Fang

In 2013 Xu and Jin reported a new class of [N,O]- and [O,N,O]-chelated [Pd­(II)–NHC] complexes (NHC = IPr, SIPr) using pyridine-2-carboxylate (pyc) and pyridine-2,6-dicarboxylate (pydc) as ancillary ligands (Scheme 122).^272^ These complexes were synthesized by a direct complexation of PdCl_2_ with imidazolium salt in the presence of Cs_2_CO_3_ or by a ligand displacement from [Pd­(NHC)­Cl_2_]_2_ dimers. In analogy to [Pd–PEPPSI] complexes, the pyridine nitrogen atom was located *trans* to the carbene ligand in [N,O]-chelated bidentate complexes, while the carboxylate oxygen was in the *cis*-position. In the case of [O,N,O]-chelated tridentate complexes, a *trans*-chelating planar configuration was determined. Interestingly, [Pd­(NHC)­(pydc)] was characterized by longer Pd–C bond lengths (IPr: 2.005 Å; SIPr: 1.996 Å) than in [Pd­(NHC)­(pyc)­Cl] complexes (IPr: 1.963 Å; SIPr: 1.970 Å) and in [Pd–PEPPSI] complexes (IPr: 1.955 Å; SIPr: 1.962 Å). [Pd­(IPr)­(pydc)­Cl] showed the highest efficiency in the model BHA reaction of mesityl chloride with morpholine using NaO^*t*^Bu in dioxane at 50 and 100 °C. This complex was next used to catalyzed BHA reaction of sterically hindered aryl chlorides with 1° and 2° aliphatic amines.

**Scheme 122:**
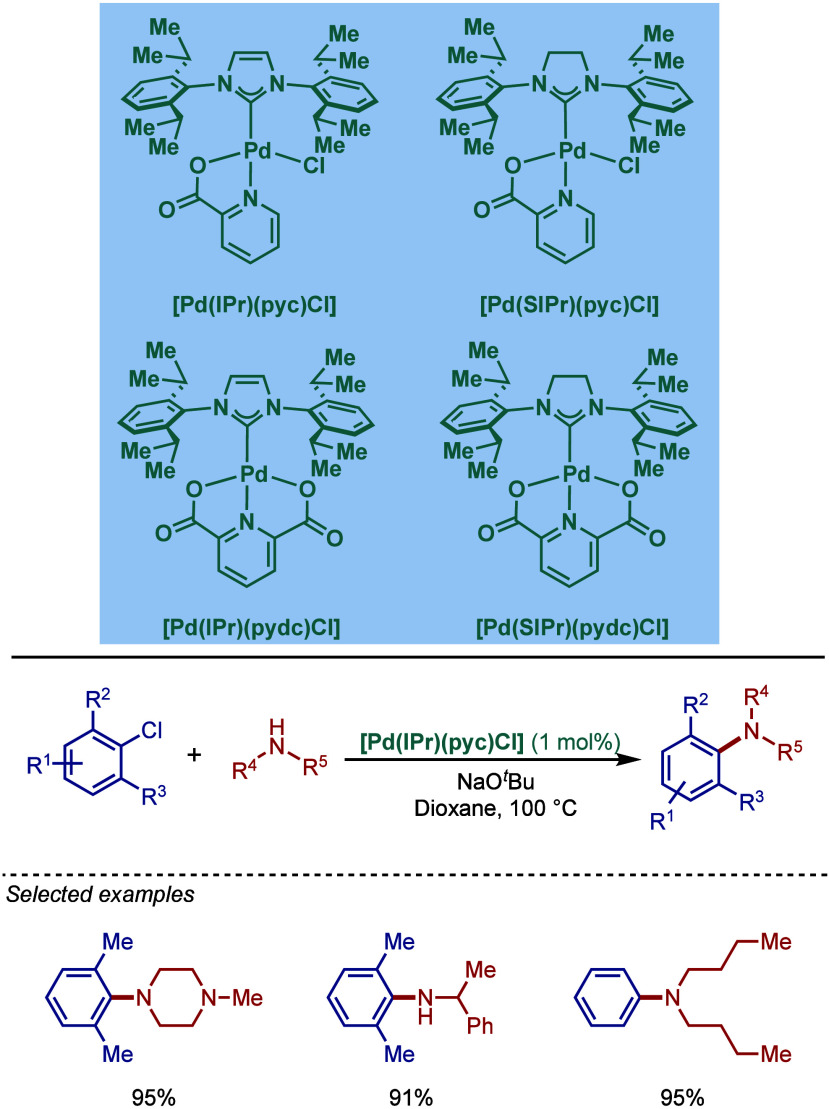
BHA Reaction Catalyzed by [Pd­(NHC)­(pyc)­Cl] and [Pd­(NHC)­(pydc)­Cl] Complexes by Xu and Jin

In 2017, Lu and co-workers reported the same class of [Pd­(NHC)­(pyc)­Cl] complexes bearing pyridine-2-carboxylate as the ancillary ligand (NHC = IPr, Mes, Xyl) (not shown).^273^ Catalytic activity was evaluated in BHA reaction of chlorobenzene and morpholine in the presence of KO^*t*^Bu in toluene at 90 °C, where [Pd­(IPr)­(pyc)­Cl] significantly outperformed its Mes and Xyl congeners (98% vs <10% yield).

#### 2.3.12 [Pd­(NHC)_2_Cl_2_] Complexes

Özdemir and co-workers reported a series of studies presenting a novel class of [Pd­(NHC)_2_Cl_2_] complexes and evaluated their activity in BHA reaction (Scheme 123).^274–277^ The authors described two general procedures for the complex synthesis, namely the direct complexation of imidazolium salts with Pd­(II) and transmetalation of the corresponding [Ag­(I)–NHC] complexes with [PdCl_2_(CH_3_CN)_2_]. X-ray crystallographic analysis showed the coordination of two NHCs to Pd with the two NHC ligands and two halide ligands in the *trans-*position. These complexes were tested in BHA reaction of bromobenzene and various aniline derivatives, leading to the triarylamine and diarylamine products in high yields. The complexes were also shown to be effective in the amination with cyclic aliphatic amines with different ring sizes.

**Scheme 123:**
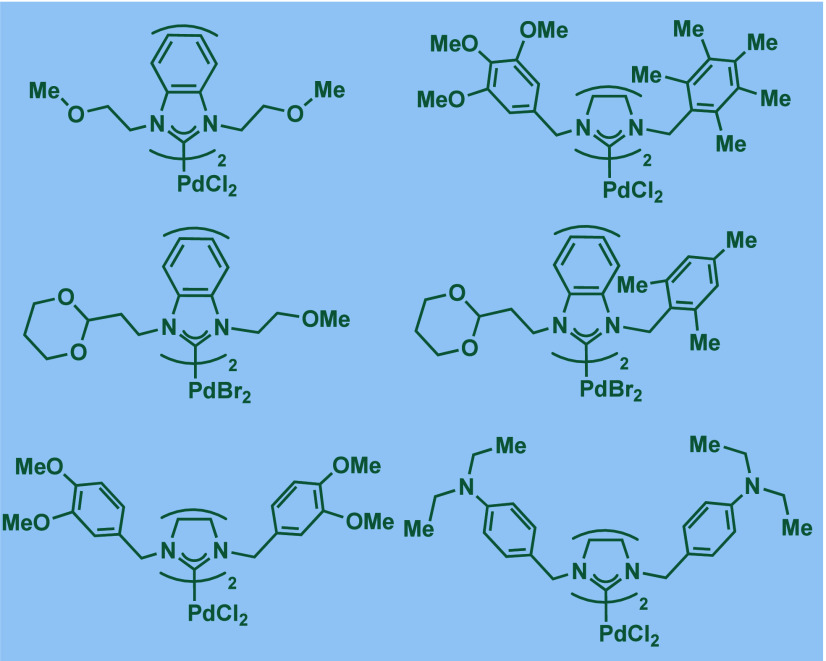
[Pd­(NHC)_2_X_2_] (X = Cl or Br) Complexes by Özdemir

In 2011, Özdemir and co-workers reported the synthesis of well-defined *ortho*-xylyl-linked [Pd­(NHC)_2_Cl_2_] complexes and evaluated their activity in BHA reaction (Scheme 124).^278^ This class of precatalysts was synthesized from the readily available *o*-xylyl-*bis*-benzimidazolium chloride by the direct complexation with Pd­(II). These complexes were found to be air- and moisture-stable with the carbenic carbon in the range of 173.5–175.7 ppm in ^13^C spectra. Crystallographic analysis of the complex bearing N-pentamethylbenzyl wingtip showed minor distortion from square planar geometry and *cis*-coordination of the two carbene ligands. The distortion around the metal was a result of a sterically restricted 9-membered ring in the presence of an *o*-xylyl bridge. The activity of these complexes was tested in BHA reaction of bromobenzene and various amines, leading to the desired products in high yields.

**Scheme 124:**
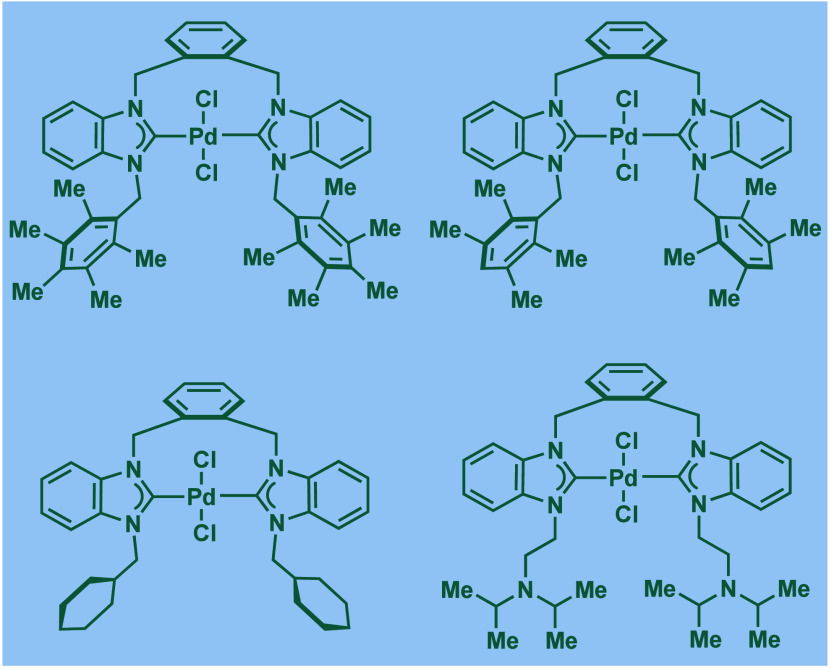
Structures of *o*-Xylyl-Linked [Pd­(NHC)_2_Cl_2_] Complexes by Özdemir

#### 2.3.13 [Pd­(NHC)­(SR_2_)­Cl_2_] Complexes

In 2022, Cazin, Nolan, and co-workers reported the synthesis of *trans*-[Pd­(NHC)­Cl_2_(SR_2_)] complexes (SR_2_ = DMS, THT; DMS = dimethyl sulfide; THT = tetrahydrothiophene) and evaluated their activity in BHA reaction (Scheme 125).^279^ These air- and bench-stable precatalysts were prepared by complexation of the imidazolium salts with *trans*-[PdCl_2_(DMS/THT)_2_] precursors in the presence of K_2_CO_3_ in acetone at 40 °C in excellent yields (78–97%) (NHC = IPr, SIPr, IMes, IPr^Cl^, IPr*, IPr^#^). The X-ray crystallography showed distorted square-planar geometry around palladium. The lengths of Pd–C bonds were in a range of 1.982–2.001 Å and the Pd–S bond in a range of 2.350–2.379 Å. The complexes were evaluated in BHA reaction of 4-chloroanisole with morpholine in the presence of KO^*t*^Bu in 2-MeTHF at 80 °C. The catalytic activity of these [Pd­(NHC)­Cl_2_(SR_2_)] complexes superseded the parent [Pd–PEPPSI–IPr] (up to 98% vs 88%). Precatalysts featuring sterically demanding IPr* and IPr^#^ ligands showed the highest efficiency, which was ascribed to an acceleration of the reductive elimination step. The sterically demanding [Pd­(IPr^#^)­Cl_2_(DMS)] was selected for amination of aryl chlorides with 1° and 2° aliphatic and aromatic amines, giving products in good to excellent yields.

**Scheme 125:**
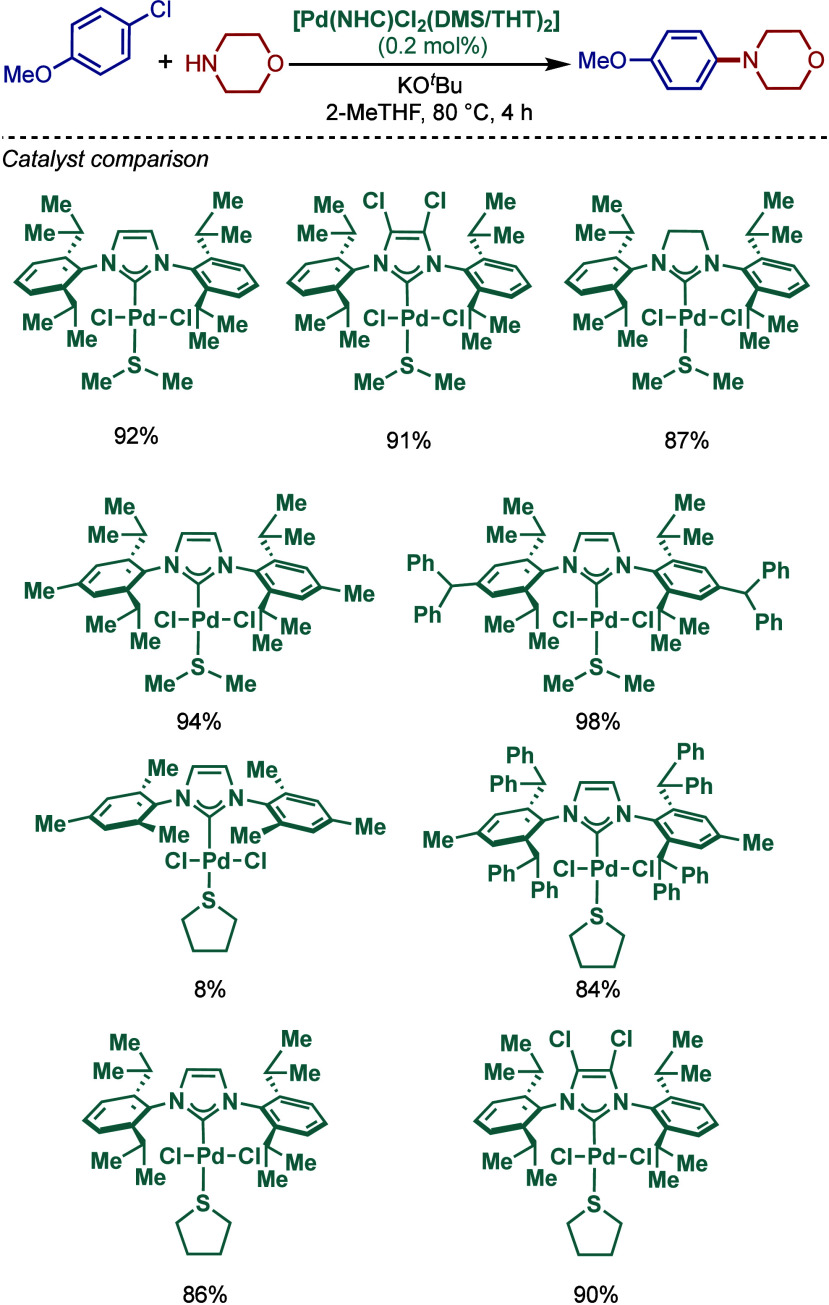
BHA Reaction Catalyzed by [Pd­(NHC)­Cl_2_(DMS/THT)_2_] Complexes by Cazin and Nolan

#### 2.3.14 [Pd­(NHC)­(MR_3_)­Cl_2_] (M = As, Sb) Complexes

In 2014, Wang and co-workers reported [Pd–NHC] complexes bearing arsine and stibine as ancillary ligands (Scheme 126).^280^ A series of complexes bearing IPr, SIPr, IMes and SIMes ligands were obtained by ligand displacement from the corresponding chloro-bridged dimers, [Pd­(NHC)­Cl]_2_, with AsPh_3_ or SbPh_3_ in 87–93% yields. In analogy to mixed Pd–NHC/phosphine complexes, AsR_3_ and SbR_3_ ligands were *trans*-positioned with respect to the carbene ligands. The Pd–As bond lengths were in a range of 2.410–2.468 Å, and Pd–Sb bond lengths were in a range of 2.581–2.594 Å, which is longer than for the corresponding Pd–N and Pd–P bond lengths in [Pd­(NHC)­Cl_2_(NR_3_)] complexes (Pd–N, 2.219 Å, [Pd­(IPr)­Cl_2_(NEt_3_)]) and [Pd­(NHC)­Cl_2_(NR_3_)] complexes (Pd–P, 2.305 Å, [Pd­(IPr)­Cl_2_(PPh_3_)]). The catalytic activity was evaluated in BHA reaction of aryl chlorides with anilines using KO^*t*^Bu in dioxane at 100 °C, showing activity similar to that of the phosphine-functionalized complex, [Pd­(SIMes)­Cl_2_(PPh_3_)].

**Scheme 126:**
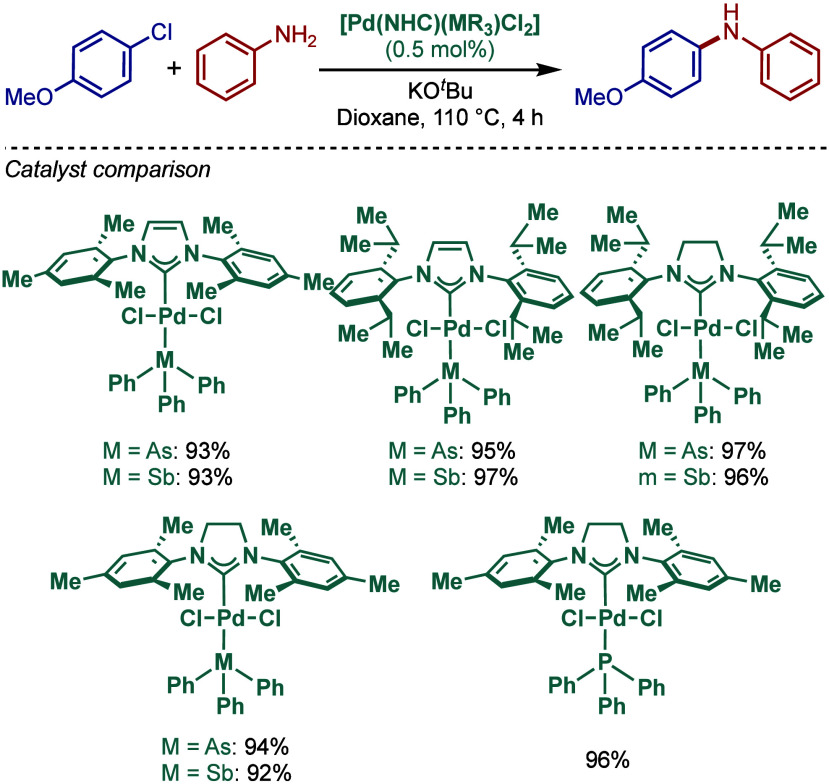
BHA Reaction Catalyzed by As- and Sb-Functionalized [Pd­(NHC)­(MR_3_)­Cl_2_] Complexes by Wang

#### 2.3.15 [Pd­(NHC–MIC)­X_2_] Complexes

In 2016, Mendoza-Espinosa and co-workers reported the synthesis of [Pd–MIC] complexes bearing triazol-5-ylidene carbene ligands with phenoxymethylene wingtips and evaluated their activity in BHA reaction (Scheme 127).^281^ Mono- and bis-mesoionic carbene (MIC) palladium complexes were obtained by direct complexation of triazolium salts with Pd­(II) by controlling the stoichiometry of palladium precursor (0.48 equiv. vs 1.1 equiv), leading to a mixture of *cis*/*trans* isomers of [Pd­(MIC)_2_I_2_] or bridged dimers, [Pd­(MIC)­I_2_]_2_. Furthermore, PEPPSI-type MIC complexes were synthesized from triazolium salts in the presence of PdCl_2_ and K_2_CO_3_ in pyridine at 100 °C. The authors found that the steric hindrance of the C4-substituent had a key influence on *cis*:*trans* isomer ratio. For example, the sterically flexible C4-benzyl substituent resulted in a ratio of 1:1, while the sterically demanding C4-mesityl resulted in 1:4 ration. X-ray analysis of the [Pd–PEPPSI–MIC] complex showed an *anti*-arrangement of the phenoxy and C4-benzylic groups with respect to the triazol-5-ylidene ring. Catalytic performance was evaluated in BHA reaction of bromobenzene and morpholine using KO^*t*^Bu in dioxane or DMF. The highest reactivity was observed for [Pd–PEPPSI–MIC] complexes, which resulted in 77–79% conversion at room temperature. These complexes were next used in BHA reaction of aryl bromides and chlorides with 1° and 2° aromatic and 2° aliphatic amines, leading to products in high yields.

**Scheme 127:**
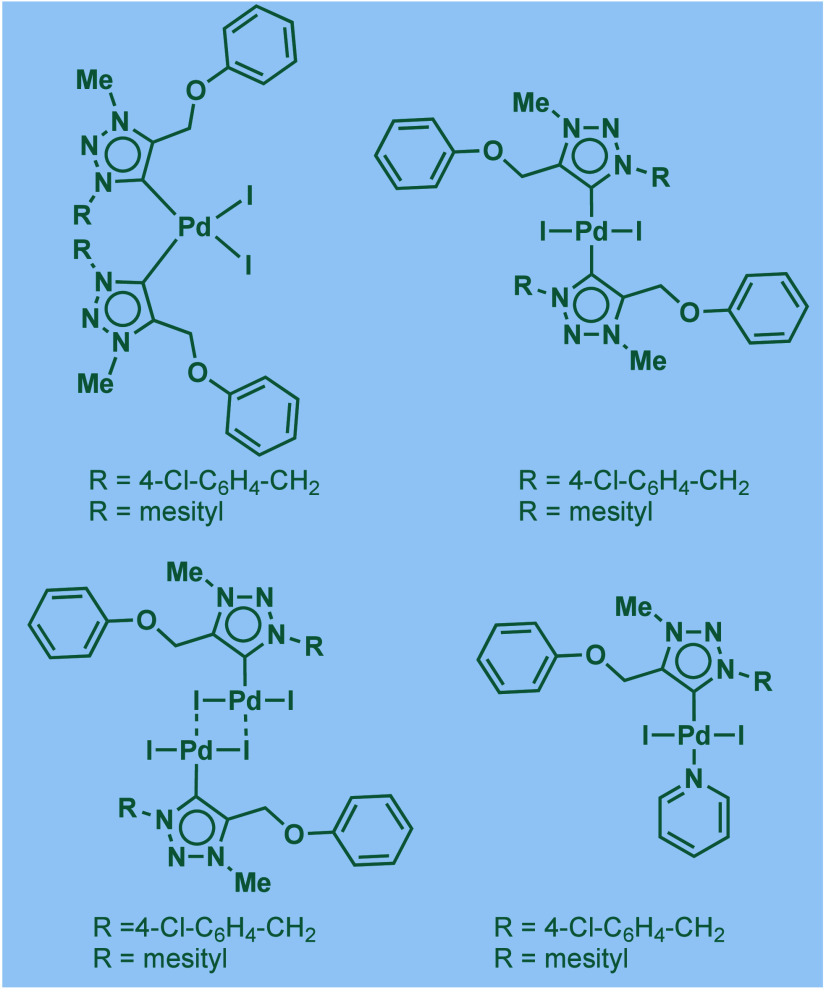
Structures of Well-Defined [Pd–MIC] Complexes by Mendoza-Espinoza

### 2.4 Well-Defined [Pd­(I)–NHC] Complexes

#### 2.4.1 [Pd­(NHC)­(μ-X)]_2_ Complexes

In 2019, Gooßen and co-workers reported a halogen-bridged [Pd­(I)–NHC] complex, [Pd­(IPr)­I]_2_, and evaluated its activity in BHA reaction (Scheme 128).^282^ This di-iodo-bridged Pd­(I) dimer was obtained by the reduction reaction of [Pd­(IPr)­I_2_]_2_ in a basic methanol solution (KOH/MeOH) in toluene at room temperature at 60% yield. It is worth noting that the synthesis of the corresponding bromo-dimer was unsuccessful, leading to a Pd­(II)–H species, [Pd­(IPr)_2_(H)­Br]. The structure was confirmed by X-ray analysis, showing C_2_-symmetry (two molecules in the unit cell) with an average Pd–I bond length of 2.601 Å, which is like that in [(*t*Bu_3_P)­PdI]_2_ (2.598 Å). This [Pd­(IPr)­I]_2_ complex was shown to promote BHA reaction of 4-chlorotoluene with morpholine in the presence of NaO^*t*^Bu in THF at room temperature at 0.5 mol % catalyst loading. The substrate scope was evaluated in the amination of aryl chlorides with anilines using KO^*t*^Bu in THF at 40 °C, where this catalyst showed high reactivity.

**Scheme 128:**
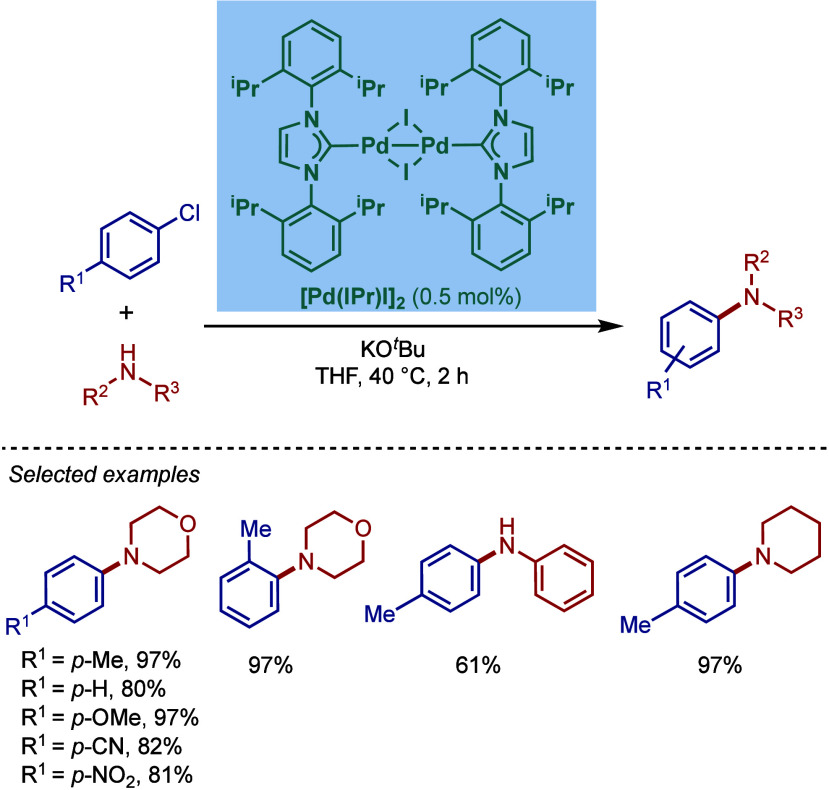
BHA Reaction Catalyzed by [Pd­(IPr)­I]_2_ by Gooßen

### 2.5 BHA Reaction of Pseudohalides

The first example of the use of a [Pd–NHC] system for BHA reaction of aryl tosylates was reported by César and co-workers in 2015 (Scheme 129).^283^ These authors evaluated a series of [Pd–PEPPSI]-type complexes bearing sterically hindered imidazol-2-ylidene ligands, such as IPr, IPr^Cl^, IPr^NMe2^, IPr^(NMe2)2^, IPent, and IPent^Cl^, in the model coupling reaction of 4-tolyl and 4-methoxybenzene tosylate with morpholine in the presence of K_3_PO_4_ in *t*AmOH at 120 °C, leading to conversions up to 99%. The order of activity was found to be as follows: IPr^(NMe2)2^ > IPent ≈ IPent^Cl^ > IPr^NMe2^ > IPr ≈ IPr^Cl^, with the most powerful precatalyst [Pd–PEPPSI–IPr^(NMe2)2^] permitting the coupling of deactivated 4-methoxybenzene tosylate in 43% yield. Under the optimized conditions, this catalyst was applied to BHA reaction with various 1° and 2° aliphatic and aromatic amines. Moreover, chemoselective sequential, one-pot bis-aminations were developed, capitalizing on the differential reactivity of aryl chlorides and aryl tosylates in a good overall yield.

**Scheme 129:**
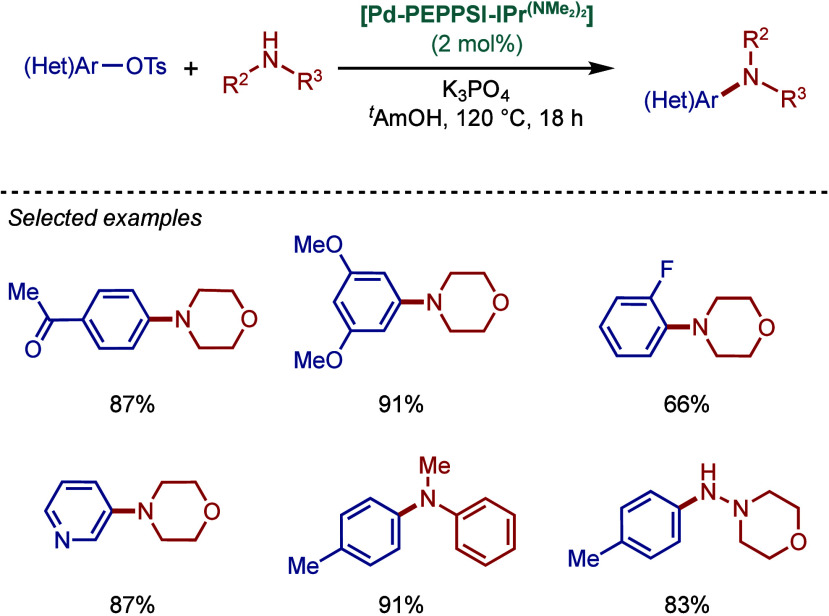
BHA Reaction of Tosylates Catalyzed by [Pd–PEPPSI–IPr^(NMe2)2^] by César

In 2018, Duan and co-workers reported the synthesis and catalytic activity in BHA reaction of a [Pd–PEPPSI–NHC] complex bearing 4-ethoxycarbonylphenyl wingtip in the absence of *ortho* substitution (Scheme 130).^284^ This palladium precatalyst was obtained by a standard complexation of imidazolium chloride with [Pd­(CH_3_CN)_2_Cl_2_] in pyridine in the presence of NaI and K_2_CO_3_. The structure was confirmed by X-ray analysis showing square planar geometry with the Pd–C bond distance of 1.964 Å and the Pd–N bond distance of 2.086 Å. The authors conducted a thermogravimetric analysis, showing that the complex is thermally stable up to 208 °C, while at higher temperatures, dissociation of iodides and pyridine was observed. Catalytic performance was tested in Buchwald–Hartwig in amination of an axially chiral tosylate with benzophenone hydrazone in the presence of KO^*t*^Bu in dioxane at 100 °C. This complex afforded the product in 61% yield and showed higher efficiency than Pd/phosphine systems, such as Pd­(OAc)_2_/BINAP, Pd­(PPh_3_)_2_Cl_2_, and Pd­(PPh_3_)_4_, evaluated under the same conditions.

**Scheme 130:**
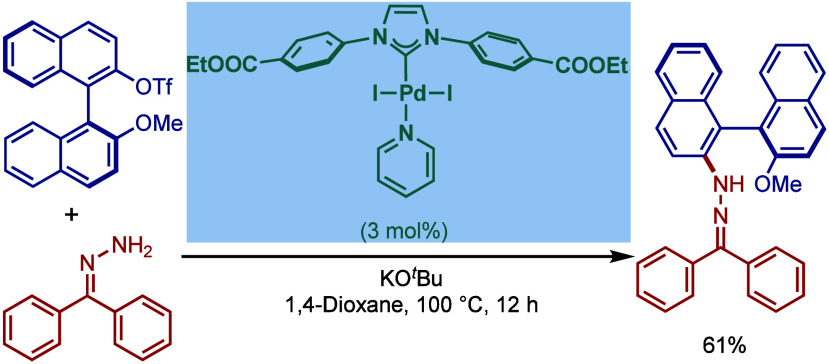
BHA Reaction of Axially Chiral 2′-Methoxy-2-trifluoromethanesulfonyloxy-1,1′-binaphthalene by Duan

### 2.6 BHA Reaction of Aryl Sulfides

The first example of BHA reaction of aryl sulfides catalyzed by [Pd–NHC] complexes was reported by Yorimitsu and co-workers in 2014 (Scheme 131).^285^ The authors tested different palladium systems and compared the catalytic activity of [Pd–PEPPSI] complexes, allyl congeners, and palladacycles in the model amination of thioanisole with *p*-toluidine at 100 °C. They found that a palladacycle-based catalyst, SingaCycle–A3, gave the highest yield (91%) using KHMDS in toluene. Furthermore, [Pd–PEPPSI–IPr] and [Pd­(IPr)­(allyl)­Cl] led to the product in 76% and 72% yields, respectively, while [Pd–PEPPSI–IMes], [Pd–PEPPSI–SIPr] and [Pd–PEPPSI–IPent] were significantly less reactive (0%, 25% and 5%, respectively). Interestingly, Pd/phosphine-based systems were also ineffective, including dppf, P*t*Bu_3_, PCy_3_, XPhos, DavePhos and RuPhos ligands. This method was then applied to the BHA reaction of aryl sulfides bearing different S–alkyl leaving groups, such as SMe, SPh, S*t*Bu, SC_12_H_25_, and various 1° aniline derivatives. Amines possessing electron-withdrawing and electron-donating groups, as well as sterically hindered anilines, can be successfully used in this cross-coupling. Furthermore, this method was applied to the modular synthesis of N-arylcarbazoles by a sequential C–S/C–H aminations. In 2015, the Yorimitsu group reported an extension of their protocol for BHA reaction of aryl sulfides with aliphatic amines using SingaCycle–A1 as a precatalyst in the presence of KHMDS in toluene at 60 °C (not shown).^286^ Under these conditions, the *ortho*-palladated dimethylbenzylamine as an ancillary ligand (SingaCycle–A1) was more effective than the amide-based SingaCycle–A3 (99% vs 77% yield). This protocol appears to be quite general for the cross-coupling of aryl sulfides with various 2° cyclic and select 1° aliphatic amines.

**Scheme 131:**
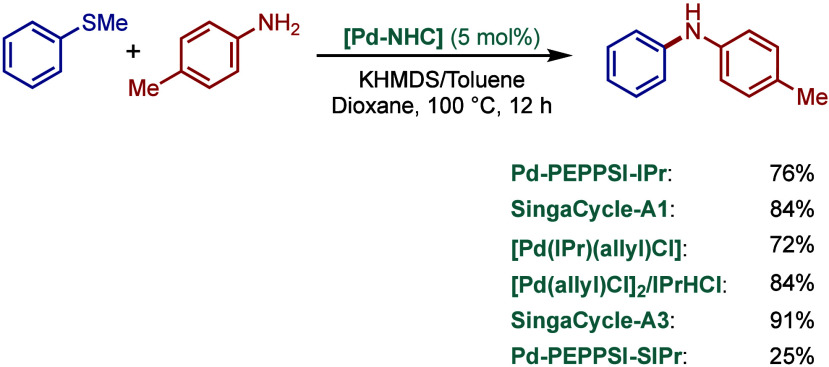
BHA Reaction of Aryl Sulfides Catalyzed by [Pd–NHC] Systems by Yorimitsu

In 2022, Poater, Nolan, Szostak, and co-workers reported selective BHA reaction of aryl sulfides catalyzed by [Pd­(IPr)­(μ-Cl)­Cl]_2_ (Scheme 132).^287^ The optimized conditions utilize 1.25 mol % of the catalyst in the presence of KHMDS in toluene at 100 °C. Extensive comparative studies showed that a palladium chloro dimer outperforms other [Pd–NHC] precatalysts, including [Pd–PEPPSI], [Pd­(NHC)­(allyl)­Cl], and palladacycle-based ones. Furthermore, a significant steric and electronic effect of the carbene ligand was found in that IMes, IPr* and SIPr complexes were completely ineffective. The optimized conditions were applied to the synthesis of diarylamines. Furthermore, the developed protocol was successfully applied to the late-stage functionalization of the antipsychotic drug Mellaril.

**Scheme 132:**
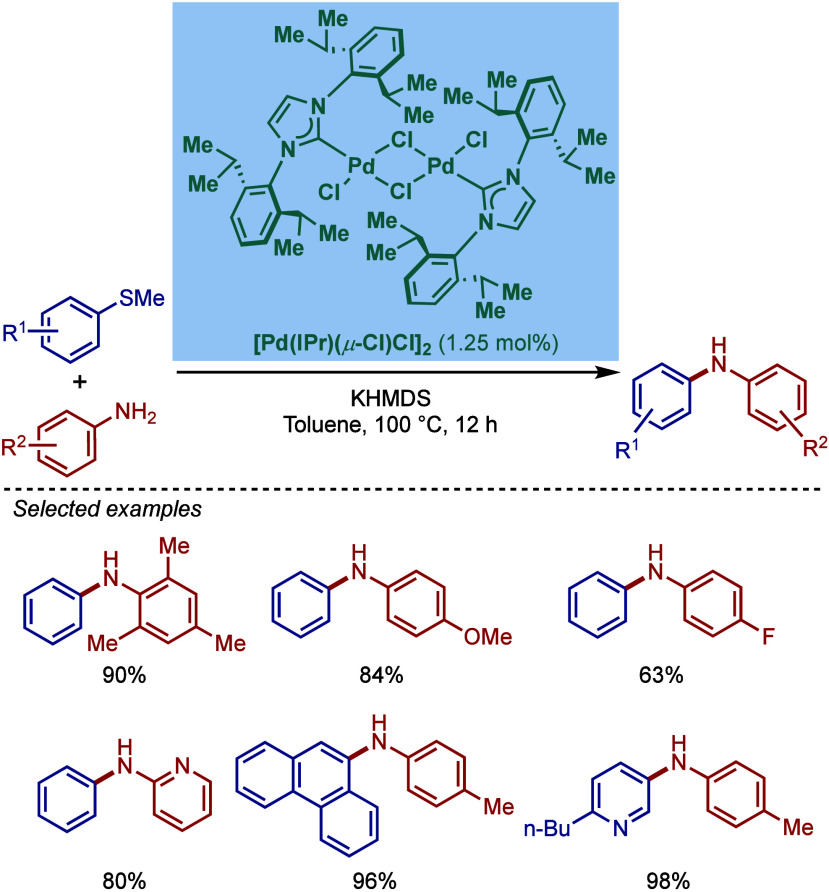
BHA Reaction of Aryl Sulfides Catalyzed by [Pd­(IPr)­(*μ*-Cl)­Cl]_2_ by Poater, Nolan, and Szostak

### 2.7 BHA Reaction of Aryl Sulfoxides

In 2018, Yorimitsu reported BHA reaction of diaryl sulfoxides catalyzed by [Pd–NHC] complexes (Scheme 133).^288^ It is worth noting that these reactions are performed under milder conditions than BHA reactions of aryl sulfides due to more electron negativity of the sulfoxide activating group. Different palladium complexes were evaluated in the model amination of diphenylsulfoxide with *p*-toluidine using KO^*t*^Bu in dioxane at 60 °C. The authors found that SingaCycle–A1 outperformed [Pd–PEPPSI–IPr] and XPhos–Pd–G2 precatalysts, giving the coupling product in 91% yield vs 11% and 47%, respectively. The presented method was applied to the BHA reaction with 1° and 2° aromatic and aliphatic amines bearing various functional groups, such as silyl, boryl, and halogen moieties. Moreover, a regioselective BHA reaction of unsymmetrical diaryl sulfoxides by using a sterically demanding 2,6-Xyl substituent was also presented.

**Scheme 133:**
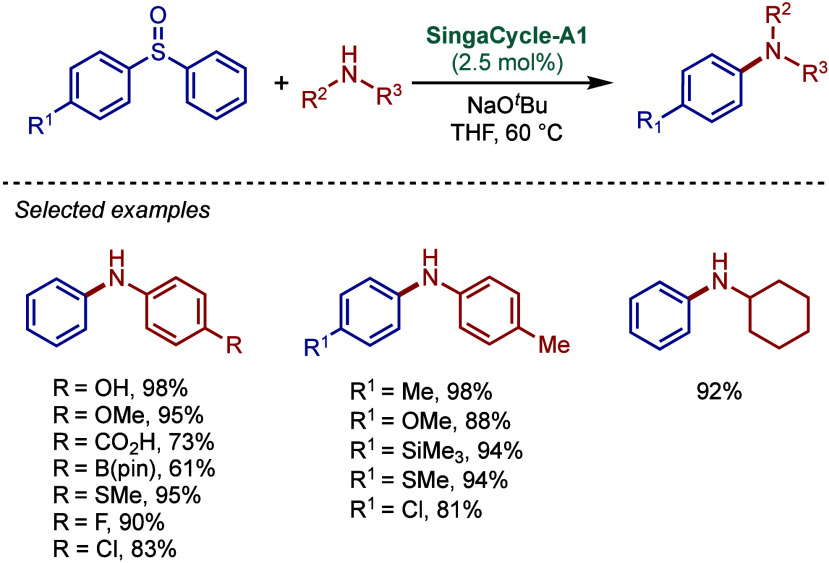
BHA Reaction of Diarylsulfoxides Catalyzed by SingaCycle–A1 by Yorimitsu

### 2.8 BHA Reaction of Nitroarenes

In 2019, Wu and Chen reported BHA reaction of nitroarenes catalyzed by [Pd–NHC] systems based on imidazol-2-ylidene­[1,5-*a*]­pyridine scaffold (ImPy) (Scheme 134).^289^ Despite the low activity of Ar–NO_2_ bonds to the oxidative addition step, these authors have found that the sterically demanding biaryl imidazo­[1,5-*a*]­pyridine template promotes this challenging process. Interestingly, classical imidazol-2-ylidenes as well as imidazol-2-ylidene­[1,5-*a*]­pyridines without sterically demanding C5-substitution were completely ineffective in this reaction. There appear to be subtle steric and electronic factors of the N2-substituents in the ligand structure, showing that the most active one was the ligand with 2,6-diethylphenyl wingtip. This ligand allowed a remarkably broad range of BHA reactions of nitroarenes with 1° and 2° aromatic and aliphatic amines using mild base, K_3_PO_4_, in dioxane at 130 °C. This permits functional group tolerance to esters, ketones, nitriles, alkynes, and heterocycles, leading to cross-coupling products in good to high yields.

**Scheme 134:**
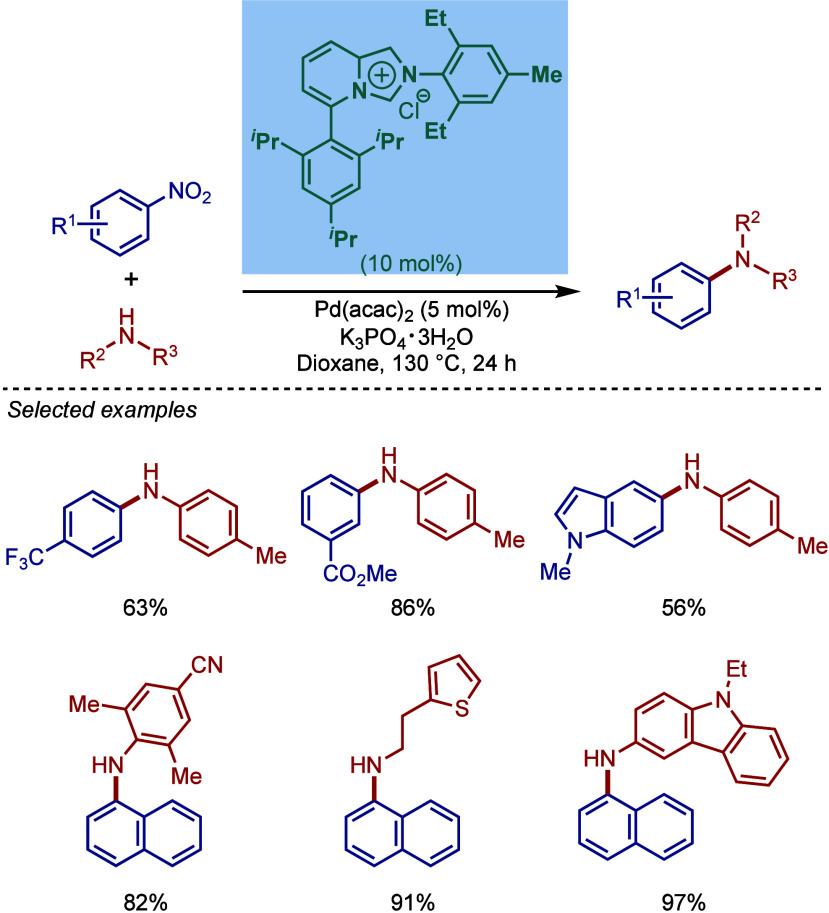
BHA Reaction of Nitroarenes Catalyzed by [Pd–ImPy–NHCs] by Wu and Chen

### 2.9 Acyl BHA Reaction

#### 2.9.1 BHA Reaction of Amides

##### 2.9.1.1 [Pd­(NHC)­(η^3^-allyl)­Cl] Complexes

The first example of transamidation of carboxamides catalyzed by [Pd–NHC] complexes was reported by Szostak in 2017 (Scheme 135).^87^ Various catalytic systems, such as Pd­(OAc)_2_/PR_3_, Pd­(OAc)_2_/NHC salt, and [Pd­(NHC)­(η^3^-allyl)­Cl] complexes, were examined and the highest reactivity was observed for the [Pd­(IPr)­(η^3^-cin)­Cl] complex using K_2_CO_3_ in DME at 110 °C. Different N-substituted amides, such as N-alkyl and N-aryl, N-Boc, and N-Ts-amides, are successful substrates for this process. The preferred class of substrates are N-Boc activated amides due to the ease of synthesis by a direct N-*tert*-butoxycarbonylation of generic 2° amides. These mild Buchwald–Hartwig conditions are compatible with a broad range of amines and amides, including sterically hindered, heterocyclic, electron-donating and electron-withdrawing. The proposed high selectivity is achieved by oxidative addition to the N-activated amide bond, leading to the acyl-palladium intermediate, which undergoes ligand exchange and reductive elimination to give the coupling the desired product.

**Scheme 135:**
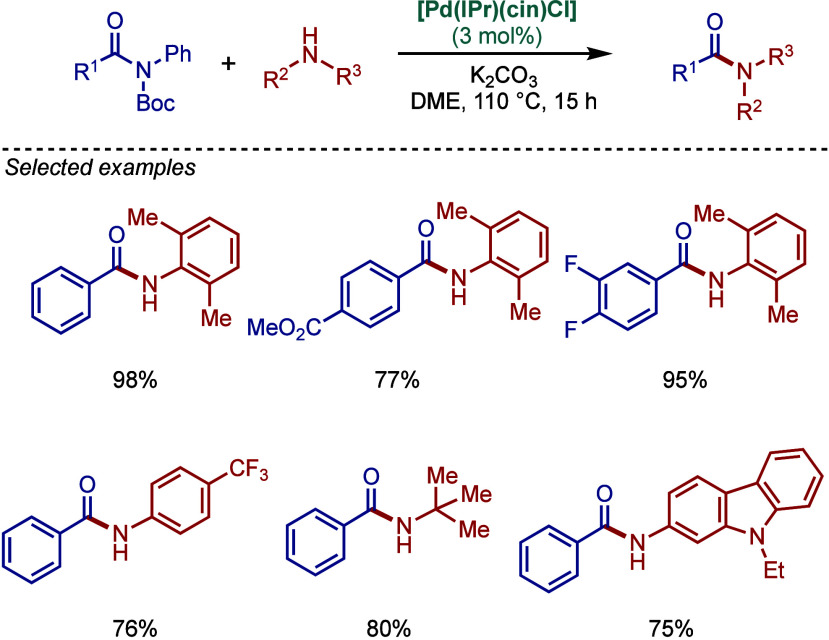
Acyl BHA Reaction Catalyzed by [Pd­(IPr)­(cin)­Cl] by Szostak

In 2020, Poater, Nolan, and Szostak reported an experimental and computational study of Buchwald–Hartwig cross-coupling of amides catalyzed by [Pd­(NHC)­(allyl)­Cl] complexes (Scheme 136).^290^ Different [Pd–NHC] precatalysts (NHC = IPr, IPr*, SIPr, IMes) were evaluated across the cross-coupling of amides and 1° and 2° anilines bearing deactivating and sensitive functional groups. It was found that [Pd­(IPr)­(cin)­Cl] and [Pd­(IPr)­(allyl)­Cl] are the most general precatalysts for BHA reactions with non-nucleophilic substrates. Based on DFT calculations, oxidative addition to the N–C­(O) bond was found to be the rate-determining step. This class of air- and moisture-stable [Pd–NHC] precatalysts permits transamidation to be performed under mild conditions with tolerance to sensitive functional groups, such as esters, nitro, NH–amides, and NH–sulfonamides.

**Scheme 136:**
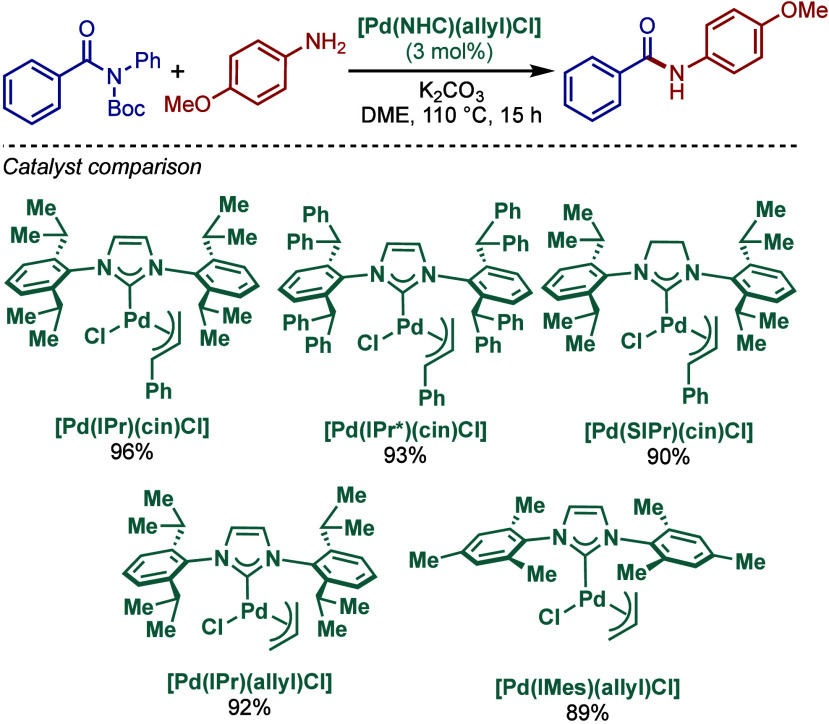
Acyl BHA Reaction Catalyzed by [Pd­(NHC)­(allyl)­Cl] Complexes by Poater, Nolan, and Szostak

In 2021, Szostak reported application in Buchwald–Hartwig transamidation of a new class of sterically hindered IPr^#^ ligands obtained by a modular peralkylation of anilines (Scheme 137).^291^ The corresponding [Pd­(IPr^#^)­(cin)­Cl] complex was prepared in 89% yield by reaction with [Pd­(cin)­Cl]_2_ in the presence of KO^*t*^Bu and characterized by X-ray analysis showing a Pd–C bond length of 2.044 Å and a %V_*bur*_ of 44.7% (see also Section 3.1). This complex showed high activity in acyl BHA reaction, leading to the amination product in 75% yield.

**Scheme 137:**
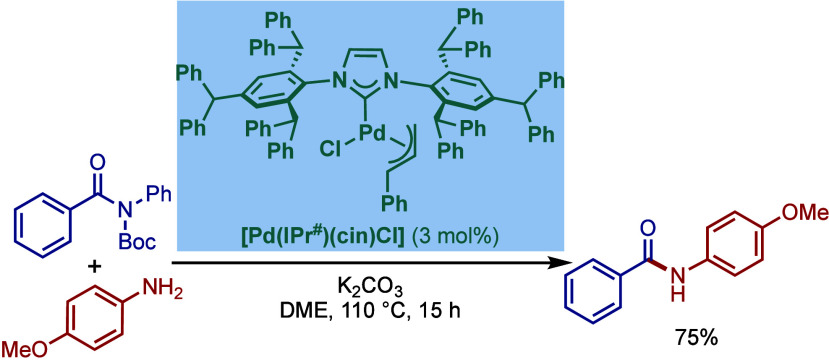
Acyl BHA Reaction Catalyzed by [Pd­(IPr^#^)­(cin)­Cl] by Szostak

In 2021, Lei and Szostak reported a green approach to the acyl Buchwald–Hartwig cross-coupling of amides using sustainable solvents (Scheme 138).^292^ In a comprehensive evaluation of different sustainable reaction media, MTBE (methyl *tert-*butyl ether) and 2-MeTHF (2-methyltetrahydrofuran) were identified as optimal solvents for Buchwald–Hartwig transamidation. Screening of various [Pd–NHC] complexes showed that [Pd­(IPr)­(cin)­Cl] outperformed other precatalysts, such as [Pd–PEPPSI–IPr], SingaCycle–A3, and [Pd­(IPr)­(allyl)­Cl]. This method is characterized by a wide scope of amides and amines that are applicable to cross-coupling. Furthermore, the excellent functional group tolerance allows for functionalization of biologically active compounds and the synthesis of agrochemicals containing amide bonds.

**Scheme 138:**
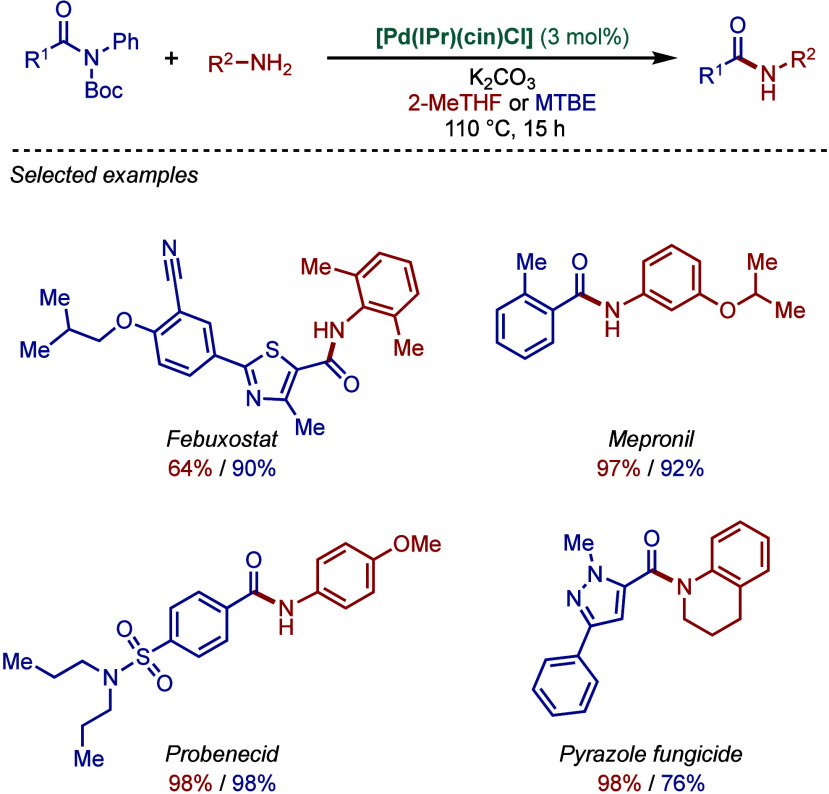
Acyl BHA Reaction of Amides in Functionalization of Bioactive Compounds in Green Solvents Catalyzed by [Pd­(IPr)­(cin)­Cl] by Lei and Szostak

##### 2.9.1.2 [Pd­(NHC)­(3-Cl-py)­Cl_2_] Complexes

In 2017, Szostak and co-workers reported the application of [Pd–PEPPSI] precatalysts to the acyl BHA reaction of amides (Scheme 139).^293^ Interestingly, the IPr complex bearing pyridine ligand, [Pd–PEPPSI–IPr-py], outperformed its 3-Cl-py congener as well as the related 1-Me-imidazole complex, [Pd­(IPr)­(1-Me-im)­Cl_2_]. Furthermore, the following order of reactivity in terms of the NHC ligand was observed: IPr > IPent > IMes. This catalyst was applied to the BHA reaction of various readily available amides, such as N-Boc-carbamates and N-sulfonamides, giving products in high yields. A TON of 320 was determined for the reaction of N-Boc-N-Ph-benzamide with aniline.

**Scheme 139:**
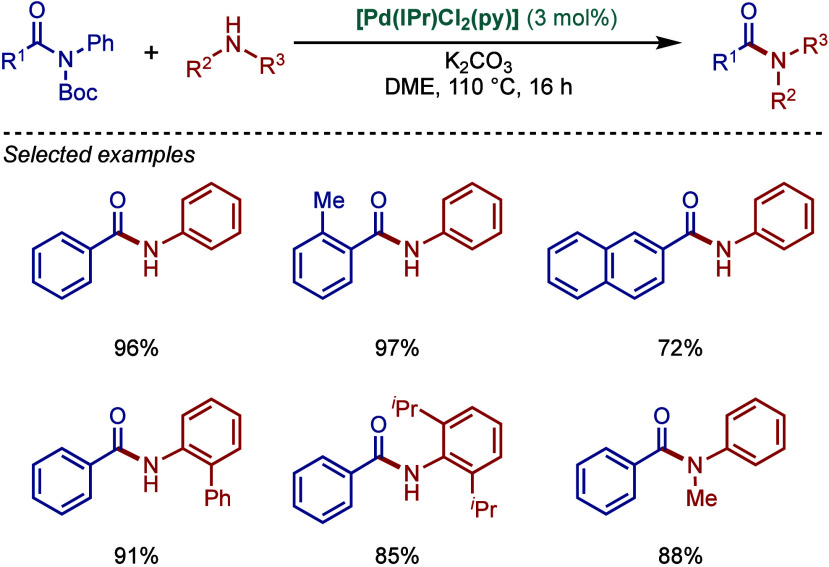
Acyl BHA Reaction Catalyzed by [Pd–PEPPSI–IPr-py] by Szostak

In 2020, Szostak and co-workers reported selective N–C­(O) activation of carbon–nitrogen bonds in N-acyl-carbazoles and application to BHA reaction using a [Pd–PEPPSI–IPr] precatalyst (Scheme 140).^294^ In this class of substrates, the high reactivity was achieved as a result of N_lp_ to Ar conjugation in the planar carbazole unit. The acyl Buchwald–Hartwig cross-coupling of a model N-benzoylcarbazole and *p*-anisidine gave the coupling product in 70% yield in the presence of K_2_CO_3_ in DME at 140 °C.

**Scheme 140:**
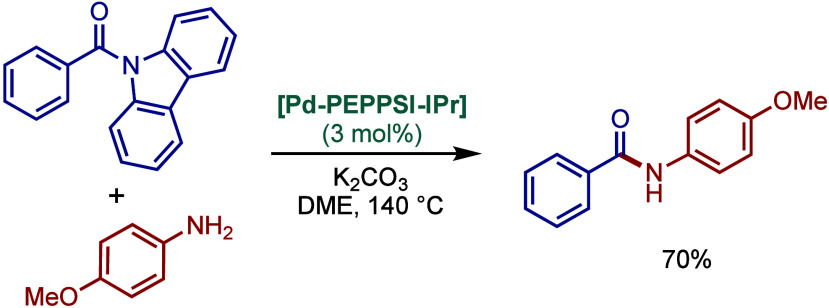
Acyl BHA Reaction of N-Benzoylcarbazoles Catalyzed by [Pd–PEPPSI–IPr] by Szostak

Furthermore, an application of a sterically demanding [Pd–PEPPSI–IPr^#^] complex (see also Section 2.3.8) to the BHA reaction of benzamides was reported, where the coupling product was formed in 85% yield using K_2_CO_3_ in DME at 110 °C.^241^

##### 2.9.1.3 [Pd­(NHC)­(μ-Cl)­Cl]_2_ Complexes

The application of chloro-dimers, [Pd­(IPr)­Cl_2_]_2_ and [Pd­(SIPr)­Cl_2_]_2_, to acyl BHA reaction was reported by Poater, Nolan and Szostak in 2020 (Scheme 141).^290^ The catalytic activity was examined in transamidation of a model N-Boc/N-Ph-benzamide with various non-nucleophilic and sterically hindered anilines. These chloro-dimer precatalysts were less effective compared to their allyl-based congeners under the conditions examined (see Section 2.9.1.1). However, these [Pd­(IPr)­Cl_2_]_2_ and [Pd­(SIPr)­Cl_2_]_2_ complexes were highly active for BHA reaction with strongly deactivated anilines bearing sulfonamide and ester groups.

**Scheme 141:**
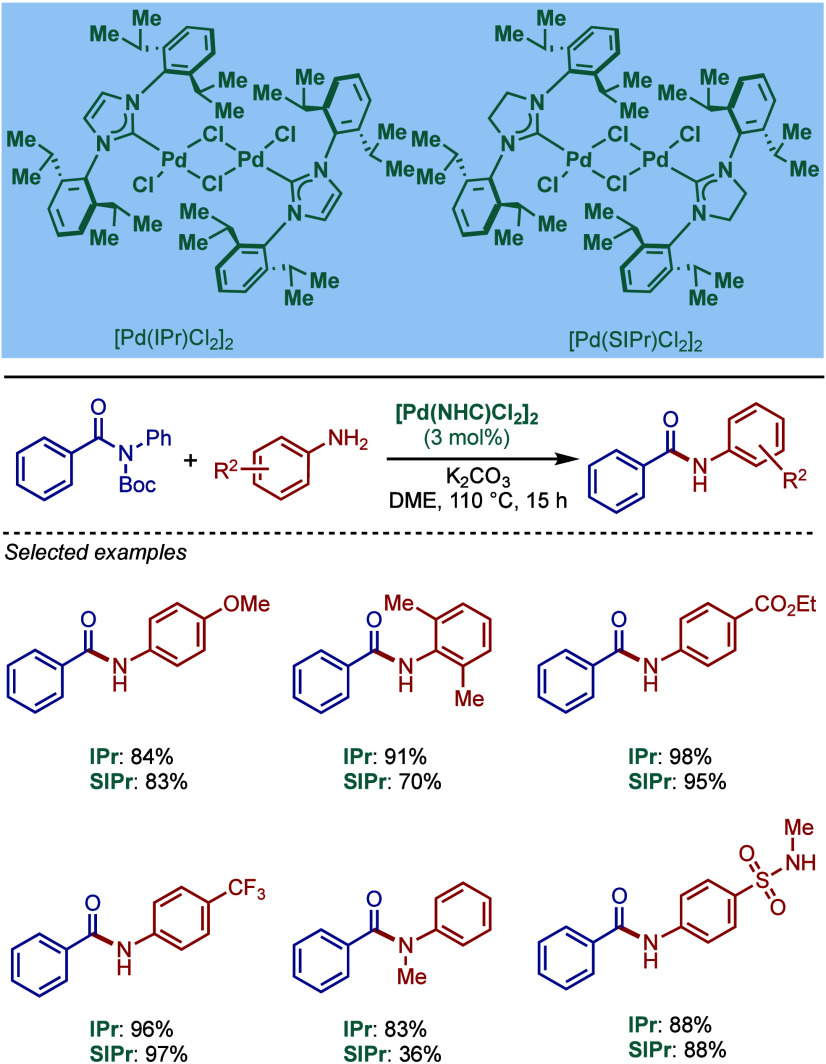
Acyl BHA Reaction Catalyzed by [Pd­(IPr)­Cl_2_]_2_ and [Pd­(SIPr)­Cl_2_]_2_ Complexes by Poater, Nolan, and Szostak

##### 2.9.1.4 [Pd­(NHC)­(acac)­Cl] Complexes

In 2019, Szostak and co-workers reported well-defined, air-stable precatalysts [Pd­(NHC)­(acac)­Cl] for the acyl Buchwald–Hartwig cross-coupling of amides (Scheme 142).^295^ The key advantage of these acac–Pd–NHC complexes is their facile synthesis by a direct complexation of the imidazolium salt in the presence of Pd­(acac)_2_, rendering them the most operationally convenient class of well-defined [Pd­(II)–NHC] precatalysts. This permitted for development of a protocol for in situ screening of NHC salts in acyl Buchwald–Hartwig cross-coupling reactions. The best results were noted for IPr, IMes and SIPr complexes, while N-aliphatic I*t*Bu, ICy, and sterically hindered IPr* congeners led to products in lower yields. The parent [Pd­(IPr)­(acac)­Cl] precatalyst was applied to the BHA reaction of a broad scope of amides and 1° and 2° anilines, giving products in high yields. Moreover, the turnover number of 410 was determined for BHA reaction at 0.10 mol % of the [Pd­(IPr)­(acac)­Cl] complex.

**Scheme 142:**
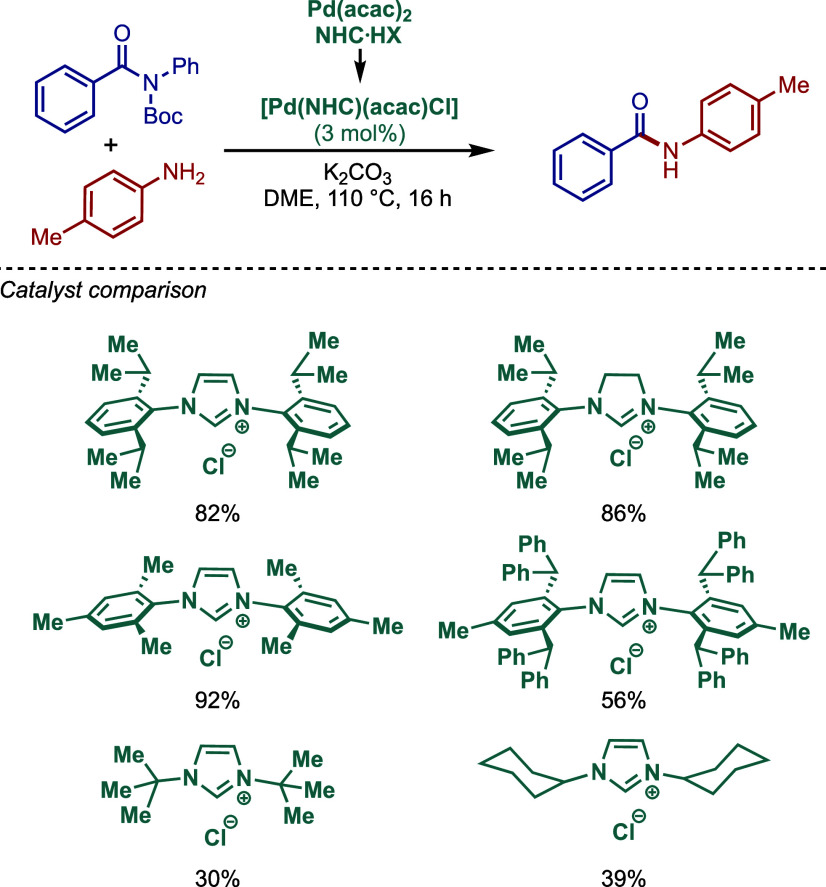
Acyl BHA Reaction Catalyzed by [Pd­(NHC)­(acac)­Cl] Complexes by Szostak

##### 2.9.1.5 [Pd­(NHC)­(OAc)_2_] Complexes

In 2024, Szostak and co-workers reported a related class of carboxylate [Pd­(NHC)­(OAc)_2_] complexes for acyl BHA reaction by selective N–C­(O) bond cleavage (Scheme 143).^296^ These air- and moisture-stable catalysts were found to be highly effective in acyl BHA reaction using K_2_CO_3_ in DME at 110 °C with functional group tolerance to sterically hindered anilines and ester groups.

**Scheme 143:**
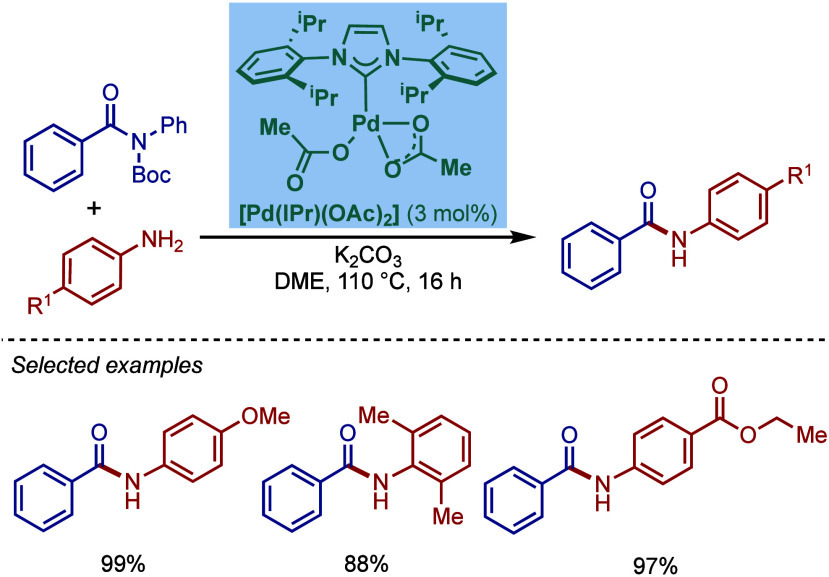
Acyl BHA reaction Catalyzed by [Pd­(NHC)­(OAc)_2_] Complexes by Szostak

#### 2.9.2 BHA Reaction of Esters

##### 2.9.2.1 [Pd­(NHC)­(η^3^-allyl)­Cl] Complexes

In 2017, Newman and co-workers reported the first method for acyl Buchwald–Hartwig cross-coupling of aryl esters with anilines catalyzed by [Pd–NHC] complexes (Scheme 144).^297^ Evaluation of different palladium catalysts revealed that [Pd­(IPr)­(η^3^-allyl)­Cl] was the preferred catalyst using conditions with K_2_CO_3_ as a base in the presence of H_2_O (10 equiv) in toluene at 110 °C. Other ligands, such as IMes, PPh_3_, PCy_3_, P*t*Bu_3_, BINAP, and SPhos, were ineffective. This method works well with O-phenolic esters, permitting the amination with non-nucleophilic anilines in the presence of a weak base. A broad scope of amides was obtained including aliphatic, aromatic, and heterocyclic in the coupling with 1° anilines. Moreover, a chiral proline ester was used as a successful substrate with minimal loss of enantiopurity. This mechanism involves C–O bond activation by a direct oxidative addition to give acyl-metal intermediate, followed by ligand exchange and reductive elimination.

**Scheme 144:**
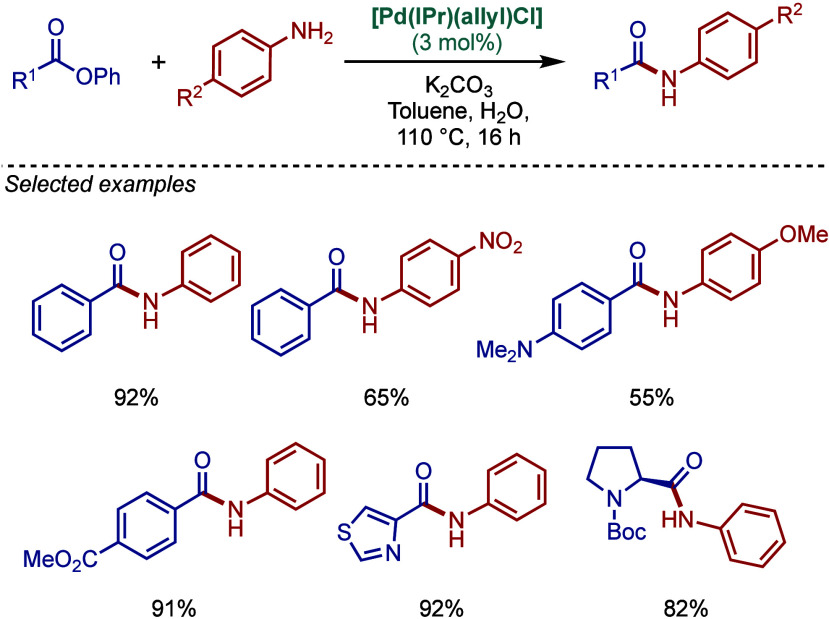
Acyl BHA Reaction of Esters Catalyzed by [Pd­(IPr)­(η^3^-allyl)­Cl] by Newman

In 2018, Hazari and co-workers reported acyl Buchwald–Hartwig cross-coupling of phenolic esters catalyzed by [Pd­(SIPr)­(1-*t*-Bu-ind)­Cl] (Scheme 145).^298^ Under the optimized reaction conditions using CsCO_3_ as a base in THF/H_2_O at 40 °C, the highest activity was observed for the imidazolin-2-ylidenyl complex, while no reaction was observed for other NHC or phosphine ligands under the same conditions, such as [Pd­(SIPr)­(allyl)­Cl], [Pd­(IPr)­(allyl)­Cl], P*t*Bu, and XPhos. Furthermore, replacing the SIPr ligand in [Pd­(SIPr)­(1-*t*-Bu-ind)­Cl] with IPr, SIMes, and IPr*^OMe^ resulted in no reactivity, highlighting finely tuned conditions for this transformation. The authors proposed that the use of water as a cosolvent enabled faster activation of the catalyst, facilitating the amidation reaction. The coupling products were obtained in good to high yields under very mild reaction conditions.

**Scheme 145:**
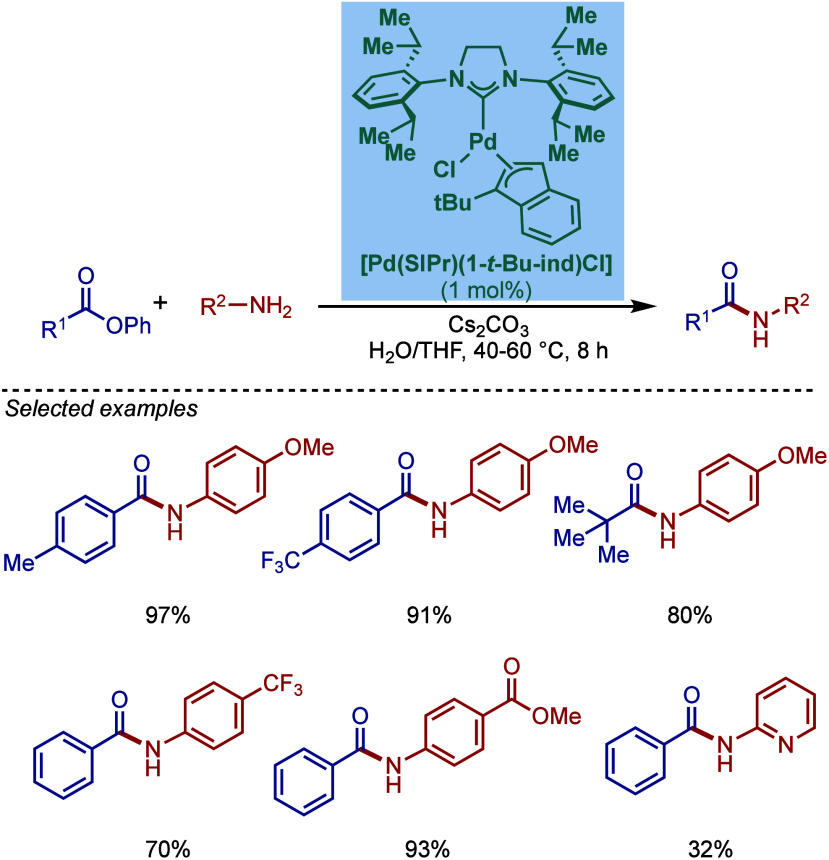
Acyl BHA Reaction of Esters Catalyzed by [Pd­(SIPr)­(1-*t*-Bu-ind)­Cl] by Hazari

An interesting example of acyl BHA reaction of phenolic esters catalyzed by mesoionic [Pd–aNHC]-type complexes was reported by Mendoza-Espinosa and co-workers in 2019 (Scheme 146).^299^ These triazolylidene complexes bearing hydroxyalkyl wingtip (alkyl = CH_2_, (CH_2_)_2_, (CH_2_)_3_) are easily accessible by the direct complexation of triazolium salts with [Pd­(allyl)­Cl]_2_ or [Pd­(cin)­Cl]_2_ in the presence of KHMDS at −78 °C. Their catalytic activity was evaluated in the BHA reaction of phenyl benzoate using K_2_CO_3_ in the presence of water in toluene at 110 °C. The authors found that cinnamyl-type precatalysts showed higher activity compared to their allyl congeners. Furthermore, the most reactive was the catalyst with the shortest hydroxyalkyl chain in the order of −CH_2_OH > −(CH)_2_OH > −(CH)_3_OH.

**Scheme 146:**
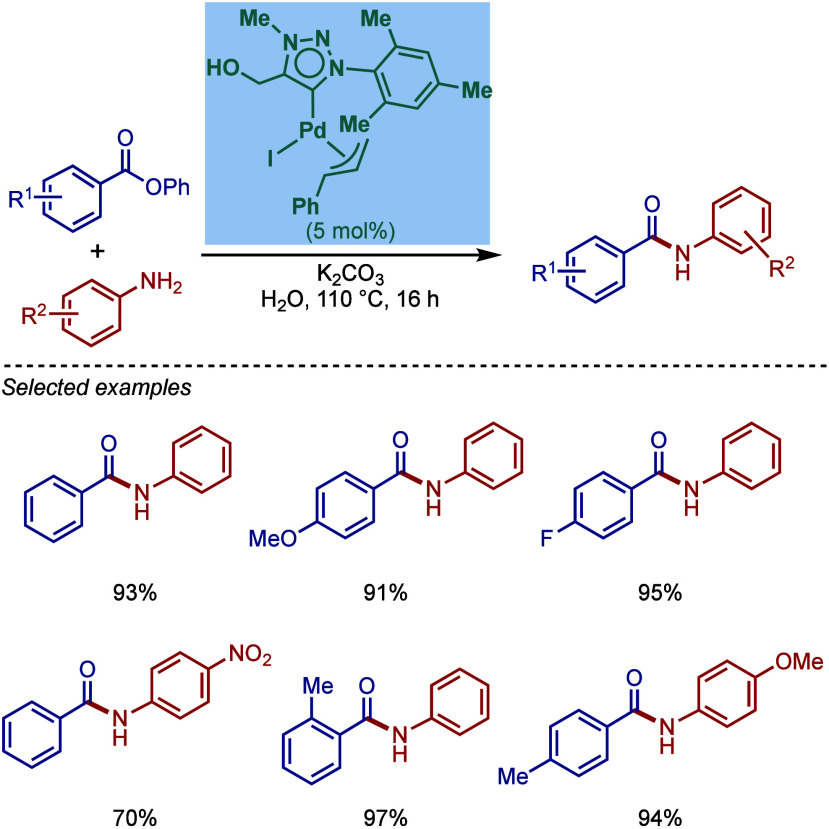
Acyl BHA Reaction of Esters Catalyzed by [Pd–aNHC] Complexes by Mendoza-Espinosa

Another example of acyl BHA reaction of esters using sterically demanding [Pd­(IPr^#^)­(cin)­Cl] was reported by Szostak and co-workers in 2019 (Scheme 147).^291^ It is interesting to note that the PEPPSI congener, [Pd–PEPPSI–IPr^#^], was significantly less reactive under the same conditions (see also Section 2.9.1.1).

**Scheme 147:**
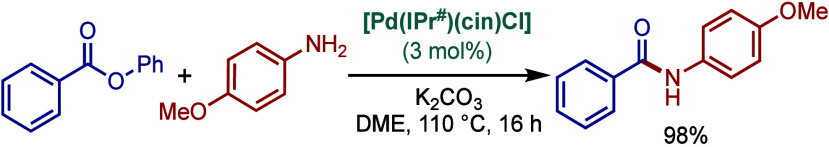
Acyl BHA Reaction of Esters Catalyzed by [Pd­(IPr^#^)­Pd­(cin)­Cl] by Szostak

##### 2.9.2.2 [Pd­(NHC)­(3-Cl-py)­Cl_2_] PEPPSI Complexes

In 2017, Szostak and co-workers reported the application of [Pd–PEPPSI] complexes to the direct acyl Buchwald–Hartwig acyl cross-coupling of phenolic esters (Scheme 148).^293^ Evaluation of complexes with different NHC ligands revealed that [Pd–PEPPSI–IPr] is more reactive than both its more and less sterically hindered analogues, [Pd–PEPPSI–IPent] and [Pd–PEPPSI–IMes] (>98% vs 59% and 26%, respectively). It is worth noting that the same conditions were also successfully applied for transamidation of amides activated with N-Boc and N-Ts groups, indicating the involvement of a similar acyl–Pd intermediate (see Section 2.9.1.2). The method was successfully applied to the BHA reaction of a broad range of phenolic esters with 1° and 2° anilines. Furthermore, a TON of 350 in the cross-coupling of phenyl benzoate with aniline was determined.

**Scheme 148:**
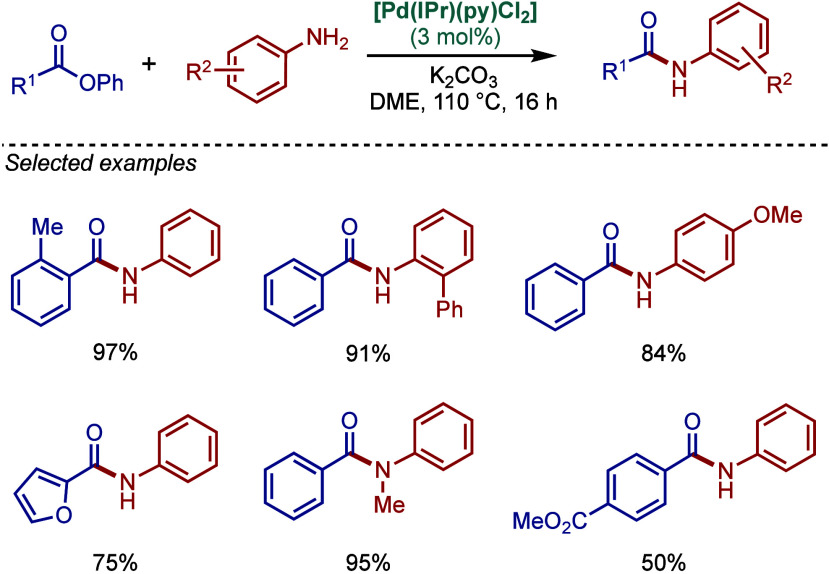
Acyl BHA Reaction of Esters Catalyzed by [Pd–PEPPSI–IPr] by Szostak

In 2022, Tan and Shen reported the application of their N-indole-functionalized [Pd–PEPPSI–NHC] complexes to the acyl BHA reaction of esters (Scheme 149).^236^ They found that the C3-^*i*^Pr/C5-methyl functionalized catalyst was effective in amidation of phenyl benzoate using K_2_CO_3_ in DME at 80–110 °C, giving the corresponding amides in 94–95% yield.

**Scheme 149:**
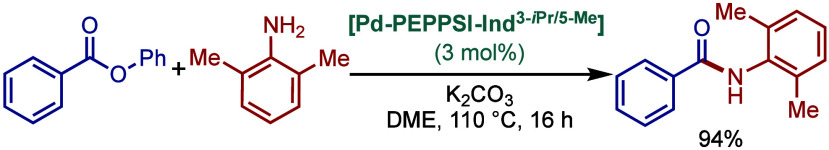
Acyl BHA Reaction of Esters Catalyzed by Indolyl-Wingtip-Modified [Pd–PEPPSI–NHC] Complexes by Tan and Shen

In 2022, Yao and Xu reported the application of their sterically hindered, backbone dianisole-functionalized [Pd–PEPPSI–IPr^4‑MeOC6H4^] complex to the BHA reaction of esters (Scheme 150).^235^ Modification of the imidazol-2-ylidene backbone with aromatic rings at the C3/C4 positions resulted in higher catalytic activity compared to [Pd–PEPPSI–IPr] and [Pd–PEPPSI–IPr^An^] congeners using K_2_CO_3_ in toluene at 110 °C (96% yield vs 84% and 57%, respectively). This catalyst was applied to the BHA reaction with a broad range of anilines, affording the amide products in high yields. It is worth noting that this catalyst is compatible with various electronically deactivated and sterically hindered anilines.

**Scheme 150:**
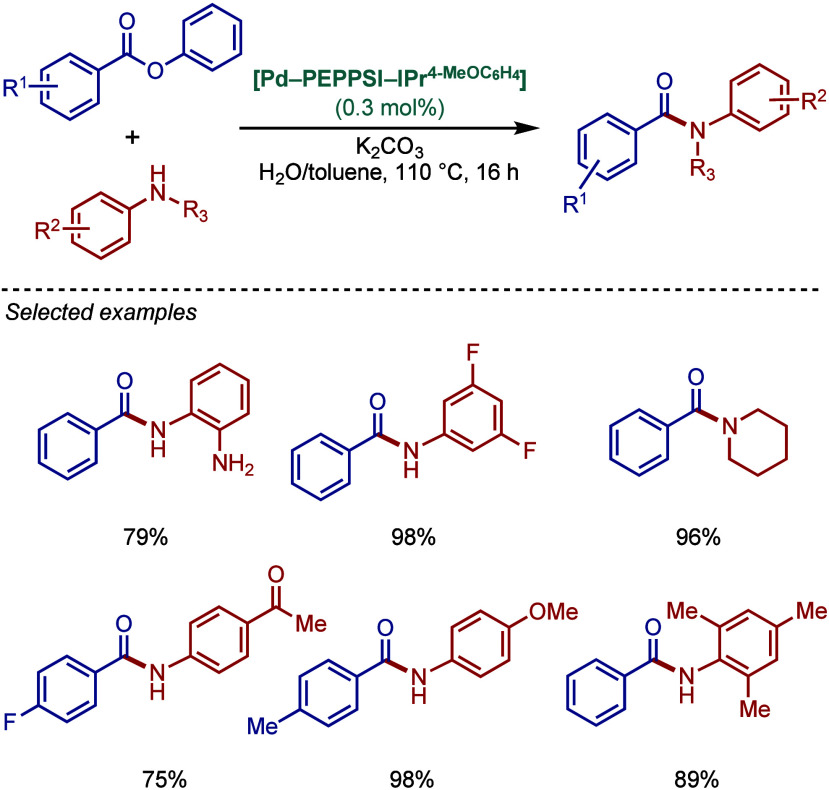
Acyl BHA Reaction of Esters Catalyzed by Backbone-Modified [Pd–PEPPSI–IPr^4‑MeOC6H4^] Complex by Yao and Xu

##### 2.9.2.3 [Pd­(NHC)­(acac)­Cl] Complexes

Acyl BHA reaction of phenolic esters catalyzed by [Pd­(IPr)­(acac)­Cl] was reported by Szostak in 2019 (Scheme 151).^295^ This easily prepared catalyst was found to be effective using K_2_CO_3_ in DME at 110 °C, confirming its general utility in C­(acyl)–X cross-coupling reactions (see also Section 2.9.1.4).

**Scheme 151:**
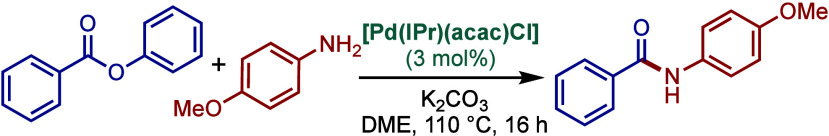
Acyl BHA Reaction of Esters Catalyzed by [Pd­(IPr)­(acac)­Cl] by Szostak

## 3 Nickel–NHC Complexes

### 3.1 In Situ-Formed Ni(0)–NHC Complexes

In 2001, the Fort group reported the first example of BHA reaction by Ni(0)–NHC catalysis (Scheme 152).^86^ They demonstrated the cross-coupling of aryl and heteroaryl chlorides with various 2° aliphatic amines, achieving excellent yields using an optimized system of Ni­(acac)_2_ and SIPr·HCl in the presence of NaH in *t*-BuOH at 65 °C. The authors credited the strong electron-donating properties and steric bulk of NHC ligands with accelerating the oxidative addition of aryl chlorides to Ni(0) and facilitating the C–N bond-forming reductive elimination. This catalytic system showed improved catalytic efficiency in terms of lower reaction temperature and higher yields compared with the original Ni­(cod)_2_/dppf system reported by Buchwald in 1997.^25^ The authors evaluated different N-heterocyclic carbene ligands, such as IMes, SIMes, IPr, and SIPr, and found that both imidazol-2-ylidene- and imidazolin-2-ylidene-based ligands, IPr and SIPr, were the most effective. The reaction conditions involved the in situ generation of NaO^*t*^Bu to reduce Ni­(acac)_2_ to Ni(0) and deprotonate the imidazolinium salt. Furthermore, Ni­(OAc)_2_ and NiCl_2_ were also effective; however, these precursors showed lower catalytic activity. The authors identified the active catalytic species as an in situ-formed Ni(0)–NHC complex, with an optimal NHC/Ni ratio of 4:1 for maximum reactivity.

**Scheme 152:**
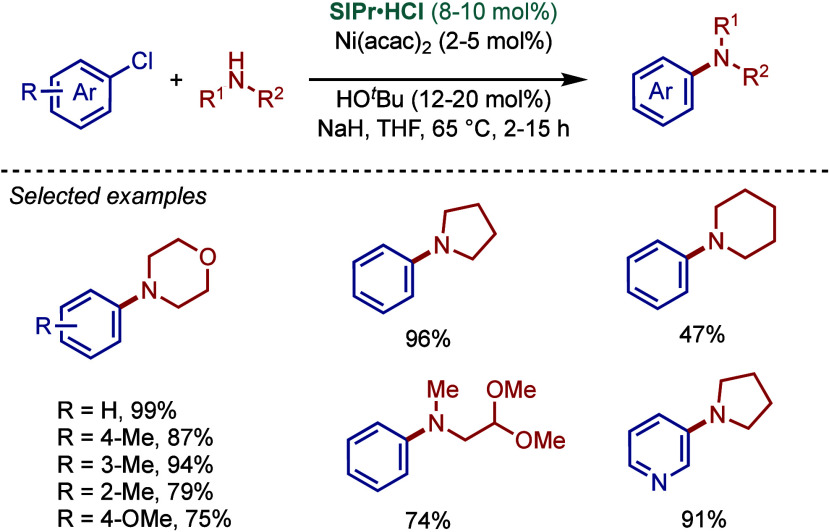
BHA Reaction of Secondary Amines Catalyzed by In Situ-Formed Ni(0)–NHC by Fort

In 2002, the same research group expanded the scope of their in situ-formed Ni(0)–NHC complex system to various 2° aliphatic amines and anilines (Scheme 153A).^300^ They also evaluated an expanded set of NHC precursors, such as bis-carbenes and tridentate ligands. Interestingly, among the ligands tested, SIPr remained the most effective (94% yield), closely followed by IPr (90% yield), while N-aliphatic imidazol-2-ylidne ligand, I*t*Bu, also demonstrated promising reactivity (44% yield) (Scheme 153B).

**Scheme 153:**
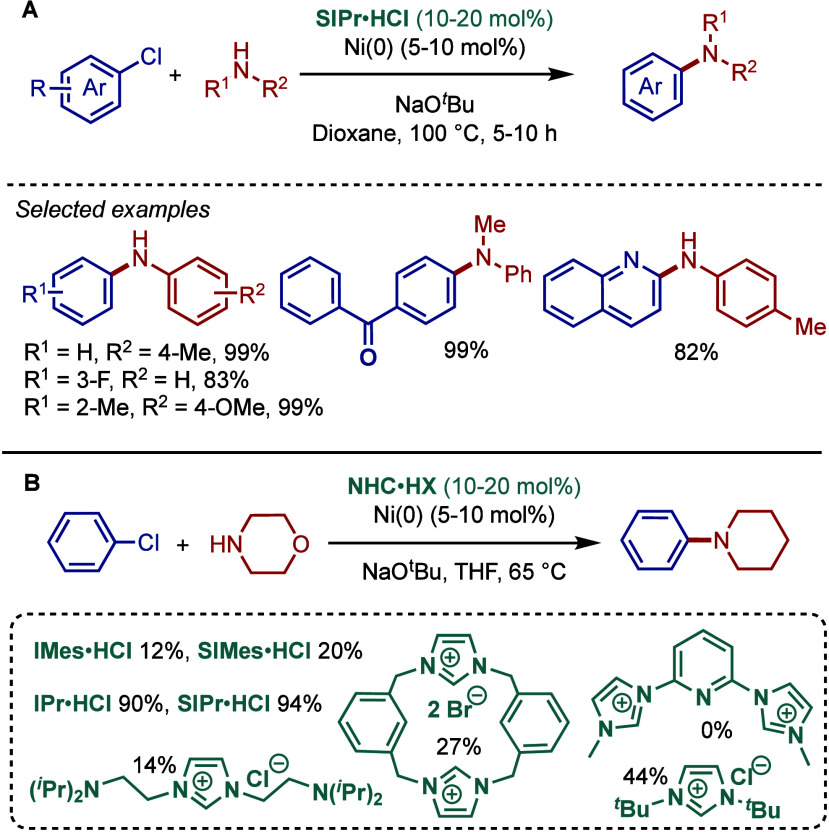
BHA Reaction of Aliphatic Amines and Anilines Catalyzed by In Situ-Formed Ni(0)–NHC by Fort

In 2003, Fort, Schneider, and co-workers reported an efficient application of their Ni(0)–NHC catalysis to the intramolecular BHA reaction of aryl chlorides, establishing a versatile cyclization protocol for the formation of five-, six-, and seven-membered azacyclic rings (Scheme 154).^301^ Notably, two distinct catalytic systems, Ni/bpy (2,2′-bipyridine) and Ni/SIPr, were thoroughly investigated, and the carbene-based system showed superior reactivity at 2–10 mol % catalyst loading in the model amination (95–97% yields vs 47–84% yields). In the substrate scope studies, both demonstrated comparable efficiency at 5 mol % catalyst loading. These conditions also enabled the cyclization of cyclic amines to obtain biologically relevant fused heterocycles.

**Scheme 154:**
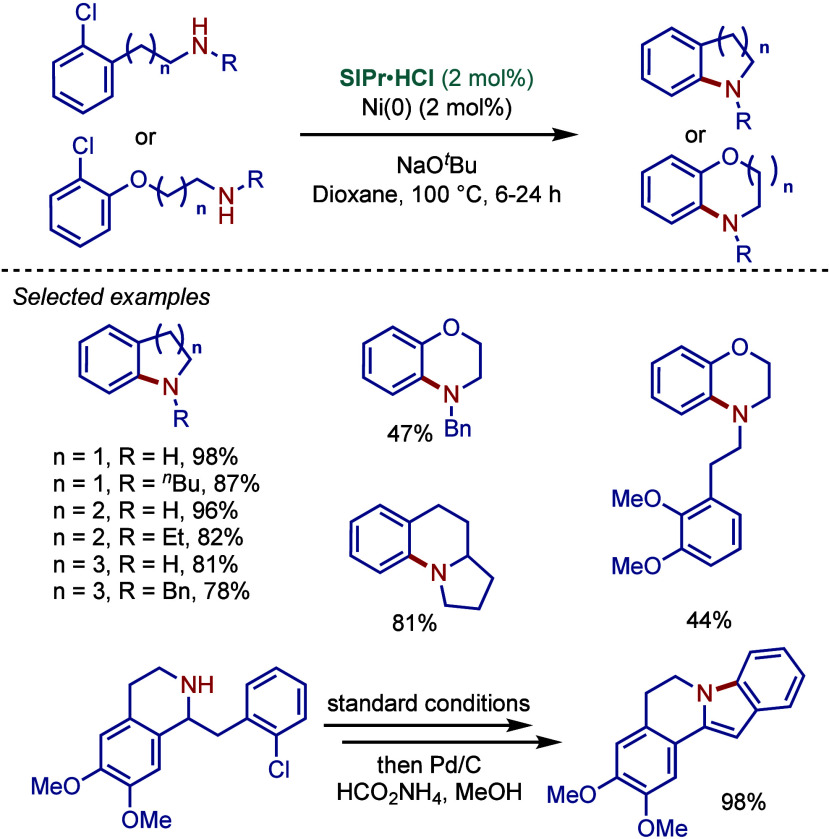
Intramolecular BHA Reaction Catalyzed by In Situ-Formed Ni(0)–NHC by Fort

In 2005, the same group further investigated their in situ-generated Ni(0)–NHC system for challenging Buchwald–Hartwig cross-coupling of aromatic diamines (Scheme 155).^302^ Mechanistic studies by ^13^C NMR spectroscopy revealed the formation of [Ni­(IPr_2_)] complex using their *tert*-butoxide/NaH conditions. This approach enabled selective diarylation or monoarylation of aryl diamines controlled by the stoichiometry of the aryl halide (2.4 vs 1.2 equiv). In agreement with their previous studies, IPr and SIPr showed significantly higher reactivity than less sterically demanding IMes and SIMes ligands (89–96% vs 11–13%).

**Scheme 155:**
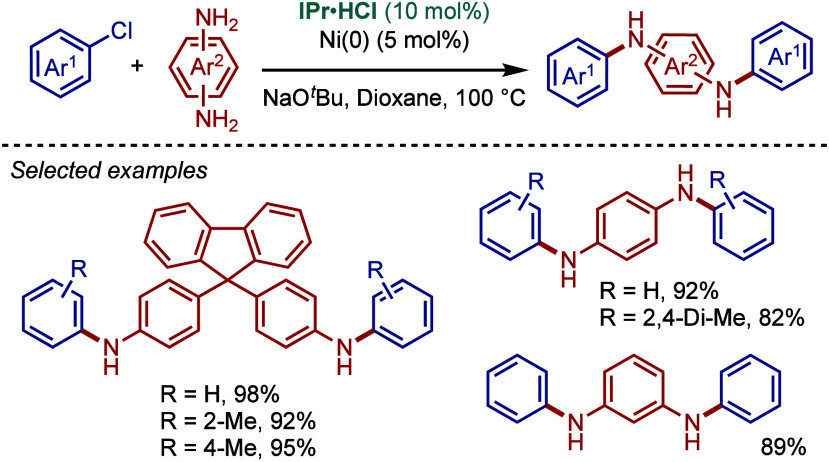
BHA Reaction of Diamines Catalyzed by In Situ-Formed Ni(0)–NHC by Schneider

In 2007, the Yang group reported an efficient Ni(0)–NHC catalytic system for BHA reactions using [Ni­(PPh_3_)_2_(aryl)­X] complexes in the presence of NHC salt and NaO^*t*^Bu in THF or dioxane at 65 or 80 °C (Scheme 156).^303^ This report presented a potential advantage in terms of catalyst generation as instead of using Ni­(acac)_2_ in the presence of an external reductant, such as NaH, they deployed air- and moisture-stable mixed NHC/phosphine [Ni­(PPh_3_)_2_(aryl)­X] precursors. These precursors were found to undergo facile reduction to Ni(0) under the reaction conditions. Stoichiometric studies revealed the reactivity in BHA reaction in the following order: [Ni­(PPh_3_)_2_(Ph)­Br] > [Ni­(PPh_3_)_2_(1-Np)­Cl] > [Ni­(PPh_3_)_2_(*o*-Tol)­Cl]. Importantly, ligand evaluation revealed that NHC ligands significantly outperformed phosphine and bipyridine ligands, such as PPh_3_, bpy, and phen, which resulted in little to no reaction under the tested conditions. Interestingly, IPr proved superior to SIPr (99% vs 26%) in this protocol. The method provided an operationally simple approach with an improved efficiency for BHA reaction of 2° aliphatic amines and anilines using an in situ-formed Ni(0)–NHC catalysis system.

**Scheme 156:**
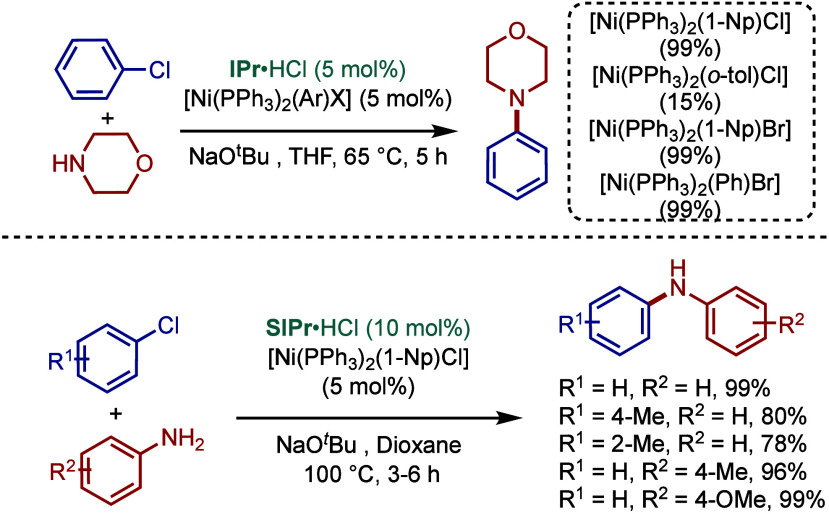
BHA Reaction Catalyzed by In Situ-Formed Ni(0)–NHC Using [Ni­(PPh_3_)_2_(Ar)­X] Complexes by Yang

In 2011, the Yang group extended their Ni(0)–NHC catalyst system to the Buchwald–Hartwig cross-coupling of heteroaryl and aryl chlorides at room temperature (Scheme 157).^304^ They found that [Ni­(PPh_3_)_2_(1-Np)­X] (X = Cl, Br) precursors combined with the imidazolium salt, IPr·HCl, in the presence of KO^*t*^Bu in toluene proved highly effective under mild room temperature conditions, while other Ni precursors, including Ni­(acac)_2_ and [Ni­(PPh_3_)_2_Cl_2_] were completely ineffective under these conditions. The method showed promising scope for cyclic 2° amines, while anilines as well as 2° acyclic and 1° aliphatic amines produced little to no yield.

**Scheme 157:**
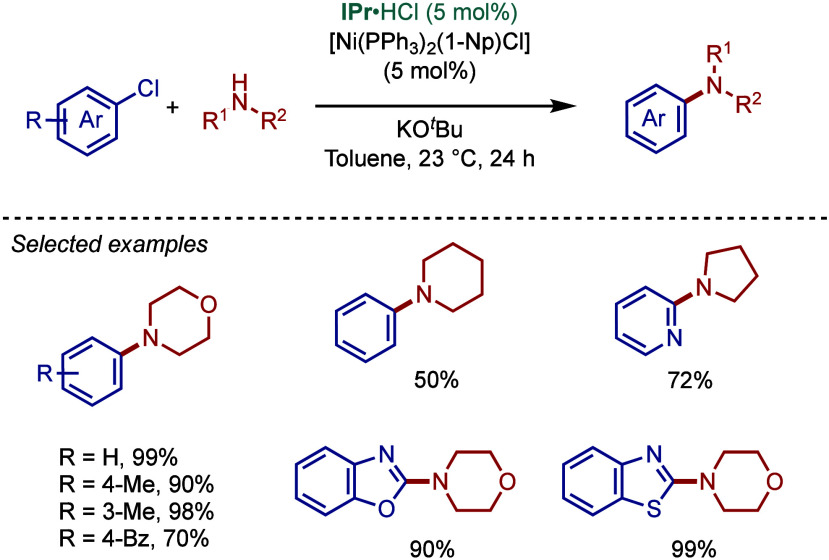
BHA reaction Catalyzed by In Situ-Formed Ni(0)–NHC Using [Ni­(PPh_3_)_2_(Ar)­X] Complexes at Room Temperature by Yang

In 2014, Yang, Fan, and co-workers reported BHA reaction of benzophenone hydrazone with aryl bromides using [Ni­(PPh_3_)_2_Cl_2_] as a precursor to generate Ni(0) in situ in the presence of IPr·HCl and NaO^*t*^Bu in dioxane at 50 °C (Scheme 158).^305^ The authors showed that other Ni sources, such as Ni­(acac)_2_, NiCl_2_, and [Ni­(PPh_3_)_2_(1-Np)­Cl], could also be employed, however, with reduced conversions (73% vs 52–59% yields). In the ligand evaluation, phosphine ligands, such like PCy_3_, dppf as well as bipyridine ligands, such as phen, proved ineffective. This reaction works well with benzophenone hydrazone; however, hydrazine and phenyl hydrazone were unreactive. Aryl chlorides showed negligible activity under these conditions.

**Scheme 158:**
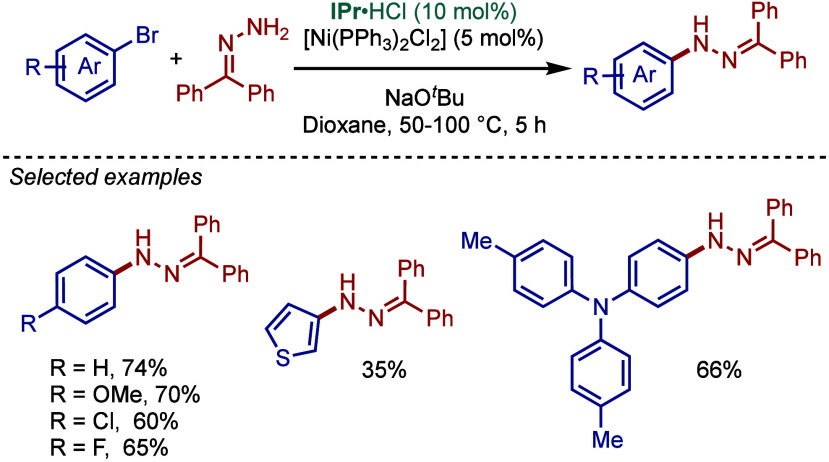
BHA Reaction of Benzophenone Hydrazone Catalyzed by In Situ-Formed Ni(0)–NHC by Yang and Fan

In 2020, the Cornella group reported the synthesis of an air-stable Ni(0)–olefin precatalyst, Ni­(^F^stb)_3_, as an alternative to Ni­(cod)_2_ (Scheme 159).^306^ They demonstrated that the in situ activated Ni­(^F^stb)_3_/SIPr catalyst system was effective in BHA reaction of an activated aryl chloride with morpholine using NaO^*t*^Bu in CPME at 100 °C.

**Scheme 159:**
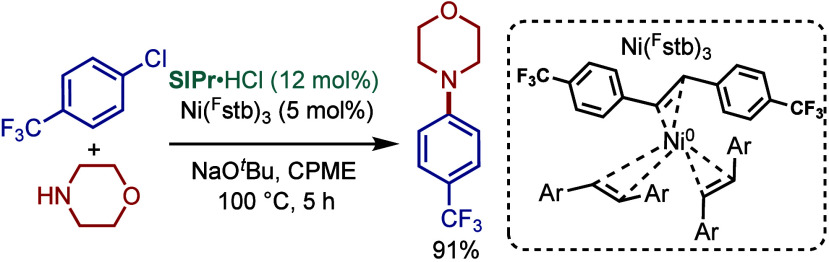
BHA Reaction Catalyzed by In Situ-Formed Ni(0)–NHC Using Well-Defined [Ni(0)­(^F^stb)_3_] Complex by Cornella

In 2021, the Shi group achieved the asymmetric Ni(0)–NHC-catalyzed BHA reaction of racemic 2-alkyl- and 2-aryl-1,2,3,4-tetrahydroquinolines using Ni­(cod)_2_ together with a C_2_-symmetric chiral BIAN–NHC ligand, (*R*,*R*,*R*,*R*)-ANIPE (Scheme 160).^307^ The bulky, asymmetric ANIPE ligand based on an acenaphthoimidazolylidene framework was found to be essential for this BHA reaction. Other nonchiral NHC ligands, such as BIAN–IPr, BIAN–IPr* as well as the imidazol-2-ylidene and imidazolin-2-ylidene counterparts of the ANIPE scaffold (IPE, SIPE) proved ineffective. Similarly, well-known chiral phosphine ligands, such as BINAP, DuanPhos, ^*i*^Pr-DUPHOS and Ph-BPE were unsuccessful. Mechanistic DFT studies revealed substantial changes in the buried volume of the ligand during key steps of the catalytic cycle. The %V_*bur*_ decreased from 58.0% to 56.3% during oxidative addition and increased from 50.6% to 51.8% during reductive elimination. The rate-determining reductive elimination was calculated to have a barrier of 14.7 kcal/mol, while oxidative addition required 8.9 kcal/mol. The scope of this asymmetric amination is remarkably broad with respect to sterically hindered α-branched secondary amines based on a 1,2,3,4-tetrahydroquinoline scaffold. Furthermore, piperazines and dihydrobenzoazepines are effective substrates for this transformation.

**Scheme 160:**
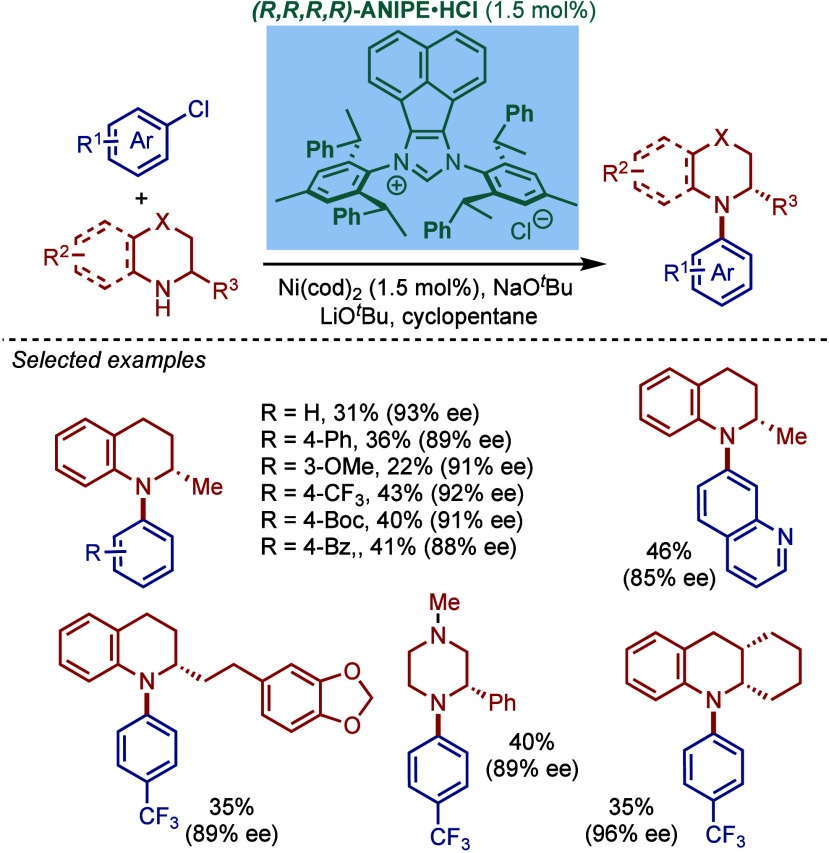
Asymmetric BHA Reaction of α-Branched Secondary Amines Catalyzed by In Situ-Formed Ni(0)–NHC by Shi

Subsequently, in 2023, the same research group expanded the substrate scope of their Ni(0)–NHC-catalyzed BHA reaction to sterically hindered 1° and 2° amines using Ni­(cod)_2_ in the presence of an achiral BIAN–NHC ligand (Scheme 161).^308^ Interestingly, this achiral ligand ANIPE^IPr/IPr^* ligand demonstrated superior efficiency compared to its symmetric counterparts, such as ANIPE^IPr^ and ANIPE^IPr^*, as well as to the chiral (*R*,*R*,*R*,*R*)-ANIPE ligand (70% vs 24–26% and 58%). The classical imidazol-2-ylidene ligand, IPr, was ineffective. This system exhibited a broad substrate scope and excellent compatibility with functional groups for amination of various cyclic and acyclic amines with aryl chlorides. The authors demonstrated successful late-stage functionalization of several pharmaceuticals, such as nornicotine, fenofibrate, and deazapurine.

**Scheme 161:**
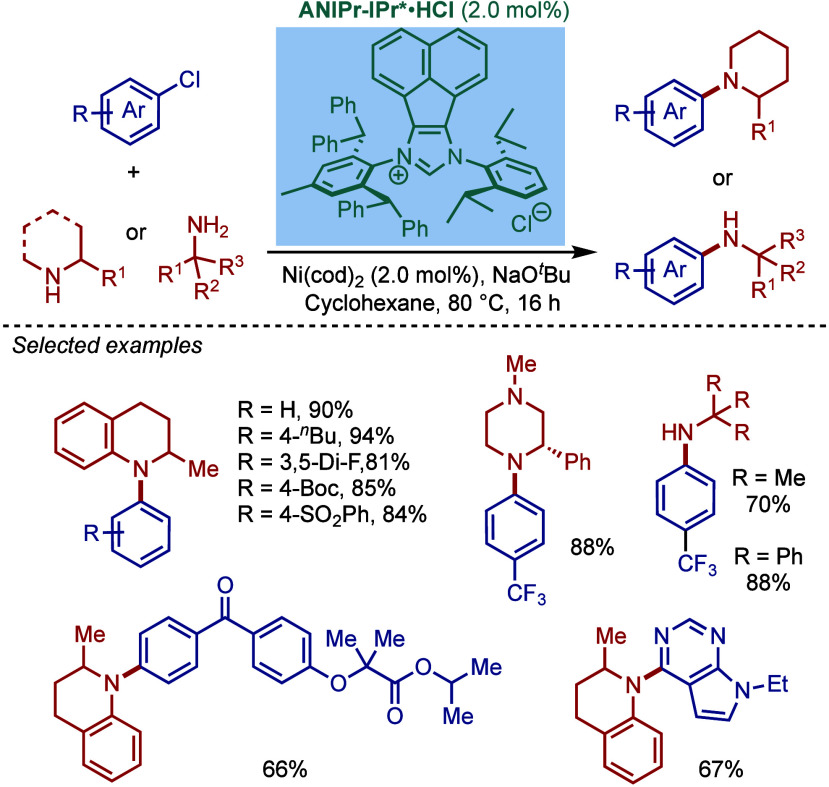
BHA Reaction of Sterically Hindered Amines Catalyzed by In Situ-Formed Ni(0)–NHC by Shi

In 2022, the Stradiotto group demonstrated the application of the Ni­(cod)_2_/IPr system in the BHA reaction of 4-chloro-1,8-naphthalimides with sterically hindered 1° alkylamines (Scheme 162).^309^ Optimization studies revealed that IPr outperformed phosphine ligands, such as dppf, XantPhos, N-Xantphos, DPEPhos, and DalPhos. IPr was also more reactive than its saturated counterpart, SIPr (95% vs 71% yield). This method provides access to 4-amino-1,8-naphthalimides as potential fluorescent probes. The successful cross-coupling of challenging sterically hindered alkylamines at mild room temperature conditions highlights the efficiency of the Ni(0)–NHC catalyst system in BHA reactions.

**Scheme 162:**
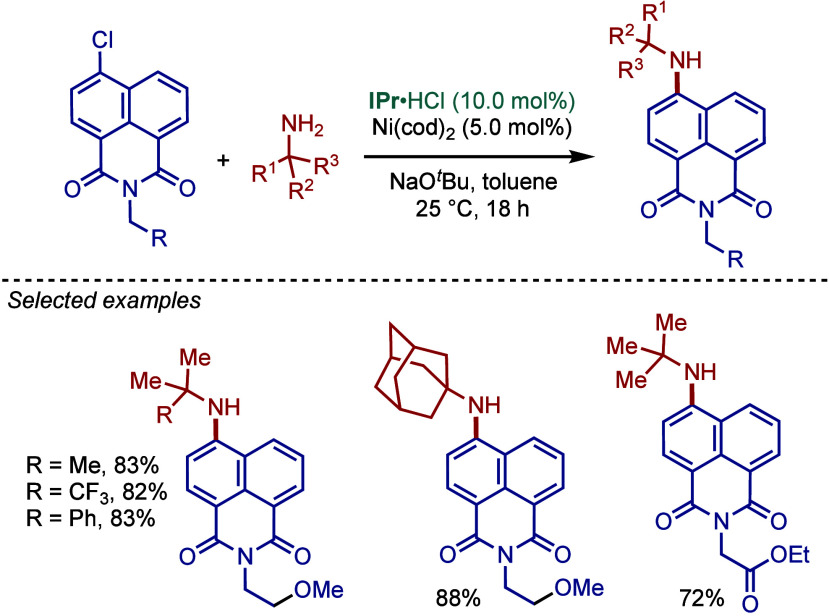
BHA Reaction of 4-Chloro-1,8-Naphthalimides Catalyzed by In Situ-Formed Ni(0)–NHC by Stradiotto

In 2023, Ananikov, Chernyshev, and co-workers reported the use of the air-stable Ni­(II) precursor, [NiCl_2_(py)_2_], for the in situ generation of active Ni(0)–NHC catalysts for BHA reaction using NaO^*t*^Bu in *o*-xylene at 150 °C under aerobic conditions (Scheme 163).^310^ This in situ method outperformed other well-defined [Ni­(II)–NHC] complexes, such as [Ni­(NHC)­(Cp)­Cl], [Ni­(NHC)­(acac)_2_], and [Ni­(NHC)_2_Cl_2_]. A detailed comparison of Ni precursors revealed that [NiCl_2_(py)_2_] showed superior performance over NiCl_2_, Ni­(OAc)_2_, Ni­(acac)_2_, and Ni­(Cp)_2_. Based on previous studies, the authors proposed that *tert*-butoxide or NHC ligands act as reducing agents, facilitating the reduction of Ni­(II) to Ni(0).^121^

**Scheme 163:**
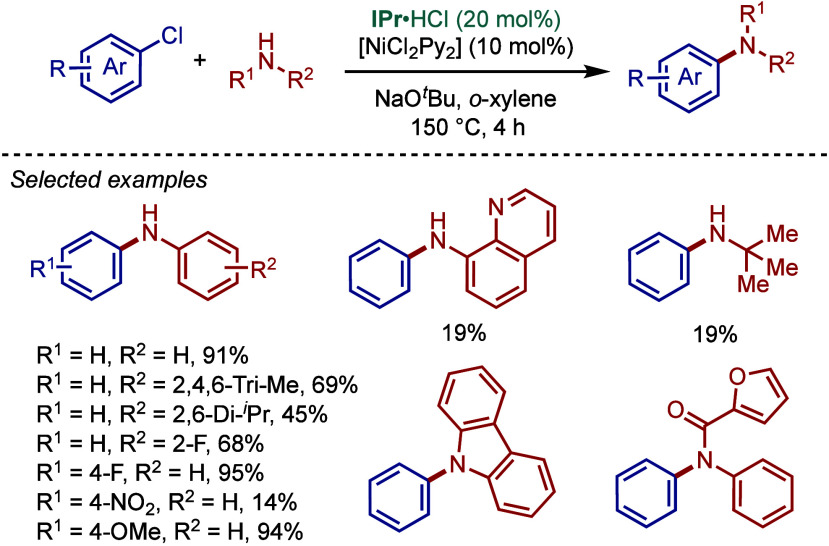
BHA Reaction Catalyzed by In Situ-Formed Ni(0)–NHC Using [NiCl_2_(Py)_2_] Complex by Ananikov

### 3.2 Well-Defined [Ni(0)–NHC] Complexes

#### 3.2.1 [Ni­(NHC)_2_] Complexes

The first well-defined [Ni(0)–NHC] complexes for BHA reaction were reported by the Matsubara group in 2008 (Scheme 164).^311^ They found that [Ni­(NHC)_2_] (NHC = IMes, IPr) could be prepared by the reduction of well-defined [Ni­(NHC)­(acac)_2_] synthesized from Ni­(acac)_2_ and the corresponding NHC salt using an excess of NaH. DFT calculations shoed a higher bond dissociation energy of [Ni­(IPr)_2_] compared to other nickel complexes, such as [NiCl_2_(IPr)_2_] and [NiCl_2_(IPr)­(PPh_3_)] (53 kcal/mol vs 37 and 47 kcal/mol), in agreement with high thermal stability of this complex at room temperature. The authors found that this [Ni­(IPr)_2_] complex showed improved catalytic activity in the BHA reaction of chlorobenzene with aniline using NaO^*t*^Bu in dioxane at 100 °C compared to the initial Fort’s in situ-generated system (see Scheme 152).^86^

**Scheme 164:**
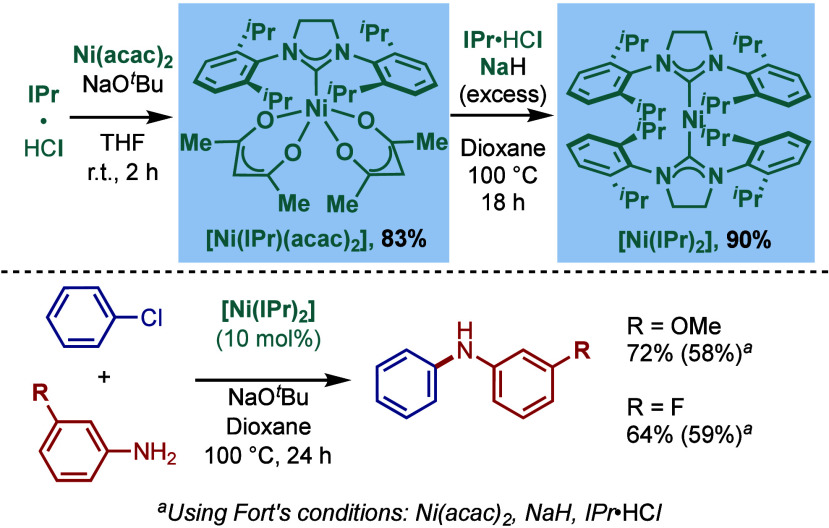
BHA Reaction Catalyzed by Well-Defined [Ni­(NHC)_2_] Complexes by Matsubara

a Using Fort’s conditions: Ni­(acac)_2_, NaH, IPr•HCl.

#### 3.2.2 [Ni­(NHC)­(sty)_2_] Complexes

In 2012, Nicasio, Belderrain, and co-workers reported the synthesis and application in the BHA reaction of aryl tosylates of a highly reactive [Ni­(IPr)­(styrene)_2_] complex (*vide infra*, see Scheme 182).^312^ This stable, well-defined [Ni(0)–NHC] served promoted the amination of a variety of aryl tosylates with cyclic aliphatic amines and anilines using LiO*t*Bu in dioxane at 110 °C. In 2015, the same group demonstrated the high activity of the same Ni(0)–NHC catalyst in a challenging BHA reaction of indoles and carbazoles (Scheme 165A).^313^ This [Ni­(IPr)­(styrene)_2_] complex showed a broad substrate scope for N-arylation with aryl chlorides using LiO*t*Bu in dioxane at 110 °C and offered an attractive alternative to the Pd/phosphine-based systems.^314^ Notably, the optimized conditions required LiO*t*Bu, while NaO^*t*^Bu gave negligible yields. In 2018, the same group reported mechanistic studies on this [Ni(0)–NHC] BHA reaction catalysis platform (Scheme 165B).^315^ Using a model system, they found that the oxidative addition of 2-chloropyridine to ([Ni­(IPr)­(sty)_2_] and [Ni­(IPr)­(η^6^-Tol)]) proceeds at room temperature, leading to the formation of mononuclear and dinuclear pyridyl–Ni­(II) in a 2:3 ratio. These complexes were active in promoting the C–N amination of indole under the standard conditions, validating the originally proposed Ni(0)/Ni­(II) cycle.

**Scheme 165:**
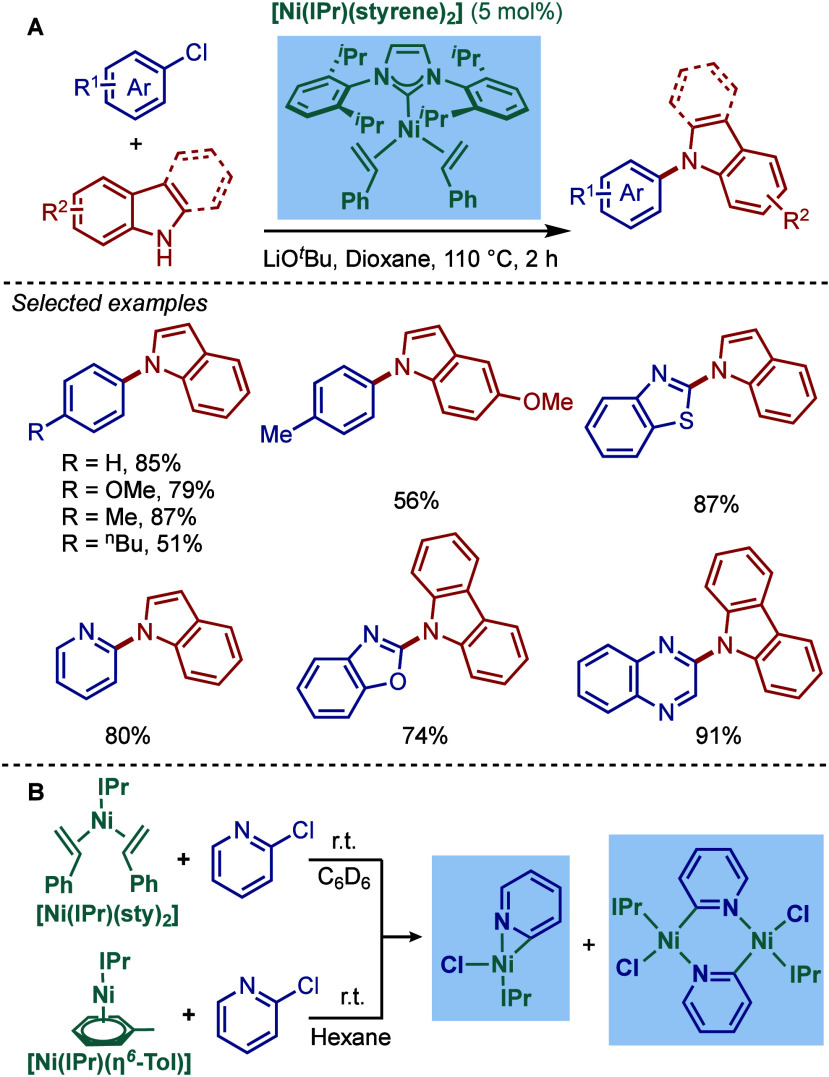
BHA Reaction of Indoles and Carbazoles Catalyzed by Well-Defined [Ni­(NHC)­(sty)_2_] Complexes by Nicasio

#### 3.2.3 [Ni­(NHC)­(acr/fum)_2_] Complexes

In 2018, the Montgomery group reported Ni(0)–NHC complexes stabilized by electron-withdrawing acrylate (acr) and fumarate (fum) ligands (Scheme 166).^316^ These [Ni­(NHC)­(acr/fum)_2_] complexes (NHC = IMes, IPr, SIPr, IPr*^MeO^) were synthesized in a single step from Ni­(cod)_2_, acr/fum and the corresponding NHC ligands. Notably, the complexes displayed remarkable air stability compared to other Ni(0)–NHC complexes. The authors extensively evaluated the reactivity of these [Ni­(NHC)­(acr/fum)_2_] catalysts in the BHA reaction of a model piperidine with an activated aryl chloride using NaO^*t*^Bu in THF at 60 °C. They found that among the complexes featuring IMes, IPr, SIPr and IPr*^OMe^, the most sterically demanding IPr*^OMe^ showed the highest reactivity, while IPr and SIPr led to significant protodechlorination products. The optimized conditions were applied to the BHA reaction of aryl chlorides with 1° and 2° aliphatic amines and anilines at only 1 mol % catalyst loading at 60 °C. This represents the highest reactivity compared with similar Ni–NHC systems.

**Scheme 166:**
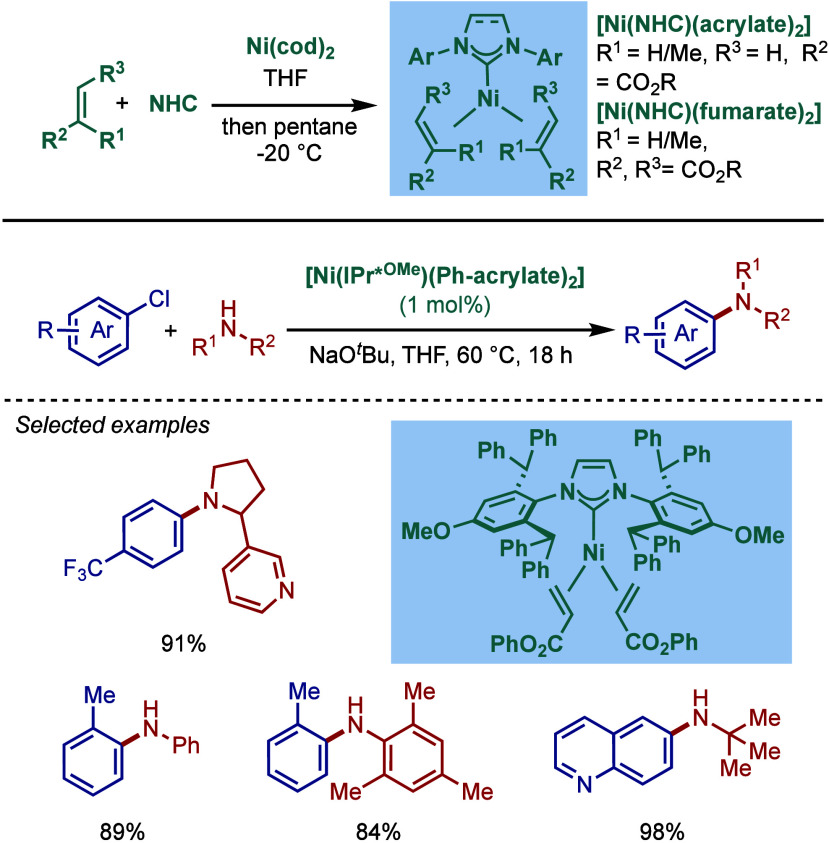
BHA Reaction Catalyzed by Well-Defined [Ni­(NHC)­(olefin)_2_] Complexes by Montgomery

### 3.3 Well-Defined [Ni­(II)–NHC] Complexes

#### 3.3.1 [Ni­(NHC)­(Cp)­Cl] Complexes

In 2005, the Nolan group reported the first application of well-defined [Ni­(II)–NHC] complexes based on the [Ni­(NHC)­(Cp)­Cl] architecture (Cp = cyclopentadienyl) in BHA reaction of aryl chlorides and aryl bromides (Scheme 167).^317^ These [Ni­(NHC)­(Cp)­Cl] complexes (NHC = IPr, SIPr, IMes, SIMes) were synthesized directly from imidazolium salts in the presence of nickelocene, NiCp_2_, in THF at reflux. In contrast to the in situ-formed Ni(0)–NHC systems, these well-defined [Ni­(II)–NHC] complexes exhibit a precise 1:1 Ni/NHC ratio and are air- and moisture-stable in both solid state and solution. The BHA reaction using [Ni­(NHC)­(Cp)­Cl] proceeded in high yields using KO^*t*^Bu in dioxane at 105 °C, with the following order of reactivity: SIPr > IPr > SIMes > IMes. It should be note that these cyclopentadienyl complexes required higher temperature for cross-coupling than the in situ system established by Fort (see Scheme 152) due to slower activation to the active Ni(0)–NHC catalyst.

**Scheme 167:**
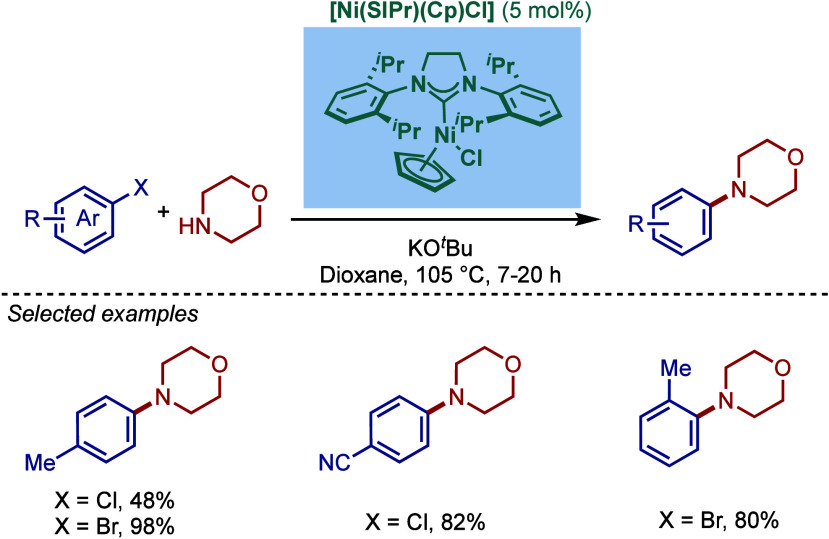
BHA Reaction Catalyzed by Well-Defined [Ni­(NHC)­(Cp)­Cl] Complexes by Nolan

In 2013, Nolan and co-workers investigated the activity of more sterically demanding well-defined [Ni­(NHC)­(Cp)­Cl] complexes in BHA reaction of aryl chlorides (Scheme 168).^318^ Notably, bulky NHC ligands based on a bulky-yet-flexible IPr* scaffold, such as IPr*, IPr*^Tol^, and IPr*^OMe^, were found to significantly enhance the catalytic performance compared to the less sterically hindered IMes, IPr, and IPent congeners in the model amination of 4-chlorotoluene with morpholine using KO^*t*^Bu in dioxane at reflux (51–90% vs 17–28% yields). The authors proposed that the increase in reactivity is due to enhanced stabilization of the Ni(0)–NHC active species. Among the complexes studied, the bulky and electron-rich [Ni­(IPr*^OMe^)­(Cp)­Cl] was identified as the most reactive complex. Furthermore, the effect of counterions was evaluated with the following order of reactivity: [Ni­(IPr*^OMe^)­(Cp)­Cl] > [Ni­(IPr*^OMe^)­(Cp)­(MeCN)­(PF_6_)] > [Ni­(IPr*^OMe^)­(Cp)­Br] > [Ni­(IPr*^OMe^)­(Cp)­I].

**Scheme 168:**
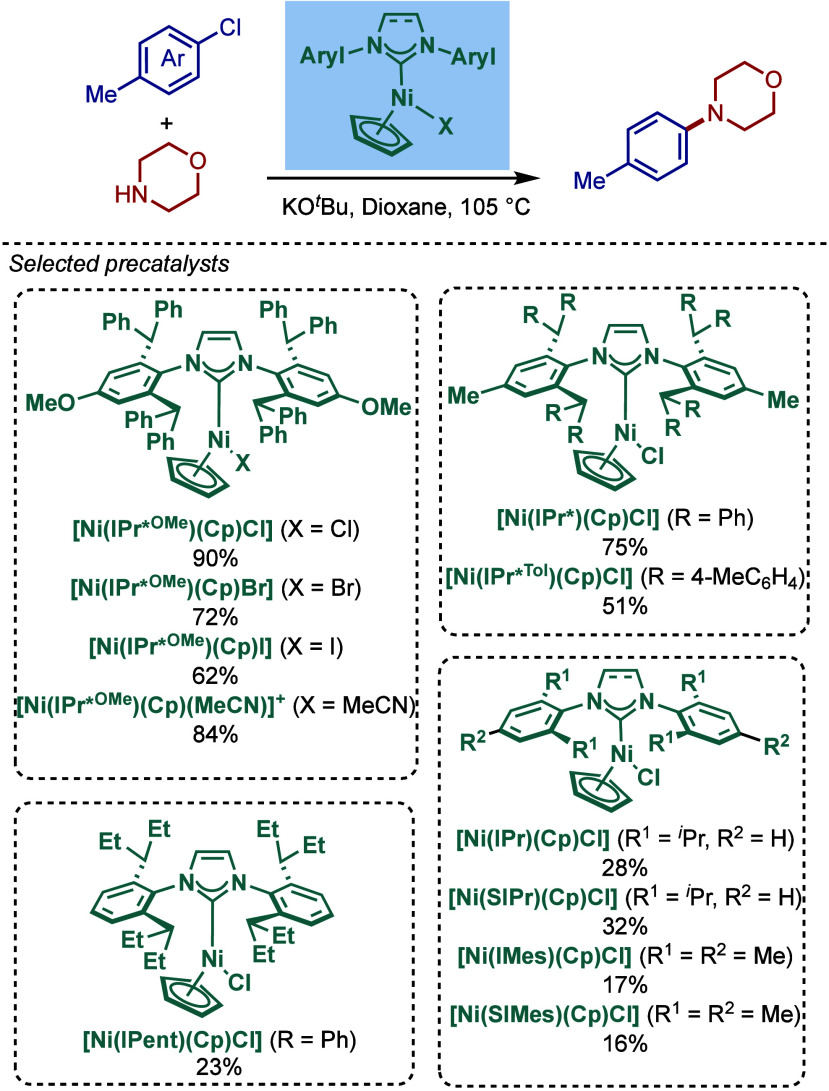
BHA Reaction Catalyzed by Well-Defined [Ni­(NHC)­(Cp)­Cl] Complexes with Sterically Hindered Ligands by Nolan

#### 3.3.2 [Ni­(NHC)­(PR_3_)­Cl_2_] Complexes

In 2007, Matsubara and co-workers reported the application of a mixed PPh_3_/NHC Ni­(II) complex, [Ni­(IPr)­(PPh_3_)­Cl_2_], in BHA reaction of aryl bromides (Scheme 169).^319^ This well-defined, air-stable Ni­(II) complex was readily prepared by the ligand displacement from [Ni­(PPh_3_)_2_Cl_2_] using IPr carbene in THF at room temperature (not shown).^320^ This complex catalyzed Buchwald–Hartwig cross-coupling of aryl bromides with cyclic 2° amines and 1° anilines using NaO^*t*^Bu in toluene at 100 °C Interestingly, Matsubara’s system favors the amination of less basic arylamines, which proceeded more efficiently than the amination of more basic alkylamines. However, this system is largely restricted to aryl bromides, with only one example of an activated chloride, 4-PhCO-C_6_H_4_-Cl, showing moderate reactivity under the developed conditions.

**Scheme 169:**
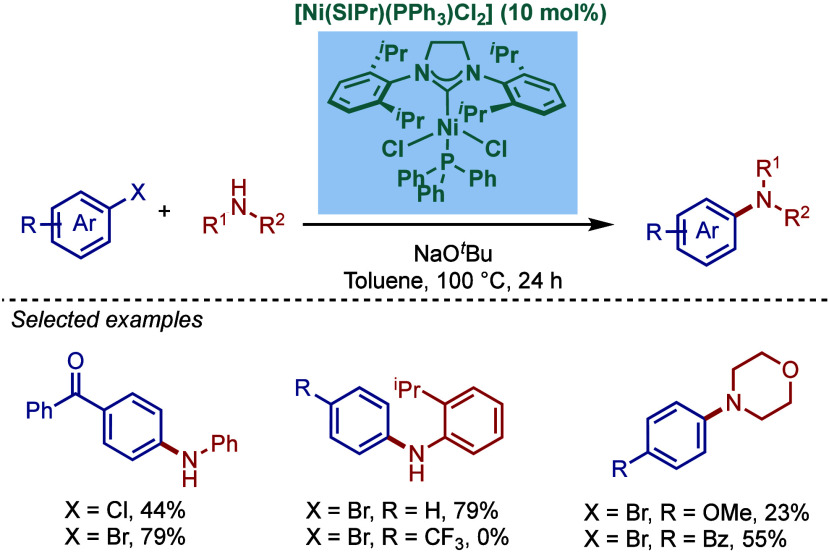
BHA Reaction Catalyzed by Well-Defined [Ni­(NHC)­(PR_3_)­Cl_2_] Complexes by Matsubara

#### 3.3.3 [Ni­(NHC)­(η^3^-allyl)­Cl] Complexes

In 2010, the Nicasio group applied well-defined [Ni­(NHC)­(η^3^-allyl)­Cl] complexes in BHA reaction of aryl chlorides (Scheme 170).^321^ These Ni­(II)–NHC complexes are accessible from Ni­(cod)_2_, allyl chloride, and NHC ligands; however, in most cases they have been shown to react with oxygen.^322^ The authors tested various complexes (NHC = IMes, IPr, SIPr) in the model amination of 4-chlorotoluene with morpholine using NaO^*t*^Bu in THF at room temperature. Among these catalysts, [Ni­(IPr)­(η^3^-allyl)­Cl] showed the highest reactivity (98% yield), while [Ni­(SIPr)­(η^3^-allyl)­Cl] and [Ni­(IMes)­(η^3^-allyl)­Cl] (71% and 5%) were less reactive. The authors attributed the high activity of [Ni­(IPr)­(η^3^-allyl)­Cl] to the facile activation of the catalytically active Ni(0)–NHC species, enabling room-temperature BHA reaction. The developed conditions were applied to the amination of a range of aryl and heteroaryl chlorides with cyclic and acyclic 2° amines and anilines at room temperature, demonstrating broad substrate compatibility.

**Scheme 170:**
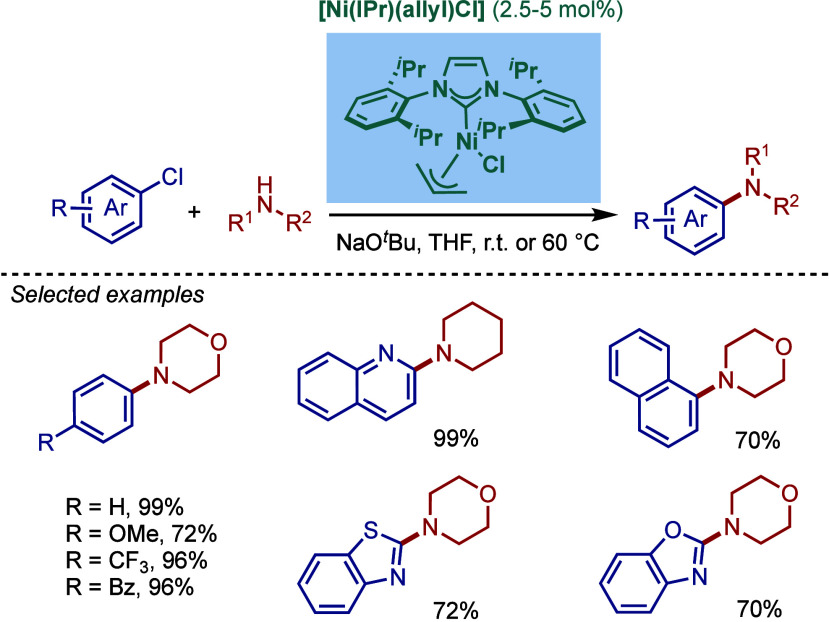
BHA Reaction Catalyzed by Well-Defined [Ni­(NHC)­(η^3^-allyl)­Cl] Complexes by Nicasio

In 2014, Nolan and co-workers reported two new [Ni­(NHC)­(η^3^-allyl)­Cl] complexes featuring sterically demanding NHC ligands, IPr* and IPr*^OMe^, and evaluated their activity in the BHA reaction of aryl chlorides (Scheme 171).^323^ The synthesis proceeded readily from Ni­(cod)_2_, allyl chloride and NHC ligands. These sterically demanding complexes showed improved reactivity compared to [Ni­(NHC)­(Cp)­Cl] complexes reported by the same group (see Scheme 168).^196^ The IPr*^OMe^ complex proved significantly more reactive than the IPr* counterpart in the model amination of 4-chloroanisole with morpholine using NaO^*t*^Bu in THF at 60 °C (52% vs 0% at 1 mol % loading). This catalyst showed high reactivity in amination of chlorarenes with cyclic 2° amines and anilines; however, acyclic amines were unreactive.

**Scheme 171:**
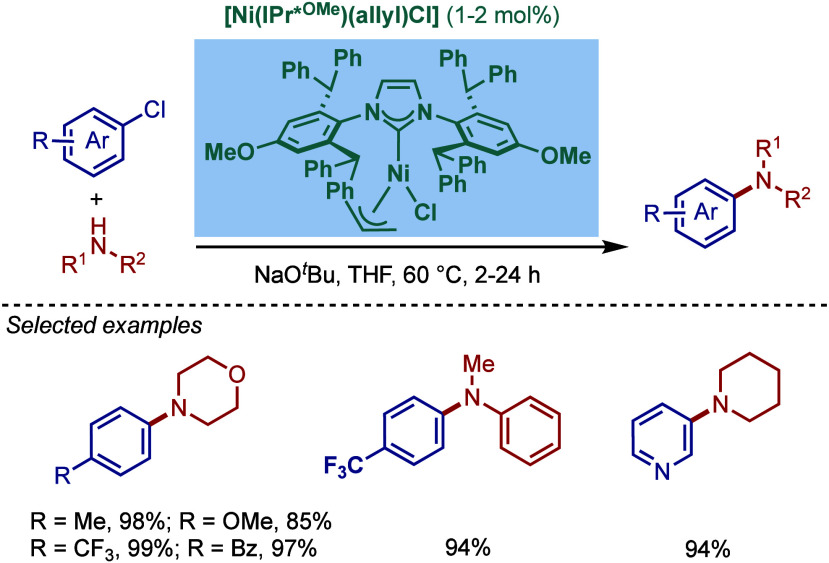
BHA Reaction Catalyzed by Well-Defined [Ni­(NHC)­(η^3^-allyl)­Cl] Complexes with Sterically Hindered Ligands by Nolan

#### 3.3.4 [Ni­(NHC)_2_X_2_] Complexes

In 2015, Viswanathamurthi and co-workers reported the synthesis and application in BHA reaction of a methylene-bridged cyclic tetradentate [Ni­(NHC)_2_X_2_] complex based on a bis­(aryloxy–NHC) (Scheme 172).^324^ This air-stable [Ni­(II)–NHC] complex was synthesized by complexation of Ni­(OAc)_2_·4H_2_O or [Ni­(PPh_3_)_2_Cl_2_] with the bis­(imidazolium) precursor in the presence of Et_3_N in EtOH. The complex demonstrated excellent efficiency in BHA reaction of aryl chlorides at a 1 mol % catalyst loading using KO^*t*^Bu in dioxane at 90 °C. The authors proposed that the high activity was due to the steric and electronic stabilization by the carbene ligand with hemilabile oxygen donors. The complex showed broad substrate compatibility, effectively coupling 1° and 2° aliphatic and aromatic amines with aryl and heteroaryl chlorides.

**Scheme 172:**
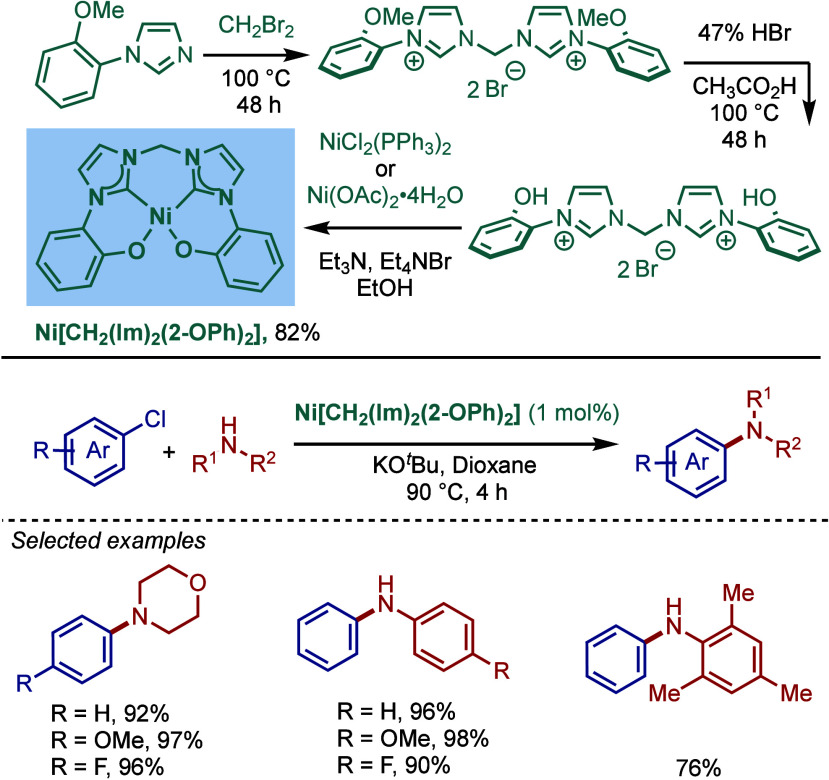
BHA Reaction Catalyzed by [Ni­(NHC)_2_X_2_] Complexes by Viswanathamurthi

In 2016, Bala and co-workers reported [Ni­(NHC)_2_X_2_] complexes with chelating 2-picolyl groups and applied them in BHA reaction of aryl bromides (Scheme 173).^325^ Complexes featuring three different N-wingtip substituents (R = 4-C_6_H_4_NO_2_, allyl, butenyl) were synthesized by carbene transfer from the corresponding Ag­(I)–NHC precursors using [Ni­(PPh_3_)_2_Cl_2_] and were found to be moderately stable to air and moisture. Catalytic studies in model BHA reaction of bromobenzene with aniline using KO^*t*^Bu in THF at reflux revealed that the complex with R = 4-C_6_H_4_NO_2_ was the most reactive. Compared to the in situ catalyst generation method (NiCl_2_/NHC·HBr) with the same ligand, these well-defined NI­(II)–NHC complexes showed improved catalytic efficiency. The catalytic system was applied to the amination of aryl bromides with anilines, affording the products in generally good yields.

**Scheme 173:**
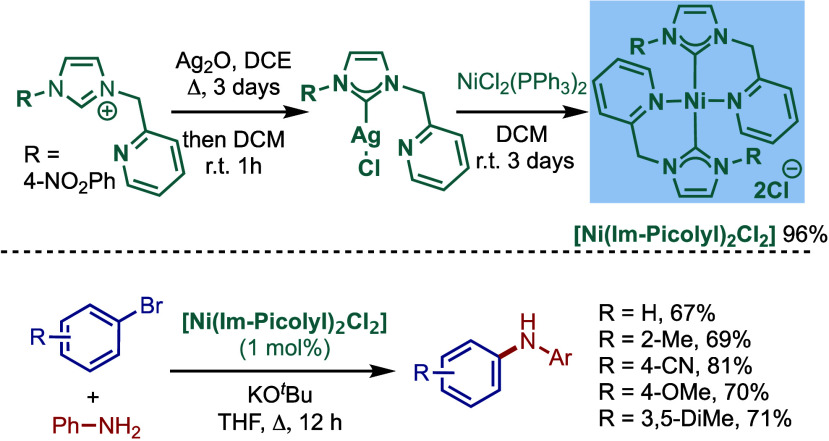
BHA Reaction Catalyzed by [Ni­(NHC)_2_X_2_] Complexes by Bala

#### 3.3.5 [Ni­(NHC)­(η^3^-allyl)­(PR_3_)]­X Complexes

In 2017, Nicasio, Belderrain, Fructos, and co-workers reported the synthesis of well-defined [Ni­(NHC)­(η^3^-allyl)­(PR_3_)]­X complexes featuring bidentate phosphine-functionalized N-heterocyclic carbenes (Scheme 174).^326^ These cationic Ni­(II)–NHC complexes were synthesized from unsymmetrical NHC salts bearing an ethylene diphenylphosphine side chain and an aryl wingtip (Ar = Mes, Dipp) by deprotonation with KHMDs followed by complexation with [Ni­(allyl)­Cl]_2_. The resulting complexes were found to be stable in air and solution. Moreover, the imidazolium precursors were reacted with Ni­(cod)_2_ and alkenes under basic conditions to generate well-defined but air-sensitive [Ni­(NHC)­(alkene)] complexes (alkene = styrene, fumarate). Comparative studies in BHA reaction of activated heterocyclic chlorides, such as 2-chloropyridine and 2-chloroquinoline, using NaO^*t*^Bu in dioxane at 110 °C revealed similar catalytic performance between these two Ni–NHC systems.

**Scheme 174:**
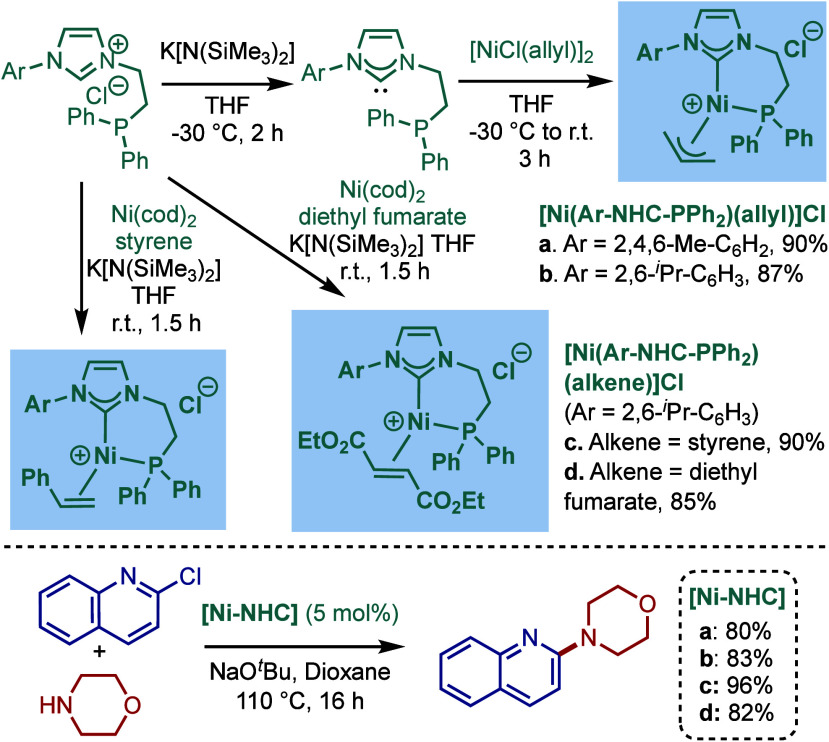
BHA Reaction Catalyzed by [Ni­(NHC)­(η^3^-allyl)­(PR_3_)]­X Complexes by Nicasio, Belderrain and Fructos

#### 3.3.6 [Ni­(NHC)_4_]­X_2_ Complexes

In 2017, Viswanathamurthi and co-workers reported new four-coordinate homoleptic [Ni­(NHC)_4_]­Br_2_ complexes featuring methylene-bridged bis-NHC chelated to the nickel center (Scheme 175).^327^ These [Ni­(NHC)_4_]­Br_2_ complexes (NHC = R-Im_2_-CH_2_-Im_2_-R, R = Me, Ph) were synthesized from the corresponding bis-imidazolium ligands and Ni­(OAc)_2_ in EtOH. Both complexes were found to be air- and moisture-stable. Evaluation in BHA reaction using KO^*t*^Bu in dioxane at 90 °C revealed that different wingtip substituents had a negligible effect on the cross-coupling efficiency. This catalytic system was applied to the amination of aryl chlorides with anilines and 1° and 2° aliphatic amines, offering an improvement over the previous system developed by the same group (see Scheme 172).^200^

**Scheme 175:**
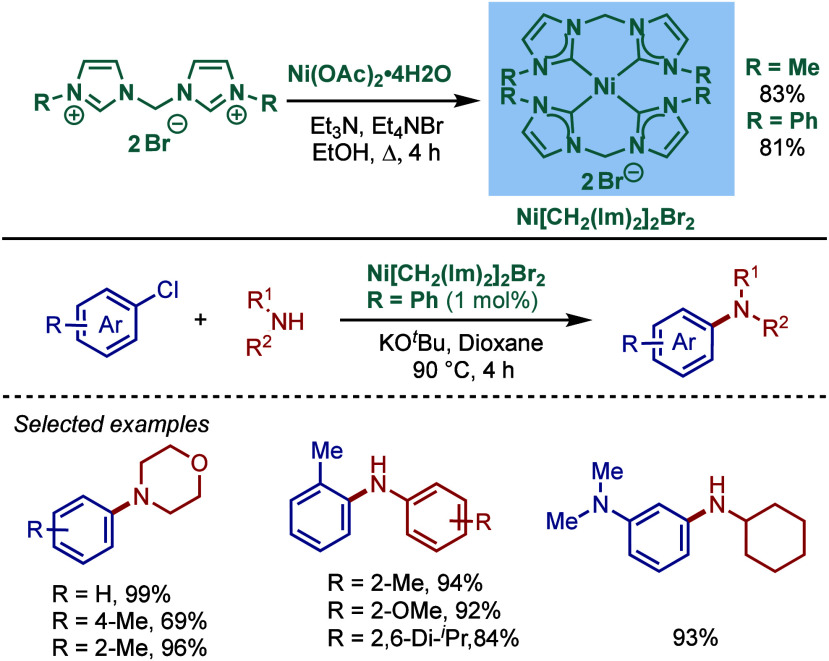
BHA Reaction Catalyzed by [Ni­(NHC)_4_]­X_2_ Complexes by Viswanathamurthi

#### 3.3.7 [Ni­(NHC)­(R)­Cl] Complexes

In 2018, the Jamison group reported a series of bench-stable [Ni­(NHC)­(Ar)­Cl] complexes and demonstrated their activity in BHA reaction of aryl chlorides (Scheme 176).^328^ These complexes feature a labile amine that stabilizes Ni­(II) and a pendant olefin that facilitates activation to the active Ni(0)–NHC by an intramolecular Heck reaction. The complexes were synthesized by a direct oxidative addition of *ortho*-substituted chlorobenzenes with Ni­(cod)_2_ in the presence of free NHCs. The complex featuring a tethered piperidine was identified as the most reactive for the BHA reaction of chlorobenzene with indole in the presence of LiO^*t*^Bu in dioxane at 110 °C. This well-defined [Ni­(II)–NHC] complex also efficiently catalyzed carbonyl-ene reactions, exhibiting greater stability than the in situ-formed Ni­(cod)_2_/IPr system.

**Scheme 176:**
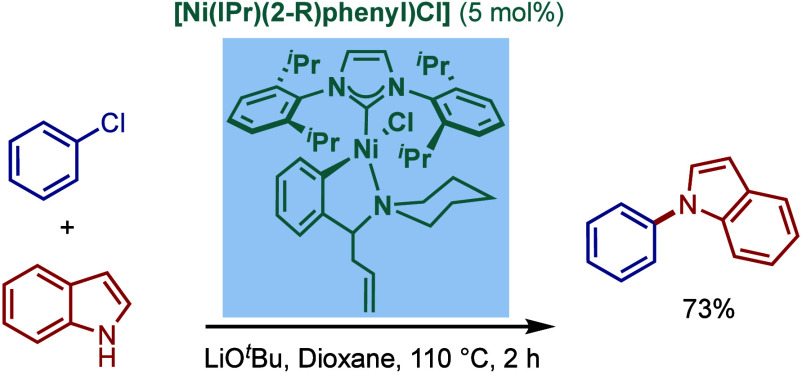
BHA Reaction Catalyzed by [Ni­(NHC)­(R)­Cl] Complexes by Jamison

### 3.4 Well-Defined [Ni­(I)–NHC] Complexes

#### 3.4.1 [(NHC)­Ni­(μ-X)]_2_ Complexes

In 2016, the Matsubara group reported the application of a dinuclear [Ni­(IPr)­(μ-Cl)]_2_ complex in Buchwald–Hartwig amination of an activated aryl bromide with diphenylamine (Scheme 177).^329^ The same group earlier studied related [Ni­(NHC)­(μ-X)]_2_ complexes in complexation with phosphines (see Scheme 179).^330^ They found that the catalytic activity of [Ni­(IPr)­(μ-Cl)]_2_ in BHA reaction can be influenced by the addition of another ligand, such as PPh_3_, P­(OPh)_3_, and pyridine. The most active were PPh_3_ (99% yield) and pyridine (98% yield), while P­(OPh)_3_ had a deleterious effect (39% yield vs 80% control). The authors proposed that the dinuclear [Ni­(IPr)­(μ-Cl)]_2_ serves as a precursor to the catalytically active monomeric nickel­(I) complexes, [Ni­(IPr)­(L)­Cl], which was established based on NMR, SQUID, and X-ray crystallographic studies. It should be noted that this is a rare example of a proposed Ni­(I)/Ni­(III) mechanism instead of the more common Ni(0)/Ni­(II) cycle.

**Scheme 177:**
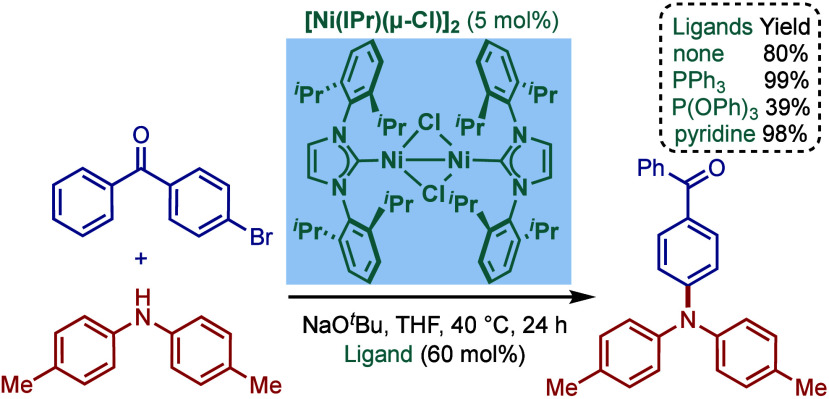
BHA Reaction Catalyzed by [Ni­(NHC)­(μ-Cl)]_2_ Complexes by Matsubara

In 2018, Nagahora and co-workers reported BHA reaction of a bromophenylphosphinine catalyzed by the dinuclear [Ni­(IPr)­(μ-Br)]_2_ complex (Scheme 178).^331^ This challenging amination in the presence of coordinating phosphine proceeded successfully using NaO^*t*^Bu in toluene at 80 °C. It is noteworthy that PPh_3_ (25 mol %) as an additive was critical for this reaction, implying the formation of [Ni­(IPr)­(PPh_3_)­Cl] as the active catalytic species. The scope of this interesting reaction is quite broad encompassing 1° and 2° aromatic and aliphatic amines, albeit in generally modest yields. Furthermore, the authors demonstrated that other widely used Pd- and Ni-catalyzed systems with phosphine or NHC ligands, such as PPh_3_, dppf, CyJohnPhos, and Pd–PEPPSI–IPr, failed to promote this cross-coupling.

**Scheme 178:**
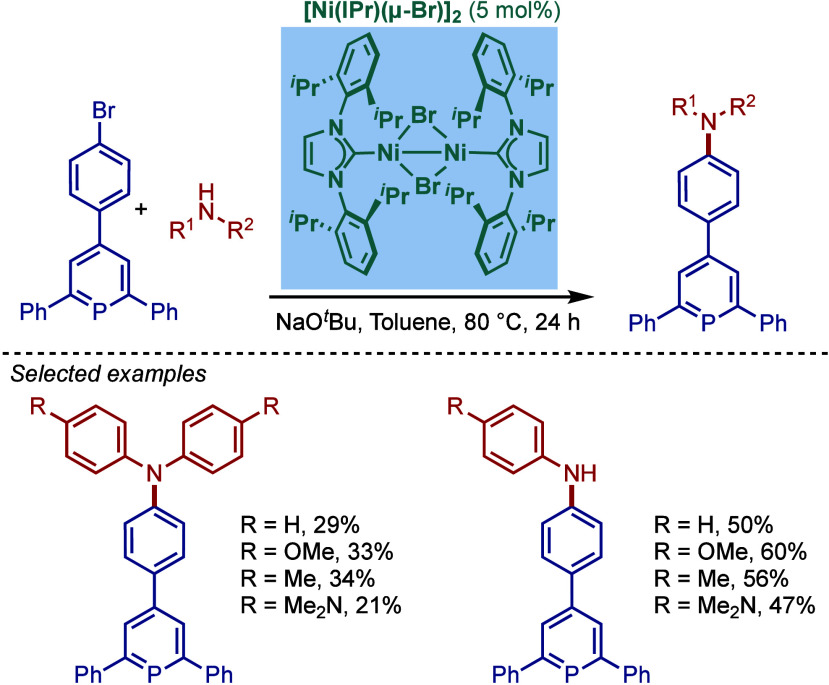
BHA Reaction of Aryl Bromophenylphosphinines Catalyzed by [Ni­(NHC)­(μ-Br)]_2_ Complexes by Nagahora

#### 3.4.2 [Ni­(NHC)­(PR_3_)­Cl] Complexes

In 2011, the Matsubara group reported the synthesis of a monomeric [Ni­(IPr)­(PPh_3_)­Cl] complex by the reaction of dinuclear [Ni­(IPr)­(μ-Cl)]_2_ with PPh_3_ at −30 °C (Scheme 179).^330^ The reactivity of [Ni­(IPr)­(PPh_3_)­Cl] in BHA reaction was found to be higher than that of its dimeric precursor. Notably, the authors found that a catalyst system consisting of [Ni­(IPr)­(PPh_3_)­Cl] (5 mol %) in the presence of PPh_3_ (25 mol %) proved effective in BHA reaction of unactivated aryl halides using NaO^*t*^Bu in toluene at 70 °C (ArI: 77% yield; ArBr: 74% yield; ArCl: 41% yield).

**Scheme 179:**
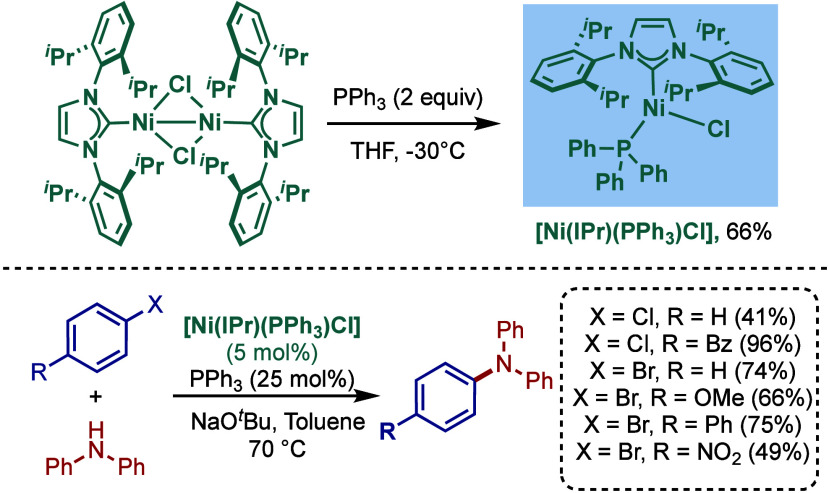
BHA Reaction Catalyzed by [Ni­(NHC)­(PR_3_)­Cl] Complexes by Matsubara

#### 3.4.3 [Ni­(NHC)­(bpy)­X] Complexes

In 2019, Matsubara and co-workers reported the synthesis of monomeric [Ni­(IPr)­(bpy)­X] complexes (X = Cl, Br) derived from complexation of dimeric [Ni­(IPr)­(μ-X)]_2_ with 2,2′-bipyridine (Scheme 180).^332^ These complexes were found to be relatively air-stable compared to other [Ni­(I)–NHC], permitting handling in air for several minutes. Evaluation of [Ni­(IPr)­(bpy)­X] in BHA reaction revealed that the scope is mostly limited to electronically activated aryl bromides using NaO^*t*^Bu in THF at 40 °C. Based on studies with an isolated [Ni­(IPr)­(NPh_2_)] intermediate, the authors proposed a Ni­(I)/Ni­(III) mechanism with bpy acting as a hemilabile ligand to the Ni­(I) center.

**Scheme 180:**
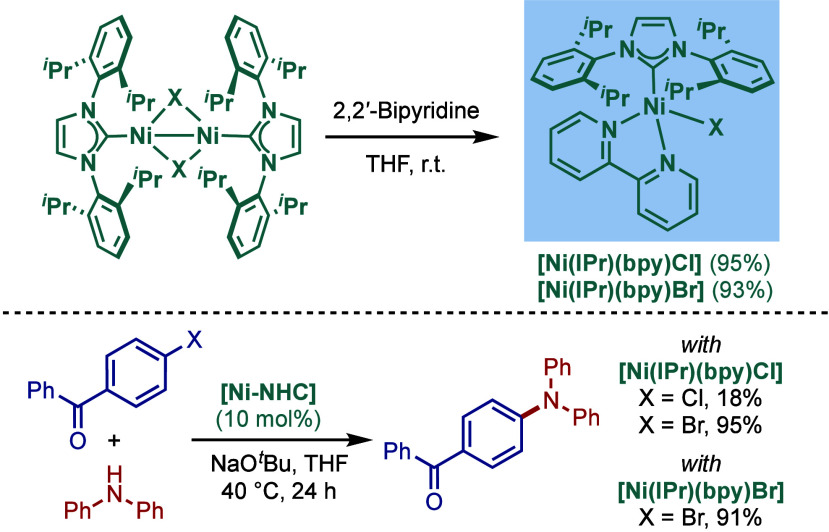
BHA Reaction Catalyzed by [Ni­(NHC)­(bpy)­X] Complexes by Matsubara

### 3.5 BHA Reaction of Pseudohalides (C–OTs)

In 2008, the Yang group reported the first BHA reaction of aryl tosylates using a [Ni­(PPh_3_)_2_(1-Np)­Cl]/IPr·HCl system (Scheme 181).^333^ The catalytically active Ni(0)–NHC species was generated in situ using NaO^*t*^Bu in dioxane at 110 °C, in analogy to their previous [Ni­(aryl)­(PPh_3_)_2_X]/NHC system for the BHA reaction of aryl chlorides (see Scheme 156).^184^ The activity of Ni­(II) precursors was in the following order: [Ni­(PPh_3_)_2_(1-Np)­X] > [Ni­(PPh_3_)_2_(4-Ac-1-Np)­Cl] > [Ni­(PPh_3_)_2_(Ph)­X]. Furthermore, imidazol-2-ylidene-based IPr was significantly more effective than its saturated imidazolin-2-ylidene SIPr counterpart (80% vs 10% yield), while a phosphine ligand PPh_3_ was completely ineffective. The scope of this amination was quite limited, with electron-rich tosylates unreactive and sterically hindered anilines yielding poorly. However, the method provided the first precedent for the BHA reaction with cyclic 2° aliphatic amines and anilines by C–O activation.

**Scheme 181:**
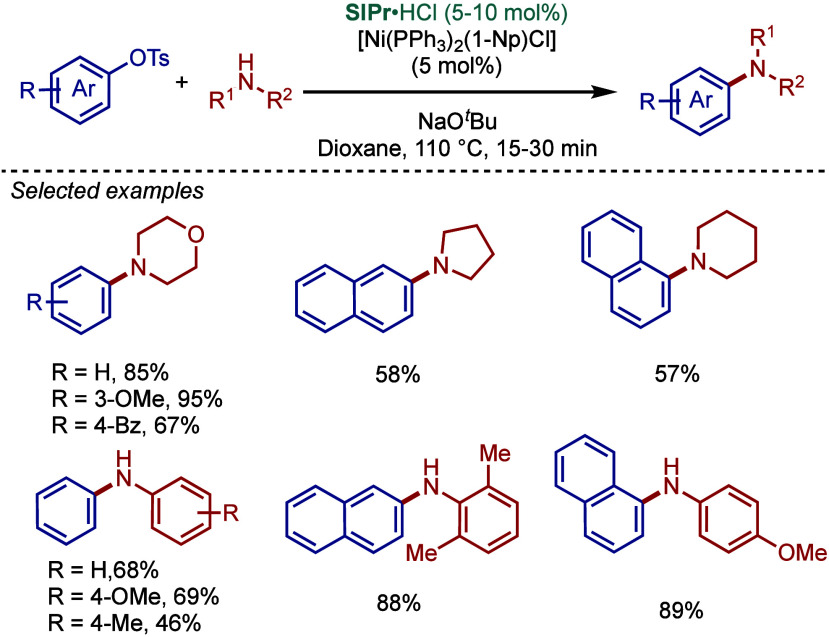
BHA Reaction of Aryl Tosylates Catalyzed by Ni–NHC Complexes by Yang

In 2012, Nicasio, Belderrain, and colleagues introduced a well-defined [Ni­(IPr)­(styrene)_2_] complex for BHA reaction of aryl tosylates (Scheme 182).^312^ This [Ni(0)–NHC] complex was synthesized from by complexation of Ni­(cod)_2_ with free IPr in the presence of styrene. Under the optimized conditions using LiO*t*Bu in dioxane at 110 °C, this catalyst promoted the BHA reaction of phenyl tosylate with morpholine in 90% yield within 15 min, outperforming the well-established [Ni­(II)–NHC] precatalyst, [Ni­(IPr)­(allyl)­Cl] (40% yield). This catalytic system demonstrated excellent yields in the cross-coupling with cyclic 2° amines and anilines.

**Scheme 182:**
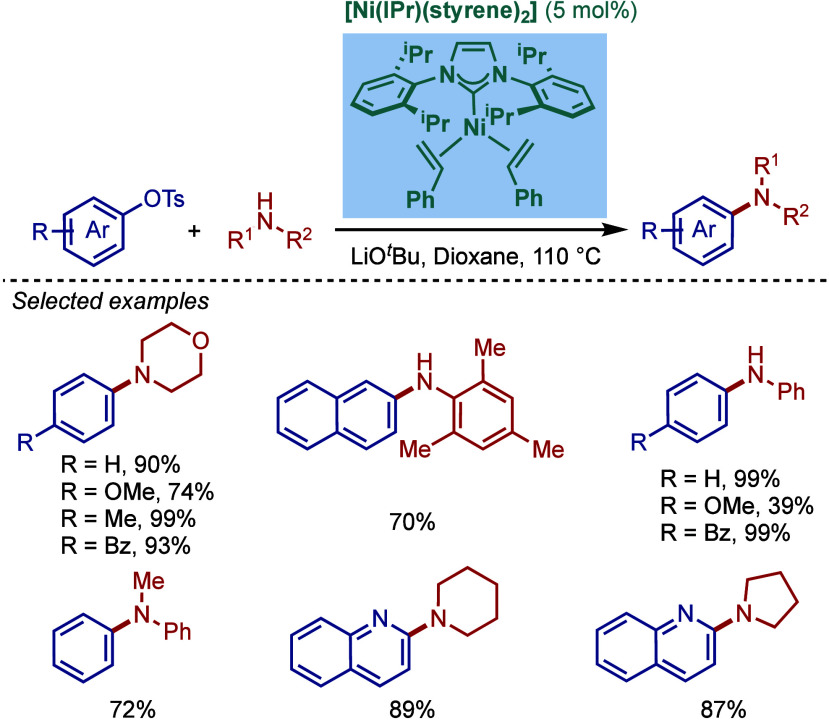
BHA Reaction of Aryl Tosylates Catalyzed by Well-Defined [Ni­(NHC)­(sty)_2_] Complexes by Nicasio and Belderrain

In 2014, the Tu group reported a NiCl_2_·DME/BIAN–IPr system for the BHA reaction of aryl tosylates (Scheme 183).^334^ Interestingly, this system utilizes PhBpin as a stoichiometric reductant (see Scheme 190).^335,336^ The bulky BIAN–IPr has outperformed less hindered IPr and IMes ligands under the reaction conditions (92% vs 51% and 13%). This catalytic system is distinguished by a broad substrate scope of 1° and 2° aliphatic amines and anilines in the cross-coupling with conjugated tosylates, such as naphthyl, anthracenyl, and phenanthrenyl. However, it appears that simple unactivated tosylates are unreactive.

**Scheme 183:**
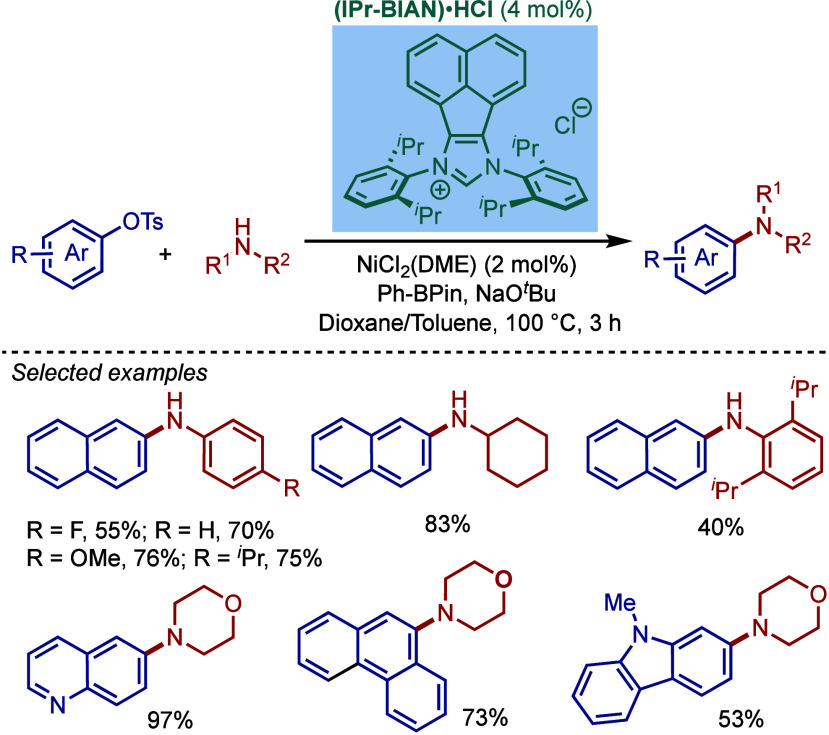
BHA Reaction of Aryl Tosylates Catalyzed by Ni–NHC Complexes by Tu

### 3.6 BHA Reaction of Aryl Silyl Ethers (C–OSiR_3_)

In 2018, Montgomery and co-workers reported the BHA reaction of silyloxyarenes using Ni­(cod)_2_/IPr^Me^ system (Scheme 184).^337^ The authors established that the backbone C3/C4-subsituted IPr^Me^ outperformed other ligands, such as IPr, IPr^Cl^, IMes, ICy, and IAd, while phosphines, such as dcype and dppf, were completely unreactive. Especially notable is the difference between IPr (58%) and IPr^Me^ (93%), suggesting that the steric substitution of the NHC ligand plays a key role in this reaction. The system proved effective for the amination of unactivated silyloxyarenes with 1° and 2° aliphatic amines and anilines using NaO^*t*^Bu in toluene at 120 °C. Furthermore, aryl phenyl ethers, pivalates, and triflates were also viable substrates for this transformation. The authors highlighted the efficiency of this approach by demonstrating a sequential coupling due to orthogonality between silyloxyarenes and other electrophiles, such as aryl methyl ethers and halides.

**Scheme 184:**
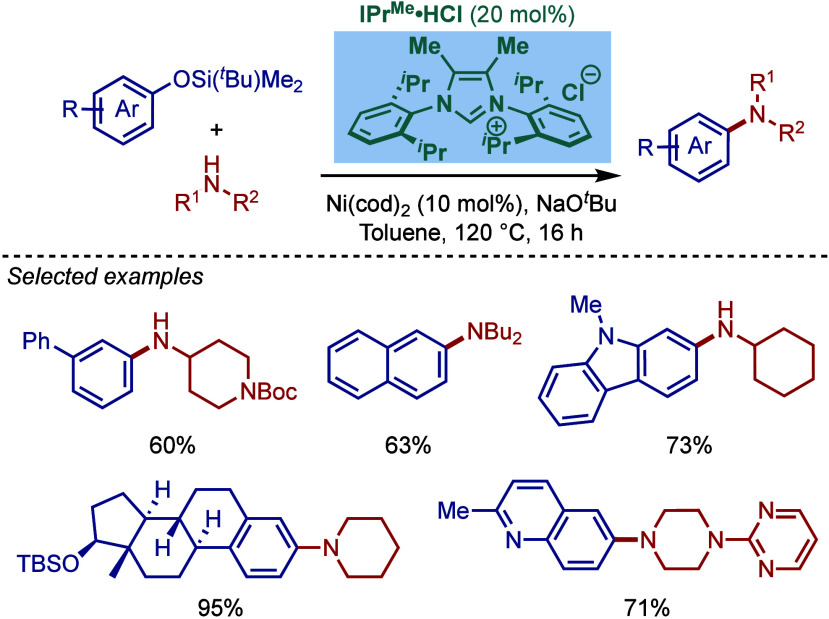
BHA Reaction of Silyloxyarenes Catalyzed by Ni–NHC Complexes by Montgomery

### 3.7 BHA Reaction of Aryl Methyl Ethers (C–OMe)

In 2009, Tobisu, Chatani, and co-workers reported the BHA reaction of anisoles catalyzed by a Ni­(cod)_2_/IPr system (Scheme 185).^338^ This challenging cross-coupling proceeded in the presence of NaO^*t*^Bu in toluene at 120 °C using 2-methoxynaphthalene as a model substrate. IPr was found to be the most effective ligand, outperforming other NHC ligands, such as IMes, IEt, and I*t*Bu, as well as phosphines, such as PCy_3_. However, the substrate scope was limited to activated anisole derivatives, while neutral anisole was completely unreactive.

**Scheme 185:**
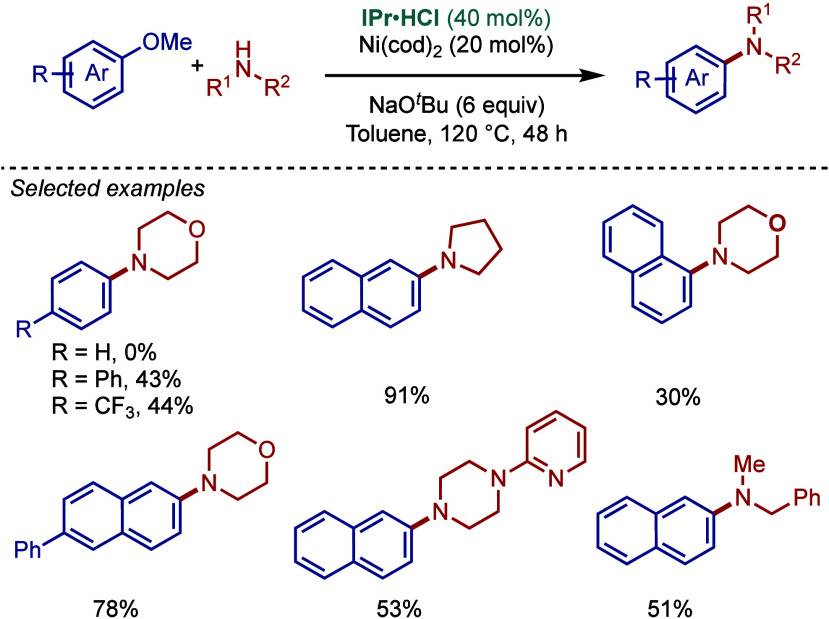
BHA Reaction of Aryl Methyl Ethers Catalyzed by Ni–NHC Complexes by Tobisu and Chatani

In 2012, the same group applied the same catalytic system to the BHA reaction of electron-deficient N-heteroarenes, such as pyridines, quinolines, isoquinolines, and quinoxalines (Scheme 186).^339^ They found that IPr outperformed its saturated counterpart SIPr in the model optimization (75% vs 54% yield). This catalytic system was compatible with cyclic 2° aliphatic amines, while acyclic amines and anilines reacted in low yields.

**Scheme 186:**
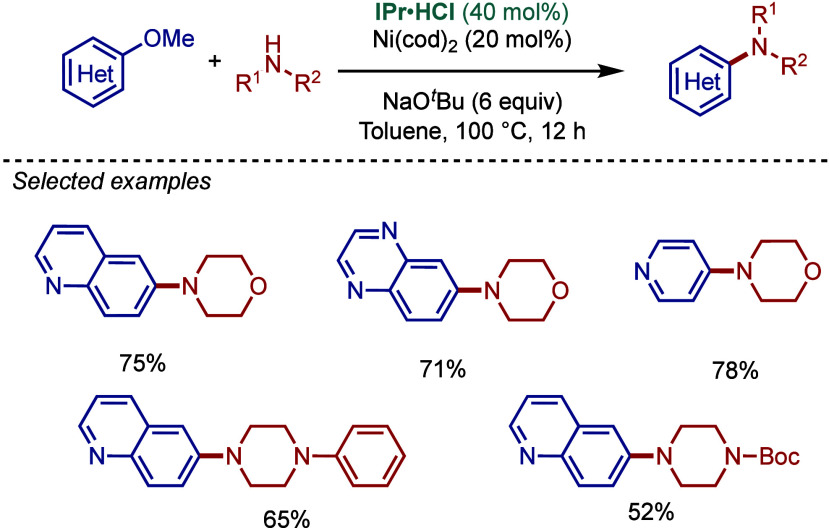
BHA Reaction of Aryl Heterocyclic Methyl Ethers Catalyzed by Ni–NHC Complexes by Tobisu and Chatani

### 3.8 BHA Reaction of Aryl Pivalates (C–OC­(O)­R)

In 2010, Tobisu, Chatani, and co-workers expanded their use of Ni­(cod)_2_/IPr system to facilitate BHA reaction of aryl carboxylates (Scheme 187).^340^ They found that the previously established conditions for the cleavage of aryl methyl esters using NaO^*t*^Bu in toluene at 120 °C (see Scheme 185)^214^ were highly effective for the cleavage of more activated aryl–O bonds. The most effective were pivalates and sulfamates (see Section 3.10), which permitted for lowering the reaction temperature to 80 °C, while phenyl esters resulted in low yields and methyl esters showed no conversion. Notably, IPr outperformed PCy_3_ as the most effective ligand (91% vs 51% yield). This methodology is characterized by a vastly expanded functional groups tolerance compared to the C–OMe amination due to the comparatively milder reaction conditions, including tolerance to amines, olefins, and heterocycles.

**Scheme 187:**
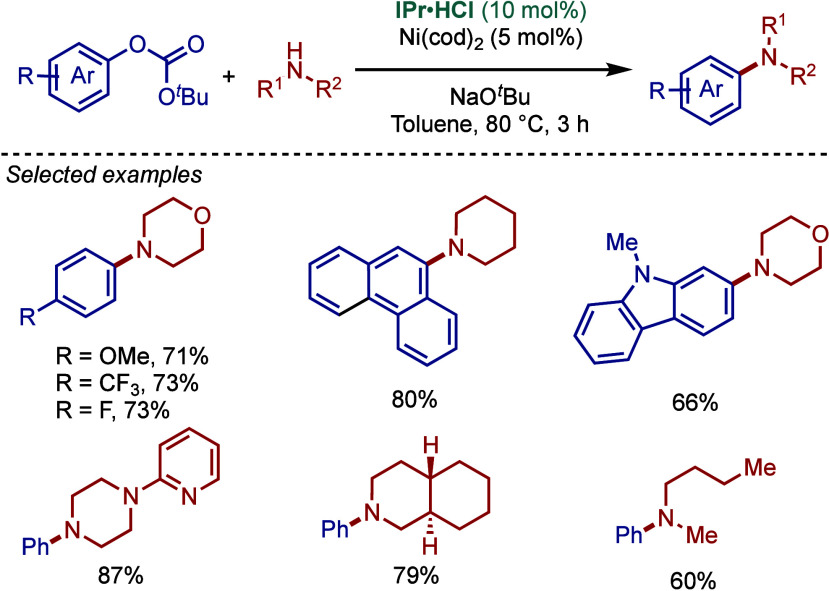
BHA Reaction of Aryl Pivalates by Catalyzed by Ni–NHC Complexes by Tobisu and Chatani

### 3.9 BHA Reaction of Aryl Carbamates (C–OC­(O)­NR_2_)

In 2011, Garg and co-workers demonstrated the BHA reaction of aryl carbamates using Ni–NHC catalysis (Scheme 188).^341^ They identified the Ni­(cod)_2_/SIPr system in the presence of NaO^*t*^Bu in dioxane at 80 °C as the most reactive, outperforming the more commonly used Ni/PCy_3_ system. DFT calculations showed that reductive elimination is the rate-determining step, with a barrier of 23.1 kcal/mol. The reaction showed a remarkably broad scope, including challenging electron-rich, heterocyclic, and sterically hindered carbamate substrates.

**Scheme 188:**
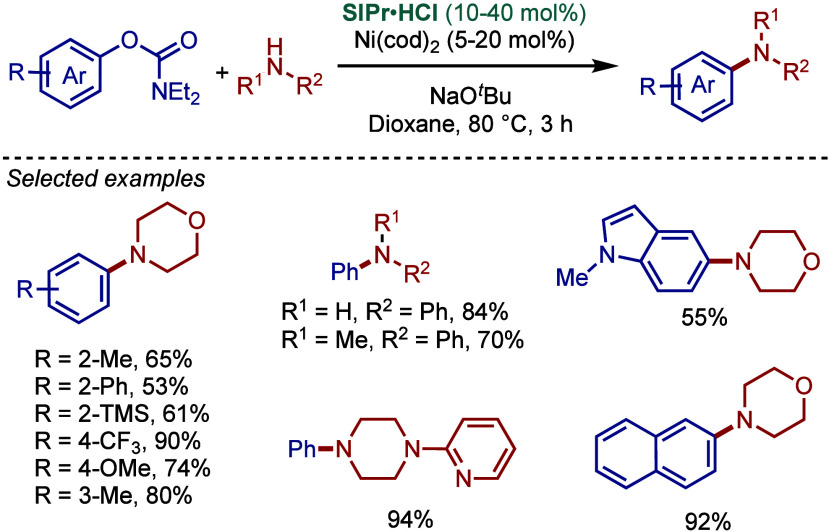
BHA Reaction of Aryl Carbamates Catalyzed by Ni–NHC Complexes by Garg

### 3.10 BHA Reaction of Aryl Sulfamates (C–OSO_2_NR_2_)

In 2010, the Garg group reported the BHA reaction of aryl sulfamates using a Ni­(cod)_2_/SIPr system (Scheme 189).^342^ In this cross-coupling, SIPr enabled the reaction more effectively than the more established phosphine-based Ni/PCy_3_ system. The method demonstrated broad substrate compatibility, encompassing electron-rich, heterocyclic and sterically hindered sulfamates in the cross-coupling with cyclic and acyclic 2° aliphatic amines and anilines.

**Scheme 189:**
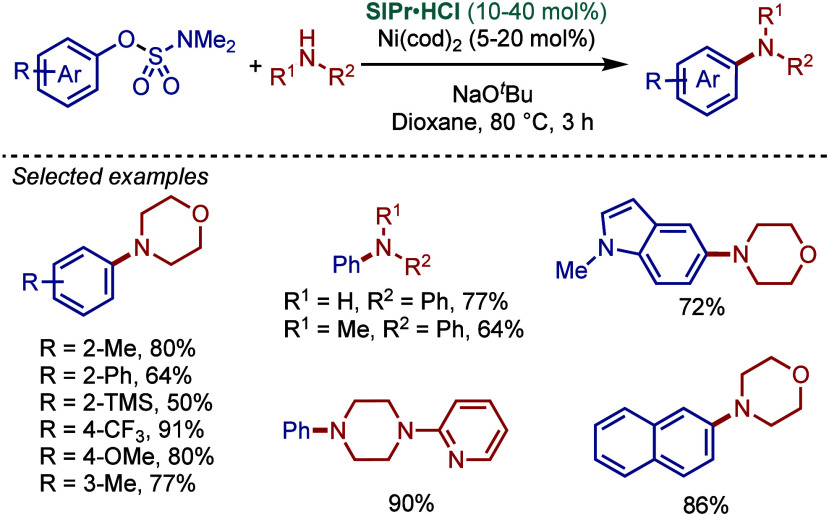
BHA Reaction of Aryl Sulfamates Catalyzed by Ni–NHC Complexes by Garg

In 2012, the Garg group reported an improved system for the BHA reaction of aryl carbamates and sulfamates using air-stable NiCl_2_·DME in the presence of PhBpin as a reductant as a practical alternative to the air-sensitive Ni­(cod)_2_ (not shown). Using this combination of reagents, aryl aryl carbamates and sulfamates could be readily cross-coupling in the presence of SIPr·HCl and NaO^*t*^Bu in dioxane at 80 °C.^335^ Subsequently, in 2014, the same group reported a similar system for BHA reaction of aryl carbamates and sulfamates using environmentally friendly 2-MeTHF as a solvent (Scheme 190).^336^ They demonstrated that this system can also be engaged to cross-coupling a variety of aryl electrophiles, such as chlorides, bromides, and tosylates, using NiCl_2_·DME/SIPr·HCl in the presence of NaO^*t*^Bu/PhBpin in 2-MeTHF at 80 °C, with sulfamates (87% yield), chlorides (95% yield), and carbamates (76% yield) showing the highest reactivity. Notably, this system could be applied to a gram-scale cross-coupling of heterocycles at 1 mol % Ni loading.

**Scheme 190:**
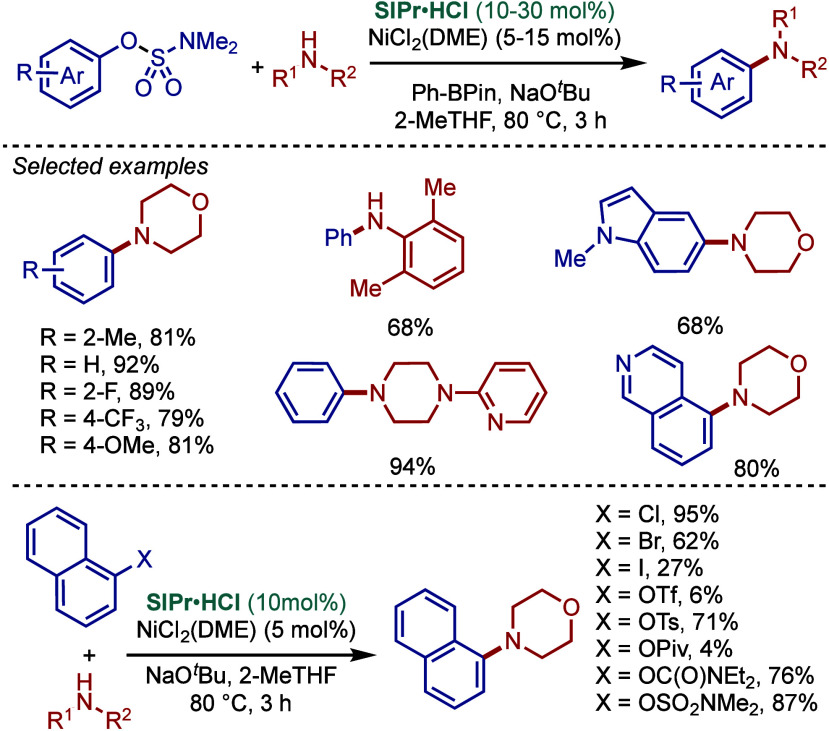
BHA Reaction of Aryl Sulfamates Catalyzed by Ni–NHC Complexes Using [NiCl_2_(DME)] in 2-MeTHF by Garg

### 3.11 BHA Reaction of Aryl Phosphates (C–OP­(O)­(OAr)_2_)

In 2011, the Yang group reported the first Ni-catalyzed BHA reaction of aryl phosphates using a Ni–NHC system (Scheme 191).^343^ They found that the previously established Ni­(II) precursor, [Ni­(PPh_3_)_2_(1-Np)­Cl],^304^ uniquely facilitated this reaction in the presence of IPr·HCl, while other Ni­(II) sources, such as NiCl_2_, Ni­(acac)_2_, and [Ni­(PPh_3_)_2_Cl_2_], were inactive. Interestingly, they found that NaH is the optimal base for this process, preventing phosphate hydrolysis and achieving the highest reactivity in the model amination (95% yield), while NaO^*t*^Bu was less effective (82% yield). This catalytic system demonstrated a broad substrate scope, efficiently coupling triaryl phosphates with 1° and 2° aliphatic amines and anilines. Electron-deficient and electron-rich phosphates were reactive under these conditions. Furthermore, diethyl aryl phosphates underwent selective cross-coupling, offering an atom-economic approach to the BHA reaction.

**Scheme 191:**
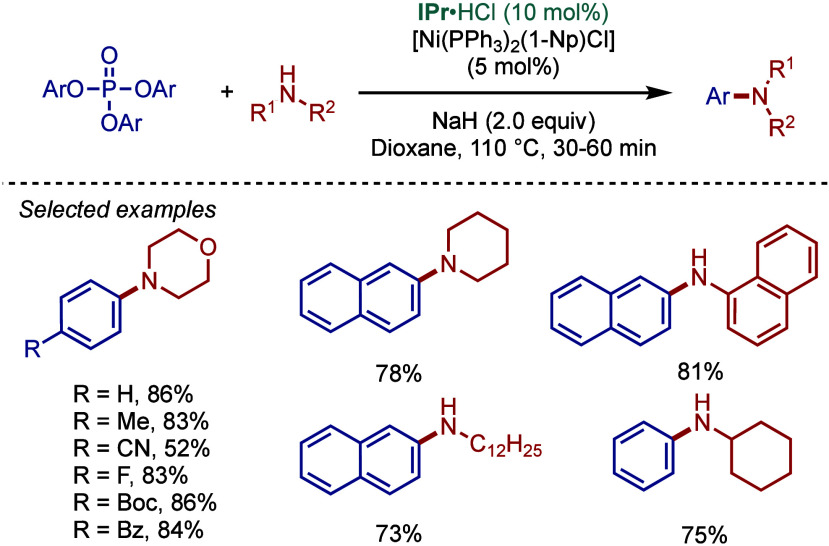
BHA Reaction of Aryl Phosphates Catalyzed by Ni–NHC Complexes by Yang

### 3.12 BHA Reaction of Aryl Fluorides (C–F)

In 2013, the Wang group pioneered Ni–NHC catalysis for BHA reaction of fluoroarenes with cyclic and acyclic amines (Scheme 192).^344^ The homogeneous system used involved Ni­(cod)_2_ and IPr·HCl in the presence of NaO^*t*^Bu in toluene at 100 °C. This challenging coupling required an excess of a base (4.2 equiv) and higher temperatures compared to a typical halide partner. The key catalytic species was identified as in situ-formed [Ni­(IPr)_2_]. Ligand screening revealed that phosphines, such as dppp and PCy_3_, were ineffective, while other NHCs ligands showed lower activity (IMes, 69%; I*t*Bu: 5% vs IPr: 92%). The functional group tolerance of this amination is surprisingly high, including ketones, olefins, amides, and heterocycles. Furthermore, various cyclic and acyclic 2° aliphatic amines and anilines are well tolerated.

**Scheme 192:**
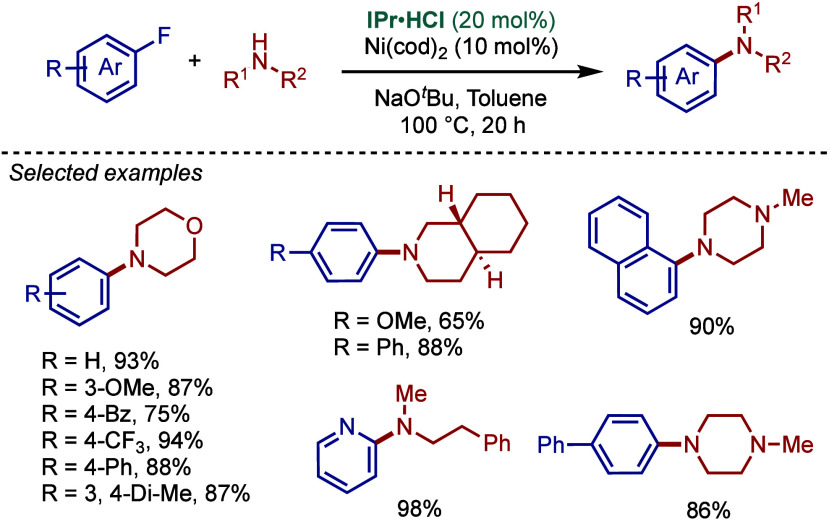
BHA Reaction of Aryl Fluorides Catalyzed by Ni–NHC Complexes by Wang

### 3.13 Decarbonylative BHA Reaction of Amides (C–C­(O)­NR_2_)

In 2017, Rueping and co-workers reported the first decarbonylative BHA reaction deploying electronically activated amides as electrophilic substrates (Scheme 193).^345^ Notably, the authors identified 2-azinecarboxamides activated by the anilide group as viable substrates using NiCl_2_/IPr·HCl in the presence of K_3_PO_4_ in toluene at 170 °C. The reaction required extremely high temperatures to facilitate decarbonylation. Ni­(cod)_2_ and different NiX_2_ salts (X = Cl, Br, I) showed similar reactivity. Different NHC ligands, such as IPr, IMes, SIPr, ICy, and I*i*Pr (42–73% yields), outperformed phosphine ligands, such as PCy_3_ and P*n*Bu_3_ (<25% yield). The mechanism for this intramolecular Buchwald–Hartwig fragment coupling of amides relies on oxidative addition of the N–C­(O) bond, CO extrusion, and reductive elimination, enabling direct interconversion of amides to amidines.

**Scheme 193:**
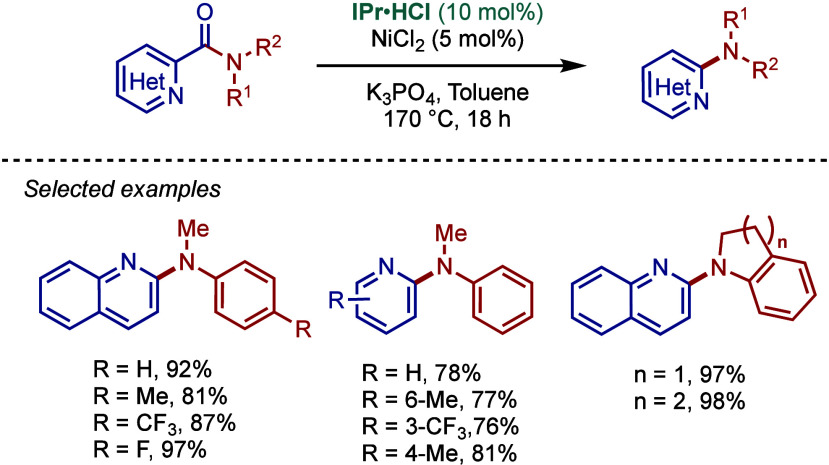
Decarbonylative BHA Reaction of Amides Catalyzed by Ni–NHC Complexes by Rueping

### 3.14 Desulfitative BHA Reaction of Sulfonamides (C–SO_2_NR_2_)

In 2020, Lian and co-workers reported a desulfitative BHA reaction of heterocyclic sulfonamides using Ni–NHC catalysis (Scheme 194).^346^ In this reaction, 2-azinesulfonamides serve as viable substrates to afford amidines at a comparatively lower temperature of 60 °C. Different N-sulfonamide groups are compatible, including cyclic and acyclic 2° amines and anilines. Interestingly, compared to the previously reported Ni­(cod)_2_/IPr and NiCl_2_/IPr systems for decarbonylation (see Scheme 193),^221^ the present system permitted to significantly lower the temperature required for SO_2_ extrusion. The most effective is Ni­(cod)_2_/IPr·HCl in the presence of a BPh_3_ additive and NaO^*t*^Bu in xylene. The authors proposed that the Lewis acid coordinates to the sulfonyl group, activating the substrate toward oxidative addition and desulfination. Among the ligands tested, IPr proved to be the most effective, outperforming IMes and SIPr, while bipyridine ligands and phosphines were ineffective in this transformation.

**Scheme 194:**
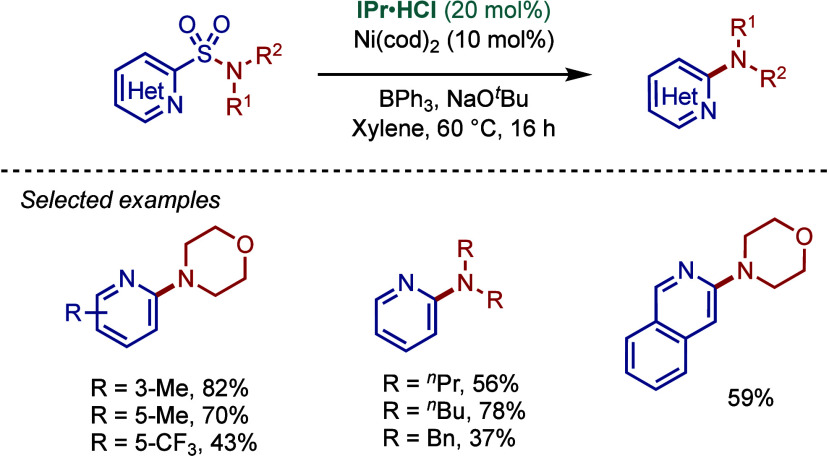
Desulfitative BHA Reaction of Sulfonamides Catalyzed by Ni–NHC Complexes by Lian

### 3.15 Acyl BHA Reaction of Esters (C­(O)–OR)

In 2016, the Garg group reported acyl BHA reaction of methyl esters using Ni–NHC catalysis (Scheme 195).^347^ They found that Ni­(cod)_2_/SIPr in the presence of stoichiometric Al­(O*t*Bu)_3_ in toluene at 60 °C promotes acyl C–O activation/C–N cross-coupling. The authors proposed that Al­(OtBu)_3_ played a dual role by (1) promoting the amidation process from endergonic (ΔG = 4.9 kcal/mol) to nearly thermoneutral (ΔG = 0.2 kcal/mol) by coordinating to the amide carbonyl oxygen atom, and (2) lowering the kinetic barrier for the rate-determining oxidative addition step. Notably, the reaction did not proceed without Ni­(cod)_2_/SIPr, while IPr was similarly effective to SIPr. In contrast, other ligands, such as mono- and bidentate phosphines, bipyridines, and bisoxazolines, resulted in either no reaction or low conversion. DFT studies revealed that methyl 1-naphthoate was more reactive than methyl phenolate due to distortion of the ester–Al­(OR)_3_ complex. Scope studies identified 2° N-alkyl-N-aryl amines as the most suitable nucleophiles and decarbonylation did not occur under standard conditions.

**Scheme 195:**
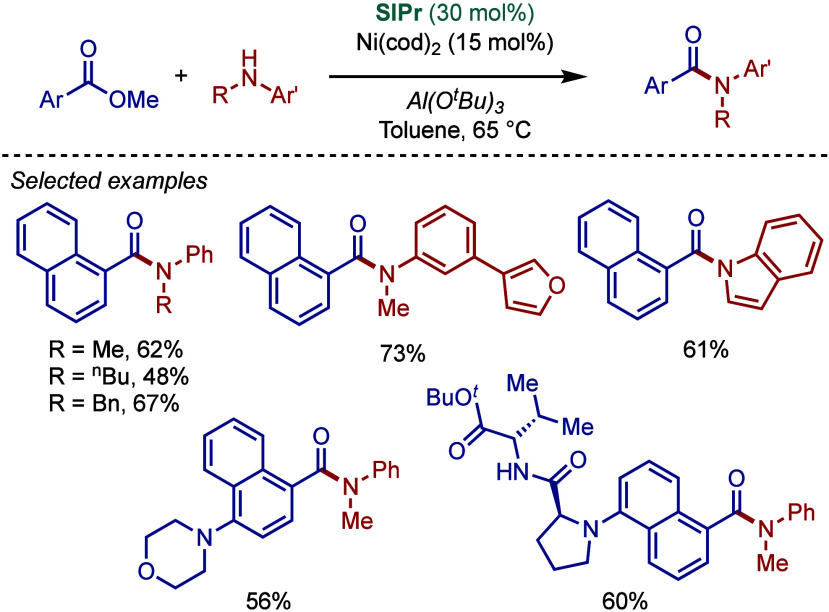
Acyl BHA Reaction of Methyl Esters Catalyzed by Ni–NHC Complexes by Garg

In 2018, Newman and co-workers reported a related Ni­(cod)_2_/IPr system for acyl Buchwald–Hartwig cross-coupling of methyl esters (Scheme 196).^348^ Compared with the previous system (see Scheme 195),^223^ this reaction is distinguished by an acid/base-free conditions and using elevated temperatures in toluene at 140 °C to drive the equilibrium forward by removal of the methanol byproduct. The mechanism has been further studied and validated by DFT studies by Hong and co-workers.^349^ This catalytic system is considerably robust, demonstrating a broad substrate scope and functional group compatibility, including aliphatic and aromatic esters, various 1° and 2° aliphatic and aromatic amines and heterocycles.

**Scheme 196:**
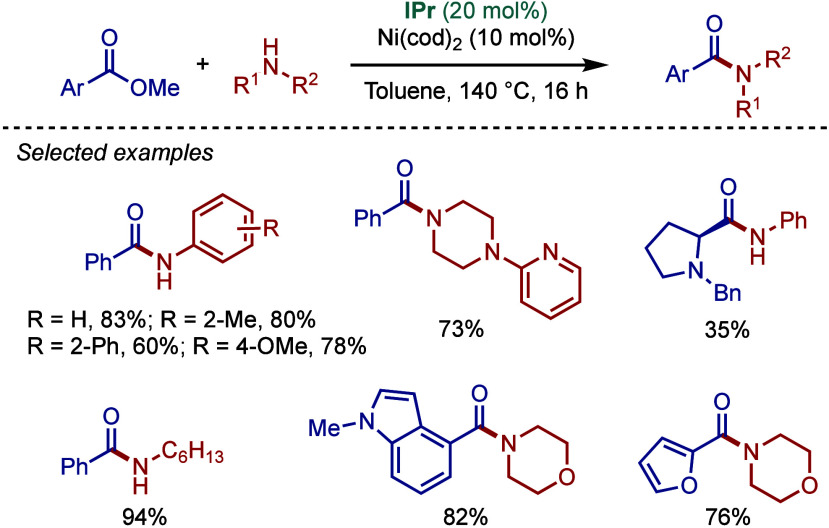
Acyl BHA Reaction of Methyl Esters Catalyzed by Ni–NHC Complexes by Newman

Subsequently, in 2019, the Newman group evaluated a broad range of ligands as a general strategy for acyl BHA reaction of methyl esters (Scheme 197).^350^ They found that phosphine ligands (XanPhos, DalPhos, SPhos, P­(*o*-tol)_3_, PCy_3_, dcypf) and select NHC ligands (BIAN–IPr, IPr^Me^, ICy, IMes, SIMes) were barely reactive using their optimized conditions. The authors identified several privileged N-heterocyclic carbene scaffolds for this amination, including IPr, IPr*, IMes^CPent^, and IPr/2-Py, showing excellent efficiency (83–90% yields). Interestingly, bidentate bipyridine and phosphine ligands, such as Me_4_-1,10-Phen and dcype, dcypp also showed high activity (73–83% yields). Using this tailored library of ligands, the scope of this amination was expanded to challenging sterically hindered esters and amines.

**Scheme 197:**
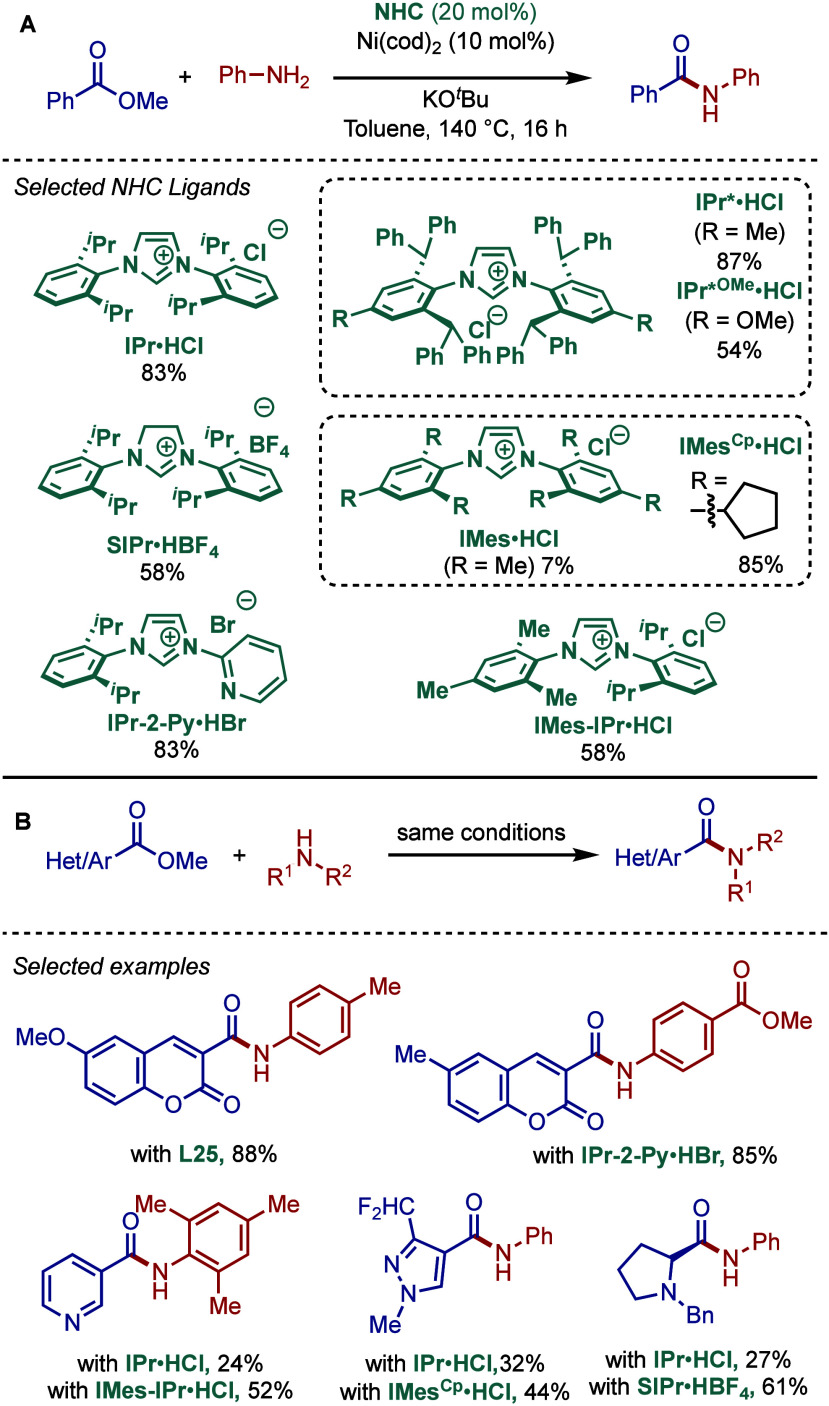
Acyl BHA Reaction of Methyl Esters Catalyzed by Ni–NHC Complexes with Tailored Ligands by Newman

### 3.16 Acyl BHA Reaction of Amides (C­(O)–NR_2_)

In 2016, Garg and co-workers reported acyl BHA reaction of amides using Ni–NHC catalysis (Scheme 198).^351^ This approach utilizes a similar Ni­(cod)_2_/SIPr system in toluene at 35–60 °C. Different aromatic N-Me-N-Boc and N-Bn-N-Boc amides could be engaged in the cross-coupling with aliphatic, aromatic, and heterocyclic amines. Notably, this approach represents a formal two-step transamidation of common 2° amides. The highlight of this method was BHA reaction using amino acid derivatives, showcasing robustness, and potential application in the synthesis of bioactive molecules.

**Scheme 198:**
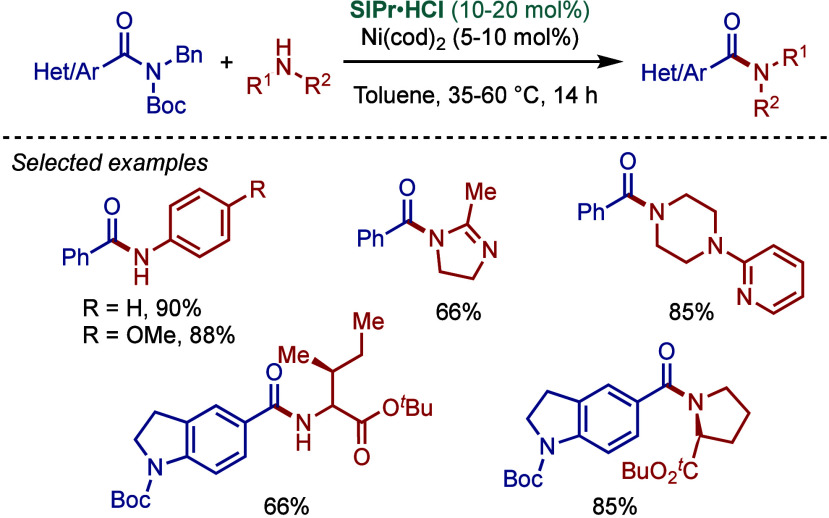
Acyl BHA Reaction of Amides Catalyzed by Ni–NHC Complexes by Garg

In 2017, the Garg group advanced their Ni–NHC catalysis to achieve acyl BHA reaction of aliphatic amides (Scheme 199).^352^ For this more challenging transamidation, they identified a more electron-rich benzimidazol-2-ylidene BenzICy ligand that proved highly effective in promoting a more efficient oxidative addition step. Interestingly, attempts using the previous imidazolin-2-ylidene SIPr system and tridentate terpyridine ligands failed to promote this reaction. Furthermore, the BenzICy ligand was used in its salt form with a catalytic amount of NaO^*t*^Bu for in situ deprotonation. This approach enables successful BHA reaction of a variety of 2° aliphatic amides with 1° and 2° aliphatic and aromatic amines.

**Scheme 199:**
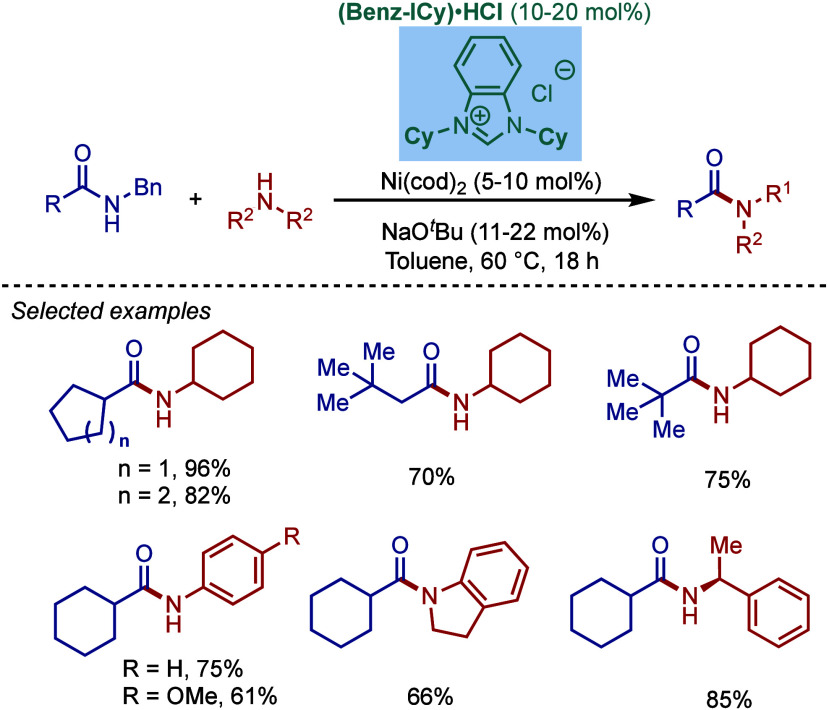
Acyl BHA Reaction of Aliphatic Amides Catalyzed by Ni–NHC Complexes by Garg

In 2020, Szostak and co-workers group reported the acyl BHA reaction of aromatic N-Boc amides using a cyclopentadienyl [Ni­(IPr)­(Cp)­Cl] complex (Scheme 200).^353^ This system features a well-defined, air- and moisture-stable Ni­(II)–NHC precatalyst in the presence of the mild base K_2_CO_3_ in toluene at 140 °C. Notably, this catalyst is also effective in BHA reaction of aliphatic amides and BHA reactions involving phenyl and methyl esters.

**Scheme 200:**
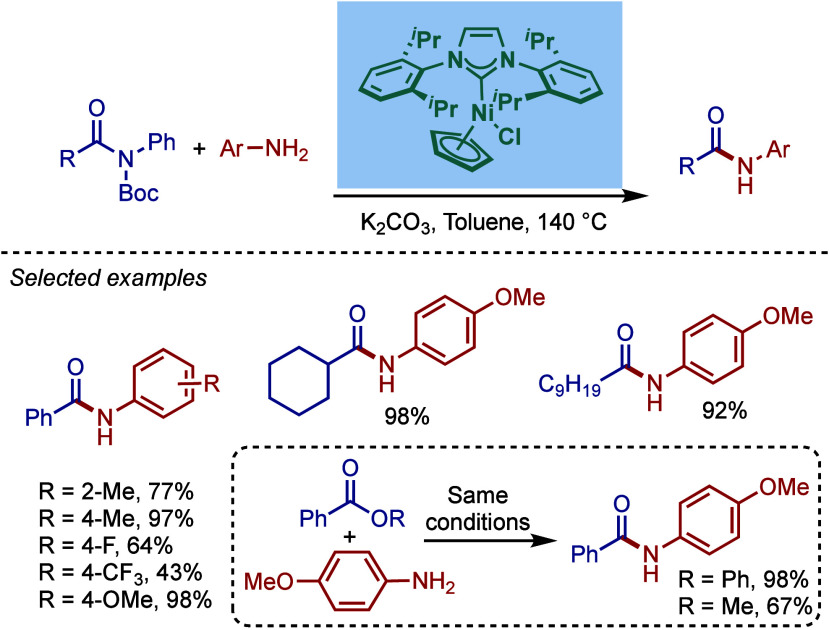
Acyl BHA Reaction of Amides Catalyzed by Well-Defined [Ni­(IPr)­(Cp)­Cl] by Szostak

## 4 Cobalt–NHC Complexes

In 2015, Bala and co-workers reported the synthesis of pincer CNC cobalt­(II)–NHC complexes and their application in BHA reaction of aryl halides (Scheme 201).^354^ These well-defined and air-stable [Co­(II)–NHC] complexes were synthesized as hexafluorophosphate salts from [CoCl_2_(PPh_3_)_2_] via Ag–NHC transfer. The complexes demonstrated high activity in BHA reaction of aryl bromides with 1° anilines using KO^*t*^Bu in THF at reflux. However, it is important to note that the substrate scope was limited to aryl bromides, with only one reported example of an activated aryl chloride, 4-nitrochlorobenzene. The authors proposed a Co(0)/Co­(II) mechanism with the pincer ligand providing a hemilabile stabilization during the catalytic cycle.

**Scheme 201:**
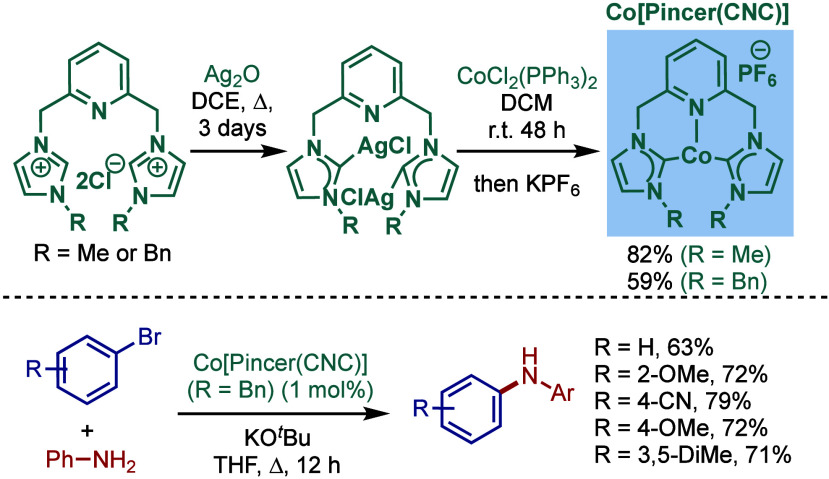
BHA Reaction Catalyzed by Pincer [Co­(CNC)­(NHC)] Complexes by Bala

In 2016, the Bala group reported a series of [Co­(NHC)_2_Cl_2_] complexes featuring chelating 2-picolyl N-wingtips in an unsymmetrical NHC scaffold (R = 4-NO_2_-C_6_H_4_, allyl, butenyl) (Scheme 202).^325^ These air-sensitive complexes were synthesized via carbene transfer from [Co­(PPh_3_)_2_Cl_2_] and the corresponding Ag­(I)–NHC precursors. Catalytic studies in the model BHA reaction of bromobenzene with aniline using KO^*t*^Bu in THF at reflux revealed that the complex with a N-allyl wingtip substituent was the most reactive (allyl: 40%; butenyl: 20%; 4-NO_2_-C_6_H_4_: 15%). However, the catalytic efficiency was lower compared to their nickel analogues under the same conditions (see Scheme 173).

**Scheme 202:**
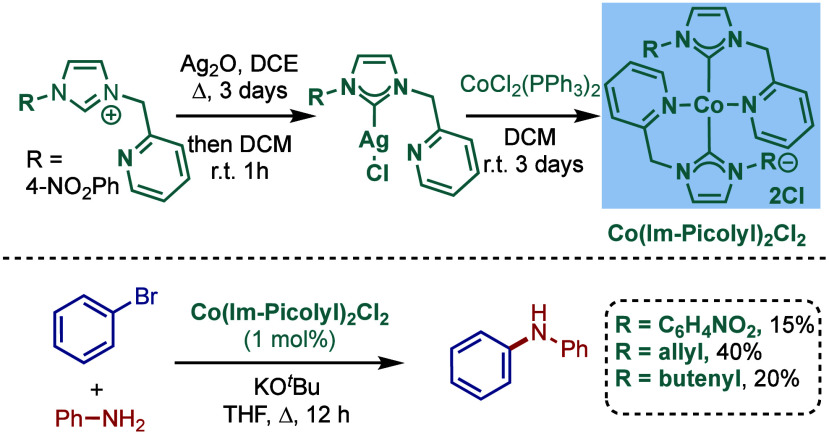
BHA Reaction Catalyzed by [Co­(NHC)_2_Cl_2_] Complexes by Bala

## 5 Rhodium–NHC Complexes

In 2010, Chang and co-workers reported the first example of BHA reaction of aryl halides catalyzed by Rh­(I)–NHC complexes (Scheme 203).^355^ The authors identified N-alkyl imidazol-2-ylidene I*i*Pr as the most effective ligand, outperforming IMes and PCy_3_. The reaction was carried out with an in situ-formed [Rh­(I*i*Pr)­(cod)­Cl] complex using [Rh­(cod)_2_]­[BF_4_] and I*i*Pr·HCl in the presence of NaO^*t*^Bu in DME at 80 °C. The authors found that although the well-defined, air- and moisture-stable [Rh­(I*i*Pr)­(cod)­Cl] was catalytically active, combining it with AgBF_4_ significantly improved its performance. This suggested the involvement of a cationic [Rh­(I*i*Pr)­(cod)]^+^ as a potential catalytically active species. The amine scope and functional group tolerance of this BHA reaction are quite broad encompassing 1° and 2° aliphatic heterocyclic and aromatic amines as well as various sensitive functional groups, such as esters, ketones, Bpin, and TMS. In contrast, the scope of aryl halides is limited to aryl bromides, with one example of an activated aryl chloride (4-Ac-C_6_H_4_-Cl) reported at elevated temperature (120 °C).

**Scheme 203:**
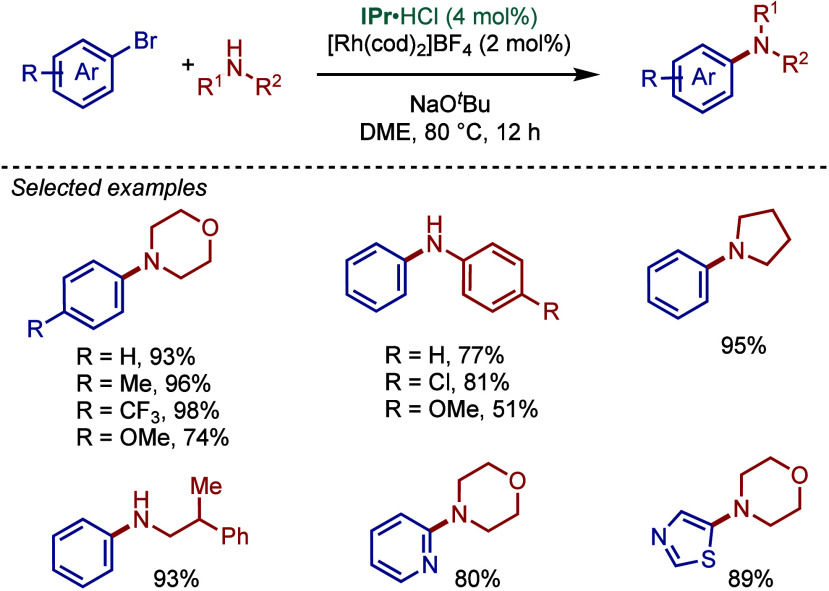
BHA Reaction Catalyzed by Rh­(I)–NHC Complexes by Chang

## 6 Summary and Outlook

Tremendous advances have been made in the use of N-heterocyclic carbenes as ancillary ligands for BHA reactions. Although the initial progress was focused solely on phosphines, there are now numerous examples where the use of N-heterocyclic carbenes surpasses the reactivity of phosphine-base systems and enables challenging BHA reactions that are beyond the scope of phosphine ligands. In this respect, during the last 25 years, N-heterocyclic carbenes have played an instrumental role in the development of new BHA reactions. Among the large variety of Pd–NHC and Ni–NHC complexes, several classes of catalysts have emerged as privileged, including [Pd­(NHC)­(allyl)­Cl], [Pd­(NHC)­(acac)­Cl], and [Pd­(NHC)­(Het)­Cl_2_] complexes as stand-out choices for challenging BHA reactions.

The progress in the area has been significant. The major advances made in the past two decades are as follows: (1) this field has now reached beyond academic research, and the use of Pd–NHC complexes is common in industrial research for C–N bond coupling reactions. A major factor that contributes to the advancement of Pd–NHCs to the industrial arena is the stability of NHC-based catalysts. For example, the sterically demanding precatalyst, [Pd­(IPr*)­(cin)­Cl], was used in large scale pharmaceutical process and could even be employed in a flow microreactor setting, reaching full conversion within 20 min at very low catalyst loading. (2) Catalytic efficiency of Pd–NHCs can reach remarkably high turnover, surpassing the activity of other catalytic systems. For example, it is common that Pd–NHC-catalyzed BHA reactions can be performed at 0.001 mol % (10 ppm) catalyst loading and a TON of 100,000 as illustrated by the [Pd­(SIPr)­(cin)­Cl] catalyst. (3) The Pd–NHC-catalyzed BHA reaction has had a major impact on the synthesis of bioactive molecules, such as Piribedil, Sonidegib, and Brexpiprazole, as a key step, demonstrating real-life impact of this catalyst platform. (4) The high robustness of Pd–NHCs means that these catalysts can be readily immobilized with Wang resin or polyvinylpyridine. These immobilized catalysts can be easily removed from the reaction mixture by simple filtration, leaving the opportunity to reuse the catalyst, while Pd leaching into the reaction media is suppressed to below 1 ppm. (5) In terms of substrate scope, Pd–NHCs are particularly well-suited for the synthesis of highly sterically hindered amines in excellent yields. (6) Another area of application are BHA reactions catalyzed by heavily alkylated NHC–Pd complexes in nonpolar alkane solvents. Nonpolar alkanes, such as heptane and cyclohexane, are considered among the most favorable solvents for industrial applications because of their high calorific power, which offers the energetic balance. (7) In terms of ligands, cinnamyl-based Pd–NHC complexes have been shown to be the most reactive; however, incorporation of N-based ligands to stabilize palladium has offered another excellent alternative to tertiary phosphine ligands and resulted in many applications of Pd–NHC catalysts in BHA reactions. This diversity of both NHC and ancillary ligands allows for further fine-tuning of efficient palladium-catalyzed BHA reactions for tailored applications. (8) Although this research field was initially focused on Pd–NHC complexes, recent developments have advanced this catalysis platform to more sustainable Ni–NHCs. Following the research in Pd–NHCs, the catalyst development has evolved from in situ-generated Ni(0)–NHC systems to well-defined Ni(0)–NHC, Ni­(II)–NHC, and Ni­(I)–NHC complexes, which has significantly improved catalytic efficiency, reactivity, and air- and moisture-stability of the complexes, resulting in operational simplicity and ease of application. (9) Importantly, Ni–NHC catalysis has enabled one to expand the substrate scope of BHA reactions to some of the most challenging electrophiles in organic synthesis, including unactivated aryl sulfamates, carbamates, and pivalates, as well as novel electrophiles, such as aryl fluorides, methyl ethers, and silyloxyarenes. (10) Furthermore, it should be clearly stated that the use of Ni–NHC has made major advances in asymmetric BHA reactions using chiral NHC ligands, which has enabled enantioselective C–N bond formations for sterically hindered amine substrates.

However, despite a very significant progress, this rapidly evolving field has also numerous challenges that should be addressed. The key challenges for the future research are as follows: (1) Considering the rising cost of palladium, N-heterocyclic carbene complexes of Earth-abundant 3d-transition metals should be evaluated for BHA reactions. (2) In the most developed Ni–NHC systems, the highly reactive, air- and moisture-stable well-defined Ni–NHC complexes remain significantly underdeveloped compared with state-of-the-art Pd–NHCs catalysts, thus limiting their application in practical and operationally simple amination reactions. (3) In Ni–NHC catalysis, relatively high catalyst loading (around 5 mol %), higher temperatures (80 °C), and strong bases are still needed for most applications, which restricts the compatibility of these systems with sensitive groups in advanced late-stage functionalization. (4) There is a significant area for improvement using green and environmentally friendly methods for BHA reactions that avoid using organic solvents. (5) Effective use of computations should be more common to provide a comprehensive mechanistic approach to elucidate reaction mechanisms and aid in reaction optimization. (6) Highly active N-heterocyclic carbene catalysts should be designed for the development of mild reaction conditions at low catalyst loading that could tolerate complex coordinating functional groups. (7) It is critical that new N-heterocyclic carbene systems for BHA reactions using weak bases are developed. The use of strong bases restricts the functional group compatibility, while a common use of mild bases would have a practical and wide-reaching implications for this reaction. The use of mild bases is also conducive to maintaining efficiency at scale and enabling a range of broader implementations in industrial settings. (8) Finally, considering the NHC–Pd(0)/Pd­(II) and NHC–Ni(0)/Ni­(II) catalytic cycles, the development of air-stable and robust NHC–Pd(0) and NHC–Ni(0) catalysts by ancillary ligand tuning could considerably increase the practical efficiency of the Buchwald–Hartwig cross-coupling platform.

Altogether, the use of N-heterocyclic carbene complexes has significantly broadened the realm of BHA reactions. It is evident that N-heterocyclic carbenes should be routinely included in the standard toolbox of ligands for the development and optimization of BHA reactions.

## References

[1] Hartwig J. F. (1998). Transition
Metal Catalyzed Synthesis of Arylamines and Aryl Ethers from Aryl
Halides and Triflates: Scope and Mechanism. Angew. Chem., Int. Ed..

[2] Yang B. H., Buchwald S. L. (1999). Palladium-Catalyzed
Amination of Aryl Halides and Sulfonates. J.
Organomet. Chem..

[3] Lundgren R. J., Stradiotto M. (2012). Addressing Challenges in Palladium-Catalyzed
Cross-Coupling
Reactions Through Ligand Design. Chem. Eur.
J..

[4] Wolfe J. P., Wagaw S., Marcoux J.-F., Buchwald S. L. (1998). Rational Development
of Practical Catalysts for Aromatic Carbon–Nitrogen Bond Formation. Acc. Chem. Res..

[5] Palladium-Catalyzed Amination of Aryl Halides and Related Reactions: Hartwig, J. F. Handbook of Organopalladium Chemistry for Organic Synthesis; Wiley-Interscience: New York, 2003.

[6] Schlummer B., Scholz U. (2004). Palladium-Catalyzed
C–N and C–O Coupling–A
Practical Guide from an Industrial Vantage Point. Adv. Synth. Catal..

[7] Buchwald S. L., Mauger C., Mignani G., Scholz U. (2006). Industrial-Scale Palladium-Catalyzed
Coupling of Aryl Halides and Amines – A Personal Account. Adv. Synth. Catal..

[8] Corbet J.-P., Mignani G. (2006). Selected Patented Cross-Coupling Reaction Technologies. Chem. Rev..

[9] Torborg C., Beller M. (2009). Recent Applications of Palladium-Catalyzed Coupling
Reactions in the Pharmaceutical, Agrochemical, and Fine Chemical Industries. Adv. Synth. Catal..

[10] Magano J., Dunetz J. R. (2011). Large-Scale Applications of Transition Metal-Catalyzed
Couplings for the Synthesis of Pharmaceuticals. Chem. Rev..

[11] Vitaku E., Smith D. T., Njardarson J. T. (2014). Analysis
of the Structural Diversity,
Substitution Patterns, and Frequency of Nitrogen Heterocycles among
U.S. FDA Approved Pharmaceuticals. J. Med. Chem..

[12] Ruiz-Castillo P., Buchwald S. L. (2016). Applications of
Palladium-Catalyzed C–N Cross-Coupling
Reactions. Chem. Rev..

[13] Dorel R., Grugel C. P., Haydl A. M. (2019). The Buchwald–Hartwig
Amination
After 25 Years. Angew. Chem., Int. Ed..

[14] Emadi R., Nekoo A. B., Molaverdi F., Khorsandi Z., Sheibani R., Sadeghi-Aliabadi H. (2023). Applications
of Palladium-Catalyzed
C–N Cross-coupling Reactions in Pharmaceutical Compounds. RSC Adv..

[15] Marshall C. M., Federice J. G., Bell C. N., Cox P. B., Njardarson J. T. (2024). An Update
on the Nitrogen Heterocycle Compositions and Properties of U.S. FDA-Approved
Pharmaceuticals (2013–2023). J. Med.
Chem..

[16] Goldberg I. (1906). Ueber Phenylirungen
bei Gegenwart von Kupfer als Katalysator. Chem.
Ber..

[17] Ullmann F. (1903). Ueber eine
neue Bildungsweise von Diphenylaminderivaten. Chem. Ber..

[18] Sambiagio C., Marsden S. P., Blacker J. A., McGowan P. C. (2014). Copper Catalysed
Ullmann Type Chemistry: From Mechanistic Aspects to Modern Development. Chem. Soc. Rev..

[19] Yang Q., Zhao Y., Ma D. (2022). Cu-Mediated Ullmann-Type Cross-Coupling
and Industrial Applications in Route Design, Process Development,
and Scale-up of Pharmaceutical and Agrochemical Processes. Org. Process Res. Dev..

[20] Kosugi M., Kameyama M., Migita T. (1983). Palladium-catalyzed
Aromatic Amination
of Aryl Bromides with n,n-Di-Ethylaminotributyltin. Chem. Lett..

[21] Paul F., Patt J., Hartwig J. F. (1994). Palladium-Catalyzed Formation of
Carbon-Nitrogen Bonds. Reaction Intermediates and Catalyst Improvements
in the Hetero Cross-Coupling of Aryl Halides and Tin Amides. J. Am. Chem. Soc..

[22] Guram A. S., Buchwald S. L. (1994). Palladium-Catalyzed Aromatic Aminations with in situ
Generated Aminostannanes. J. Am. Chem. Soc..

[23] Guram A. S., Rennels R. A., Buchwald S. L. (1995). A Simple
Catalytic Method for the
Conversion of Aryl Bromides to Arylamines. Angew.
Chem., Int. Ed. Engl..

[24] Louie J., Hartwig J. F. (1995). Palladium-Catalyzed Synthesis of Arylamines from Aryl
Halides. Mechanistic Studies Lead to Coupling in the Absence of Tin
Reagents. Tetrahedron Lett..

[25] Wolfe J. P., Buchwald S. L. (1997). Nickel-Catalyzed
Amination of Aryl Chlorides. J. Am. Chem. Soc..

[26] Hartwig J. F. (2008). Carbon-heteroatom
Bond Formation Catalysed by Organometallic Complexes. Nature.

[27] Surry D. S., Buchwald S. L. (2011). Dialkylbiaryl Phosphines in Pd-catalyzed Amination:
a User’s Guide. Chem. Sci..

[28] Bariwal J., Van der Eycken E. (2013). C–N
Bond Forming Cross-Coupling Reactions: an
Overview. Chem. Soc. Rev..

[29] Heravi M. M., Kheilkordi Z., Zadsirjan V., Heydari M., Malmir M. (2018). Buchwald–Hartwig
reaction: An overview. J. Organomet. Chem..

[30] Surry D. S., Buchwald S. L. (2008). Biaryl Phosphane
Ligands in Palladium-Catalyzed Amination. Angew.
Chem., Int. Ed..

[31] Hartwig J. F. (2008). Evolution
of a Fourth Generation Catalyst for the Amination and Thioetherification
of Aryl Halides. Acc. Chem. Res..

[32] Ingoglia B. T., Wagen C. C., Buchwald S. L. (2019). Biaryl
Monophosphine Ligands in Palladium-Catalyzed
C–N coupling: An Updated User’s guide. Tetrahedron.

[33] Dhanjee H. H., Buslov I., Windsor I. W., Raines R. T., Pentelute B. L., Buchwald S. L. (2020). Palladium–Protein Oxidative
Addition Complexes
by Amine-Selective Acylation. J. Am. Chem. Soc..

[34] Buchwald S. L., Hartwig J. F. (2020). In Praise of Basic
Research as a Vehicle to Practical
Applications: Palladium-Catalyzed Coupling to Form Carbon-Nitrogen
Bonds. Isr. J. Chem..

[35] Hartwig J. F., Shaughnessy K. H., Shekhar S., Green R. A. (2019). Palladium-Catalyzed
Amination of Aryl Halides. Organic Reactions.

[36] N-Heterocyclic Carbenes: Effective Tools for Organometallic Synthesis; Nolan, S. P. , Ed.; Wiley: Weinheim, 2014; pp 1–568.

[37] N-Heterocyclic Carbenes in Transition Metal Catalysis; Cazin, C. S. J. , Ed.; Springer: New York, 2011; pp 1–336.

[38] Jimenez-Nunez, E. ; Alcarazo, M. C–N Bond Formation. In N-Heterocyclic Carbenes in Transition Metal Catalysis and Organocatalysis; Cazin, C. S. J. , Ed.; Springer: Heidelberg, 2011.

[39] Chartoire, A. ; Nolan, S. P. Amination Reactions. In New Trends in Cross-Coupling; Colacot, T. J. , Ed.; Royal Society of Chemistry: Cambridge, 2015.

[40] N-Heterocyclic Carbenes: From Laboratory Curiosities to Efficient Synthetic Tools; Diez-Gonzalez, S. J. , Ed.; RSC: Cambridge, 2016; pp 1–442.

[41] Fortman G. C., Nolan S. P. (2011). Buchwald–Hartwig Amination In N-Heterocyclic
Carbene (NHC) Ligands and Palladium in Homogeneous Cross-Coupling
Catalysis: a Perfect Union. Chem. Soc. Rev..

[42] Kantchev E. A. B., O’Brien C. J., Organ M. G. (2007). Palladium Complexes
of N-Heterocyclic Carbenes as Catalysts for Cross-Coupling Reactions—A
Synthetic Chemist-s Perspective. Angew. Chem.,
Int. Ed..

[43] Froese R. D. J., Lombardi C., Pompeo M., Rucker R. P., Organ M. G. (2017). Designing
Pd–N-Heterocyclic Carbene Complexes for High Reactivity and
Selectivity for Cross-Coupling Applications. Acc. Chem. Res..

[44] Arduengo A. J., Harlow R. L., Kline M. (1991). A Stable Crystalline
Carbene. J. Am. Chem. Soc..

[45] Herrmann W. A., Elison M., Fischer J., Köcher C., Artus G. R. J. (1995). Metal Complexes of N-Heterocyclic
Carbenes—A
New Structural Principle for Catalysts in Homogeneous Catalysis. Angew. Chem., Int. Ed. Engl..

[46] Herrmann W. A. (2002). N-Heterocyclic
Carbenes: A New Concept in Organometallic Catalysis. Angew. Chem., Int. Ed..

[47] Crabtree R. H. (2013). Abnormal,
Mesoionic and Remote N-Heterocyclic Carbene Complexes. Coord. Chem. Rev..

[48] Hopkinson M. N., Richter C., Schedler M., Glorius F. (2014). An Overview
of N-Heterocyclic
Carbenes. Nature.

[49] Soleilhavoup M., Bertrand G. (2015). Cyclic (Alkyl)­(Amino)
Carbenes (CAACs): Stable Carbenes
on the Rise. Acc. Chem. Res..

[50] Peris E. (2018). Smart N-Heterocyclic
Carbene Ligands in Catalysis. Chem. Rev..

[51] Vivancos A., Segarra C., Albrecht M. (2018). Mesoionic
and Related Less Heteroatom-Stabilized
N-Heterocyclic Carbene Complexes: Synthesis, Catalysis, and Other
Applications. Chem. Rev..

[52] Zhao Q., Meng G. R., Nolan S. P., Szostak M. (2020). N-Heterocyclic Carbene
Complexes in C–H Activation Reactions. Chem. Rev..

[53] Bellotti P., Koy M., Hopkinson M. N., Glorius F. (2021). Recent Advances in the Chemistry
and Applications of N-Heterocyclic Carbenes. Nat. Rev. Chem..

[54] Maity R., Sarkar B. (2022). Chemistry of Compounds Based on 1,2,3-Triazolylidene-Type
Mesoionic Carbenes. JACS Au.

[55] Vougioukalakis G. C., Grubbs R. H. (2010). Ruthenium-Based
Heterocyclic Carbene-Coordinated Olefin
Metathesis Catalysts. Chem. Rev..

[56] Wang F. J., Liu L. J., Wang W. F., Li S. K., Shi M. (2012). Chiral NHC-Metal-Based
Asymmetric Catalysis. Coord. Chem. Rev..

[57] Valente C., Çalimsiz S., Hoi K. H., Mallik D., Sayah M., Organ M. G. (2012). The Development
of Bulky Palladium NHC Complexes for
the Most-Challenging Cross-Coupling Reactions. Angew. Chem., Int. Ed..

[58] Riener K., Haslinger S., Raba A., Högerl M. P., Cokoja M., Herrmann W. A., Kühn F. E. (2014). Chemistry
of Iron N-Heterocyclic Carbene Complexes: Syntheses, Structures, Reactivities,
and Catalytic Applications. Chem. Rev..

[59] Iglesias M., Oro L. A. (2018). A Leap Forward in
Iridium–NHC Catalysis: New
Horizons and Mechanistic Insights. Chem. Soc.
Rev..

[60] Bera S. S., Szostak M. (2022). Cobalt–N-Heterocyclic
Carbene Complexes in Catalysis. ACS Catal..

[61] Yang S., Zhou T., Yu X., Szostak M. (2023). Ag–NHC Complexes
in The π-Activation of Alkynes. Molecules.

[62] Gao P., Szostak M. (2023). Hydration Reactions
Catalyzed by Transition Metal–NHC
(NHC = N-Heterocyclic Carbene) Complexes. Coord.
Chem. Rev..

[63] Zhou T., Utecht-Jarzyńska G., Szostak M. (2024). Ring-Expanded N-Heterocyclic
Carbene (reNHC) Complexes: Applications in Transition Metal Catalysis. Coord. Chem. Rev..

[64] Zhang J., Rahman M. M., Zhao Q., Feliciano J., Bisz E., Dziuk B., Lalancette R., Szostak R., Szostak M. (2022). N-Heterocyclic Carbene Complexes
of Nickel­(II) from Caffeine and Theophylline: Sustainable Alternative
to Imidazol-2-ylidenes. Organometallics.

[65] Lei P., Wang Y., Zhang C., Hu Y., Feng J., Ma Z., Liu X., Szostak R., Szostak M. (2022). Sonogashira Cross-Coupling
of Aryl Ammonium Salts by Selective C–N Activation Catalyzed
by Air- and Moisture-Stable, Highly Active [Pd­(NHC)­(3-CF_3_-An)­Cl_2_] (An = Aniline) Precatalysts. Org. Lett..

[66] Zhou T., Gao P., Bisz E., Dziuk B., Lalancette R., Szostak R., Szostak M. (2022). Well-defined, Air- and Moisture-stable
Palladium–imidazo­[1,5-*a*]­pyridin-3-ylidene
Complexes: A Versatile Catalyst Platform for Cross-Coupling Reactions
by L-shaped NHC Ligands. Catal. Sci. Technol..

[67] Gao P., Xu J., Zhou T., Liu Y., Bisz E., Dziuk B., Lalancette R., Szostak R., Zhang D., Szostak M. (2023). L-Shaped Heterobidentate
Imidazo­[1,5-a]­pyridin-3-ylidene­(N,C)-Ligands for Oxidant-Free Au^I^/Au^III^ Catalysis. Angew.
Chem., Int. Ed..

[68] Yang S., Yu X., Liu Y., Tomasini M., Caporaso L., Poater A., Cavallo L., Cazin C. S. J., Nolan S. P., Szostak M. (2023). Suzuki–Miyaura
Cross-Coupling of Amides by N–C Cleavage Mediated by Air-Stable,
Well-Defined [Pd­(NHC)­(sulfide)­Cl_2_] Catalysts: Reaction
Development, Scope, and Mechanism. J. Org. Chem..

[69] Zhou T., Gao P., Lalancette R., Szostak R., Szostak M. (2024). Gold-Catalysed Amine
Synthesis by Reductive Hydroamination of Alkynes with Nitroarenes. Nat. Chem..

[70] Utecht-Jarzyńska G., Shi S., Gao P., Jarzyński S., Rahman M., Lalancette R., Szostak R., Szostak M. (2024). IPr*^F^ – Highly
Hindered, Fluorinated N-Heterocyclic Carbenes. Chem. Eur. J..

[71] Diez-Gonzalez S., Nolan S. P. (2007). Stereoelectronic Parameters Associated with N-Heterocyclic
Carbene (NHC) Ligands: A Quest for Understanding. Coord. Chem. Rev..

[72] Gómez-Suárez A., Nelson D. J., Nolan S. P. (2017). Quantifying
and Understanding the
Steric Properties of N-Heterocyclic Carbenes. Chem. Commun..

[73] Chu W., Lalancette R., Zhou T., Bisz E., Szostak R., Dziuk B., Szostak M. (2022). CAAC–IPr*: Easily Accessible,
Highly Sterically Hindered Cyclic­(Alkyl)­(Amino)­Carbenes. Chem. Commun..

[74] Podchorodecka P., Dziuk B., Szostak R., Szostak M., Bisz E. (2023). IPr*Oxa –
A New Class of Sterically-Hindered, Wingtip-Flexible N,C-Chelating
Oxazole-Donor N-Heterocyclic Carbene Ligands. Dalton Trans..

[75] Zhang J., Liu T., Zhang G., Cai J., Wang Y., Tong J., Ma Y., Szostak R., Szostak M. (2024). Indazolin-3-ylidenes (Indy*): Easily
Accessible, Sterically-Hindered Indazole-Derived N-Heterocyclic Carbenes
and Their Application in Gold Catalysis. Dalton
Trans..

[76] Huynh, H. V. The Organometallic Chemistry of N-Heterocyclic Carbenes; Wiley: Hoboken, 2017; pp 1–368.

[77] Huynh H. V. (2018). Electronic
Properties of N-Heterocyclic Carbenes and Their Experimental Determination. Chem. Rev..

[78] Jacobsen H., Correa A., Poater A., Costabile C., Cavallo L. (2009). Understanding the M­(NHC)­(NHC= N-Heterocyclic Carbene)­Bond. Coord. Chem. Rev..

[79] Gusev D. G. (2009). Donor Properties
of a Series of Two-Electron Ligands. Organometallics.

[80] Kelly R. A., Clavier H., Giudice S., Scott N. M., Stevens E. D., Bordner J., Samardjiev I., Hoff C. D., Cavallo L., Nolan S. P. (2008). Determination
of
N-Heterocyclic Carbene (NHC) Steric and Electronic Parameters Using
the [(NHC)­Ir­(CO)_2_Cl] System. Organometallics.

[81] Zhang J., Wang Y., Zhang Y., Liu T., Fang S., Wang R., Ma Y., Fang R., Szostak R., Szostak M. (2022). Application of Indazolin-3-ylidenes
in Catalysis: Steric
Tuning of Nonclassical Formally Normal N-Heterocyclic Carbenes with
Dual Electronic Character for Catalysis. Organometallics.

[82] Zhang J., Li T., Li X., Lv A., Li X., Wang Z., Wang R., Ma Y., Fang R., Szostak R., Szostak M. (2022). Thiazol-2-ylidenes
as N-Heterocyclic Carbene Ligands
with Enhanced Electrophilicity for Transition Metal Catalysis. Comm. Chem..

[83] Zhang J., Li X., Li T., Zhang G., Wan K., Ma Y., Fang R., Szostak R., Szostak M. (2022). Copper­(I)–Thiazol-2-ylidenes:
Highly Reactive N-Heterocyclic Carbenes for the Hydroboration of Terminal
and Internal Alkynes. Ligand Development, Synthetic Utility, and Mechanistic
Studies. ACS Catal..

[84] Huang J., Grasa G., Nolan S. P. (1999). General
and Efficient Catalytic Amination
of Aryl Chlorides Using a Palladium/Bulky Nucleophilic Carbene System. Org. Lett..

[85] Voloshkin V. A., Zorba L. P., Nolan S. P. (2025). The Influential IPr: 25 Years After
its Discovery. Chem. Sci..

[86] Gradel B., Brenner E., Schneider R. L., Fort Y. (2001). Nickel-Catalysed Amination
of Aryl Chlorides Using a Dihydroimidazoline Carbene Ligand. Tetrahedron Lett..

[87] Meng G., Lei P., Szostak M. (2017). A General Method for
Two-Step Transamidation of Secondary
Amides Using Commercially Available, Air- and Moisture-Stable Palladium/NHC
(N-Heterocyclic Carbene) Complexes. Org. Lett..

[88] Huang J., Schanz H.-J., Stevens E. D., Nolan S. P. (1999). Stereoelectronic
Effects Characterizing Nucleophilic Carbene Ligands Bound to the Cp*RuCl
(Cp* = η^5^-C_5_Me_5_) Moiety: A
Structural and Thermochemical Investigation. Organometallics.

[89] Stauffer S. R., Lee S., Stambuli J. P., Hauck S. I., Hartwig J. F. (2000). High Turnover Number
and Rapid, Room-Temperature Amination of Chloroarenes Using Saturated
Carbene Ligands. Org. Lett..

[90] Cheng J., Trudell M. L. (2001). Synthesis of N-Heteroaryl-7-azabicyclo[2.2.1]­heptane
Derivatives via Palladium-Bisimidazol-2-ylidene Complex Catalyzed
Amination Reactions. Org. Lett..

[91] Viciu M. S., Germaneau M. F., Navarro-Fernandez O., Stevens E. W., Nolan S. P. (2002). Activation
and Reactivity of (NHC)­Pd­(allyl)Cl (NHC) N-Heterocyclic Carbene) Complexes
in Cross-Coupling Reactions. Organometallics.

[92] Ernst J. B., Schwermann C., Yokota G.-I., Tada M., Muratsugu S., Doltsinis N. L., Glorius F. (2017). Molecular Adsorbates Switch on Heterogeneous
Catalysis: Induction of Reactivity by N-Heterocyclic Carbenes. J. Am. Chem. Soc..

[93] Chen C., Li Y.-F., Yang L.-M. (2007). Efficient Synthesis of Triarylamines
Catalyzed by Palladium/N-heterocyclic Carbene. J. Mol. Catal. A: Chem..

[94] Zhang X., Xie W., Chen W. (2010). Palladium/Imidazolium
Salt as a Versatile Catalyst
for Sequential Coupling Reactions of Aryl Dihalides to Unsymmetrically
Substituted Arenes. Tetrahedron.

[95] Omar-Amrani R., Schneider R., Fort Y. (2004). Novel Synthetic Strategy of N-Arylated
Heterocycles via Sequential Palladium-Catalyzed Intra- and Inter-Arylamination
Reactions. Synthesis.

[96] Ackermann L. (2005). General and
Efficient Indole Syntheses Based on Catalytic Amination Reactions. Org. Lett..

[97] Kaspar L. T., Ackermann L. (2005). Three-Component Indole Synthesis Using *Ortho*-dihaloarenes. Tetrahedron.

[98] Ackermann L., Sandmann R., Schinkel M., Kondrashov M. V. (2009). Palladium-Catalyzed
Sequential Indole Synthesis Using Sterically Hindered Amines. Tetrahedron.

[99] Arai K., Yamamoto Y. (1990). Metabolic Products of Aspergillus
terreus. X.: Biosynthesis
of Asterriquinones. Chem. Pharm. Bull..

[100] Yang L., Bian H., Mai W., Mao P., Xiao Y., Wei D., Qu L. (2015). Synthesis of Tetrahydropyrimidinium
Salts and Their in Situ Catalytic Activities Towards the Buchwald–Hartwig
Amination Reaction Under Microwave Irradiation. Turk. J. Chem..

[101] Küçükbay H., Yilmaz Ü., Yavuz K., Bugday N. (2015). Synthesis, Characterization, and Microwave-Assisted
Catalytic Activity in Heck, Suzuki, Sonogashira, and Buchwald–Hartwig
Cross-Coupling Reactions of Novel Benzimidazole Salts Bearing N-phthalimidoethyl
and Benzyl Moieties. Turk. J. Chem..

[102] Zhu Y.-Q., Zhang R., Sang W., Wang H.-J., Wu Y., Yu B.-Y., Zhang J.-C., Cheng H., Chen C. (2020). Ligand-Controlled
Palladium Catalysis Enables Switch Between Mono- and Di-arylation
of Primary Aromatic Amines with 2-Halobenzothiazoles. Org. Chem. Front..

[103] Wheaton C. A., Bow J.-P. J., Stradiotto M. (2013). New Phosphine-Functionalized
NHC Ligands: Discovery of an Effective Catalyst for the Room-Temperature
Amination of Aryl Chlorides with Primary and Secondary Amines. Organometallics.

[104] Merschel A., Glodde T., Neumann B., Stammler H.-G., Ghadwal R. S. (2021). Nickel-Catalyzed
Intramolecular 1,2-Aryl Migration
of Mesoionic Carbenes (iMICs). Angew. Chem.,
Int. Ed..

[105] Böhm V. P. W., Gstöttmayr C.
W. K., Weskamp T., Herrmann W. A. (2000). N-Heterocyclic carbenes Part 26. N-Heterocyclic Carbene
Complexes of Palladium(0): Synthesis and Application in the Suzuki
Cross-Coupling Reaction. J. Organomet. Chem..

[106] Titcomb L. R., Caddick S., Cloke F. G. N., Wilson D. J., McKerrecher D. (2001). Unexpected Reactivity of Two-coordinate
Palladium–Carbene
Complexes; Synthetic and Catalytic Implications. Chem. Commun..

[107] Caddick S., Cloke F.G. N., Clentsmith G. K. B., Hitchcock P. B., McKerrecher D., Titcomb L. R., Williams M. R.V. (2001). An Improved
Synthesis of Bis­(1,3-di-N-tert-butylimidazol-2-ylidene)­Palladium(0)
and Its Use in C–C and C–N Coupling Reactions. J. Organomet. Chem..

[108] Lerma I. C., Cawley M. J., Cloke F. G. N., Arentsen K., Scott J. S., Pearson S. E., Hayler J., Caddick S. (2005). Studies on
Pd/imidazolium Salt Protocols for Aminations of Aryl Bromides and
Iodides Using Lithium Hexamethyldisilazide (LHMDS). J. Organomet. Chem..

[109] Arentsen K., Caddick S., Cloke F. G. N. (2005). On the
Efficiency
of Two-coordinate Palladium(0) N-heterocyclic Carbene Complexes in
Amination and Suzuki–Miyaura Reactions of Aryl Chlorides. Tetrahedron.

[110] Jackstell R., Gomez Andreu M., Frisch A., Selvakumar K., Zapf A., Klein H., Spannenberg A., Rottger D., Briel O., Karch R., Beller M. (2002). A Highly Efficient
Catalyst for the Telomerization of 1,3-Dienes with Alcohols: First
Synthesis of a Monocarbenepalladium(0)–Olefin Complex. Angew. Chem., Int. Ed..

[111] Selvakumar K., Zapf A., Spannenberg A., Beller M. (2002). Synthesis of Monocarbenepalladium(0) Complexes and
Their Catalytic Behavior in Cross-Coupling Reactions of Aryldiazonium
Salts. Chem. Eur. J..

[112] Frisch A. C., Zapf A., Briel O., Kayser B., Shaikh N., Beller M. (2004). Comparison of Palladium
Carbene and
Palladium Phosphine Catalysts for Catalytic Coupling Reactions of
Aryl Halides. J. Mol. Catal. A: Chem..

[113] Gooßen L. J., Paetzold J., Briel O., Rivas-Nass A., Karch R., Kayser B. (2005). Buchwald–Hartwig
Aminations
of Aryl Chlorides: A Practical Protocol Based on Commercially Available
Pd(0)-NHC Catalysts. Synlett.

[114] Viciu M. S., Germaneau R. F., Navarro-Fernandez O., Stevens E. D., Nolan S. P. (2002). Activation and Reactivity of (NHC)­Pd­(allyl)­Cl
(NHC) = N-Heterocyclic Carbene) Complexes in Cross-Coupling Reactions. Organometallics.

[115] Cämmerer S. S., Viciu M. S., Stevens E. D., Nolan S. P. (2003). Use of
(NHC)­Pd­(*η*
^3^-allyl)Cl (NHC = N-Heterocyclic
Carbene) in a Palladium-mediated Approach to *Cryptocarya* Alkaloids. Synlett.

[116] Navarro O., Kaur H., Mahjoor P., Nolan S. P. (2004). Cross-Coupling
and Dehalogenation Reactions Catalyzed by (N-Heterocyclic carbene)­Pd­(allyl)­Cl
Complexes. J. Org. Chem..

[117] Viciu M. S., Navarro O., Germaneau R. F., Kelly R. A., Sommer W., Marion N., Stevens E. D., Cavallo L., Nolan S. P. (2004). Synthetic and Structural Studies
of (NHC)­Pd­(allyl)Cl Complexes (NHC = N-heterocyclic carbene). Organometallics.

[118] Galardon E., Ramdeehul S., Brown J. M., Cowley A., Hii K. K., Jutand A. (2002). Profound Steric Control of Reactivity
in Aryl Halide Addition to Bisphosphane Palladium(0) Complexes. Angew. Chem., Int. Ed..

[119] Marion N., Navarro O., Mei J., Stevens E. D., Scott N. M., Nolan S. P. (2006). Modified (NHC)­Pd­(allyl)­Cl
(NHC =
N-Heterocyclic Carbene) Complexes for Room-Temperature Suzuki-Miyaura
and Buchwald–Hartwig Reactions. J. Am.
Chem. Soc..

[120] Navarro O., Marion N., Mei J., Nolan S. P. (2006). Rapid Room
Temperature Buchwald–Hartwig and Suzuki–Miyaura Couplings
of Heteroaromatic Compounds Employing Low Catalyst Loadings. Chem. Eur. J..

[121] Cawley M. J., Cloke F. G. N., Fitzmaurice R. J., Pearson S. E., Scott J. S., Caddick S. (2008). Development of a Practical
Buchwald–Hartwig Amine Arylation Protocol Using a Conveniently
Prepared (NHC)­Pd­(R-allyl)Cl Catalyst. Org. Biomol.
Chem..

[122] Egbert J.
D., Chartoire A., Slawin A. M. Z., Nolan S. P. (2011). Synthesis,
Characterization, and Reactivity of N-Heterocyclic Carbene Palladium­(II)
Hydroxide Dimers. Organometallics.

[123] Schoeps D., Sashuk V., Ebert K., Plenio H. (2009). Solvent-Resistant
Nanofiltration of Enlarged (NHC)­Pd­(allyl)Cl Complexes for Cross-Coupling
Reactions. Organometallics.

[124] Hoyos M., Sprick R. S., Wang C., Turner M. L., Navarro O. (2012). (N-Heterocyclic Carbene)-Pd Catalyzed
Synthesis of
Poly­(triarylamine)­s by Buchwald–Hartwig Coupling of Aryl Chlorides. J. Polym. Sci., Part A: Polym. Chem..

[125] Sprick R. S., Hoyos M., Navarro O., Turner M. L. (2012). Synthesis
of Poly­(triarylamine)­s by C–N Coupling Catalyzed by (N-Heterocyclic
Carbene)-Palladium Complexes. React. Funct.
Polym..

[126] Abi
Fayssal S., Naret T., Huc V., Buendia J., Martini C., Schulz E. (2021). Benzyloxycalix­[8]­arene Supported
Pd–NHC Cinnamyl Complexes for Buchwald–Hartwig C–N
Cross-Couplings. Catal. Sci. Technol..

[127] Bergbreiter D. E., Su H.-L., Koizumi H., Tian J. (2011). Polyisobutylene-Supported
N-Heterocyclic Carbene Palladium Catalysts. J. Organomet. Chem..

[128] Vieille-Petit L., Luan X., Mariz R., Blumentritt S., Linden A., Dorta R. (2009). A New Class of Stable,
Saturated
N-Heterocyclic Carbenes with N-Naphthyl Substituents: Synthesis, Dynamic
Behavior, and Catalytic Potential. Eur. J. Inorg.
Chem..

[129] Rahman M. M., Gao P., Zhao Q., Lalancette R., Szostak R., Szostak M. (2023). **[**Au­(Np#)­Cl]: Highly
Reactive and Broadly Applicable Au­(I)–NHC Catalysts for Alkyne
π-Activation Reactions. Catal. Sci. Technol..

[130] Rahman M. M., Zhao Q., Meng G., Szostak R., Szostak M. (2022). [Ni­(Np#)­(η5-Cp)­Cl]: Flexible,
Sterically Bulky,
Well-Defined, Highly Reactive Complex for Nickel-Catalyzed Cross-Coupling. Organometallics.

[131] Dastgir S., Coleman K. S., Cowley A. R., Green M. L. H. (2010). Synthesis,
Structure, and Temperature-Dependent Dynamics of Neutral Palladium
Allyl Complexes of Annulated Diaminocarbenes and Their Catalytic Application
for C-C and C-N Bond Formation Reactions. Organometallics.

[132] Fang W., Deng Q., Xu M., Tu T. (2013). Highly Efficient
Aminocarbonylation of Iodoarenes at Atmospheric Pressure Catalyzed
by a Robust Acenaphthoimidazolyidene Allylic Palladium Complex. Org. Lett..

[133] Berthon-Gelloz G., Siegler M. A., Spek A. L., Tinant B., Reek J. N. H., Markó I. E. (2010). IPr* an Easily Accessible Highly
Hindered *N*-heterocyclic Carbene. Dalton Trans..

[134] Utecht-Jarzyńska G., Jarzyński S., Rahman M. M., Meng G., Lalancette R., Szostak R., Szostak M. (2024). IPr# Complexes Highly-Hindered, Sterically-Bulky
Cu­(I) and Ag­(I) N-Heterocyclic Carbenes: Synthesis, Characterization,
and Reactivity. Organometallics.

[135] Chartoire A., Frogneux X., Nolan S. P. (2012). An Efficient
Palladium-NHC
(NHC = N-Heterocyclic Carbene) and Aryl Amination Pre-Catalyst: [Pd­(IPr*)­(cinnamyl)­Cl]. Adv. Synth. Catal..

[136] Bastug G., Nolan S. P. (2014). [Pd­(IPr*OMe)­(cin)­Cl] (cin = Cinnamyl):
A Versatile Catalyst for C–N and C–C Bond Formation. Organometallics.

[137] Chartoire A., Boreux A., Martin A. R., Nolan S. P. (2013). Solvent-free
Arylamination Catalysed by [Pd­(NHC)] Complexes. RSC Adv..

[138] Pommella A., Tomaiuolo G., Chartoire A., Caserta S., Toscano G., Nolan S. P., Guido S. (2013). Palladium-N-heterocyclic
Carbene (NHC) Catalyzed C–N Bond Formation in a Continuous
Flow Microreactor. Effect of Process Parameters and Comparison with
Batch Operation. Chem. Eng. J..

[139] Perazzo A., Tomaiuolo G., Sicignano L., Toscano G., Meadows R. E., Nolan S. P., Guido S. (2015). A Microfluidic
Approach for Flexible and Efficient Operation of a Cross-Coupling
Reactive Flow. RSC Adv..

[140] Chartoire A., Claver C., Corpet M., Krinsky J., Mayen J., Nelson D., Nolan S. P., Peñafiel I., Woodward R., Meadows R. E. (2016). Recyclable NHC Catalyst
for the Development
of a Generalized Approach to Continuous Buchwald–Hartwig Reaction
and Workup. Org. Process Res. Dev..

[141] Falß S., Tomaiuolo G., Perazzo A., Hodgson P., Yaseneva P., Zakrzewski J., Guido S., Lapkin A., Woodward R., Meadows R. E. (2016). A Continuous
Process for Buchwald–Hartwig
Amination at Micro-, Lab-, and Mesoscale Using a Novel Reactor Concept. Org. Process Res. Dev..

[142] Yaseneva P., Hodgson P., Zakrzewski J., Falß S., Meadows R. E., Lapkin A. A. (2016). Continuous Flow
Buchwald–Hartwig Amination of a Pharmaceutical Intermediate. React. Chem. Eng..

[143] Marelli E., Chartoire A., Le Duc G., Nolan S. P. (2015). Arylation
of Amines in Alkane Solvents by using Well-Defined Palladium–N-Heterocyclic
Carbene Complexes. ChemCatChem..

[144] Ruhling A., Rakers L., Glorius F. (2017). Long Alkyl
Chain NHC
Palladium Complexes for the Amination and Hydrodehalogenation of Aryl
Chlorides in Lipophilic Media. ChemCatChem..

[145] Sharif S., Day J., Hunter H. N., Lu Y., Mitchell D., Rodriguez M. J., Organ M. G. (2017). Cross-Coupling of
Primary Amides to Aryl and Heteroaryl Partners Using (DiMeIHept^Cl^)Pd Promoted by Trialkylboranes or B­(C_6_F_5_)_3_. J. Am. Chem. Soc..

[146] Semeniuchenko V., Sharif S., Rana N., Chandrasoma N., Braje W. M., Baker R. T., Manthorpe J. M., Pietro W. J., Organ M. G. (2024). Experimental Evidence for Zerovalent
Pd­(NHC) as a CompetentCatalyst in C–N Cross-Coupling (NHC =
DiMeIHept^Cl^). J. Am. Chem. Soc..

[147] Topchiy M. A., Dzhevakov P. B., Rubina M. S., Morozov O. S., Asachenko A. F., Nechaev M. S. (2016). Solvent-Free Buchwald–Hartwig
(Hetero)­arylation of Anilines, Diarylamines, and Dialkylamines Mediated
by Expanded-Ring N-Heterocyclic Carbene Palladium Complexes. Eur. J. Org. Chem..

[148] Zhang Y., Lavigne G., Lugan N., César V. (2017). Buttressing
Effect as a Key Design Principle towards Highly Efficient Palladium/N-Heterocyclic
Carbene Buchwald–Hartwig Amination Catalysts. Chem. Eur. J..

[149] Fukaya N., Mizusaki T., Hatakeyama K., Seo Y., Inaba Y., Matsumoto K., Lee V. Y., Takagi Y., Kuwabara J., Kanbara T., Choe Y.-K., Choi J.-C. (2019). [Pd­(4-R_3_Si-IPr)­(allyl)­Cl], a Family of Silyl-Substituted Pd–NHC
Complexes: Catalytic Systems for the Buchwald–Hartwig Amination. Organometallics.

[150] Mizusaki T., Matsumoto K., Takeuchi K., Fukaya N., Takagi Y., Choi J.-C. (2019). Direct Installation of a Silyl Linker
on Ready-Made NHC Ligands: Immobilized NHC-Pd Complex for Buchwald–Hartwig
Amination. Organometallics.

[151] Winkler A., Brandhorst K., Freytag M., Jones P. G., Tamm M. (2016). Palladium­(II) Complexes
with Anionic N-Heterocyclic Carbene–Borate
Ligands as Catalysts for the Amination of Aryl Halides. Organometallics.

[152] Yin J., Rainka M. P., Zhang X.-X., Buchwald S. L. (2002). A Highly
Active
Suzuki Catalyst for the Synthesis of Sterically Hindered Biaryls:
Novel Ligand Coordination. J. Am. Chem. Soc..

[153] Zinser C. M., Warren K. G., Nahra F., Al-Majid A., Barakat A., Islam M. S., Nolan S. P., Cazin C. S. J. (2019). Palladate
Precatalysts for the Formation of C–N and C–C Bonds. Organometallics.

[154] Ouyang J.-S., Liu S., Pan B., Zhang Y., Liang H., Chen B., He X., Chan A. S. C., Chan W. T. K., Sun T.-Y., Wu Y.-D., Qiu L. (2021). A Bulky and
Electron-Rich N-Heterocyclic Carbene–Palladium Complex (SIPr)^Ph2^Pd­(cin)­Cl: Highly Efficient and Versatile for the Buchwald–Hartwig
Amination of (Hetero)­aryl Chlorides with (Hetero)­aryl Amines at Room
Temperature. ACS Catal..

[155] Ouyang J.-S., Zhang X., Pan B., Zou H., Chan A. S. C., Qiu L. (2023). Solvent-Free Buchwald–Hartwig
Amination of Heteroaryl Chlorides by N-Heterocyclic Carbene–Palladium
Complex (SIPr)^Ph2^Pd­(cin)Cl at Room Temperature. Org. Lett..

[156] Liu Y., Scattolin T., Gobbo A., Beliš M., Van Hecke K., Nolan S. P., Cazin C. S. J. (2022). A Simple Synthetic
Route to Well-Defined [Pd­(NHC)­Cl­(1-^t^Buindenyl)] Pre-catalysts
for Cross-Coupling Reactions. Eur. J. Inorg.
Chem..

[157] Espinosa M. R., Doppiu A., Hazari N. (2020). Differences in the
Performance of Allyl Based Palladium Precatalysts for Suzuki-Miyaura
Reactions. Adv. Synth. Catal..

[158] Gribanov P. S., Philippova A. N., Topchiy M. A., Minaeva L. I., Asachenko A. F., Osipov S. N. (2022). General Method of Synthesis of 5-(Het)­arylamino-1,2,3-triazoles
via Buchwald–Hartwig Reaction of 5-Amino- or 5-Halo-1,2,3-triazoles. Molecules.

[159] Gribanov P. S., Philippova A. N., Topchiy M. A., Lypenko D. A., Dmitriev A. V., Tokarev S. D., Smol’yakov A. F., Rodionov A. N., Asachenko A. F., Osipov S. N. (2024). Synthesis of 5-(Aryl)­amino-1,2,3-triazole-containing
2,1,3-Benzothiadiazoles via Azide–Nitrile Cycloaddition Followed
by Buchwald–Hartwig Reaction. Molecules.

[160] Viciu M. S., Kissling R. M., Stevens E. D., Nolan S. P. (2002). An Air-Stable
Palladium/N-Heterocyclic Carbene Complex and Its Reactivity in Aryl
Amination. Org. Lett..

[161] Yang S., Zhou T., Yu X., Nolan S. P., Szostak M. (2024). [Pd­(NHC)­(μ-Cl)­Cl]_2_: The Highly Reactive
Air- and Moisture-Stable, Well-Defined Pd­(II)-N-Heterocyclic Carbene
(NHC) Complexes for Cross-Coupling Reactions. Acc. Chem. Res..

[162] Zhang J., Li T., Li X., Zhang G., Fang S., Yan W., Li X., Yang X., Ma Y., Szostak M. (2022). An Air-Stable, Well-Defined Palladium–BIAN–NHC
Chloro Dimer: A Fast-Activating, Highly Efficient Catalyst for Cross-coupling. Chem. Commun..

[163] Zhang J., Cai J., Li X., Zhang G., Yan W., Li R., Tong J., Szostak M. (2024). Air-stable, Well-Defined
Palladium–BIAN–NHC Chloro Dimer: Highly Efficient N-Heterocyclic
Carbene (NHC) Catalyst Platform for Buchwald–Hartwig C–N
Cross-Coupling Reactions. J. Catal..

[164] Li J., Cui M., Yu A., Wu Y. (2007). Carbene Adduct of Cyclopalladated
Ferrocenylimine as an Efficient Catalyst for the Amination of Aryl
Chlorides. J. Organomet. Chem..

[165] Mu B., Li J., Wu Y. (2013). Carbene Adduct
of Cyclopalladated
Ferrocenylimine-assisted Synthesis of Aminopyridine Derivatives by
the Amination of Chloropyridines with Primary and Secondary Amines. Appl. Organometal. Chem..

[166] Xu C., Li H.-M., Liu H., Zhang Z.-Q., Wang Z.-Q., Fu W.-J., Zhang Y.-Q. (2012). N-Heterocyclic
Carbene Adducts of
Cyclopalladated Ferrocenylpyridine: Synthesis, Structural Characterization
and Reusable Catalytic System for Suzuki and Amination of Aryl Chlorides
in Poly­(ethylene glycol-400). Inorg. Chim. Acta.

[167] Viciu M. S., Kelly R. A., Stevens E. D., Naud F., Studer M., Nolan S. P. (2003). Synthesis, Characterization,
and
Catalytic Activity of N-Heterocyclic Carbene (NHC) Palladacycle Complexes. Org. Lett..

[168] Broggi J., Clavier H., Nolan S. P. (2008). N-Heterocyclic Carbenes
(NHCs) Containing N-C-Palladacycle Complexes: Synthesis and Reactivity
in Aryl Amination Reactions. Organometallics.

[169] Deng Q., Zhang Y., Zhu H., Tu T. (2017). Robust Acenaphthoimidazolylidene
Palladacycles: Highly Efficient Catalysts for the Amination of N-heteroaryl
Chlorides. Chem. Asian J..

[170] Anusha G., Reddy M. V. K., Reddy P. V. G. (2018). SingaCycle^TM^-A1-Catalyzed Successive Suzuki-Miyaura and Buchwald Couplings
for the Synthesis of Various New Pyridine Analogues. ChemistrySelect.

[171] Kantchev E. A. B., Peh G.-R., Zhang C., Ying J. Y. (2008). Practical
Heck–Mizoroki Coupling Protocol for Challenging Substrates
Mediated by an N-Heterocyclic Carbene-Ligated Palladacycle. Org. Lett..

[172] Zuo B., Shao H., Qu E., Ma Y., Li W., Huang M., Deng Q. (2021). An Alkoxy Modified N-Heterocyclic
Carbene-Palladacycle: Synthesis, Characterization and Application
towards Buchwald–Hartwig and Suzuki-Miyaura Coupling Reactions. ChemistrySelect.

[173] Liu F., Hu Y.-Y., Li D., Zhou Q., Lu J.-M. (2018). N-Heterocyclic
Carbene-Palladacyclic Complexes: Synthesis, Characterization and Their
Applications in the C-N Coupling and Arylation of Ketones using Aryl
Chlorides. Tetrahedron.

[174] Ostrowska S., Czapik A., Kwit M., Nolan S. P. (2023). Palladates
of the [NHC·H]­[Pd­(NH_2_)­(CC)­Cl_2_] Type as
Effective Pre-catalysts for the Formation of C–C and C–N
Bonds. ChemCatChem..

[175] Navarro O., Marion N., Scott N. M., González J., Amoroso D., Bell A., Nolan S. P. (2005). Synthesis
of Novel
(NHC)­Pd­(acac)Cl Complexes (acac = acetylacetonate) and Their Activity
in Cross-Coupling Reactions. Tetrahedron.

[176] Baba S., Ogura T., Kawaguchi S. (1974). Reaction of
Bis­(acetylacetonato)­Palladium­(II) with Triphenylphosphine and Nitrogen
Bases. Bull. Chem. Soc. Jpn..

[177] Marion N., Ecarnot E. C., Navarro O., Amoroso D., Bell A., Nolan S. P. (2006). (IPr)­Pd­(acac)­Cl:
An Easily Synthesized,
Efficient, and Versatile Precatalyst for C-N and C-C Bond Formation. J. Org. Chem..

[178] Marion N., de Frémont P., Puijk I. M., Ecarnot E. C., Amoroso D., Bell A., Nolan S. P. (2007). N-Heterocyclic Carbene–Palladium
Complexes [(NHC)­Pd-(acac)­Cl]: Improved Synthesis and Catalytic Activity
in Large-Scale Cross-Coupling Reactions. Adv.
Synth. Catal..

[179] Winkelmann O. H., Riekstins A., Nolan S. P., Navarro O. (2009). A Comparative
Study on (NHC)­Pd­(acac)Cl Complexes (NHC = N-heterocyclic carbene):
Indications for the Origin of the Different Reactivity of Saturated
and Unsaturated NHC in Cross-Coupling Reactions. Organometallics.

[180] Marion N., Navarro O., Stevens E. D., Ecarnot E. C., Bell A., Amoroso D., Nolan S. P. (2010). Modified [(IPr)­Pd­(R-acac)­Cl]
Complexes: Influence of the acac Substitution on the Catalytic Activity
in Aryl Amination. Chem. Asian J..

[181] Meiries S., Chartoire A., Slawin A. M. Z., Nolan S. P. (2012). [Pd­(IPr*)­(acac)­Cl]:
An Easily Synthesized, Bulky Precatalyst for C–N Bond Formation. Organometallics.

[182] Meiries S., Speck K., Cordes D. B., Slawin A. M. Z., Nolan S. P. (2013). [Pd­(IPr*OMe)­(acac)­Cl]:
Tuning the N-Heterocyclic Carbene
in Catalytic C–N Bond Formation. Organometallics.

[183] Tian X., Lin J., Zou S., Lv J., Huang Q., Zhu J., Huang S., Wang Q. (2018). [Pd­(IPr*R)­(acac)­Cl]:
Efficient Bulky Pd-NHC Catalyst for Buchwald- Hartwig C-N Cross-Coupling
Reaction. J. Organomet. Chem..

[184] Meiries S., Le Duc G., Chartoire A., Collado A., Speck K., Arachchige K. S. A., Slawin A. M. Z., Nolan S. P. (2013). Large yet Flexible N-Heterocyclic
Carbene Ligands for Palladium Catalysis. Chem.
Eur. J..

[185] Scott N. M., Nolan S. P. (2005). Stabilization of Organometallic Species
Achieved by the Use of N-Heterocyclic Carbene (NHC) Ligands. Eur. J. Inorg. Chem..

[186] Le Duc G., Meiries S., Nolan S. P. (2013). Effect of Electronic
Enrichment of NHCs on the Catalytic Activity of [Pd­(NHC)­(acac)­Cl]
in Buchwald–Hartwig Coupling. Organometallics.

[187] Subramaniyan V., Dutta B., Govindaraj A., Mani G. (2019). Facile Synthesis of Pd­(II) and Ni­(II) Pincer Carbene Complexes by
the Double C–H Bond Activation of a New Hexahydropyrimidine-based
Bis­(phosphine):Catalysis of C–N Couplings. Dalton Trans..

[188] Kremzow D., Seidel G., Lehmann C. W., Fürstner A. (2005). Diaminocarbene-
and Fischer-Carbene Complexes of Palladium and Nickel by Oxidative
Insertion: Preparation, Structure, and Catalytic Activity. Chem. Eur. J..

[189] Kim M., Shin T., Lee A., Kim H. (2018). Synergistic Ligand
Effect between N-Heterocyclic Carbene (NHC) and Bicyclic Phosphoramidite
(Briphos) Ligands in Pd-Catalyzed Amination. Organometallics.

[190] Ageshina A. A., Sterligov G. K., Rzhevskiy S. A., Topchiy M. A., Chesnokov G. A., Gribanov P. S., Melnikova E. K., Nechaev M. S., Asachenko A. F., Bermeshev M. V. (2019). Mixed er-NHC/Phosphine
Pd­(II) Complexes and Their Catalytic Activity in the Buchwald Hartwig
Reaction Under Solvent-Free Conditions. Dalton
Trans..

[191] Chesnokov G. A., Gribanov P. S., Topchiy M. A., Minaeva L. I., Asachenko A. F., Nechaev M. S., Bermesheva E. V., Bermeshev M. V. (2017). Solvent-free
Buchwald–Hartwig Amination with
Low Palladium Loadings. Mendeleev Commun..

[192] Scattolin T., Voloshkin V. A., Martynova E., Vanden Broeck S. M. P., Beliš M., Cazin C. S. J., Nolan S. P. (2021). Synthesis
and Catalytic Activity of Palladium Complexes Bearing N-Heterocyclic
Carbenes (NHCs) and 1,4,7-Triaza-9-phosphatricyclo[5.3.2.1] Tridecane
(CAP) Ligands. Dalton Trans..

[193] Yang J., Li P., Zhang Y., Wang L. (2014). Dinuclear
N-Heterocyclic Carbene Palladium­(II) Complexes as Efficient Catalysts
for the Buchwald–Hartwig Amination. J.
Organomet. Chem..

[194] Wheaton C. A., Stradiotto M. (2013). Probing the
Utility of Palladium
Complexes Supported by Morpholine-Functionalized N-Heterocyclic Carbene
Ligands in Buchwald–Hartwig Amination. Can. J. Chem..

[195] Esposito O., Gois P. M. P., de K. Lewis A. K., Caddick S., Cloke F. G. N., Hitchcock P. B. (2008). Alkylpalladium
N-Heterocyclic Carbene Complexes: Synthesis, Reactivity, and Catalytic
Properties. Organometallics.

[196] Love J. A., Morgan J. P., Trnka T. M., Grubbs R. H. (2002). A Practical
and Highly Active Ruthenium-Based Catalyst that Effects the Cross
Metathesis of Acrylonitrile. Angew. Chem., Int.
Ed..

[197] O’Brien J. C., Kantchev E. A. B., Valente C., Hadei N., Chass G. A., Lough A., Hopkinson A. C., Organ M. G. (2006). Easily Prepared Air- and Moisture-Stable Pd–NHC
(NHC = N-Heterocyclic Carbene) Complexes: A Reliable, User-Friendly,
Highly Active Palladium Precatalyst for the Suzuki–Miyaura
Reaction. Chem. Eur. J..

[198] Guillet S. G., Voloshkin V. A., Saab M., Beliš M., Van Hecke K., Nahra F., Nolan S. P. (2020). Designing a Novel
Synthetic Route to [Pd­(Cl)_2_(L)­(py)] (L = NHC and PR_3_) Precatalysts. Chem. Commun..

[199] Organ M., Abdel-Hadi M., Avola S., Dubovyk I., Hadei N., Kantchev E. A. B., O’Brien C. J., Sayah M., Valente C. (2008). Pd-Catalyzed
Aryl Amination Mediated
by Well Defined, N-Heterocyclic Carbene (NHC)–Pd Precatalysts,
PEPPSI. Chem. Eur. J..

[200] Mennecke K., Kirschning A. (2008). Immobilization
of NHC-Bearing Palladium
Catalysts on Polyvinylpyridine; Applications in Suzuki–Miyaura
and Hartwig–Buchwald Reactions under Batch and Continuous-Flow
Conditions. Synthesis.

[201] Hoi K. H., Calimsiz S., Froese R. D. J., Hopkinson A. C., Organ M. G. (2011). Amination with Pd–NHC Complexes:
Rate and Computational
Studies on the Effects of the Oxidative Addition Partner. Chem. Eur. J..

[202] Tu T., Fang W., Jiang J. (2011). A Highly Efficient Precatalyst for
Amination of Aryl Chlorides: Synthesis, Structure and Application
of a Robust Acenaphthoimidazolylidene Palladium Complex. Chem. Commun..

[203] Nasielski J., Hadei N., Achonduh G., Kantchev E. A. B., O’Brien C. J., Lough A., Organ M. G. (2010). Structure–Activity
Relationship Analysis of Pd–PEPPSI Complexes in Cross-Couplings:
A Close Inspection of the Catalytic Cycle and the Precatalyst Activation
Model. Chem. Eur. J..

[204] Peng M., Gao H., Gu Y., Tan B. (2025). Hyper-Cross-Linked
Pyridine-Functionalized Bis­(imino)­acenaphthene-N-heterocyclic Carbene
(BIAN-NHC) Palladium Catalysts for Superior Suzuki-Miyaura and Buchwald–Hartwig
Coupling Reactions. ACS Appl. Polym. Mater..

[205] Hoi K. H., Calimsiz S., Froese R. D. J., Hopkinson A. C., Organ M. G. (2012). Amination with Pd–NHC Complexes:
Rate and Computational
Studies Involving Substituted Aniline Substrates. Chem. Eur. J..

[206] Hoi K. H., Organ M. G. (2012). Potassium 2,2,5,7,8-Pentamethylchroman-6-oxide:
A Rationally Designed Base for Pd-Catalysed Amination. Chem. Eur. J..

[207] Chartoire A., Frogneux X., Boreux A., Slawin A. M. Z., Nolan S. P. (2012). [Pd­(IPr*)­(3-Cl-pyridinyl)­Cl_2_]: A Novel and
Efficient PEPPSI Precatalyst. Organometallics.

[208] Hoi K. H., Coggan J. A., Organ M. G. (2013). Pd-PEPPSI-IPentCl:
An Effective Catalyst for the Preparation of Triarylamines. Chem. -Eur. J..

[209] Pompeo M., Froese R. D. J., Hadei N., Organ M. G. (2012). Pd-PEPPSI-IPent^Cl^: A Highly Effective Catalyst for the Selective Cross-Coupling
of Secondary Organozinc Reagents. Angew. Chem.,
Int. Ed..

[210] Fang W., Jiang J., Xu Y., Zhou J., Tu T. (2013). Novel Robust
Benzimidazolylidene Palladium Complexes: Synthesis,
Structure, and Catalytic Applications in Amination of Chloroarenes. Tetrahedron.

[211] Zhao Q., Rahman M., Zhou T., Yang S., Lalancette R., Szostak R., Szostak M. (2024). Wingtip-Flexible N-Heterocyclic
Carbenes: Unsymmetrical Connection between IMes and IPr. Angew. Chem., Int. Ed..

[212] Suzuki Y., Fukui N., Murakami K., Yorimitsu H., Osuka A. (2013). Amination of meso-Bromoporphyrins and 9-Haloanthracenes with Diarylamines
Catalyzed by a Palladium–PEPPSI Complex. Asian J. Org. Chem..

[213] Krinsky J. L., Martínez A., Godard C., Castillón S., Claver C. (2014). Modular Synthesis of Functionalisable Alkoxy-Tethered
N-Heterocyclic Carbene Ligands and an Active Catalyst for Buchwald–Hartwig
Aminations. Adv. Synth. Catal..

[214] Pompeo M., Farmer J. L., Froese R. D. J., Organ M. G. (2014). Room-Temperature
Amination of Deactivated Aniline and Aryl Halide Partners with Carbonate
Base Using a Pd-PEPPSI-IPent^Cl^-*o*-Picoline
Catalyst. Angew. Chem., Int. Ed..

[215] Zhang Y., César V., Storch G., Lugan N., Lavigne G. (2014). Skeleton Decoration
of NHCs by Amino Groups and its
Sequential Booster Effect on the Palladium-Catalyzed Buchwald–Hartwig
Amination. Angew. Chem., Int. Ed..

[216] Zhang Y., César V., Lavigne G. (2015). Efficient and Versatile
Buchwald–Hartwig Amination of (Hetero)­aryl Chlorides Using
the Pd–PEPPSI-IPr­(NMe_2_)_2_ Precatalyst
in the Presence of Carbonate Base. Eur. J. Org.
Chem..

[217] Sharif S., Rucker R. P., Chandrasoma N., Mitchell D., Rodriguez M. J., Froese R. D. J., Organ M. G. (2015). Selective
Monoarylation of Primary Amines Using the Pd-PEPPSI-IPent^Cl^ Precatalyst. Angew. Chem., Int. Ed..

[218] Wang F., Zhu L., Zhou Y., Bao X., Schaefer H. F. (2015). Is PdII-Promoted σ-Bond Metathesis Mechanism
Operative for the Pd–PEPPSI Complex-Catalyzed Amination of
Chlorobenzene with Aniline? Experiment and Theory. Chem. Eur. J..

[219] Sharif S., Mitchell D., Rodriguez M. J., Farmer J. L., Organ M. G. (2016). N-Heteroarylation of Optically Pure
α-Amino Esters using the Pd-PEPPSI-IPent^Cl^-*o*-picoline Pre-Catalyst. Chem. Eur.
J..

[220] Topchiy M. A., Zotova M. A., Masoud S. M., Mailyan A. K., Ananyev I. V., Nefedov S. E., Asachenko A. F., Osipov S. N. (2017). Fluorinated Unsymmetrical
N,N’-Diaryl Imidazolium
Salts—New Functionalized NHC-Ligand Precursors. Chem. Eur. J..

[221] Balogh J., Hlil A. R., El-Zoghbi I., Rafique M. G., Chouikhi D., Al-Hashimi M., Bazzi H. S. (2017). Phase-Separable Polyisobutylene Palladium-PEPPSI Precatalysts:
Synthesis and Application in Buchwald–Hartwig Amination. Macromol. Rapid Commun..

[222] Khadra A., Mayer S., Organ M. G. (2017). Pd-PEPPSI-IPent^Cl^: A Useful
Catalyst for the Coupling of 2-Aminopyridine Derivatives. Chem. Eur. J..

[223] Khadra A., Mayer S., Mitchell D., Rodriguez M. J., Organ M. G. (2017). A General Protocol for the Broad-Spectrum Cross-Coupling
of Nonactivated Sterically Hindered 1° and 2° Amines. Organometallics.

[224] Lan X.-B., Li Y., Li Y.-F., Shen D.-S., Ke Z., Liu F.-S. (2017). Flexible Steric Bulky Bis­(Imino)­acenaphthene (BIAN)-Supported
N-Heterocyclic Carbene Palladium Precatalysts: Catalytic Application
in Buchwald–Hartwig Amination in Air. J. Org. Chem..

[225] Chen C., Liu F.-S., Szostak M. (2021). BIAN-NHC Ligands in
Transition-Metal-Catalysis: A Perfect Union of Sterically Encumbered,
Electronically Tunable N-Heterocyclic Carbenes?. Chem. Eur. J..

[226] Richardson J., Ruble J. C., Love E. A., Berritt S. (2017). A Method for
Identifying and Developing Functional Group Tolerant Catalytic Reactions:
Application to the Buchwald–Hartwig Amination. J. Org. Chem..

[227] Huang F.-D., Xu C., Lu D.-D., Shen D.-S., Li T., Liu F.-S. (2018). Pd-PEPPSI-IPent^An^ Promoted Deactivated Amination
of Aryl Chlorides with Amines under Aerobic Conditions. J. Org. Chem..

[228] Cao Q., Nicholson W. I., Jones A. C., Browne D. L. (2019). Robust Buchwald–Hartwig
Amination Enabled by Ball-milling. Org. Biomol.
Chem..

[229] Davalos A. R., Sylvester E., Diver S. T. (2019). Macrocyclic N-Heterocyclic
Carbenes: Synthesis and Catalytic Applications. Organometallics.

[230] Lombardi C., Rucker R. P., Froese R. D. J., Sharif S., Champagne P. A., Organ M. G. (2019). Rate and Computational Studies for
Pd-NHC-Catalyzed Amination with Primary Alkylamines and Secondary
Anilines: Rationalizing Selectivity for Monoarylation versus Diarylation
with NHC Ligands. Chem. Eur. J..

[231] Zhang F.-Y., Lan X.-B., Xu C., Yao H.-G., Li T., Liu F.-S. (2019). Rigid Hindered N-Heterocyclic
Carbene Palladium Precatalysts:
Synthesis, Characterization and Catalytic Amination. Org. Chem. Front..

[232] Reddy M. V. K., Anusha G., Reddy P. V. G. (2020). Sterically Enriched
Bulky 1,3-Bis­(N,N’-aralkyl)-Benzimidazolium Based Pd-PEPPSI
Complexes for Buchwald–Hartwig Amination Reactions. New J. Chem..

[233] Chen Y.-C., Faver J. C., Ku A. F., Miklossy G., Riehle K., Bohren K. M., Ucisik M. N., Matzuk M. M., Yu Z., Simmons N. (2020). C–N Coupling of DNA-Conjugated (Hetero)­aryl
Bromides and Chlorides for DNA-Encoded Chemical Library Synthesis. Bioconjugate Chem..

[234] Hayhow T. G., Borrows R. E. A., Diène C. R., Fairley G., Fallan C., Fillery S. M., Scott J. S., Watson D. W. (2020). A Buchwald–Hartwig Protocol to Enable Rapid
Linker Exploration of Cereblon E3-Ligase PROTACs. Chem. Eur. J..

[235] Zheng D.-Z., Xiong H.-G., Song A.-X., Yao H.-G., Xu C. (2022). Buchwald–Hartwig Amination of Aryl Esters and Chlorides Catalyzed
by the Dianisole-Decorated Pd–NHC Complex. Org. Biomol. Chem..

[236] Ye Y., Liu Z., Wang Y., Zhang Y., Yin F., He Q., Peng J., Tan K., Shen Y. (2022). N-Indole-Substituted
N-heterocyclic Carbene Palladium Precatalysts: Synthesis, Characterization
and Catalytic Cross-Couplings. Tetrahedron Lett..

[237] Li D.-H., Lan X.-B., Song A.-X., Rahman M. M., Xu C., Huang F.-D., Szostak R., Szostak M., Liu F.-S. (2022). Buchwald–Hartwig
Amination of Coordinating Heterocycles Enabled by Large-but-Flexible
Pd-BIAN-NHC Catalysts. Chem. Eur. J..

[238] Suwal S., Rahman M., O’Brien G., Karambizi V. G., Wrotny M., Goodman M. S. (2022). Chemo-Selective
Syntheses of N-t-boc-Protected Amino Ester Analogs Through Buchwald–Hartwig
Amination. New J. Chem..

[239] Chernyshev V. M., Khazipov O. V., Shevchenko M. A., Pasyukov D. V., Burykina J. V., Minyaev M. E., Eremin D. B., Ananikov V. P. (2022). Discovery of the N–NHC Coupling Process under
the Conditions of Pd/NHC- and Ni/NHC-Catalyzed Buchwald–Hartwig
Amination. Organometallics.

[240] Rahman M. M., Meng G., Bisz E., Dziuk B., Lalancette R., Szostak R., Szostak M. (2023). I*t*Oct (I*t*Octyl) – Pushing the Limits of I*t*Bu: Highly Hindered Electron-rich N-Aliphatic N-Heterocyclic
Carbenes. Chem. Sci..

[241] Rahman M. M., Zhao Q., Meng G., Lalancette R., Szostak R., Szostak M. (2023). [IPr^#^–PEPPSI]:
A Well-Defined, Highly Hindered and Broadly Applicable Pd­(II)–NHC
(NHC = N-Heterocyclic Carbene) Precatalyst for Cross-Coupling Reactions. Molecules.

[242] Astakhov A. V., Chernenko A. Y., Kutyrev V. V., Ranny G. S., Minyaev M. E., Chernyshev V. M., Ananikov V. P. (2022). Selective Buchwald–Hartwig
Arylation of C-Amino-1,2,4-Triazoles and Other Coordinating Aminoheterocycles
Enabled by Bulky NHC Ligands and TPEDO Activator. Inorg. Chem. Front..

[243] Marigo N., Morgenstern B., Biffis A., Munz D. (2023). (CAAC)­Pd­(py)
Catalysts Disproportionate to Pd­(CAAC)_2_. Organometallics.

[244] Grebennikov N. O., Boiko D. A., Prima D. O., Madiyeva M., Minyaev M. E., Ananikov V. P. (2024). Boosting the Generality
of Catalytic
Systems by the Synergetic Ligand Effect in Pd-Catalyzed C-N Cross-Coupling. J. Catal..

[245] Saberov V. S., Korotkikh N. I., Avksentiev A. S., Yenya V. I., Rayenko G. F. (2024). Catalytic
Properties of Sterically
Shielded Carbene Complexes of Palladium in C–C and C–N
Coupling Reactions of Haloarenes. Theor Exp
Chem..

[246] Guillet S. G., Voloshkin V. A., Saab M., Beliš M., Van Hecke K., Nahra F., Nolan S. P. (2020). Understanding Existing
and Designing Novel Synthetic Routes to Pd-PEPPSI-NHC and Pd-PEPPSI-PR_3_ Pre-Catalysts. Chem. Commun..

[247] Peng J., He Q., Wen J., Zhang Y., Wang Y., Ye Y., Shen Y. (2024). *N*
**-**Indole-Substituted Imidazolylidene Pd-PEPPSI Precatalysts:
Enhanced Performance with a 3,5-Diisopropyl-4-indolyl Moiety. J. Org. Chem..

[248] Meng G., Kakalis L., Nolan S. P., Szostak M. (2019). A Simple ^1^H NMR Method for Determining the *σ*-Donor
Properties of N-Heterocyclic Carbenes. Tetrahedron
Lett..

[249] Jin Z., Guo S.-X., Gu X.-P., Qiu L.-L., Song H.-B., Fang J.-X. (2009). Highly Active, Well-Defined (Cyclopentadiene)­(N-Heterocyclic
Carbene)Palladium Chloride Complexes for Room-Temperature Suzuki–Miyaura
and Buchwald–Hartwig Cross-Coupling Reactions of Aryl Chlorides
and Deboronation Homocoupling of Arylboronic Acids. Adv. Synth. Catal..

[250] Huang P., Wang Y.-X., Yu H.-F., Lu J.-M. (2014). N-Heterocyclic
Carbene–Palladium­(II)–4,5-Dihydrooxazole Complexes:
Synthesis and Catalytic Activity toward Amination of Aryl Chlorides. Organometallics.

[251] Sun K.-X., Zhou J.-H., He Q.-W., Shao L.-X., Lu J.-M. (2020). N-Heterocyclic
Carbene-Pd­(II)-2-methyl-4,5-dihydrooxazole Complex
Catalyzed Highly Chemoselective Mono-Amination of Dichlorobenzenes. Tetrahedron.

[252] Sun K.-X., He Q.-W., Xu B.-B., Wu X.-T., Lu J.-M. (2018). Synthesis
of N-Heterocyclic Carbene–Pd^II^–2-Methyl-4,5-dihydrooxazole
Complexes and Their Application Toward Highly Chemoselective Mono-Suzuki–Miyaura
Coupling of Dichlorobenzenes. Asian J. Org.
Chem..

[253] Zhang Z.-M., Xu Y.-T., Shao L.-X. (2021). Synthesis of *N*-Heterocyclic
Carbene-Pd­(II)-5-Phenyloxazole Complexes
and Initial Studies of Their Catalytic Activity Toward the Buchwald–Hartwig
Amination of Aryl Chlorides. J. Organomet. Chem..

[254] Zhu L., Gao T.-T., Shao L.-X. (2011). Well-Defined
NHC-Pd­(II)-Im (NHC =
N-Heterocyclic Carbene; Im = 1-methylimidazole) Complexes Catalyzed
Amination of Aryl Chlorides. Tetrahedron.

[255] Zhu L., Ye Y.-M., Shao L.-X. (2012). Well-Defined
NHC-Pd­(II)-Im (NHC =
N-Heterocyclic Carbene; Im = 1-methylimidazole) Complex Catalyzed
C-N Coupling of Primary Amines with Aryl Chlorides. Tetrahedron.

[256] Chen W.-X., Shao L.-X. (2012). N-Heterocyclic Carbene–Palladium­(II)-1-Methylimidazole
Complex Catalyzed Amination between Aryl Chlorides and Amides. J. Org. Chem..

[257] Chen W.-X., Zhang C.-Y., Lu J.-M. (2013). Highly Efficient
Amination in Neat Water of Benzyl Chlorides with Dialkylformamides
Catalysed by N-Heterocyclic Carbene-Palladium­(II)-1-methylimidazole
Complex. J. Chem. Res..

[258] Chen M.-T., Vicic D. A., Turner M. L., Navarro O. (2011). (N-Heterocyclic
Carbene)­PdCl_2_ (TEA) Complexes: Studies on the Effectof
the “Throw-Away” Ligand in Catalytic Activity. Organometallics.

[259] Semeniuchenko V., Braje W. M., Organ M. G. (2021). Sodium Butylated
Hydroxytoluene: A Functional Group Tolerant, Eco-Friendly Base for
Solvent-Free, Pd-Catalysed Amination. Chem.
Eur. J..

[260] Chen M.-T., Hsu M., Lin M.-C., Lien Y.-C., Chen K.-W. (2022). Bis­(imino)­acenaphthene *N*-Heterocyclic
Carbene Palladium Complexes Bearing Tertiary Amines: Structural and
Catalytic Studies on the Interaction between Soft Acid and Hard Base. ChemistrySelect.

[261] Bracho Pozsoni N., Nahra F., van Hecke K., Cazin C. S. J., Nolan S. P. (2024). Impact of the *N*-Heterocyclic
Carbene (NHC) Ligand on the *trans*-[Pd­(NHC)­(NH_2_
^
*n*
^Bu)­Cl_2_] Precatalyst
Architecture in C–N Bond-Forming Reactions. Organometallics.

[262] Liu F., Zhu Y.-R., Song L.-G., Lu J.-M. (2016). Synthesis
of N-Heterocyclic
Carbene–PdCl_2_–(iso)­quinoline Complexes and
Their Application in Arylamination at Low Catalyst Loadings. Org. Biomol. Chem..

[263] Xia Q., Shi S., Gao P., Lalancette R., Szostak R., Szostak M. (2021). [(NHC)­PdCl_2_(Aniline)]
Complexes: Easily Synthesized, Highly Active Pd­(II)–NHC Precatalysts
for Cross-Coupling Reactions. J. Org. Chem..

[264] Hsu Y.-C., Chen M.-T. (2022). N-Heterocyclic Carbene
Palladium­(II)
Amine Complexes: The Role of Primary Aryl- or Alkylamine Binding and
Applications in the Buchwald–Hartwig Amination Reaction. Eur. J. Inorg. Chem..

[265] Fekri S., Mansoori Y., Esquivel D., Navarro M. A. (2023). A New Bis-(NHC)-P­(II)
Complex Supported on Magnetic Mesoporous Silica: An Efficient Pd­(II)
Catalyst for the Selective Buchwald–Hartwig Monoarylation of
Ammonia. ChemistrySelect.

[266] Kaloğlu M., Özdemir I. (2024). Palladium *N*-Heterocyclic
Carbene Pre-catalysts Bearing Different Ancillary Ligands for the
Buchwald–Hartwig Amination of Cyclic Secondary Amines with
Aryl Chlorides. Catal. Lett..

[267] Zhao X.-Y., Zhou Q., Lu J.-M. (2016). Synthesis
and characterization
of N-Heterocyclic Carbene-Palladium­(II) Chlorides-1-Methylindazole
and −1-Methylpyrazole Complexes and Their Catalytic Activity
toward C–N Coupling of Aryl Chlorides. RSC Adv..

[268] Yang J. (2017). Heteroleptic
(N-Heterocyclic Carbene)-Pd-Pyrazole (Indazole) Complexes:
Synthesis, Characterization and Catalytic Activities towards C-C and
C-N Cross-Coupling Reactions. Appl. Organometal
Chem..

[269] Wang T., Xie H., Liu L., Zhao W.-X. (2016). *N*-Heterocyclic Carbene-Palladium­(II)
Complexes with Benzoxazole
or Benzothiazole Ligands: Synthesis, Characterization, and Application
to Suzuki–Miyaura Cross-Coupling Reaction. J. Organomet. Chem..

[270] Wang T., Xu K., Liu L., Xie H., Li Y., Zhao W.-X. (2016). An Easily Available N-Heterocyclic
Carbene–Palladium­(II)
Catalyst for Buchwald–Hartwig Amination of Aryl Chlorides. Transit Met Chem..

[271] Jin Z., Qiu L.-L., Li Y.-Q., Song H.-B., Fang J.-X. (2010). Insight
into the Steric and Electronic Effects of Ancillary Ligands: Synthesis
and Structure-Reactivity Relationship of Well-Defined, Air- and Moisture-Stable
(NHC)­Pd­(sal)Cl Complexes (sal = Salicylaldimine). Organometallics.

[272] Li Y.-J., Zhang J.-L., Li X.-J., Geng Y., Xu X.-H., Jin Z. (2013). (IPr)­Pd­(pydc) (pydc = pyridine-2,6-dicarboxylate)
- A Highly Active Precatalyst for the Sterically Hindered C-N Coupling
Reactions. J. Organomet. Chem..

[273] Zhang Z.-M., Gao Y.-J., Lu J.-M. (2017). Synthesis
of N-Heterocyclic
Carbene-Pd­(II) Complexes and Their Catalytic Activity in the Buchwald–Hartwig
Amination of Aryl Chlorides. Tetrahedron.

[274] Özdemir I., Demir S., Gök Y., Cetinkaya E., Cetinkaya E. (2004). Synthesis of Novel Palladium–Carbene
Complexes as Efficient Catalysts for Amination of Aryl Chlorides in
Ionic Liquid. J. Mol. Catal. A.

[275] Özdemir I., Demir S., Sahin O., Büyükgüngör O., Cetinkaya B. (2010). Palladium
N-Heterocyclic Carbene Complexes: Synthesis,
Characterization and Catalytic Properties in Amination. J. Organomet. Chem..

[276] Doğan Ö., Demir S., Özdemir I., Cetinkaya B. (2011). Palladium­(II)–NHC Complexes Containing Benzimidazole
Ligand as a Catalyst for C–N Bond Formation. Appl. Organometal. Chem..

[277] Karaca E. Ö., Gürbüz N., Sahin O., Büyükgüngör O., Özdemir I. (2016). Synthesis
of Palladium Complexes Derived from Imidazolidin-2-ylidene Ligands
and Used for Catalytic Amination Reactions. Appl. Organometal. Chem..

[278] Demir S., Özdemir I., Cetinkaya B., Arslan H., VanDerveer D. (2011). Synthesis
and Characterization of
Bidentate NHC–Pd Complexes and Their Role in Amination Reactions. Polyhedron.

[279] Liu Y., Voloshkin V. A., Scattolin T., Peng M., Van Hecke K., Nolan S. P., Cazin C. S. J. (2022). Versatile
and Highly Efficient *trans*-[Pd­(NHC)­Cl_2_(DMS/THT)] Precatalysts for
C- N and C- C Coupling Reactions in Green Solvents. Eur. J. Org. Chem..

[280] Yang J., Li P., Zhang Y., Wang L. (2014). A New Library
of Arsine, Stibine-Stabilized N-Heterocyclic Carbene Palladium Complexes:
Synthesis, Structures and Activities in C–C and C–N
Coupling Reactions. Dalton Trans..

[281] Mendoza-Espinosa D., González-Olvera R., Osornio C., Negrón-Silva G.
E., Álvarez-Hernández A., Bautista-Hernández C. I., Suárez-Castillo O. R. (2016). Structural
Diversity of Phenoxy Functionalized Triazol-5-ylidene Palladium­(II)
Complexes and Their Application in C-N Bond Formation. J. Organomet. Chem..

[282] Pirkl N., Del Grosso A., Mallick B., Doppiu A., Gooβen L. J. (2019). Dihalogen-Bridged
NHC–Palladium­(I) Dimers: Synthesis,
Characterisation and Applications in Cross-Coupling Reactions. Chem. Commun..

[283] Zhang Y., Lavigne G., César V. (2015). Buchwald–Hartwig
Amination of (Hetero)­Aryl Tosylates Using a Well-Defined N-Heterocyclic
Carbene/Palladium­(II) Precatalyst. J. Org. Chem..

[284] Duan W., Sun Z., Huo Y., Liu Y., Wu G., Wang R., Wu S., Yao Q., Gong S. (2018). NHC-Pd Complex
Based on 1,3-bis (4-Ethoxycarbonylphenyl)­imidazolium Chloride: Synthesis,
Structure and Catalytic Activity in the Synthesis of Axially Chiral
Benzophenone Hydrazone. Appl. Organometal Chem..

[285] Sugahara T., Murakami K., Yorimitsu H., Osuka A. (2014). Palladium-Catalyzed Amination of Aryl Sulfides with Anilines. Angew. Chem., Int. Ed..

[286] Gao K., Yorimitsu H., Osuka A. (2015). Palladium-Catalyzed Amination of
Aryl Sulfides with Aliphatic Amines. Eur. J.
Org. Chem..

[287] Yang S., Yu X., Poater A., Cavallo L., Cazin C. S. J., Nolan S. P., Szostak M. (2022). Buchwald–Hartwig
Amination and C–S/S–H Metathesis of Aryl Sulfides by
Selective C–S Cleavage Mediated by Air- and Moisture-Stable
[Pd­(NHC)­(*μ*-Cl)­Cl]_2_ Precatalysts:
Unified Mechanism for Activation of Inert C–S Bonds. Org. Lett..

[288] Yoshida Y., Otsuka S., Nogi K., Yorimitsu H. (2018). Palladium-Catalyzed
Amination of Aryl Sulfoxides. Org. Lett..

[289] Chen W., Chen K., Chen W., Liu M., Wu H. (2019). Well-Designed N-Heterocyclic Carbene Ligands for Palladium-Catalyzed
Denitrative C–N Coupling of Nitroarenes with Amines. ACS Catal..

[290] Li G., Zhou T., Poater A., Cavallo L., Nolan S. P., Szostak M. (2020). Buchwald–Hartwig Cross-Coupling
of Amides (transamidation)
by Selective N–C­(O) Cleavage Mediated by Air- and Moisture-stable
[Pd­(NHC)­(allyl)­Cl] Precatalysts: Catalyst Evaluation and Mechanism. Catal. Sci. Technol..

[291] Zhao Q., Meng G., Li G., Flach C., Mendelsohn R., Lalancette R., Szostak R., Szostak M. (2021). IPr# –
Highly Hindered, Broadly Applicable N-Heterocyclic Carbenes. Chem. Sci..

[292] Lei P., Wang Y., Mu Y., Wang Y., Ma Z., Feng J., Liu X., Szostak M. (2021). Green-Solvent Selection
for Acyl Buchwald–Hartwig Cross-Coupling of Amides (Transamidation). ACS Sustainable Chem. Eng..

[293] Shi S., Szostak M. (2017). Pd–PEPPSI: a
General Pd–NHC Precatalyst
for Buchwald–Hartwig Cross-Coupling of Esters and Amides (Transamidation)
under the Same Reaction Conditions. Chem. Commun..

[294] Buchspies J., Rahman M. M., Szostak R., Szostak M. (2020). N-Acylcarbazoles
and N-Acylindoles: Electronically Activated Amides for N–C­(O)
Cross-Coupling by N_lp_ to Ar Conjugation Switch. Org. Lett..

[295] Zhou T., Li G., Nolan S. P., Szostak M. (2019). [Pd­(NHC)­(acac)­Cl]:
Well-Defined, Air-Stable, and Readily Available Precatalysts for Suzuki
and Buchwald–Hartwig Cross-coupling (Transamidation) of Amides
and Esters by N–C/O–C Activation. Org. Lett..

[296] Zhu Y., Yang S., Zhou T., Szostak M. (2024). [(NHC)­Pd­(OAc)_2_]: Highly Active Carboxylate Pd­(II)–NHC (NHC = N**-**Heterocyclic Carbene) Precatalysts for Suzuki–Miyaura and
Buchwald–Hartwig Cross-Coupling of Amides by N–C­(O)
Activation. J. Org. Chem..

[297] Ben Halima T., Vandavasi J. K., Shkoor M., Newman S. G. (2017). A Cross-Coupling
Approach to Amide Bond Formation from Esters. ACS Catal..

[298] Dardir A. H., Melvin P. R., Davis R. M., Hazari N., Mohadjer
Beromi M. (2018). Rapidly Activating Pd-Precatalyst for Suzuki–Miyaura
and Buchwald–Hartwig Couplings of Aryl Esters. J. Org. Chem..

[299] De la Fuente-Olvera A. A., Suárez-Castillo O. R., Mendoza-Espinosa D. (2019). Synthesis and Catalytic Applications of Palladium­(II)
Complexes Supported by Hydroxyl-Functionalized Triazolylidenes. Eur. J. Inorg. Chem..

[300] Desmarets C., Schneider R., Fort Y. (2002). Nickel­(0)/Dihydroimidazol-2-ylidene
Complex Catalyzed Coupling of Aryl Chlorides and Amines. J. Org. Chem..

[301] Omar-Amrani R., Thomas A., Brenner E., Schneider R., Fort Y. (2003). Efficient Nickel-Mediated Intramolecular Amination of Aryl Chlorides. Org. Lett..

[302] Kuhl S., Fort Y., Schneider R. (2005). Nickel­(0)/N-Heterocyclic
Carbene Complexes Catalysed Arylation of Aromatic Diamines. J. Organomet. Chem..

[303] Chen C., Yang L. M. (2007). Ni­(II)-(σ-aryl)
Complex: a
Facile, Efficient Catalyst for Nickel-Catalyzed Carbon-Nitrogen Coupling
Reactions. J. Org. Chem..

[304] Fan X.-H., Li G., Yang L.-M. (2011). Room-Temperature
Nickel-Catalyzed Amination of Heteroaryl/Aryl Chlorides with Ni­(II)-(σ-Aryl)
Complex as Precatalyst. J. Organomet. Chem..

[305] Wu W., Fan X.-H., Zhang L.-P., Yang L.-M. (2014). Nickel-Catalyzed
N-Arylation of Benzophenone Hydrazone with Bromoarenes. RSC Adv..

[306] Nattmann L., Saeb R., Nöthling N., Cornella J. (2020). An Air-Stable Binary
Ni(0)-Olefin Catalyst. Nat. Catal..

[307] Wang Z.-C., Xie P.-P., Xu Y., Hong X., Shi S.-L. (2021). Low-Temperature Nickel-Catalyzed
C–N Cross-Coupling
via Kinetic Resolution Enabled by a Bulky and Flexible Chiral N-Heterocyclic
Carbene Ligand. Angew. Chem., Int. Ed..

[308] Wang Z.-C., Li Y.-Y., Zhang S.-Q., Hong X., Shi S.-L. (2023). Unsymmetric N-Heterocyclic Carbene
Ligand Enabled Nickel-Catalysed
Arylation of Bulky Primary and Secondary Amines. Chem. Sci..

[309] Tassone J. P., Lundrigan T., Ashton T. D., Stradiotto M. (2022). Nickel-Catalyzed
C-N Cross-Coupling of 4-Chloro-1,8-naphthalimides and Bulky, Primary
Alkylamines at Room Temperature. J. Org. Chem..

[310] Khazipov O. V., Pyatachenko A. S., Chernyshev V. M., Ananikov V. P. (2023). A Simple Protocol for the C–
N Cross-Coupling
of Aryl Chlorides with Amines Applying Ni/NHC Catalysis. ChemCatChem..

[311] Matsubara K., Miyazaki S., Koga Y., Nibu Y., Hashimura T., Matsumoto T. (2008). An Unsaturated Nickel(0) NHC Catalyst:
Facile Preparation and Structure of Ni(0)­(NHC)_2_, Featuring
a Reduction Process from Ni­(II)­(NHC)­(acac)_2_. Organometallics.

[312] Iglesias M. J., Blandez J. F., Fructos M. R., Prieto A., Álvarez E., Belderrain T. R., Nicasio M. C. (2012). Synthesis, Structural
Characterization, and Catalytic Activity of IPrNi­(styrene)_2_ in the Amination of Aryl Tosylates. Organometallics.

[313] Rull S. G., Blandez J. F., Fructos M. R., Belderrain T. R., Nicasio M. C. (2015). C-N Coupling of Indoles and Carbazoles
with Aromatic
Chlorides Catalyzed by a Single-Component NHC-Nickel(0) Precursor. Adv. Synth. Catal..

[314] Crawford S. M., Lavery C. B., Stradiotto M. (2013). BippyPhos:
A Single Ligand With Unprecedented Scope in the Buchwald–Hartwig
Amination of (Hetero)­aryl Chlorides. Chem. -
Eur. J..

[315] Rull S. G., Funes-Ardoiz I., Maya C., Maseras F., Fructos M. R., Belderrain T. R., Nicasio M. C. (2018). Elucidating the
Mechanism of Aryl Aminations Mediated by NHC-Supported Nickel Complexes:
Evidence for a Nonradical Ni(0)/Ni­(II) Pathway. ACS Catal..

[316] Nett A. J., Cañellas S., Higuchi Y., Robo M. T., Kochkodan J. M., Haynes M. T., Kampf J. W., Montgomery J. (2018). Stable, Well-Defined
Nickel(0) Catalysts for Catalytic
C–C and C–N Bond Formation. ACS
Catal..

[317] Kelly R. A., Scott N. M., Díez-González S., Stevens E. D., Nolan S. P. (2005). Simple Synthesis of CpNi­(NHC)Cl Complexes
(Cp = Cyclopentadienyl; NHC = N-Heterocyclic Carbene). Organometallics.

[318] Martin A. R., Makida Y., Meiries S., Slawin A. M. Z., Nolan S. P. (2013). Enhanced
Activity of [Ni­(NHC)­CpCl] Complexes in Arylamination
Catalysis. Organometallics.

[319] Matsubara K., Ueno K., Koga Y., Hara K. (2007). Nickel–NHC-Catalyzed
α-Arylation of Acyclic Ketones and Amination of Haloarenes and
Unexpected Preferential N-Arylation of 4-Aminopropiophenone. J. Org. Chem..

[320] Matsubara K., Ueno K., Shibata Y. (2006). Synthesis and Structures
of Nickel Halide Complexes Bearing Mono- and Bis-coordinated N-Heterocyclic
Carbene Ligands, Catalyzing Grignard Cross-Coupling Reactions. Organometallics.

[321] Iglesias M. J., Prieto A., Nicasio M. C. (2010). Well-Defined Allylnickel
Chloride/*N*-Heterocyclic Carbene [Ni­(NHC)­(Allyl)­Cl]
Complexes as Highly Active Precatalysts for C–N and C–S
Cross-Coupling Reactions. Adv. Synth. Catal..

[322] Dible B. R., Sigman M. S. (2003). Unusual Reactivity
of Molecular Oxygen
with π-Allylnickel­(N-Heterocyclic Carbene) Chloride Complexes. J. Am. Chem. Soc..

[323] Martin A. R., Nelson D. J., Meiries S., Slawin A. M. Z., Nolan S. P. (2014). Efficient
C-N and C-S Bond Formation Using the Highly
Active [Ni­(allyl)­Cl­(IPr*OMe)] Precatalyst. Eur.
J. Org. Chem..

[324] Nirmala M., Prakash G., Ramachandran R., Viswanathamurthi P., Malecki J. G., Linert W. (2015). Nickel­(II) Complex
Incorporating Methylene Bridged Tetradentate Dicarbene Ligand as an
Efficient Catalyst toward C-C and C-N Bond Formation Reactions. J. Mol. Catal. A: Chem..

[325] Ibrahim H., Bala M. D. (2016). Earth Abundant Metal Complexes of
Donor Functionalised N-Heterocyclic Carbene Ligands: Synthesis, Characterisation
and Application as Amination Catalysts. New
J. Chem..

[326] Rull S. G., Rama R. J., Alvarez E., Fructos M. R., Belderrain T. R., Nicasio M. C. (2017). Phosphine-Functionalized NHC Ni­(II)
and Ni(0) Complexes: Synthesis, Characterization and Catalytic Properties. Dalton Trans..

[327] Nirmala M., Saranya G., Viswanathamurthi P., Bertani R., Sgarbossa P., Malecki J. G. (2017). Organonickel Complexes
Encumbering Bis-Imidazolylidene Carbene Ligands: Synthesis, X-ray
Structure and Catalytic Insights on Buchwald–Hartwig Amination
Reactions. J. Organomet. Chem..

[328] Strieth-Kalthoff F., Longstreet A. R., Weber J. M., Jamison T. F. (2018). Bench-Stable *N*-Heterocyclic
Carbene Nickel Precatalysts for C–C
and C–N Bond-Forming Reactions. ChemCatChem..

[329] Matsubara K., Fukahori Y., Inatomi T., Tazaki S., Yamada Y., Koga Y., Kanegawa S., Nakamura T. (2016). Monomeric
Three-Coordinate N-Heterocyclic Carbene Nickel­(I) Complexes: Synthesis,
Structures, and Catalytic Applications in Cross-Coupling Reactions. Organometallics.

[330] Nagao S., Matsumoto T., Koga Y., Matsubara K. (2011). Monovalent
Nickel Complex Bearing a Bulky *N*-Heterocyclic Carbene
Catalyzes Buchwald–Hartwig Amination of Aryl Halides under
Mild Conditions. Chem. Lett..

[331] Nagahora N., Goto S., Inatomi T., Tokumaru H., Matsubara K., Shioji K., Okuma K. (2018). Buchwald–Hartwig
Amination of Phosphinines and the Effect of Amine Substituents on
Optoelectronic Properties of the Resulting Coupling Products. J. Org. Chem..

[332] Inatomi T., Fukahori Y., Yamada Y., Ishikawa R., Kanegawa S., Koga Y., Matsubara K. (2019). Ni­(I)–Ni­(III)
Cycle in Buchwald–Hartwig Amination of Aryl Bromide Mediated
by NHC-Ligated Ni­(I) Complexes. Catal. Sci.
Technol..

[333] Gao C. Y., Yang L. M. (2008). Nickel-Catalyzed Amination of Aryl
Tosylates. J. Org. Chem..

[334] Jiang J., Zhu H., Shen Y., Tu T. (2014). Acenaphthoimidazolium
Chloride-Enabled Nickel-Catalyzed Amination of Bulky Aryl Tosylates. Org. Chem. Front..

[335] Hie L., Ramgren S. D., Mesganaw T., Garg N. K. (2012). Nickel-Catalyzed
Amination of Aryl Sulfamates and Carbamates Using an Air-Stable Precatalyst. Org. Lett..

[336] Fine
Nathel N. F., Kim J., Hie L., Jiang X., Garg N. K. (2014). Nickel-Catalyzed Amination of Aryl Chlorides and Sulfamates
in 2-Methyl-THF. ACS Catal..

[337] Wiensch E. M., Montgomery J. (2018). Nickel-Catalyzed
Amination of Silyloxyarenes
through C-O Bond Activation. Angew. Chem., Int.
Ed..

[338] Tobisu M., Shimasaki T., Chatani N. (2009). Ni^0^-Catalyzed
Direct Amination of Anisoles Involving the Cleavage of Carbon–Oxygen
Bonds. Chem. Lett..

[339] Tobisu M., Yasutome A., Yamakawa K., Shimasaki T., Chatani N. (2012). Ni­(0)/NHC-Catalyzed Amination of *N*-Heteroaryl Methyl Ethers through the Cleavage of Carbon–Oxygen
Bonds. Tetrahedron.

[340] Shimasaki T., Tobisu M., Chatani N. (2010). Nickel-Catalyzed
Amination
of Aryl Pivalates by the Cleavage of Aryl C-O Bonds. Angew. Chem., Int. Ed..

[341] Mesganaw T., Silberstein A. L., Ramgren S. D., Nathel N. F. F., Hong X., Liu P., Garg N. K. (2011). Nickel-Catalyzed
Amination of Aryl Carbamates and Sequential Site-Selective Cross-Couplings. Chem. Sci..

[342] Ramgren S. D., Silberstein A. L., Yang Y., Garg N. K. (2011). Nickel-Catalyzed
Amination of Aryl Sulfamates. Angew. Chem.,
Int. Ed..

[343] Huang J.-H., Yang L.-M. (2011). Nickel-Catalyzed Amination of Aryl
Phosphates through Cleaving Aryl C–O Bonds. Org. Lett..

[344] Zhu F., Wang Z. X. (2013). Nickel-Catalyzed Coupling of Fluoroarenes and Amines. Adv. Synth. Catal..

[345] Liu X., Yue H., Jia J., Guo L., Rueping M. (2017). Synthesis
of Amidines from Amides Using a Nickel-Catalyzed Decarbonylative Amination
through CO Extrusion Intramolecular Recombination Fragment Coupling. Chem. -Eur. J..

[346] Liu J., Jia X., Chen X., Sun H., Li Y., Kramer S., Lian Z. (2020). Nickel-Catalyzed Intramolecular Desulfitative
C—N Coupling: A Synthesis of Aromatic Amines. J. Org. Chem..

[347] Hie L., Fine Nathel N. F., Hong X., Yang Y.-F., Houk K. N., Garg N. K. (2016). Nickel-Catalyzed Activation of Acyl
C–O Bonds of Methyl Esters. Angew. Chem.,
Int. Ed..

[348] Ben Halima T., Masson-Makdissi J., Newman S. G. (2018). Nickel-Catalyzed
Amide Bond Formation from Methyl Esters. Angew.
Chem., Int. Ed..

[349] Ji C. L., Xie P. P., Hong X. (2018). Computational Study
of Mechanism and Thermodynamics of Ni/IPr-Catalyzed Amidation of Esters. Molecules.

[350] Zheng Y.-L., Newman S. G. (2019). Methyl Esters as Cross-Coupling Electrophiles:
Direct Synthesis of Amide Bonds. ACS Catal..

[351] Baker E. L., Yamano M. M., Zhou Y., Anthony S. M., Garg N. K. (2016). A Two-Step Approach to Achieve Secondary
Amide Transamidation
Enabled by Nickel Catalysis. Nat. Commun..

[352] Dander J. E., Baker E. L., Garg N. K. (2017). Nickel-Catalyzed
Transamidation of Aliphatic Amide Derivatives. Chem. Sci..

[353] Buchspies J., Rahman M. M., Szostak M. (2021). Transamidation of Amides
and Amidation of Esters by Selective N–C­(O)/O–C­(O) Cleavage
Mediated by Air- and Moisture-Stable Half-Sandwich Nickel­(II)–NHC
Complexes. Molecules.

[354] Ibrahim H., Bala M. D. (2015). Air Stable Pincer
(CNC) N-Heterocyclic
Carbene–Cobalt Complexes and Their Application as Catalysts
for C–N Coupling Reactions. J. Organomet.
Chem..

[355] Kim M., Chang S. (2010). Rhodium­(NHC)-Catalyzed Amination of Aryl Bromides. Org. Lett..

